# Characterization of Chikungunya Virus Induced Host Response in a Mouse Model of Viral Myositis

**DOI:** 10.1371/journal.pone.0092813

**Published:** 2014-03-25

**Authors:** Rekha Dhanwani, Mohsin Khan, Vinay Lomash, Putcha Venkata Lakshmana Rao, Hinh Ly, Manmohan Parida

**Affiliations:** 1 Department of Virology, Defence Research & Development Establishment (DRDE), Gwalior, India; 2 Department of Pharmacology and Toxicology, Defence Research & Development Establishment (DRDE), Gwalior, India; 3 DRDO-BU Center for Life Sciences, Bharathiar University Campus, Coimbatore, India; 4 Department of Veterinary and Biomedical Sciences, University of Minnesota, Twin Cities, Saint Paul, Minnestoa, United States of America; German Primate Center, Germany

## Abstract

While a number of studies have documented the persistent presence of chikungunya virus (CHIKV) in muscle tissue with primary fibroblast as the preferable cell target, little is known regarding the alterations that take place in muscle tissue in response to CHIKV infection. Hence, in the present study a permissive mouse model of CHIKV infection was established and characterized in order to understand the pathophysiology of the disease. The two dimensional electrophoresis of muscle proteome performed for differential analysis indicated a drastic reprogramming of the proteins from various classes like stress, inflammation, cytoskeletal, energy and lipid metabolism. The roles of the affected proteins were explained in relation to virus induced myopathy which was further supported by the histopathological and behavioural experiments proving the lack of hind limb coordination and other loco-motor abnormalities in the infected mice. Also, the level of various pro-inflammatory mediators like IL-6, MCP-1, Rantes and TNF-α was significantly elevated in muscles of infected mice. Altogether this comprehensive study of characterizing CHIKV induced mouse myopathy provides many potential targets for further evaluation and biomarker study.

## Introduction

Chikungunya fever (CHIKF) is an enervating, but non-fatal, arthropod-borne disease transmitted to humans by the bite of infected *Aedes aegypti* and *Aedes albopictus* mosquitoes. It is a debilitating viral illness of global concern due to its escalating outbreaks in different parts of the world particularly in Africa, Europe and South East Asia [1]–[3]. There have been a number of epidemics associated with severe morbidity in Philippines, Thailand, Cambodia, Vietnam, India, Myanmar, Sri Lanka and on the islands of the Indian Ocean, including Madagascar, Comoros, Mauritius, and Reunion Island [4]–[6].

Chikungunya virus (CHIKV), an *Alphavirus* belonging to the family *Togaviridae*, is an enveloped virus with a positive sense RNA of approximately 11.8 kb. The virus is responsible for an acute infection characterized by high fever, arthralgia, myalgia, head-ache, chills, photophobia and rash [7]–[9]. However, the infection is classically self- limiting and usually resolves within 3-4 days except for the joint symptoms that may persist for a longer period. Despite the pathological significance of chikungunya, the physiological and molecular mechanisms occurring during viral infection are still not well defined. With respect to the alphavirus induced myopathy, Ross River Virus (RRV) pathogenesis has been studied in detail. The arthritic disease is thought to be initiated by viral replication and inflammatory infiltrates that is comprised of monocytes/macrophages in the affected joints [10], [11], [12]. Like other alphaviruses of old world class, CHIKV is also primarily arthritogenic in nature and muscle tissue has been proposed to be their target [13]–[17]. Studies done on animal models have identified muscle fibres and/or infiltrating cells as the cellular target of alphavirus infection. However, a study done on muscle biopsies of CHIKV infected patients with myositic syndrome has identified muscle satellite cells and not muscle fibres as the primary target of viral infection [18]. Recently, evidence of arthritis, tenosynovitis, and myositis in CHIKV infected neonatal and adult mice of different strains has been reported by several groups [19]–[25].

It is important to investigate the changes that occur in response to the infection in laboratory animal models that can simulate clinical infection in humans. Viral infections generally result in alterations in the host proteome that can determine the fate of the infected host cells or tissues, which can impact the disease progression and outcome. Hence, in order to uncover the host protein responses to CHIKV infection, a comprehensive study of interaction between CHIKV and a permissive mouse model was undertaken. In one of our previous studies, protein profiling of CHIKV infected mouse liver and brain tissues was reported explaining the implications of differentially affected proteins in disease manifestation [26]. The neonatal mice (2–3 day old) serve as a useful system for understanding the arthritic manifestations and identify host factors that may contribute to CHIKV induced inflammation and myositis. Using two dimensional electrophoresis (2-DE) method, the differential proteome analysis of muscle tissue was undertaken. The roles of the affected proteins were explained in relation to virus induced myopathy which was further supported by the histopathological and behavioural experiments proving the lack of hind limb coordination and other loco-motor abnormalities in the infected mice. Altogether this comprehensive study of characterizing CHIKV induced mouse myopathy provides many potential targets for further evaluation and biomarker study.

## Materials and Methods

### Virus propagation and titration

An Indian strain of CHIKV DRDE-06 (Genbank accession no: EF- 210157) of ECSA genotype was used in the present study [27]. The virus was isolated during 2006 epidemic from a serologically confirmed human patient. The clinical virus isolate was cultured in BHK-21 cells and further passaged in C_6/36_ cell line to increase the viral titre. The virus stock was harvested from the culture supernatant and stored at −80°C. CHIKV was titrated by plaque formation on monolayers of Vero cells (obtained from NCCS, Pune, India).

### Ethics statement

All animal experiments were carried out strictly following the approved study protocol of Institutional Animal Ethics Committee (IAEC) of the Defence Research and Development Establishment (DRDE) Gwalior, India (Protocol Number: Viro-07/50/MMP). The institutional committee is registered with committee for the purpose of control and supervision of experiments on animals (CPCSEA), Ministry of Environment and Forestry, Government of India (Regd. No – 37/GO/c/1999/CPCSEA). Balb/c mice of 2–3 days of age along with their mothers, obtained from the institutional animal facility, were housed in polypropylene cages with dust-free rice husk as bedding material at an ambient temperature of 25–26°C with a standard 12-hour light/12-hour dark cycle and the mothers were allowed ad libitum access to food and water while the pups were allowed to suckle freely.

### Animal experimentation

Mice (n = 8) were inoculated subcutaneously with CHIKV in the loose skin on their back (10^6^ pfu in 50 ul of infected C_6/36_ cell culture supernatant). In parallel, mock-infected group (n = 8) was inoculated with uninfected C_6/36_ cell supernatant. Following virus inoculation, the mice were monitored at 24 hour interval for disease signs like retarded growth, hair loss, skin rashes and hind limb paralysis. The clinical signs of the disease were also determined by behavioural parameters of surface righting reflex and walking traits. For surface righting reflex, mice pups were placed on their back and were allowed some time to turn over to upright position.

Clinical scoring of the mice inoculated with CHIKV was done as follows: 0, no disease signs; 1, ruffled fur; 2, mild hind limb weakness; 3, moderate hind limb weakness; 4, severe hind limb weakness and dragging and 5, moribund. The clinical score of 5 was set as the terminal point. When the mice inoculated with CHIKV reached the terminal point, they were euthanized by cervical dislocation and tissues were processed for further studies.

### Determination of viral titre

To determine the viral titre in muscles, mice were perfused with PBS and hind limbs were removed by dissection. Upon removal of bone from hind limbs, tissue was homogenized in 1× minimum essential media (MEM) and viral RNA was extracted using Qiagen kit according to manufacturer's protocol. Viral presence in muscle was assessed by SYBR green I based one-step real-time quantitative RT-PCR using primers specific to a region of 200 base pairs of the viral envelope (E1) gene. Standard curve was constructed with Ct (threshold cycle) values obtained using dilutions of the titrated CHIKV virus, and genome equivalent of CHIKV in muscle was determined from respective Ct values obtained from standard curve as reported [28].

### Histopathology and histochemistry

At necropsy, skin, spleen and hind limbs from each mouse were cut and fixed in 10% neutral buffered formalin. After 48 h of fixation, muscles were transferred to 70% ethanol, processed by automated tissue processor (Leica TP1020), dehydrated and embedded in paraffin wax. Multiple sections of 12 μm thickness were prepared using automatic microtome (Microm HM360) and stained with hematoxylin and eosin in Leica Autostain-XL. Muscle sections of 4 μm thickness were used for the localization of viral antigen. Anti-CHIKE2 antibody raised in rabbit immunized with purified recombinant envelope protein (rCHIKE2) was used to recognize the CHIKV antigen.

### Protein extraction

During the peak symptomatic phase (i.e 9–10 days post infection), when the animals were in moribund condition with severe paralysis, four of the suckling mice (n = 4) from each group (mock-infected and CHIKV-infected) were dissected and tissues were frozen in liquid nitrogen and stored at −80°C until further processed for proteome analysis.

Tissue samples were homogenized according to a previously described method with some modifications [29]. Briefly, the muscle tissue was thawed on ice, separated from the bones using scalpel and combined with an equivalent volume of solubilization buffer [7M urea, 2% CHAPS, 1× concentration of protease inhibitor cocktail (Sigma, St. Louise, MO, USA)]. Thereafter, the tissue was continuously homogenized for 3 min on ice using Tissue ruptor (Qiagen, Hilden, Germany). The clear supernatant was collected by centrifugation and subjected to five pulses of sonication on ice. Total soluble protein fraction was recovered by centrifugation at 40,000×g for 30 min and the protein concentration was determined using Bradford reagent (Sigma, USA). The solubilized protein in the supernatant was then precipitated with 10% (wt/vol) trichloroacetic acid, kept at 4°C overnight and centrifuged at 10,000×*g* for 10 min at 4°C. The resulting protein precipitate was washed twice with cold acetone containing 0.07% β-mercapto-ethanol, air-dried, and stored at −80°C until use.

### Two dimensional electrophoresis (2-DE) and image analysis

2-DE was performed using 7 cm Readystrip IPG strips (Linear, pI 4–7, Biorad, Hercules, CA) in the PROTEAN IEF Cell and PROTEAN plus Dodeca Cell (Biorad). Before proceeding for isoelectric focusing, the IPG strips were passively rehydrated for 16 hours with 150 μl of rehydration buffer (8 M urea, 2% CHAPS, 15 mM DTT and 0.5% v/v IPG buffer pH 3–10) which contained 500 μg of protein. The isoelectric focusing of the rehydrated strips was automatically processed using the following parameters: 250 V rapid, 15 min; 4000 V rapid 2 h; 8000–10000 Vh at 20°C under mineral oil. After focusing, the strips were incubated for 10 min in equilibration buffer (6 M urea, 30% w/v glycerol, 2% w/v SDS and 0.375 M Tris/HCl buffer, pH 8.8) containing 1% w/v DTT, followed by additional equilibration for 15 min in equilibration buffer containing 4% w/v iodoacetamide. The equilibrated strips were then further resolved with 12% SDS PAGE gels keeping constant current of 10 mA per gel until the dye front reached the bottom of the gel. Gels were then stained with Coomassie Brilliant Blue G-250 and scanned at 300 dpi using GS800 densitometer (Biorad). Comparative analysis of protein spots was performed using PD Quest 2D analysis software (Biorad). The gels were normalized according to the total quantity in the analysis set. The spots were checked manually to eliminate any possible artifacts and spots that were consistently reproducible in all gel images, including both the biological and technical replicates, were chosen for subsequent analysis. The student's *t*-test was used for statistical analysis of data. Expression intensity ratios of CHIKV-infected/mock-infected values greater than 1.8 (p≤0.05) or less than 0.5 (p≤0.05) were set as the thresholds to indicate significant changes. Two such independent experiments were carried out. In each experiment a total of 8 gels derived from pooled protein samples of four animals from each group were analysed.

### Identification of differentially expressed proteins by MALDI-TOF/TOF Mass Spectrometry and database search

The protein spots of interest were manually excised from the coomassie stained gels and subjected to ingel digestion as previously described [30]. Tryptic digests were extracted using 10 μL of Milli-Q water initially, followed by two extractions with a total of 20 μL of 0.1% TFA. The combined extracts were dried in a vacuum concentrator at room temperature, and then dissolved in 1 μL of 5% acetonitrile with 0.5% TFA. The prepared MS samples were mixed with equal volume of the α-cyano-4-hydroxycinnamic acid (CHCA) matrix solution (10 mg/mL) and then spotted onto the MALDI plate. Samples were allowed to air dry and analysed by a 4800 MALDI-TOF-TOF Analyzer (Applied Biosystems, Framingham, MA). A default calibration was applied using a six component peptide standard spotted onto 13 calibration spot spread on the plate and four corners of 384 well MALDI plate. The UV laser was operated at a 200-Hz repetition rate at a wavelength of 355 nm. The accelerated voltage was operated at 20 kV. The MS/MS mass spectra were acquired by the data dependent acquisition method with the 20 strongest precursors selected from one MS scan. All MS and MS/MS spectra were obtained by accumulation of at least 800 and 2100 laser shots, respectively. MS and MS/MS data were analysed and peak list were generated using Protein Pilot 2.0 (Applied Biosystems). Twenty MS peaks were selected between 850 and 4000 Da and filtered with a signal-to-noise ratio greater than 20. A peak intensity filter was used with no more than 50 peaks per 200 Da in the setting parameter of MASCOT search after acquisition. MS/MS peaks were selected based on a signal-to-noise ratio greater than 10 over a mass range of 60 to 20 Da below the precursor mass. MS and MS/MS were analysed using MASCOT 2.0 search engine (Matrix Science, London, UK) to search against the MSDB protein sequence database in the taxonomy group of *Mus musculus*. Searching parameters were as follows: trypsin digestion with one missed cleavage, variable modifications (oxidation of methionine and carbamidomethylation of cysteine), and the peptide mass tolerance of 50 ppm for precursor ion and mass tolerance of ±0.6 for fragment ion for +1 charged state. For all proteins successfully identified by Peptide Mass Fingerprint and/or MS/MS, Mascot score greater than 62 was accepted as significant (*p* value <0.05).

### Real-Time qRT-PCR

Total RNA was extracted using the Qiagen (GmbH, Hilden, Germany) RNAEasy Mini kit. The quantitative real-time RT-PCR was carried out for the analysis of host gene expression in muscles using gene-specific primers from Quanti Tect primer assay kit and Quanti Fast one-step RT-PCR kit (Qiagen, Germany). The thermal profile consists of 10 min of reverse transcription at 50°C one cycle and 5 min of polymerase activation at 95°C, followed by 40 cycles of PCR at 95°C for 10 s, 60°C for 30 s for combined annealing/extension. Following amplification, a melting curve analysis was performed to verify the authenticity of the amplified product by its specific melting temperature (*T*m) with the melting curve analysis software of the Mx3005p. The threshold cycle (*C*t) of gene of interest (GOI) and housekeeping gene (HKG) and the difference between their *C*t values (*C*t) were determined. Relative changes of gene expression were calculated using delta delta Ct method [31] and the data were represented as fold up-regulation/down-regulation compared to the mock-infected group.

### Western blot analysis

Total protein was extracted from hind limb muscle tissues of the mock and CHIKV infected animals after 9 days post infection. Muscle tissue was homogenized in lysis buffer (Tris-Cl 50 mM (pH 7.4), NaCl 150 mM, EDTA 1 mM, Triton X-100 1%, SDS 0.1%, sodium deoxycholate 1%, Na_3_VO_4_ 1 mM) and centrifuged at 12000×g for 20 min. Protein concentration was quantified in supernatants by Bradford reagent. Equivalent amounts of the tissue lysates (40 μg) were subjected to 12% SDS-PAGE and then transferred to PVDF–immobilon membrane (Millipore, Bedford, MA, USA), incubated with respective primary antibodies (1∶1000) at 4°C overnight, thoroughly washed and incubated with the recommended horseradish peroxidise (HRP) conjugated secondary antibodies (1∶30000) at 37°C for 1 hr. The bound antibodies were detected using chemiluminiscence kit (Life technologies, Carlsbad, CA, USA).

### Statistical analysis

For the statistical analysis of 2-DE gels, student's unpaired *t*-test was done. The data were expressed as mean±SE of four gels per group. Expression intensity ratios of CHIKV-infected/mock-infected values greater than 1.8 (p≤0.05) or less than 0.5 (p≤0.05) were set as the thresholds to indicate significant changes. Two such independent experiments were carried out. In each experiment, a total of 8 gels derived from pooled protein samples of four animals from each group were analysed.

For all other experiments, the data were expressed as mean±SE of three animals per group. The level of significance was set at p<0.05. All experiments were repeated at least twice.

## Results and Discussion

### Animal experimentation

Among the available CHIKV disease animal models, newborn mice represent an ideal model to unravel the host response during CHIKV infection because CHIKV-infected newborn mice exhibit muscle and joint pathologies similar to those observed in humans. Although CHIKV infected suckling mice exhibit 10–20% mortality, majority of the animals eventually recover from the disease with serum virus clearance after 12 days of infection except in the muscles where the viral antigen may persist for a longer period [19], [20]. The neurotropic and neuroinvasive nature of CHIKV has also been explored in different strains of newborn mice [20], [26]. The behavioral traits, loco-motor abnormalities and microscopic observations reported in this study collectively supported the fact that CHIKV infected newborn mice clinically mimic human CHIKV patients in terms of symptoms and disease progression. Based on the clinical similarity between CHIKV infected patients and newborn suckling mice, we aim to explore the host cellular pathophysiological processes that occur during viral infection.

**Figure 1:**
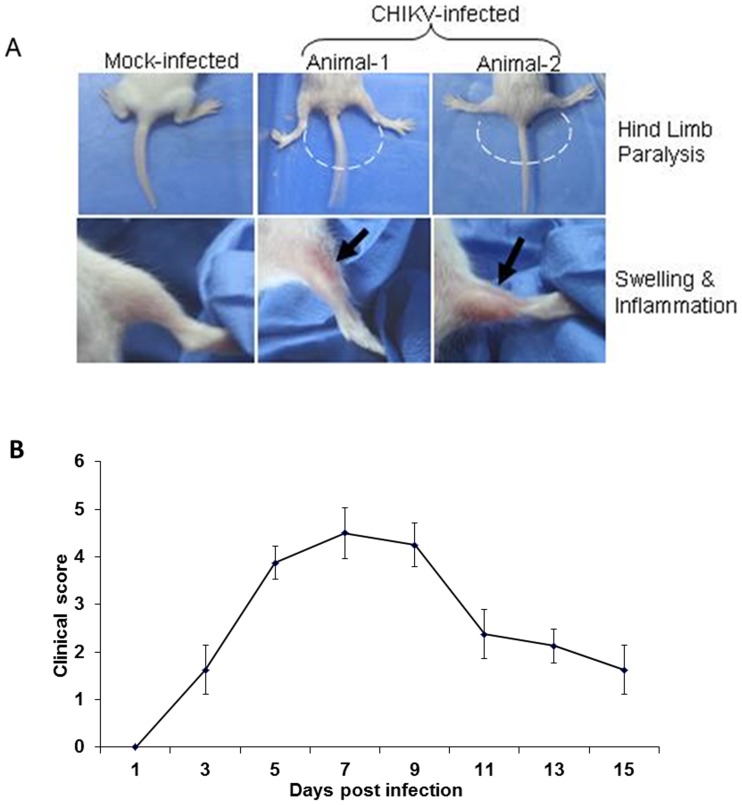
Chikungunya virus (CHIKV) induced disease signs. A. Pictures of the hind limbs of mock- and CHIKV-infected animals. Chikungunya virus infection induced severe hind limb disease in new born mice. 2–3 day old mice inoculated with 10^6^ PFU (50 ul) of CHIKV by subcutaneous injection in the loose fold of skin on the back of the animal developed peak clinical signs on 8 day post inoculation whereas mock-infected group injected with uninfected tissue culture supernatant remained healthy. B. Mice were scored for the development of hind limb dysfunction and disease based on the following scale: 0, no disease signs; 1, ruffled fur; 2, mild hind limb weakness; 3, moderate hind limb weakness; 4, severe hind limb weakness and dragging and 5, moribund. Each data point represents the arithmetic mean ± SD for eight animals. Data is representative of three independent experiments.

The CHIKV infected new-born mice developed symptoms, such as retarded growth, alopecia, lethargy, dragging of hind limbs on 8 day post infection (dpi). The animals developed stiffness in the hind limbs as they failed to bend their limbs at the joints (Figure 1A). The loco-motor behaviour of CHIKV infected mice was severely affected and exhibited a loss of coordination. When tested for surface righting reflex, the infected animals showed difficulty in turning back to the upright position confirming loco-motor disturbance of the hind limbs. The video clips demonstrating the walking traits and surface righting reflex of CHIKV infected mice are provided as supplementary materials (Movie S1, S2 and S3). In addition, the average walking stride length of CHIKV infected mice was significantly decreased. The details of this parameter are given in Figure S1. These data implicate CHIKV induced severe myopathic symptoms in infected new-born mice.

**Figure 2:**
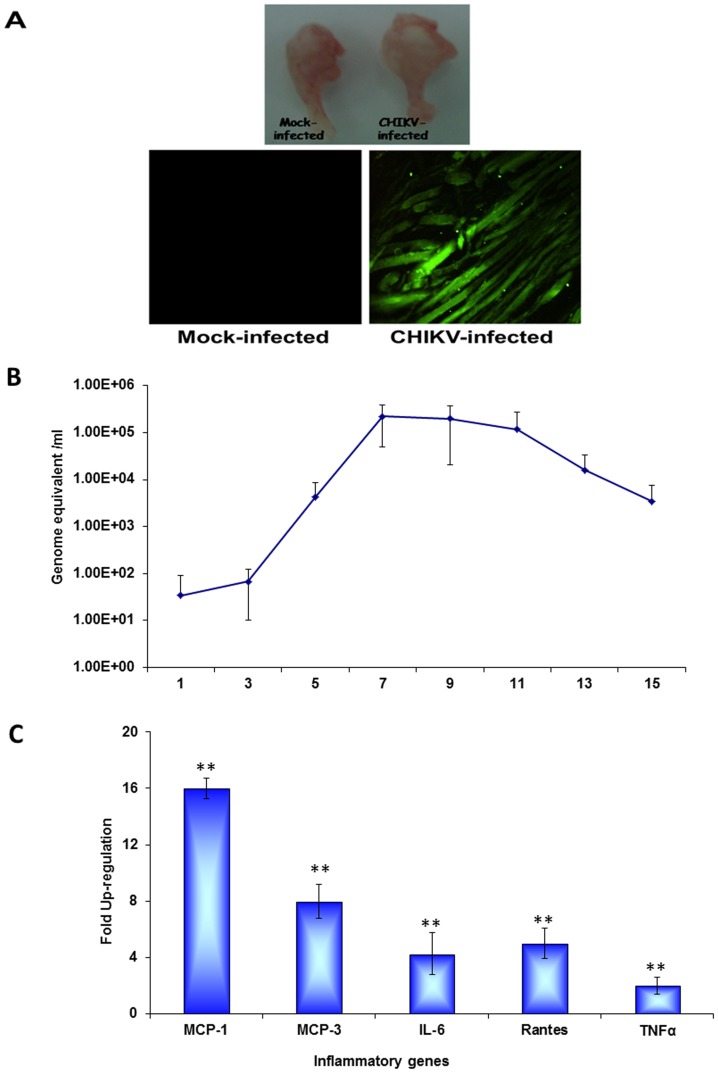
Gross pathology, viral replication and profile of inflammatory cytokines. A. Surgically removed hind limb muscles from mock- and CHIKV-infected mice showing gross pathology of the muscles (in terms of swelling) along with the 4 μm sections showing the localization of CHIKV antigen in the hind limb skeletal muscle on day 9 post infection. B. Virus titre in the hind limb muscles. C. Real time analysis showing relative fold change in inflammatory genes (MCP-1, MCP-3, IL-6, TNF-α, Rantes) in muscle tissue on day 9 post infection. ** Genes were considered significantly up-regulated if the change in their relative expression level was ≥2 fold at p<0.05 by student's *t* test.

It was also noted that the severity of the symptoms subsided at 12 dpi. With this observation, all subsequent experiments were conducted when animals exhibited peak clinical signs between 8–9 dpi. The disease severity in terms of clinical score is depicted in Figure 1B. The gross pathology of the CHIKV infected muscle showed swelling due to possibly to overt inflammation of the tissue (Figure 1A). Using immunoflourescence analysis, we showed that high levels of CHIKV antigen were localised in sections of muscle tissue at 9 dpi (Figure 2A). The muscle sections showing the localization of CHIKV antigen by immunofluorescence at different days of infection are provided as Figure S2. Viral load in muscles was determined on different days post infection to indicate that the virus titre peaked at day 7 post infection and started to decline at day 11 (Figure 2B). In order to confirm the virus induced inflammation in swollen muscles, we conducted gene expression profiling of pro-inflammatory cytokines at 9 dpi. As shown in Figure 2C, significant elevated levels of pro-inflammatory cytokines, such as IL-6, TNF-α, MCP-1, MCP-3 and RANTES, were observed (Figure 2C). Collectively, our data showed that CHIKV successfully invaded the mouse muscle tissue, which led to enhanced inflammation followed by myopathy.

### Microscopic observation

**Figure 3:**
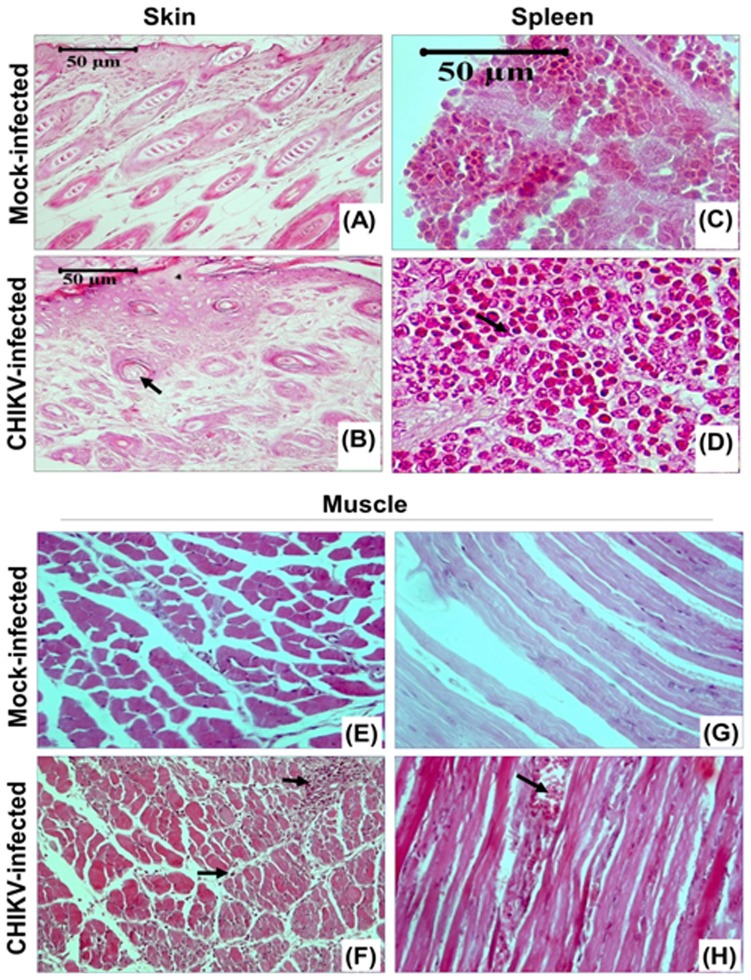
Skin, spleen and muscle pathology of CHIKV infected mice. Representative picture showing the pathology staining of the tissues (skin, spleen and hind limb muscle) from mock-infected and CHIKV-infected mice on 8th day of post infection. Characteristic histological features are indicated by arrows. Skin of CHIKV-infected mice showed hyperplasia of basal keratinocytes and hyperkeratinisation. Hair follicles showed atrophy with no dividing cells in the matrix. Spleen of CHIKV-infected mice showed considerable lymphoid proliferation and haemorrhage. Muscle sections showed degenerative changes with dark pink stained, atrophied and necrotic muscle fibres, infiltration of neutrophiles and monocytoid cells between the muscle fibres.

Histological analyses of skin, spleen and muscle tissue at 8 dpi showed differential changes that took place in response to CHIKV infection as compared to tissues collected from uninfected animals. The skin sections the of mock-infected mice showed that the matrix of hair follicle consisted of dividing cells and the growing hair shaft was surrounded with multi-layered root sheath whereas the skin of CHIKV-infected mice showed hyperplasia of the basal keratinocytes along with swelling and hyperkeratinisation. Hair follicles of the infected animals showed atrophy with no dividing cells in the matrix that was eosinophilic in nature. Contrary to the uninfected tissue, the external root sheath in infected animals was single layered (compared panel A to B in Figure 3). The spleen of the CHIKV-infected animals showed considerable lymphoid proliferation and haemorrhage (Figure 3D, arrow). The most significant pathologic changes in the new born mice were in the skeletal muscle and adjacent connective tissues. The CHIKV infected mice showed degenerative changes with dark pink staining indicating atrophied and necrotic muscle fibres. In addition, neutrophils and monocytoid cells heavily infiltrated the muscle fibres (Figure 3F and H, arrows).

### Analysis of differentially expressed proteins after CHIK infection

Whole cell lysates of muscle tissue from mock and CHIKV-infected mice were subjected to 2-DE analysis and their proteomic profiles were obtained. More than 800-900 protein spot features were matched upon inspection of 2-DE gels between mock and CHIKV infected muscle samples.

**Figure 4:**
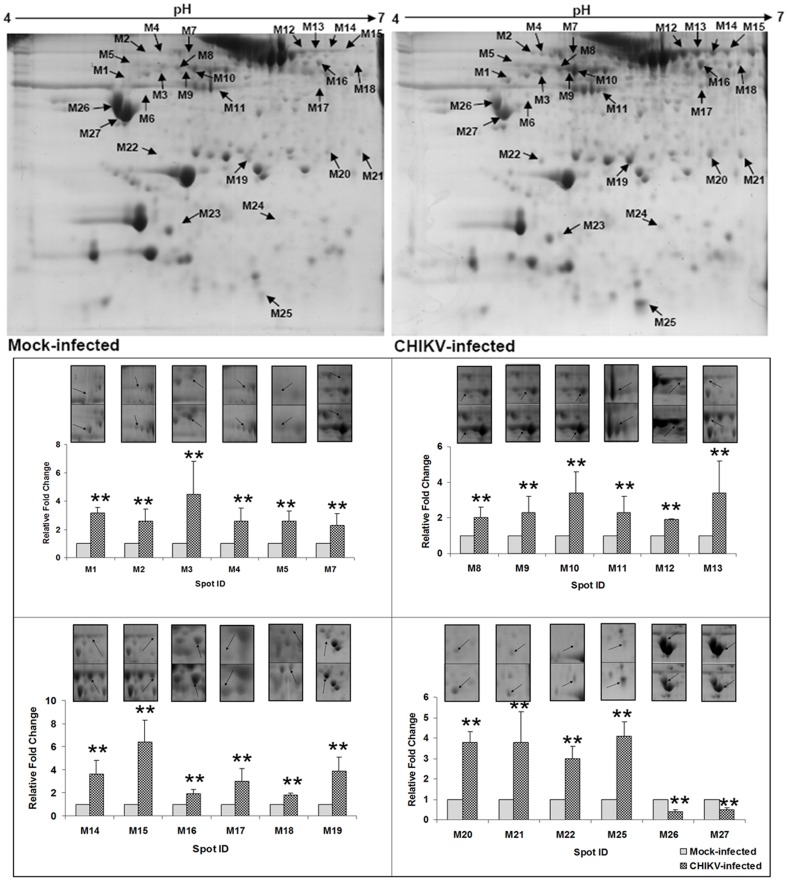
Representative 2-D gel image obtained from muscle tissue of mock-infected and CHIKV-infected mice. Equal amount of protein sample (750 μg) was first separated in a linear gradient of pH 4–7, followed by separation in SDS-PAGE (12%) and coomassie staining. A total of 27 protein spots were found to be significantly altered. Fold changes (mean values± SD) of the identified proteins are illustrated graphically (** signifies p≤0.05). Spots M6, M23 and M24 were found to be affected qualitatively and hence were not presented graphically.

**Figure 5:**
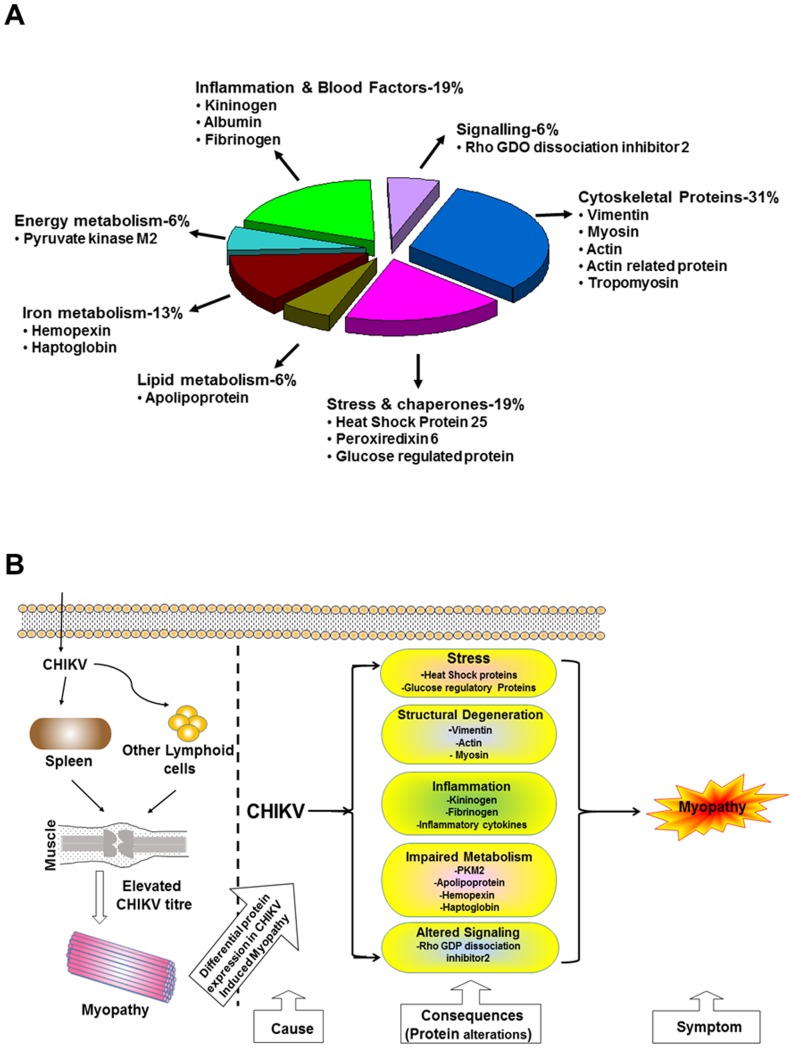
Functional classification of differentially affected proteins and their possible role in disease pathogenesis. A. Functional classification of the differentially affected proteins of muscle tissue in CHIKV infection. B. Schematic representation of the possible roles of identified proteins of different classes showing metabolic and rheumatic implications in CHIKV induced myopathy.

A total of 27 protein spots showing significant changes in mean intensity upon CHIKV infection in muscle tissue were observed (Figure 4). Twenty five spots showed up regulation of gene expressions whereas 2 spots showed down regulation at the ratio of _CHIK/mock_ ≥|1.8|, p<0.05). These protein spots were excised, digested in-gel with trypsin and identified by MALDI TOF MS analysis. The MS and MS/MS spectra are listed in File S1. The resulting peptide profiles and fragment ion spectra were searched against *Mus musculus* protein sequence database (MSDB). Table 1 provides the identity of each of the protein spots including MOWSE score, sequence coverage, number of peptides matched/searched, theoretical/observed Mr and pI obtained after tandem MS analysis. The identified proteins could be functionally classified into various groups (http://ca.expasy.org/), including those involved in inflammation, iron metabolism, cytoskeletal, energy metabolism, fatty acid metabolism, and stress chaperons. The 27 differentially expressed protein spots correspond to 15 proteins that include cytoskeleton-associated (structural) proteins (31%), stress proteins (19%), iron metabolism (13%), energy metabolism (6%), lipid metabolism (6%), inflammation and blood factors (19%) and signal transduction proteins (6%) (Figure 5A). The importance of the differentially expressed proteins in disease manifestation has been hypothesized in Figure 5B.

**Table 1:** Summary of proteins differentially expressed in mouse muscle tissue infected by Chikungunya virus.

									Spot intensity Mean± SD			
S.No	Spot ID	Protein Identified	Accession*^a^*	Mowse Score*^b^* PMF/MS-MS	% Cov*^c^*	Peptides Matched (searched)	Mr/pI theoretical	Mr/pI observed	Mock Infected	CHIKV Infected	Fold change Mean± SD	P value
1	M1	Vimentin	P20152	290/530	77	59 (132)	53.68/5.06	63/4.9	5.8±1.7	18.5±6.4	3.15±0.41	8×10^−3^
2	M2	a.Albumin b.Kininogen	P07724 O08677	132/NA 86/77	59 51	36 (119) 31 (119)	53.17/4.88	95/5.2	3.6±1.2	8.2±1.7	2.6±0.82	1×10^−2^
3	M3	Vimentin	P20152	241/241	85	61 (176)	53.5/5.06	70/5.2	5.4±2.3	26±11.4	4.5±2.3	3×10^−2^
4	M4	Kininogen	O08677	105/NS	56	28 (147)	47.8/5.74	95/5.2	6.1±1.3	14.9±1.7	2.6±0.9	9×10^−3^
5	M5	Vimentin	P20152	214/73	73	54 (173)	51.5/4.9	75/5.0	6.9±1.6	17.6±5.8	2.6±0.7	1×10^−2^
6	M6	Haptoglobin	Q3UBS3	64/117	54	21 (129)	38.8/6.08	52/5.0	0	5.4±1.4	DIV/0^e^	6×10^−3^
7	M7	Myosin	Q5SX40	120/58	48	95 (167)	60.9/5.4	92/5.4	4.9±1.9	12.2±3.0	2.3±0.8	1×10^−2^
8	M8	Vimentin	P20152	262/219	89	65 (162)	51.5/4.96	72/5.3	27.7±4.3	52±9.2	2±0.6	5×10^−3^
9	M9	Vimentin	Q5FWJ3	265/474	89	77 (181)	53.6/5.06	72/5.3	16.2±2.5	35.9±8.1	2.3±0.9	5×10^−3^
10	M10	Vimentin	P20152	217/337	83	64 (178)	53.6/5.06	72/5.4	30±1.6	101±37.4	3.4±1.2	9×10^−3^
11	M11	Actin	P60710	160/215	82	37 (125)	41.7/5.3	57/5.6	17.5±4.3	36.4±3.9	2.3±0.9	1×10^−3^
12	M12	a.Albumin b.Hemopexin	P07724 Q91X72	154/NA 98/417	71 56	54 (164) 27 (164)	50.51/7.32	95/6.3	41.5±12	81±27.05	1.9±0.04	4×10^−2^
13	M13	a.Hemopexin b.Albumin	Q91X72 P07724	152/404 114/NA	63 69	31 (126) 46 (126)	51.3/7.92	95/6.4	17.5±6.8	53±14.6	3.4±1.8	4×10^−3^
14	M14	Hemopexin	Q91X72	170/55	51	25 (108)	51.3/7.92	95/6.5	13±7.9	40±12.2	3.6±1.2	9×10^−3^
15	M15	Hemopexin	Q91X72	211/115	58	29 (111)	51.3/7.92	95/6.7	5.67±0.7	35.5±6.3	6.4±1.9	2×10^−2^
16	M16	Glucose regulated protein	P27773	203/421	69	45 (170)	56.59/5.78	72/6.4	21.4±3.6	40.1±6.8	1.9±0.4	2×10^−3^
17	M17	Pyruvate kinase isozyme M2	P52480	120/84	83	35 (159)	43.1/5.88	58/6.4	3.9±0.43	11.5±3.2	3.0±1.1	3×10^−3^
18	M18	Fibrinogen	Q3TGR2	110/124	66	42 (182)	54.7/6.68	78/6.7	12±1.7	22±1.0	1.85±0.18	3×10^−2^
19	M19	Apolipoprotein A-1	Q3V2G1	120/268	60	24 (147)	30.66/5.64	34/5.9	18.7±7.1	66.3±4.0	3.9±1.2	2×10^−5^
20	M20	Peroxiredoxin-6	O08709	176/306	88	30 (119)	24.7/5.72	36/6.5	7.2±0.9	23.6±5.9	3.8±0.5	1×10^−4^
21	M21	Heat shock protein 25	P14602	117/160	71	18 (110)	23/6.12	36/6.7	11.4±3.8	40.6±10	3.8±1.6	9×10^−3^
22	M22	Rho GDP dissociation inhibitor 2	Q61599	92/37	81	17 (97)	22.7/4.97	34/5.1	3.4±0.7	10±0.96	3.0±0.6	3×10^−5^
23	M23	Actin	P60710	NS/55	NA	NA	39/5.8	26/5.3	0	7.6±1.2	DIV/0^e^	1×10^−5^
24	M24	Actin related protein	Q6ZQK2	64/90	37	65 (180)	191.2/6.15	26/6.3	0	4.7±0.95	DIV/0^e^	5×10^−4^
25	M25	NA*^d^*	NA	NA	NA	NA	NA	NA	23.8±5.3	94.5±11	4.1±0.7	2×10^−5^
26	M26	Tropomyosin alpha chain	P58772	167/420	70	35 (129)	32.8/4.6	50/4.8	512.6±22	208±15	0.4±0.3	5×10^−3^
27	M27	Tropomyosin alpha chain	P58772	126/323	69	31 (142)	32.6/4.8	45/4.9	637±128	284.5±52	0.5±0.1	2×10^−3^

*a* = Swiss Prot accession number. *b* =  By MASCOT. *c* =  % Coverage of the identified sequence. NA*^d^*  =  not available. DIV/0^e^ =  Divided by zero. Sopt ID represents the number on 2-DE gels. NS = Non significant.

**Figure 6:**
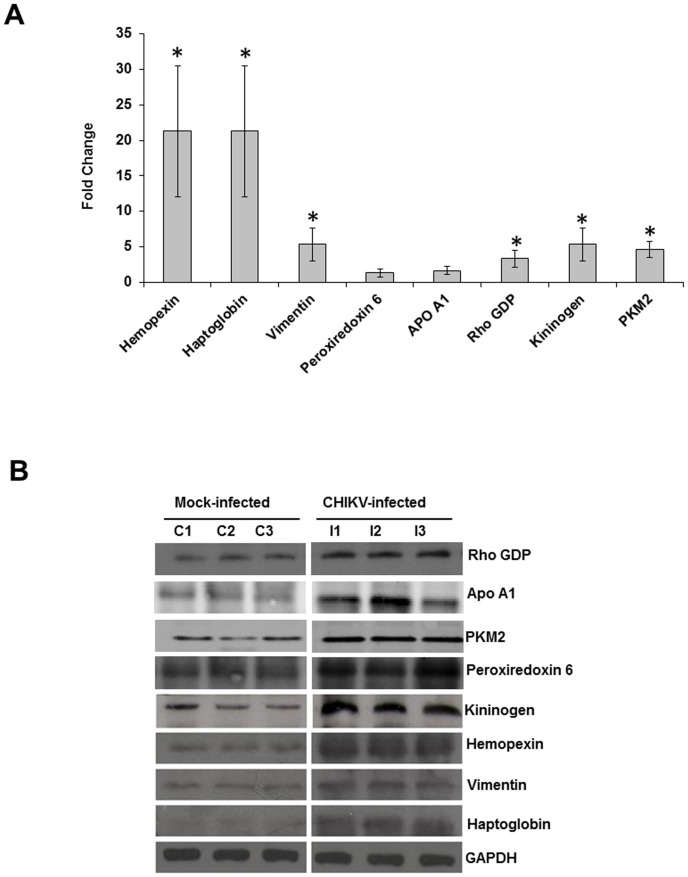
Validation of proteomic results using Q-PCR and immunoblotting. A. Transcript alteration of the differentially expressed proteins in muscle tissue upon CHIKV infection. Total RNA of muscle tissue (infected/uninfected) was analysed by real time RT-PCR. House-keeping GAPDH gene was used for normalization purpose. RNA expression changes of vimentin, hemopexin, haptoglobin, Rho GDP, PKM2 and kininogen were in concordance with protein expression changes and were determined to be statistically significant (p≤0.05). *Genes were considered to be significantly up-regulated if the change in their relative expression levels was ≥2 fold. No significant difference in the RNA expression of ApoA1 and peroxiredoxin 6 was found. B. Immunoblot of representative proteins showing increased expressions in muscle tissue upon CHIKV infection.

The differentially affected proteins were validated by Q-PCR and western blotting. The transcriptional alteration of eight selected genes was analysed at the mRNA transcript level using the housekeeping GAPDH gene for normalization purpose. The candidate proteins were selected as being representative of structural (vimentin), stress (peroxiredoxin 6), lipid metabolism (ApoA1), signalling (Rho GDP), iron metabolism (hemopexin and haptoglobin), inflammation (kininogen), and energy metabolic (pyruvate kinase M2) pathways. Out of eight selected genes, six genes (hemopexin, haptoglobin, vimentin, kininogen, PKM2 and Rho GDP) exhibited the same trend of up-regulation as observed in 2-DE gels of the corresponding proteins (Figure 6A). On the contrary, the transcript levels of ApoA1 and peroxiredoxin 6 remained unchanged. To further confirm the alterations of protein expression during CHIKV infection, all the selected proteins (i.e., hemopexin, haptoglobin, vimentin, ApoA1, peroxiredoxin 6, Rho GDP, PKM2, and kininogen) were analysed by western blotting. Equal amount of mock and CHIKV infected muscle-tissue protein were examined by western blot analysis with specific antibodies. The data obtained agreed with the expression changes by the 2-DE analysis as all the proteins were up-regulated (Figure 6B). The discrepancy at the transcriptional and protein levels of ApoA1 and peroxiredoxin 6 could be due to the altered regulation of transcription and translation of these genes.

Among the differentially expressed proteins, 31% of proteins were assigned to the structural protein category. Vimentin, actin, actin related protein, myosin and tropomyosin were among the differentially expressed cytoskeleton-associated proteins. Vimentin, an intermediate filament protein normally expressed in cells of mesenchymal origin, plays important roles in many biological reactions [32], including but not necessarily limited to cell growth [33], cellular motility and invasiveness of some cancers [34], and as a signal transducer, relaying information from extracellular matrix to the nucleus [35]. Interestingly, vimentin is also expressed on the cell surface of activated macrophages and apoptotic neutrophils. Hence, one possible reason for the increased expression of vimentin could be due to the increased neutrophil infiltration in CHIKV infected muscles as observed in histological analysis.

Many of other differentially expressed structural proteins may play important roles in the development of arthritis and myositis in CHIKV-infected individuals. For example, there are several reports documenting that the over-expression of cytoskeletal proteins such as actin, myosin and vimentin lead to development of auto-antibodies in rheumatoid arthritis and other inflammatory diseases [36], [37]. Likewise, overexpression of proteins, such as haptoglobin and hemopexin, has been reported as a result of inflammation and injury [38], [39] and in myopathic disorders [40]. We also observed a significant level of increase of kininogen and fibrinogen expression in the muscle of CHIKV infected mice. Kininogen and fibrinogen have been shown to be associated with inflammation and arthritis [41], [42] and kininogen is also one of the known regulators of stress/energy metabolic proteins [43]. Among the differentially affected proteins, 19% of the proteins were stress-related proteins that included heat shock protein 25 (HSP25), peroxiredoxin 6 and glucose regulated protein (Grp). The upregulation of heat shock proteins in skeletal muscles have been shown to provide some protection against a variety of stressors, such as disuse atrophy [44], [45], oxidative stress [45], [46], increased calcium concentrations [47] and muscle damage [48], [49], [50]. Furthermore several observations suggest that the small HSPs, such as HSP25 play crucial roles in the normal function and integrity of skeletal muscles as well in arthritis [51], [52]. Glucose regulated protein (Grp), an ER molecular chaperone that assists in protein folding and assembly has been reported to be differentially expressed in rheumatoid synovitis [53]. Similarly, we observed a significant increase in expression of peroxiredoxin-6, another antioxidant enzyme that functions to catalyze peroxides and protects cells against reactive oxygen species. Apart from the antioxidant properties, peroxiredoxins have also been shown to be important in various cellular processes such as regulation of apoptosis [54] and antiviral activities [55]. The possible involvement of stress-related proteins in CHIKV infection has been reported in our previous studies [26], [56] and by other researchers [57].

The metabolic proteins that were overexpressed in CHIKV-infected muscles were PKM2, an isoenzyme of the glycolytic enzyme pyruvate kinase, and ApoA1, a protein associated with lipid metabolism. PKM2 protein expression has been reported to be increased in rheumatic diseases and has also been shown to support the replication of RNA viruses [58], [59]. Hence, it is possible that the elevated level of PKM2 might be responsible for supporting CHIKV replication in infected muscle. Interestingly, expressions of ApoA1 and Rho GDP dissociation inhibitor 2 have also been shown to be elevated in synovial fluid of patients with rheumatoid arthritis and osteoarthritis [60], [61]. Collectively, elevated expressions of many of the proteins observed in our study might be directly involved in CHIKV infection and disease pathogenesis and directly or indirectly correlated with muscle-related disorders such as arthritis and myositis.

Further studies are needed for in depth characterization of affected proteins and their interactions with CHIKV that will likely open new avenues for the design of antiviral therapies against CHIKV and related disorders caused by alphaviruses. Also, the validation of differentially expressed proteins in serum samples of CHIKV infected patients may reveal relevant clinical biomarkers and thereby allow for early detection of the disease that can improve outcomes by providing better medical treatment and/or disease management strategies.

## Supporting Information

Figure S1 **Average walking stride length.** This parameter was measured manually by allowing the control (mock-infected) and CHIKV-infected mice to walk on a sheet of paper with their foot-steps being marked. The total distance covered was then divided by the number of steps taken. The data was expressed as mean±SE of five animals per group and the experiment was repeated twice (p≤0.05).
(TIF)

Figure S2 **Muscle sections showing the localisation of CHIKV antigen by immunofluorescence at different days of infection.**
(TIF)

File S1 **The MS and MS/MS spectra of the differentially affected proteins.**
(PDF)

Movie S1 **Walking behavior of CHIKV infected mice showing lack of coordination and hind limb paralysis.**
(WMV)

Movie S2 **Surface righting reflex of control (mock-infected) mice.**
(MPG)

Movie S3 **Surface righting reflex of CHIKV-infected mice.** The infected mice demonstrated difficulty in turning to the upright position confirming weakness of the hind limbs.
(MPG)
