# Supplementary figures and images for: Characterization of Chikungunya Virus Induced Host Response in a Mouse Model of Viral Myositis
Source: PLoS One. 2014 Mar 25;9(3):e92813. doi: 10.1371/journal.pone.0092813 (PMC3965460; doi:10.1371/journal.pone.0092813)

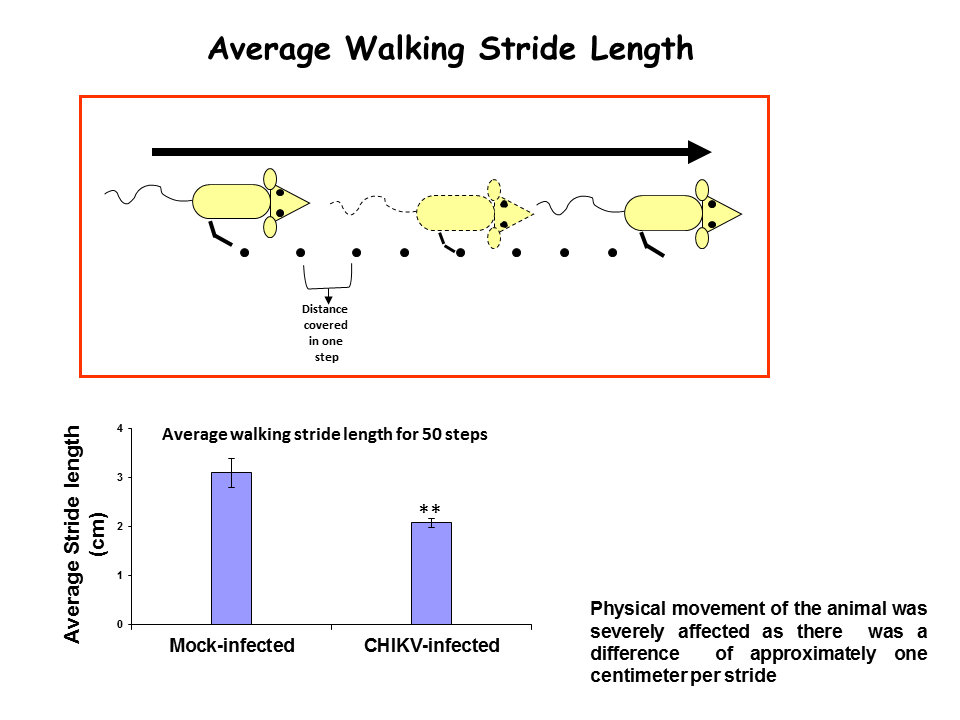

Supplement: Figure S1 — Average walking stride length. This parameter was measured manually by allowing the control (mock-infected) and CHIKV-infected mice to walk on a sheet of paper with their foot-steps being marked. The total distance covered was then divided by the number of steps taken. The data was expressed as mean±SE of five animals per group and the experiment was repeated twice (p≤0.05). (TIF) [file pone.0092813.s001.tif]

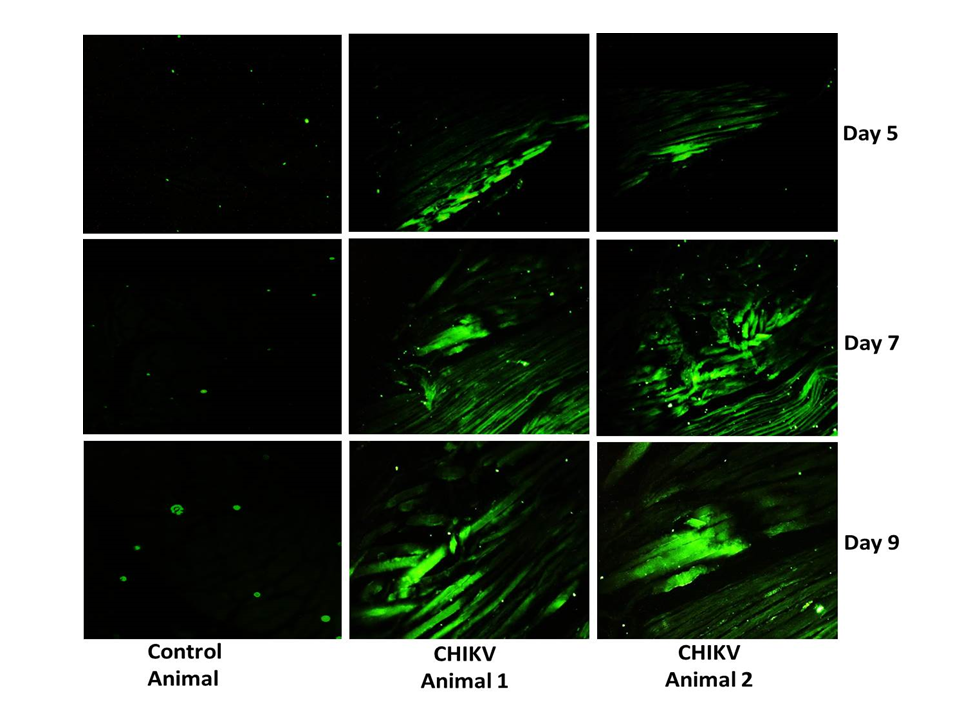

Supplement: Figure S2 — Muscle sections showing the localisation of CHIKV antigen by immunofluorescence at different days of infection. (TIF) [file pone.0092813.s002.tif]
