# Supplementary material for: Characterization of Chikungunya Virus Induced Host Response in a Mouse Model of Viral Myositis
Source: PLoS One. 2014 Mar 25;9(3):e92813. doi: 10.1371/journal.pone.0092813 (PMC3965460; doi:10.1371/journal.pone.0092813)
Supplement: File S1 — The MS and MS/MS spectra of the differentially affected proteins. (PDF) [file pone.0092813.s003.pdf]

Sample M1

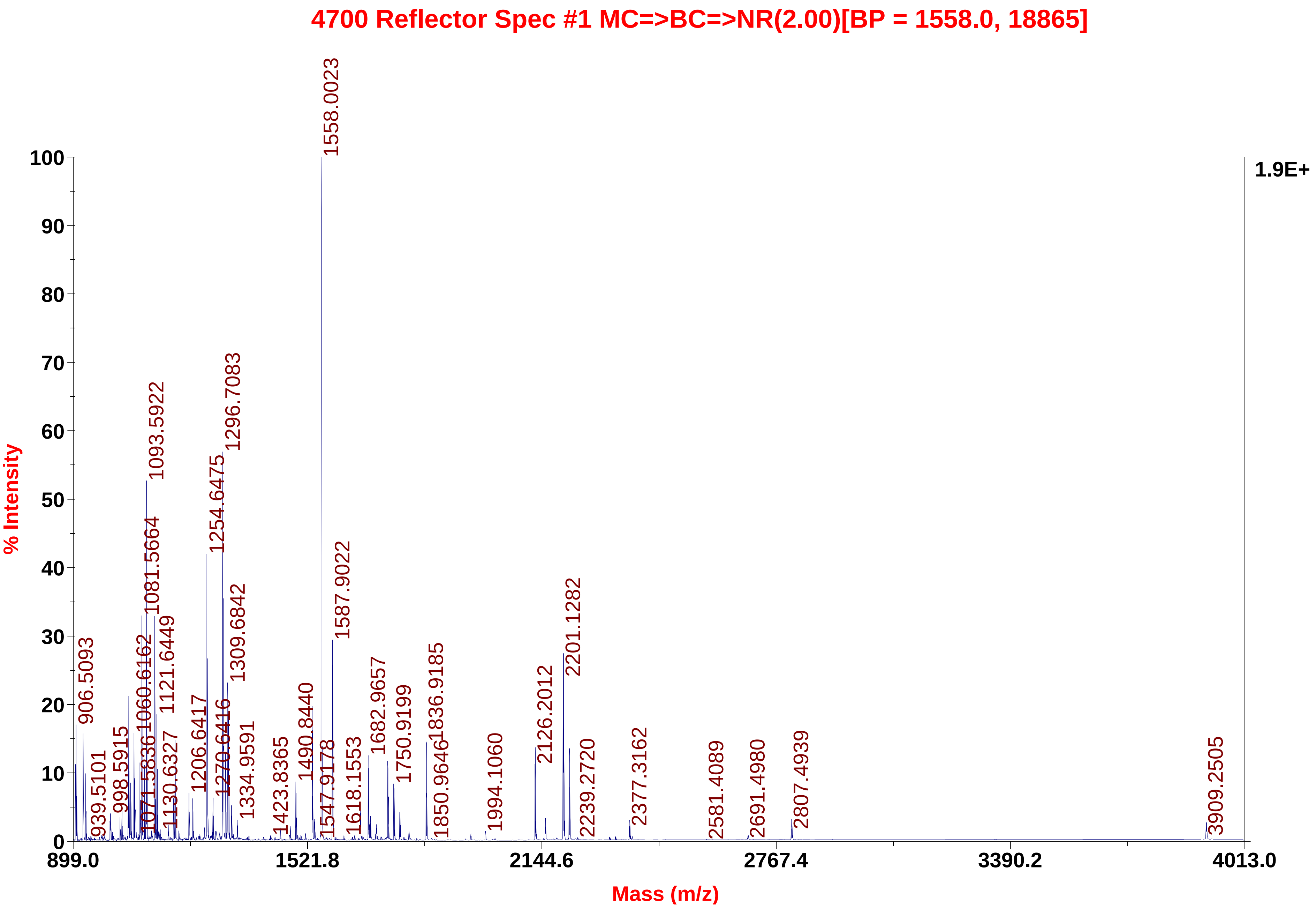

4700 Reflector Spec #1 MC=>BC=>NR(2.00)[BP = 1558.0, 18865]
% Intensity
Mass (m/z)
1.9E+
100
90
80
70
60
50
40
30
20
10
0
899.0
1521.8
2144.6
2767.4
3390.2
4013.0
906.5093
939.5101
998.5915
1060.6162
1071.5836
1081.5664
1093.5922
1121.6449
1130.6327
1206.6417
1254.6475
1270.6416
1296.7083
1309.6842
1334.9591
1423.8365
1490.8440
1547.9178
1558.0023
1587.9022
1618.1553
1682.9657
1750.9199
1836.9185
1850.9646
1994.1060
2126.2012
2201.1282
2239.2720
2377.3162
2581.4089
2691.4980
2807.4939
3909.2505

Mascot Score Histogram

Protein score is -10*Log(P), where P is the probability that the observed match is a random event.
Protein scores greater than 62 are significant (p<0.05).

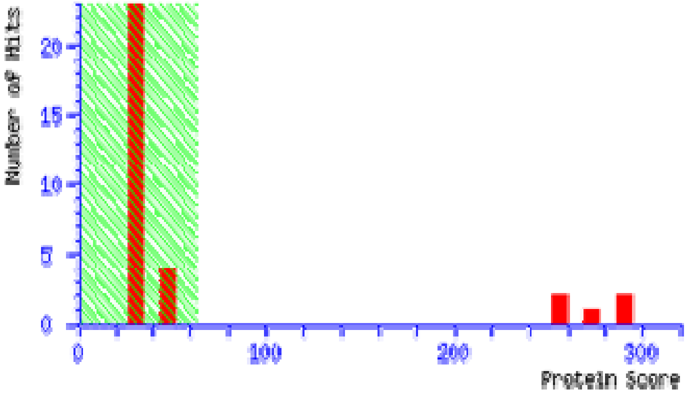

Concise Protein Summary Report

Format As | Concise Protein Summary | Help

Significance threshold p< 0.05 Max. number of hits AUTO

Re-Search All | Search Unmatched

1. CAA39807 **Mass**: 53689 **Score**: 290 **Expect**: 9.2e-25 **Matches**: 59
   MMVMTM NID: - Mus musculus

---

2. VIME_MOUSE **Mass**: 53524 **Score**: 290 **Expect**: 9.2e-25 **Matches**: 59
   Vimentin.- Mus musculus (Mouse).
   Q5FWJ3_MOUSE **Mass**: 53655 **Score**: 289 **Expect**: 1.2e-24 **Matches**: 59
   Vimentin (NOD-derived CD11c +ve dendritic cells cDNA, RIKEN full- length enriched library, clone:F630103E23 product:vimentin,
   CAA69019 **Mass**: 51533 **Score**: 282 **Expect**: 5.8e-24 **Matches**: 58
   MMVIMENT NID: - Mus musculus
   Q3TFD9_MOUSE **Mass**: 53656 **Score**: 276 **Expect**: 2.3e-23 **Matches**: 57
   17 days embryo heart cDNA, RIKEN full-length enriched library, clone:I920087O13 product:vimentin, full insert sequence.- Mus

Protein View

Match to: **CAA39807** Score: **290** Expect: **9.2e-25**
MMVMTM NID: - Mus musculus
Found in search of pmf_A2_129990848800.txt

Nominal mass (M_r): **53689**; Calculated pI value: **5.06**
NCBI BLAST search of CAA39807 against nr
Unformatted sequence string for pasting into other applications

Taxonomy: Mus musculus

Variable modifications: Carbamidomethyl (C),Deamidated (NQ),Oxidation (M)
Cleavage by Trypsin: cuts C-term side of KR unless next residue is P
Number of mass values searched: **132**
Number of mass values matched: **59**
Sequence Coverage: **77%**

Matched peptides shown in **Bold Red**

```
  1 MSTRSVSSSS YRRMFGGSGT SSRPSSNRSY VTTSTRTYSL GSALRPSTSR
 51 SLYSSSPGGA YVTRSSAVRL RSSVPGVRLL QDSVDFSLAD AINTEFKNTR
101 TNEKVELQEL NDRFANYIDK VRFLEQQNKI LLAELEQLKG QGKSRLGDLY
151 EEEMRELRRQ VDQFTNDKAR VEVERDNLAE DIMRLREKLQ EEMLQREEAE
201 STLQSFRQDV DNASLARLDL ERKVESLQEE IAFLKKLHDE EIQELQAQIQ
251 EQHVQIDVDV SKPDLTAALR DVRQQYESVA AKNLQEAEEW YKSKFADLSE
301 AANRNNDALR QAKQESNEYR RQVQSLTCEV DALKGTNESL ERQMREMEEN
351 FALEAANYQD TIGRLQDEIQ NMKEEMARHL REYQDLLNVK MALDIEIATY
401 RKLLEGEESR ISLPLPTFSS LNLRETNLES LPLVDTHSKR TLLIKTVETR
451 DGQVINETSQ HHDDLE
```

Show predicted peptides also

Sort Peptides By ◉ Residue Number ○ Increasing Mass ○ Decreasing Mass

Start - End	Observed	Mr(expt)	Mr(calc)	ppm	Miss	Sequence
2 - 12	1216.7050	1215.6977	1215.5844	93	1	M.STRSVSSSSYR.R
5 - 13	1028.5620	1027.5547	1027.5047	49	1	R.SVSSSSYRR.M
79 - 97	2126.1985	2125.1912	2125.0579	63	0	R.LLQDSVDFSLADAINTEFK.N
101 - 113	1587.9003	1586.8930	1586.7900	65	1	R.TNEKVELQELNDR.F
105 - 113	1115.6379	1114.6306	1114.5618	62	0	K.VELQELNDR.F
114 - 122	1125.6663	1124.6590	1124.5978	54	1	R.FANYIDKVR.F
123 - 129	906.5092	905.5019	905.4607	46	0	R.FLEQQNK.I
123 - 129	908.4391	907.4318	907.4287	3	0	R.FLEQQNK.I 2 Deamidated (NQ)
130 - 139	1169.7773	1168.7700	1168.7067	54	0	K.ILLAELEQLK.G
130 - 143	1540.0043	1538.9970	1538.9032	61	1	K.ILLAELEQLKGQGK.S
146 - 155	1254.6475	1253.6402	1253.5598	64	0	R.LGDLYEEEMR.E
146 - 155	1270.6410	1269.6337	1269.5547	62	0	R.LGDLYEEEMR.E Oxidation (M)
146 - 158	1668.8984	1667.8911	1667.7824	65	1	R.LGDLYEEEMRELR.R Oxidation (M)
160 - 170	1323.7225	1322.7152	1322.6102	79	1	R.QVDQFTNDKAR.V 2 Deamidated (NQ)
171 - 184	1688.9368	1687.9295	1687.8199	65	1	R.VEVERDNLAEDIMR.L
171 - 184	1704.9309	1703.9236	1703.8148	64	1	R.VEVERDNLAEDIMR.L Oxidation (M)

M1

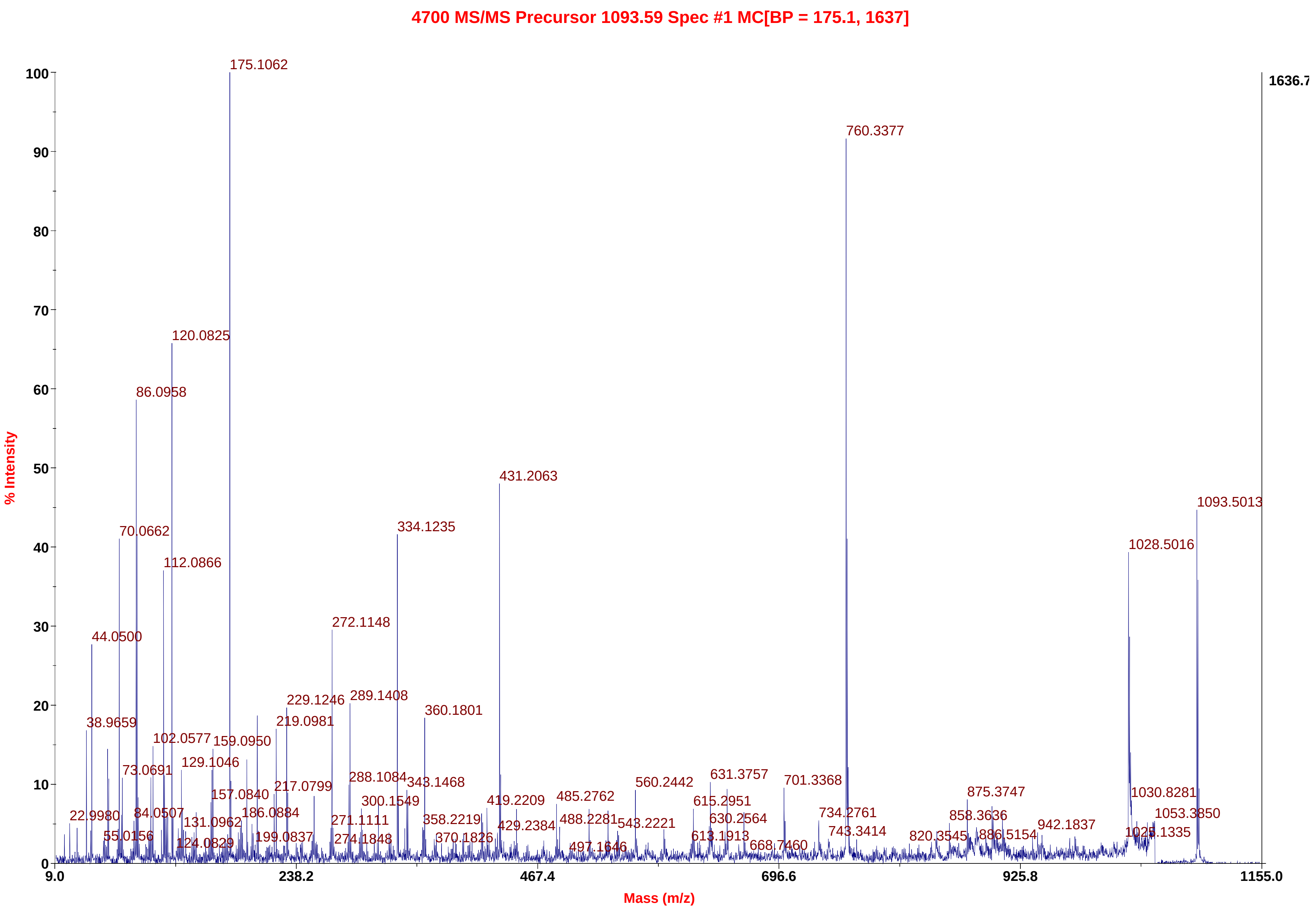
4700 MS/MS Precursor 1093.59 Spec #1 MC[BP = 175.1, 1637]
% Intensity
Mass (m/z)
175.1062
760.3377
120.0825
86.0958
431.2063
1093.5013
1028.5016
70.0662
334.1235
112.0866
44.0500
272.1148
229.1246
289.1408
219.0981
360.1801
38.9659
102.0577
159.0950
129.1046
73.0691
631.3757
701.3368
560.2442
288.1084
343.1468
217.0799
157.0840
875.3747
1030.8281
485.2762
419.2209
300.1549
615.2951
84.0507
186.0884
22.9980
734.2761
858.3636
1053.3850
131.0962
271.1111
358.2219
488.2281
630.2564
543.2221
942.1837
429.2384
743.3414
1028.1335
55.0156
199.0837
370.1826
613.1913
820.3545
886.5154
274.1848
124.0829
497.1646
668.7460
1636.7

M1

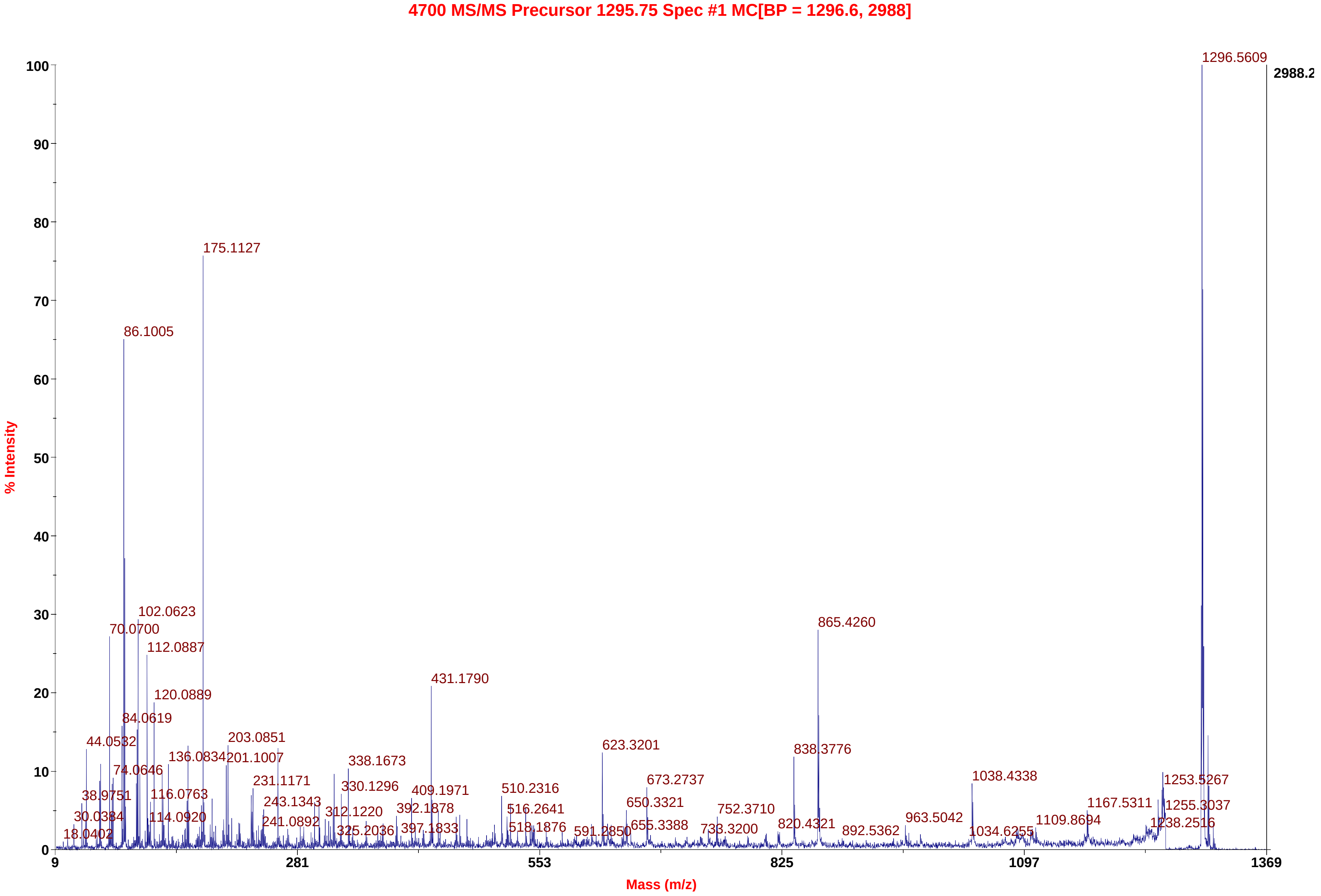
4700 MS/MS Precursor 1295.75 Spec #1 MC[BP = 1296.6, 2988]
% Intensity
Mass (m/z)
1296.5609
2988.2
175.1127
86.1005
102.0623
70.0700
112.0887
120.0889
84.0619
44.0532
74.0646
136.0834
201.1007
203.0851
231.1171
243.1343
241.0892
38.9751
30.0384
18.0402
116.0763
114.0920
338.1673
330.1296
312.1220
325.2036
431.1790
409.1971
392.1878
397.1833
510.2316
516.2641
518.1876
591.2850
623.3201
650.3321
655.3388
673.2737
752.3710
733.3200
820.4321
838.3776
865.4260
892.5362
963.5042
1038.4338
1034.6255
1109.8694
1167.5311
1238.2516
1253.5267
1255.3037
9
281
553
825
1097
1369
0
10
20
30
40
50
60
70
80
90
100

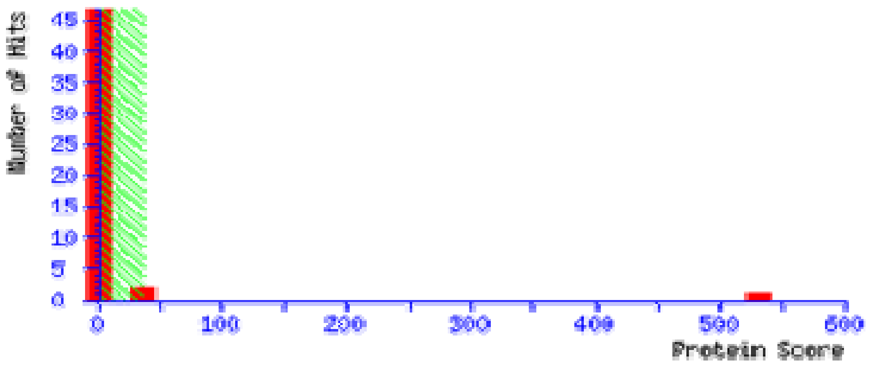

Peptide Summary Report

Format As | Peptide Summary | Help

Significance threshold p< 0.05 | Max. number of hits AUTO

Standard scoring | MudPIT scoring | Ions score or expect cut-off 0 | Show sub-sets 0

Show pop-ups | Suppress pop-ups | Sort unassigned Decreasing Score | Require bold red

Select All | Select None | Search Selected | Error tolerant

1. VIME_MOUSE Mass: 53524 Score: 530 Matches: 20(14) Sequences: 19(14) emPAI: 4.20
Vimentin.- Mus musculus (Mouse).
Check to include this hit in error tolerant search

Query	Observed	Mr(expt)	Mr(calc)	ppm	Miss	Score	Expect	Rank	Unique	Peptide
1	906.5092	905.5019	905.4607	45.6	0	36	0.072	1		R.FLEQQNK.I
2	925.4561	924.4488	924.3937	59.7	0	33	0.12	1	U	K.QESNEYR.R
3	1046.6010	1045.5937	1045.5226	68.0	0	18	4.4	1	U	K.LQEEMLQR.E
4	1081.5643	1080.5570	1080.4948	57.6	1	16	6	1	U	K.QESNEYRR.Q
5	1093.5905	1092.5832	1092.5200	57.9	0	58	0.00051	1	U	K.FADLSEAANR.N
6	1115.6379	1114.6306	1114.5618	61.7	0	38	0.043	1	U	K.VELQELNDR.F
7	1169.7773	1168.7700	1168.7067	54.2	0	63	1.9e-05	1	U	K.ILLAELEQLK.G
8	1254.6475	1253.6402	1253.5598	64.2	0	52	0.002	1	U	R.LGDLYEEEMR.E
9	1295.7474	1294.7401	1294.6591	62.6	0	29	0.26	1	U	K.MALDIEIATYR.K

Protein View

Match to: **VIME_MOUSE** Score: 530
Vimentin.- Mus musculus (Mouse).
Found in search of ppw_A2_129990854000.txt

Nominal mass (M_r): **53524**; Calculated pI value: **5.06**
NCBI BLAST search of VIME_MOUSE against nr
Unformatted sequence string for pasting into other applications

Taxonomy: Mus musculus

Variable modifications: Carbamidomethyl (C),Deamidated (NQ),Oxidation (M)
Cleavage by Trypsin: cuts C-term side of KR unless next residue is P
Sequence Coverage: **45%**

Matched peptides shown in **Bold Red**

1 STRSVSSSSY RRMFGGSGTS SRPSSNRSYV TTSTRTYSLG SALRPSTSRS
51 LYSSSPGGAY VTRSSAVRLR SSVPGVR**LLQ** **DSVDFSLADA** **INTEFK**NTR**T**
101 **NEKVELQELN** **DR**FANYIDKV R**FLEQQNKIL** **LAELEQLK**GQ GKSR**LGDLYE**
151 **EEMR**ELRRQV DQLTNDKARV EVERDNLAED IMRLREK**LQE** **EMLQR**EEAES
201 TLQSFRQDVD NASLARLDLE R**KVESLQEEI** **AFLK**KLHDEE IQELQAQIQE
251 QHVQIDVDVS KPDLTAALRD VRQQYESVAA K**NLQEAEEWY** **K**SK**FADLSEA**
301 **ANR**NNDALRQ AK**QESNEYRR** **QVQSLTCEVD** **ALK**GTNESLE RQMR**EMEENF**
351 **ALEAANYQDT** **IGRLQDEIQN** **MKEEMAR**HLR EYQDLLNVK**M** **ALDIEIATYR**
401 KLLEGEESR**I** **SLPLPTFSSL** **NLRETNLESL** **PLVDTHSK**RT LLIKTVETR**D**
451 **GQVINETSQH** **HDDLE**

Show predicted peptides also

Sort Peptides By ◉ Residue Number ○ Increasing Mass ○ Decreasing Mass

Start - End	Observed	Mr(expt)	Mr(calc)	ppm	Miss	Sequence	
78 - 96	2126.1985	2125.1912	2125.0579	63	0	R.LLQDSVDFSLADAINTEFK.N	(Ions score 109)
100 - 112	1587.9003	1586.8930	1586.7900	65	1	R.TNEKVELQELNDR.F	(Ions score 58)
104 - 112	1115.6379	1114.6306	1114.5618	62	0	K.VELQELNDR.F	(Ions score 38)
122 - 128	906.5092	905.5019	905.4607	46	0	R.FLEQQNK.I	(Ions score 36)
129 - 138	1169.7773	1168.7700	1168.7067	54	0	K.ILLAELEQLK.G	(Ions score 63)
145 - 154	1254.6475	1253.6402	1253.5598	64	0	R.LGDLYEEEMR.E	(Ions score 52)
188 - 195	1046.6010	1045.5937	1045.5226	68	0	K.LQEEMLQR.E	(Ions score 18)
222 - 234	1533.9469	1532.9396	1532.8450	62	1	R.KVESLQEEIAFLK.K	(Ions score 78)
282 - 291	1309.6841	1308.6768	1308.5986	60	0	K.NLQEAEEWYK.S	(Ions score 46)

Sample M2

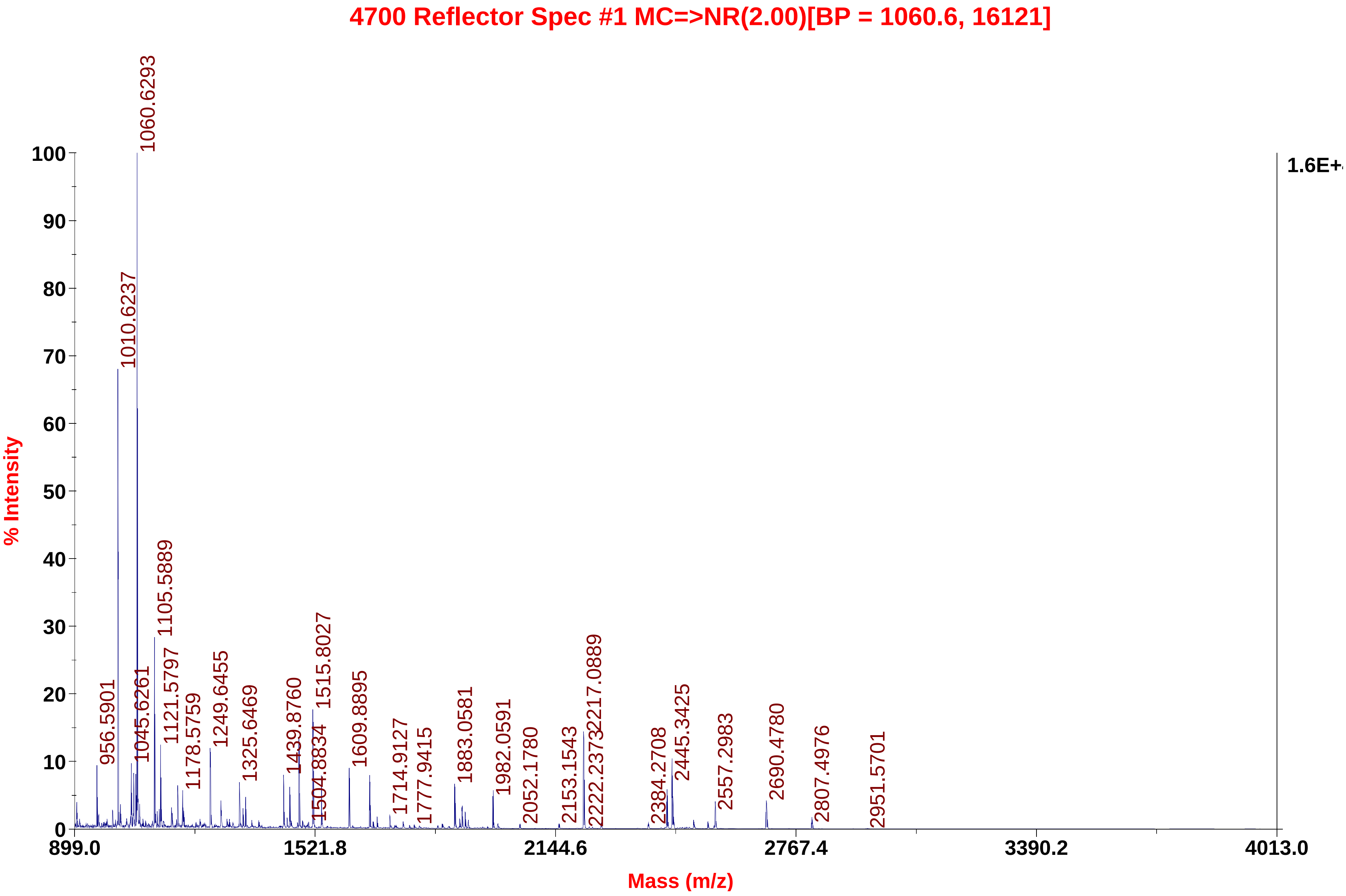

4700 Reflector Spec #1 MC=>NR(2.00)[BP = 1060.6, 16121]
% Intensity
Mass (m/z)
1.6E+4
1060.6293
1010.6237
1105.5889
956.5901
1045.6261
1121.5797
1178.5759
1249.6455
1325.6469
1439.8760
1504.8834
1515.8027
1609.8895
1714.9127
1777.9415
1883.0581
1982.0591
2052.1780
2153.1543
2217.0889
2222.2373
2384.2708
2445.3425
2557.2983
2690.4780
2807.4976
2951.5701
899.0
1521.8
2144.6
2767.4
3390.2
4013.0

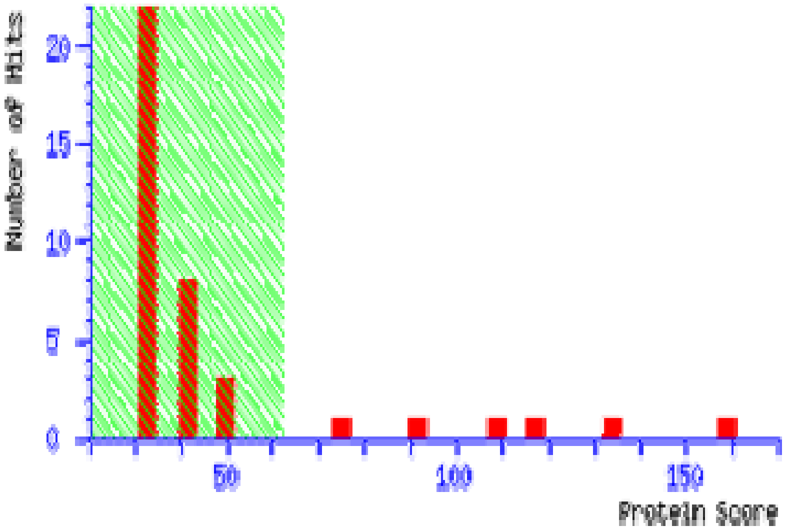

Concise Protein Summary Report

Format As | Concise Protein Summary | Help

Significance threshold p< 0.05 Max. number of hits AUTO

Re-Search All | Search Unmatched

1. **Mixture 1** **Total score**: 159 **Expect**: 1.2e-11 **Matches**: 60

Components (only one family member shown for each component):

Q8C7C7_MOUSE **Mass**: 64961 **Score**: 132 **Expect**: 5.8e-09 **Matches**: 36

2 days neonate thymus thymic cells cDNA, RIKEN full-length enriched library, clone:C920028B14 product:albumin 1,

Q6S9I1_MOUSE **Mass**: 53172 **Score**: 86 **Expect**: 0.00021 **Matches**: 31

HMW kininogen-I variant (High molecular weight kininogen I isoform DeltaD5).- Mus musculus (Mouse).

Protein View

Match to: **Q8C7C7_MOUSE** Score: **132** Expect: **5.8e-09**
2 days neonate thymus thymic cells cDNA, RIKEN full-length enriched library, clone
Found in search of pmf_B2_129990848801.txt

Nominal mass (M_r): **64961**; Calculated pI value: **5.49**
NCBI BLAST search of Q8C7C7_MOUSE against nr
Unformatted sequence string for pasting into other applications

Taxonomy: Mus musculus
Links to retrieve other entries containing this sequence from NCBI Entrez:
(no taxonomy information for this entry)

Variable modifications: Carbamidomethyl (C),Deamidated (NQ),Oxidation (M)
Cleavage by Trypsin: cuts C-term side of KR unless next residue is P
Number of mass values searched: **119**
Number of mass values matched: **36**
Sequence Coverage: **59%**

Matched peptides shown in **Bold Red**

```
  1 NRYNDLGEQH FKGLVLIAFS QYLQKCSYDE HAKLVQEVTD FAKTCVADES
 51 AANCDKSLHT LFGDKLCAIP NLRENYGELA DCCTKQEPER NECFLQHKDD
101 NPSLPPFERP EAEAMCTSFK ENPTTFMGHY LHEVARRHPY FYAPELLYYA
151 EQYNEILTQC CAEADKESCL TPKLDGVKEK ALVSSVRQRM KCSSMQKFGE
201 RAFKAWAVAR LSQTFPNADF AEITKLATDL TKVNKECCHG DLLECADDRA
251 ELAKYMCENQ ATISSKLQTC CDKPLLKKAH CLSEVEHDTM PADLPAIAAD
301 FVEDQEVCKN YAEAKDVFLG TFLYEYSRRH PDYSVSLLLR LAKKYEATLE
351 KCCAEANPPA CYGTVLAEFQ PLVEEPKNLV KTNCDLYEKL GEYGFQNAIL
401 VRYTQKAPQV STPTLVEAAR NLGRVGTKCC TLPEDQRLPC VEDYLSAILN
451 RVCLLHEKTP VSEHVTKCCS GSLVERRPCF SALTVDETYV PKEFKAETFT
501 FHSDICTLPE KEKQIKKQTA LAELVKHKPK ATAEQLKTVM DDFAQFLDTC
551 CKAADKDTCF STEGPNLVTR CKDALA
```

Show predicted peptides also

Sort Peptides By ◉ Residue Number ○ Increasing Mass ○ Decreasing Mass

Start - End	Observed	Mr(expt)	Mr(calc)	ppm	Miss	Sequence	
13 - 25	1479.8933	1478.8860	1478.8497	25	0	K.GLVLIAFSQYLQK.[illegible]	
13 - 33	2472.3254	2471.3181	2471.2042	46	1	K.GLVLIAFSQYLQKC[illegible]	
34 - 43	1149.6647	1148.6574	1148.6077	43	0	K.LVQEVTDFAK.T	
57 - 65	1017.5847	1016.5774	1016.5291	48	0	K.SLHTLFGDK.L	
66 - 73	956.5888	955.5815	955.5273	57	0	K.LCAIPNLR.E	Ca[illegible]
74 - 85	1459.6769	1458.6696	1458.5755	65	0	R.ENYGELADCCTK.Q[illegible]	

Protein View

Match to: **Q6S9I1_MOUSE** Score: **86** Expect: **0.00021**
HMW kininogen-I variant (High molecular weight kininogen I isoform DeltaD5).- Mus muscul
Found in search of pmf_B2_129990848801.txt

Nominal mass (M_r): **53172**; Calculated pI value: **4.88**
NCBI BLAST search of Q6S9I1_MOUSE against nr
Unformatted sequence string for pasting into other applications

Taxonomy: Mus musculus
Links to retrieve other entries containing this sequence from NCBI Entrez:
(no taxonomy information for this entry)
(no taxonomy information for this entry)

Variable modifications: Carbamidomethyl (C),Deamidated (NQ),Oxidation (M)
Cleavage by Trypsin: cuts C-term side of KR unless next residue is P
Number of mass values searched: **119**
Number of mass values matched: **31**
Sequence Coverage: **51%**

Matched peptides shown in **Bold Red**

```
  1 MKLITTLLLC SGLLLTLTQG EEAQEIDCND EAVFQAVDFS LKQFNPGVKS
 51 GNQYMLHRVI EGTKTDGSPT FYSFKYLIKE GNCSAQSGLA WQDCDFKDAE
101 EAATGECTAT VGKRENEFFI VTQTCKIAPS KAPILKAYFP CIGCVHAIST
151 DSPDLEPVLK HSIEHFNNNT DHSHLFTLRK VKSAHRQVVA GLNFDITYTI
201 VQTNCSKERF PSLHGDCVAL PNGDDGECRG NLFMDINNKI ANFSQSCTLY
251 SGDDLVEALP KPCPGCPRDI PVDSPELKEV LGHSIAQLNA ENDHPFYYKI
301 DTVKKATSQV VAGTKYVIEF IARETKCSKE SNTELAEDCE IKHLGQSLDC
351 NANVYMRPWE NKVVPTVKCQ ALDMTEMARR PPGFSPFRSV TVQETKEGRT
401 DSDFIEDVVA TTPPYDTGAH DDLIPDIHVQ PDSLSFKLIS DFPEATSPKC
451 PGRPWKPASW EDPNTETTEF SDFDLLDALS
```

Show predicted peptides also

Sort Peptides By ◉ Residue Number ○ Increasing Mass ○ Decreasing Mass

Start – End	Observed	Mr(expt)	Mr(calc)	ppm	Miss	Sequence	
43 – 58	1895.0566	1894.0493	1893.8679	96	1	K.QFNPGVKSGNQYMLHR.V	
43 – 58	1896.0194	1895.0121	1894.8519	85	1	K.QFNPGVKSGNQYMLHR.V	
50 – 58	1105.5889	1104.5816	1104.5134	62	0	K.SGNQYMLHR.V	
50 – 58	1121.5780	1120.5707	1120.5084	56	0	K.SGNQYMLHR.V	Oxidati
50 – 64	1749.7764	1748.7691	1748.8515	-47	1	K.SGNQYMLHRVIEGTK.T	De
65 – 75	1249.6459	1248.6386	1248.5663	58	0	K.TDGSPTFYSFK.Y	
98 – 114	1765.9055	1764.8982	1764.7948	59	1	K.DAEEAATGECTATVGKR.E	
114 – 126	1671.9216	1670.9143	1670.8086	63	1	K.RENEFFIVTQTCK.I	Carl
115 – 126	1459.6769	1458.6696	1458.6701	-0	0	R.ENEFFIVTQTCK.I	Deam
115 – 126	1515.8025	1514.7952	1514.7075	58	0	R.ENEFFIVTQTCK.I	Carb

M2

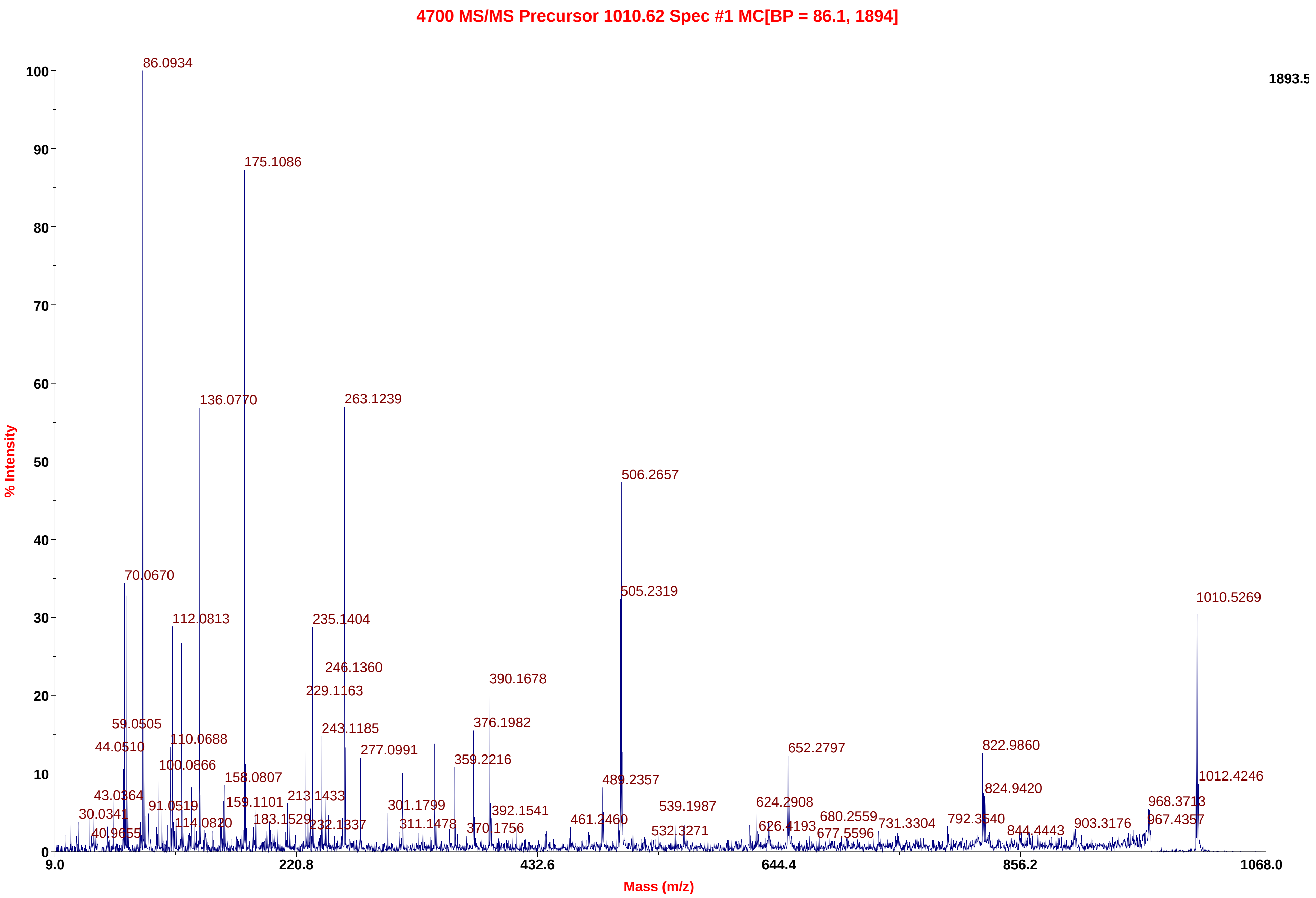

4700 MS/MS Precursor 1010.62 Spec #1 MC[BP = 86.1, 1894]
% Intensity
Mass (m/z)
86.0934
175.1086
136.0770
263.1239
506.2657
505.2319
1010.5269
70.0670
112.0813
235.1404
246.1360
229.1163
390.1678
59.0505
110.0688
376.1982
243.1185
44.0510
277.0991
652.2797
822.9860
100.0866
359.2216
158.0807
489.2357
1012.4246
824.9420
43.0364
213.1433
91.0519
159.1101
301.1799
539.1987
624.2908
968.3713
30.0341
183.1529
392.1541
680.2559
114.0820
232.1337
311.1478
461.2460
626.4193
731.3304
792.3540
903.3176
967.4357
40.9655
370.1756
532.3271
677.5596
844.4443
1893.5

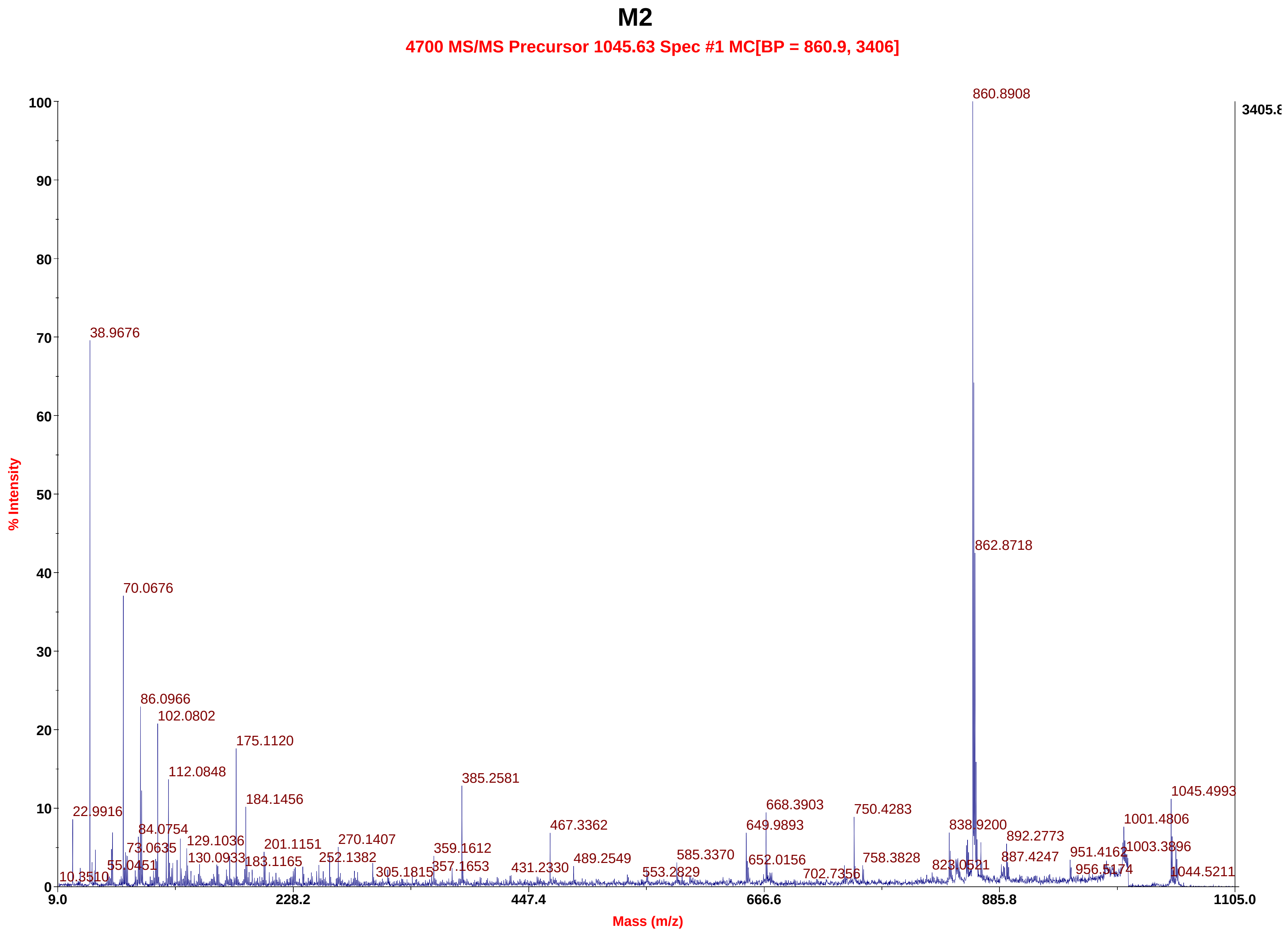
M2
4700 MS/MS Precursor 1045.63 Spec #1 MC[BP = 860.9, 3406]
% Intensity
Mass (m/z)
3405.8
860.8908
862.8718
38.9676
70.0676
86.0966
102.0802
175.1120
112.0848
385.2581
184.1456
22.9916
84.0754
73.0635
129.1036
130.0933
55.0451
10.3510
183.1165
201.1151
270.1407
252.1382
305.1815
359.1612
357.1653
431.2330
467.3362
489.2549
553.2829
585.3370
649.9893
652.0156
668.3903
702.7356
750.4283
758.3828
823.0521
838.9200
887.4247
892.2773
951.4162
956.5174
1001.4806
1003.3896
1044.5211
1045.4993

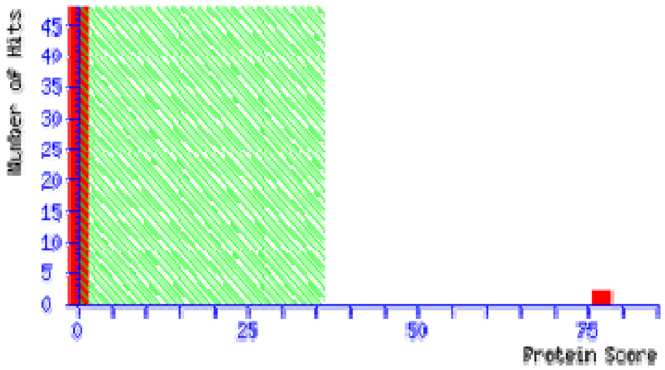

Peptide Summary Report

Format As | Peptide Summary | Help

Significance threshold p< 0.05 | Max. number of hits AUTO

Standard scoring ○ MudPIT scoring ◉ | Ions score or expect cut-off 0 | Show sub-sets 0

Show pop-ups ◉ Suppress pop-ups ○ | Sort unassigned Decreasing Score | Require bold red ☐

Select All | Select None | Search Selected | ☐ **Error tolerant**

1. Q6S9I1_MOUSE **Mass:** 53172 **Score:** 77 **Matches:** 8(1) **Sequences:** 8(1) **emPAI:** 0.58

HMW kininogen-I variant (High molecular weight kininogen I isoform DeltaD5).- Mus musculus (Mouse).

☐ Check to include this hit in error tolerant search

	Query	Observed	Mr(expt)	Mr(calc)	ppm	Miss	Score	Expect	Rank	Unique	Peptide
☑	2	1010.6234	1009.6161	1009.5596	55.9	0	29	0.1	1	U	K.YVIEFIAR.E
☑	6	1105.5889	1104.5816	1104.5134	61.7	0	12	21	1	U	K.SGNQYMLHR.V
☑	8	1249.6459	1248.6386	1248.5663	57.9	0	32	0.2	1	U	K.TDGSPTFYSFK.Y
☑	9	1325.6459	1324.6386	1324.5574	61.4	0	26	0.94	1	U	K.CQALDMTEMAR.R + Carbamidomethyl (C)
☑	13	1515.8025	1514.7952	1514.7075	57.9	0	32	0.2	1	U	R.ENEFFIVTQTCK.I + Carbamidomethyl (C)
	25	1982.0591	1981.0518	1980.9319	60.6	1	2	1.7e+02	9	U	K.VVPTVKCQALDMTEMAR.R + Carbamidomethyl (C); Deamid
☑	45	2216.0903	2215.0830	2214.9423	63.5	0	61	0.00026	1	U	R.FPSLHGDCVALPNGDDGECR.G + 2 Carbamidomethyl (C)
☑	60	2432.2815	2431.2742	2431.1161	65.0	0	8	51	1		K.HLGQSLDCNANVYMRPWENK.V + Carbamidomethyl (C)

Protein View

Match to: Q6S9I1_MOUSE Score: 77
HMW kininogen-I variant (High molecular weight kininogen I isoform DeltaD5).- Mus musculus (Mouse).
Found in search of ppw_B2_129990854101.txt

Nominal mass (M_r): **53172**; Calculated pI value: **4.88**
NCBI BLAST search of Q6S9I1_MOUSE against nr
Unformatted sequence string for pasting into other applications

Taxonomy: Mus musculus
Links to retrieve other entries containing this sequence from NCBI Entrez:
(no taxonomy information for this entry)
(no taxonomy information for this entry)

Variable modifications: Carbamidomethyl (C),Deamidated (NQ),Oxidation (M)
Cleavage by Trypsin: cuts C-term side of KR unless next residue is P
Sequence Coverage: 20%

Matched peptides shown in **Bold Red**

```
  1 MKLITTLLLC SGLLLTLTQG EEAQEIDCND EAVFQAVDFS LKQFNPGVKS
 51 GNQYMLHRVI EGTKTDGSPT FYSFKYLIKE GNCSAQSGLA WQDCDFKDAE
101 EAATGECTAT VGKRENEFFI VTQTCKIAPS KAPILKAYFP CIGCVHAIST
151 DSPDLEPVLK HSIEHFNNNT DHSHLFTLRK VKSAHRQVVA GLNFDITYTI
201 VQTNCSKERF PSLHGDCVAL PNGDDGECRG NLFMDINNKI ANFSQSCTLY
251 SGDDLVEALP KPCPGCPRDI PVDSPELKEV LGHSIAQLNA ENDHPFYYKI
301 DTVKKATSQV VAGTKYVIEF IARETKCSKE SNTELAEDCE IKHLGQSLDC
351 NANVYMRPWE NKVVPTVKCQ ALDMTEMARR PPGFSPFRSV TVQETKEGRT
401 DSDFIEDVVA TTPPYDTGAH DDLIPDIHVQ PDSLSFKLIS DFPEATSPKC
451 PGRPWKPASW EDPNTETTEF SDFDLLDALS
```

Show predicted peptides also

Sort Peptides By ◉ Residue Number ○ Increasing Mass ○ Decreasing Mass

Start – End	Observed	Mr(expt)	Mr(calc)	ppm	Miss	Sequence
50 – 58	1105.5889	1104.5816	1104.5134	62	0	K.SGNQYMLHR.V (Ions score 12)
65 – 75	1249.6459	1248.6386	1248.5663	58	0	K.TDGSPTFYSFK.Y (Ions score 32)
115 – 126	1515.8025	1514.7952	1514.7075	58	0	R.ENEFFIVTQTCK.I Carbamidomethyl
210 – 229	2216.0903	2215.0830	2214.9423	64	0	R.FPSLHGDCVALPNGDDGECR.G 2 Carbam
316 – 323	1010.6234	1009.6161	1009.5596	56	0	K.YVIEFIAR.E (Ions score 29)
343 – 362	2432.2815	2431.2742	2431.1161	65	0	K.HLGQSLDCNANVYMRPWENK.V Carbamid
363 – 379	1982.0591	1981.0518	1980.9319	61	1	K.VVPTVKCQALDMTEMAR.R Carbamidome
369 – 379	1325.6459	1324.6386	1324.5574	61	0	K.CQALDMTEMAR.R Carbamidomethyl (

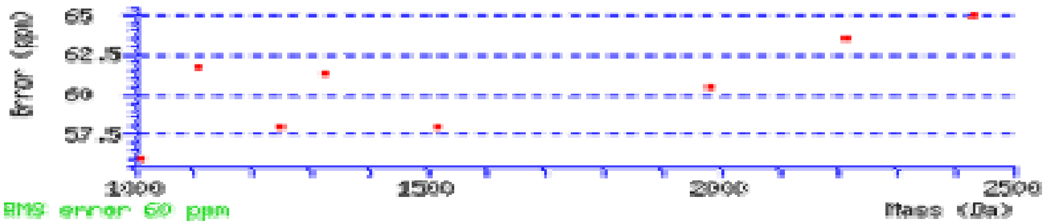

Sample M3

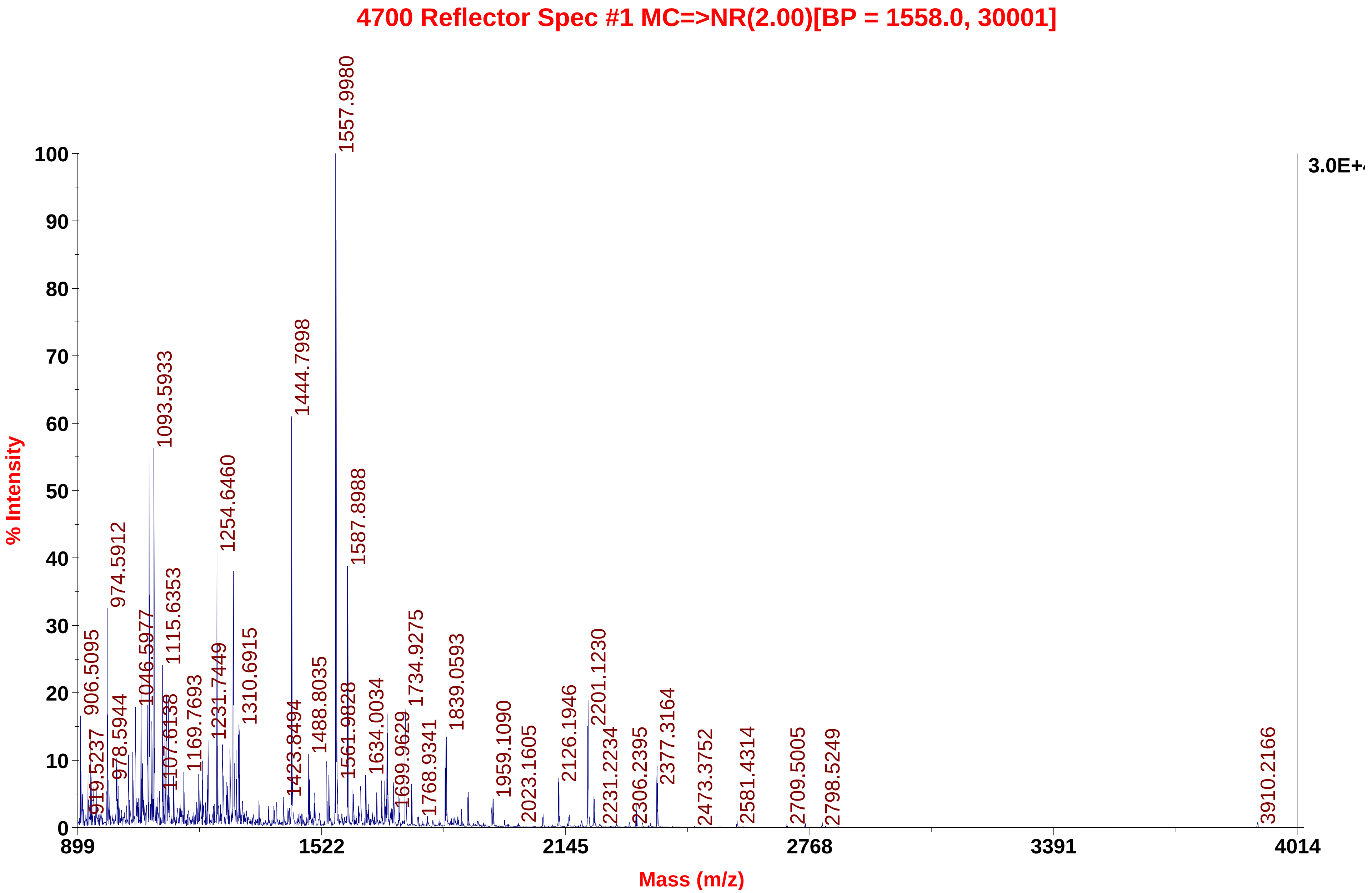
4700 Reflector Spec #1 MC=>NR(2.00)[BP = 1558.0, 30001]
% Intensity
Mass (m/z)
3.0E+4
1557.9980
1444.7998
1093.5933
1254.6460
1587.8988
974.5912
1115.6353
1046.5977
906.5095
1310.6915
1231.7449
1169.7693
1107.6138
978.5944
919.5237
1488.8035
1423.8494
1561.9828
1634.0034
1699.9629
1734.9275
1768.9341
1839.0593
1959.1090
2023.1605
2126.1946
2201.1230
2231.2234
2306.2395
2377.3164
2473.3752
2581.4314
2709.5005
2798.5249
3910.2166
899
1522
2145
2768
3391
4014
0
10
20
30
40
50
60
70
80
90
100

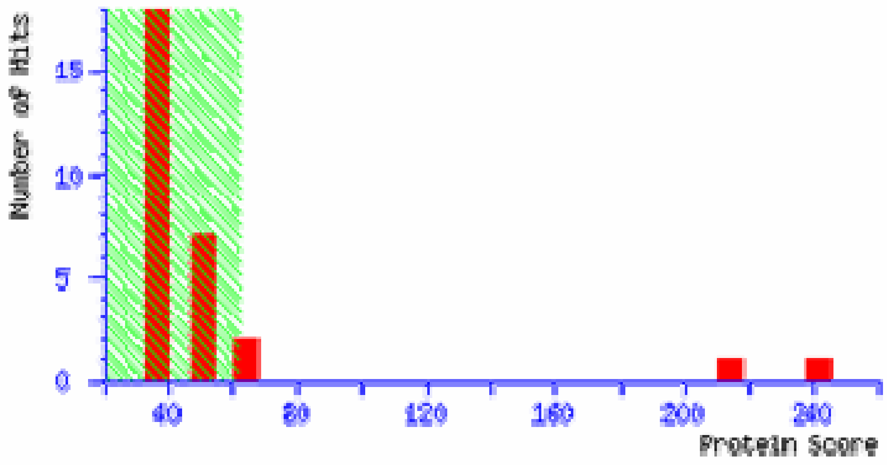

Concise Protein Summary Report

Format As | Concise Protein Summary | Help

Significance threshold p< 0.05 Max. number of hits AUTO

Re-Search All | Search Unmatched

1. VIME_MOUSE **Mass**: 53524 **Score**: 241 **Expect**: 7.3e-20 **Matches**: 61
Vimentin.- Mus musculus (Mouse).
Q5FWJ3_MOUSE **Mass**: 53655 **Score**: 241 **Expect**: 7.3e-20 **Matches**: 61
Vimentin (NOD-derived CD11c +ve dendritic cells cDNA, RIKEN full- length enriched library, clone:F
CAA39807 **Mass**: 53689 **Score**: 233 **Expect**: 4.6e-19 **Matches**: 58
MMVMTM NID: - Mus musculus
Q3U6S1_MOUSE **Mass**: 53641 **Score**: 232 **Expect**: 5.8e-19 **Matches**: 59
Bone marrow macrophage cDNA, RIKEN full-length enriched library, clone:I830119C09 product:vimentin
Q3TFD9_MOUSE **Mass**: 53656 **Score**: 230 **Expect**: 9.2e-19 **Matches**: 59
17 days embryo heart cDNA, RIKEN full-length enriched library, clone:I920087O13 product:vimentin,
AAA40555 **Mass**: 53641 **Score**: 229 **Expect**: 1.2e-18 **Matches**: 58

Protein View

Match to: **VIME_MOUSE** Score: **241** Expect: **7.3e-20**
Vimentin.- Mus musculus (Mouse).
Found in search of pmf_C2_129990848802.txt

Nominal mass (M_r): **53524**; Calculated pI value: **5.06**
NCBI BLAST search of VIME_MOUSE against nr
Unformatted sequence string for pasting into other applications

Taxonomy: Mus musculus

Variable modifications: Carbamidomethyl (C),Deamidated (NQ),Oxidation (M)
Cleavage by Trypsin: cuts C-term side of KR unless next residue is P
Number of mass values searched: **176**
Number of mass values matched: **61**
Sequence Coverage: **85%**

Matched peptides shown in **Bold Red**

```
  1 STRSVSSSSY RRMFGGSGTS SRPSSNRSYV TTSTRTYSLG SALRPSTSRS
 51 LYSSSPGGAY VTRSSAVRLR SSVPGVRLLQ DSVDFSLADA INTEFKNTRT
101 NEKVELQELN DRFANYIDKV RFLEQQNKIL LAELEQLKGQ GKSRLGDLYE
151 EEMRELRRQV DQLTNDKARV EVERDNLAED IMRLREKLQE EMLQREEAES
201 TLQSFRQDVD NASLARLDLE RKVESLQEEI AFLKKLHDEE IQELQAQIQE
251 QHVQIDVDVS KPDLTAALRD VRQQYESVAA KNLQEAEEWY KSKFADLSEA
301 ANRNNDALRQ AKQESNEYRR QVQSLTCEVD ALKGTNESLE RQMREMEENF
351 ALEAANYQDT IGRLQDEIQN MKEEMARHLR EYQDLLNVKM ALDIEIATYR
401 KLLEGEESRI SLPLPTFSSL NLRETNLESL PLVDTHSKRT LLIKTVETRD
451 GQVINETSQH HDDLE
```

Show predicted peptides also

Sort Peptides By ◉ Residue Number ○ Increasing Mass ○ Decreasing Mass

Start – End	Observed	Mr(expt)	Mr(calc)	ppm	Miss	Sequence	
4 – 12	1028.5881	1027.5808	1027.5047	74	1	R.SVSSSSYRR.M	
12 – 27	1699.9598	1698.9525	1698.7856	98	1	R.RMFGGSGTSSRPSSNR.S	Oxida
28 – 49	2391.3157	2390.3084	2390.2190	37	1	R.SYVTTSTRTYSLGSALRPSTSR.S	
50 – 63	1444.7992	1443.7919	1443.6994	64	0	R.SLYSSSPGGAYVTR.S	
78 – 96	2126.1914	2125.1841	2125.0579	59	0	R.LLQDSVDFSLADAINTEFK.N	
100 – 112	1587.8977	1586.8904	1586.7900	63	1	R.TNEKVELQELNDR.F	
104 – 112	1115.6348	1114.6275	1114.5618	59	0	K.VELQELNDR.F	
113 – 121	1125.6698	1124.6625	1124.5978	58	1	R.FANYIDKVR.F	
122 – 128	906.5101	905.5028	905.4607	47	0	R.FLEQQNK.I	
129 – 138	1169.7720	1168.7647	1168.7067	50	0	K.ILLAELEQLK.G	
129 – 142	1540.0029	1538.9956	1538.9032	60	1	K.ILLAELEQLKGQGK.S	

M3

4700 MS/MS Precursor 1295.75 Spec #1 MC[BP = 175.1, 1640]

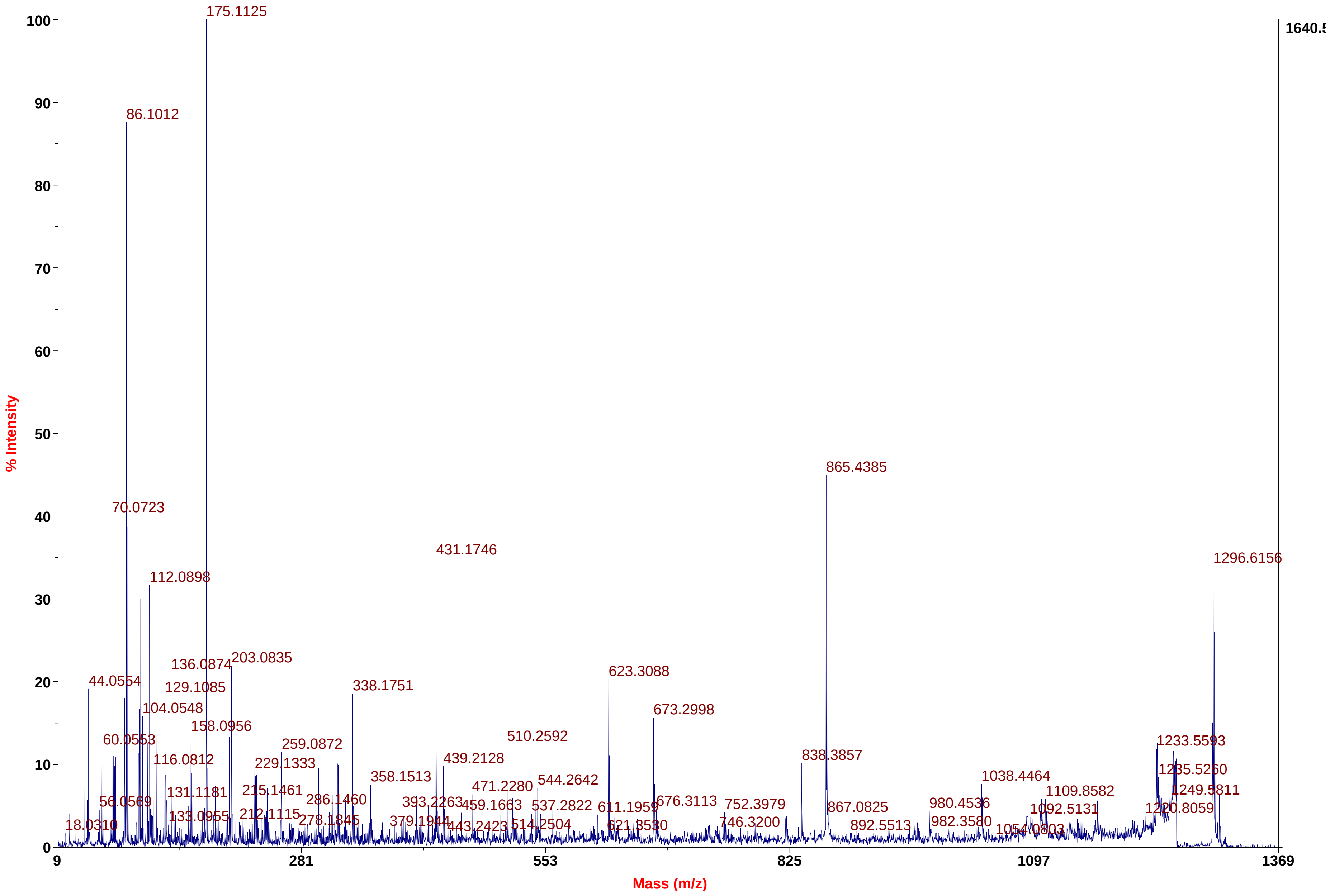

M3

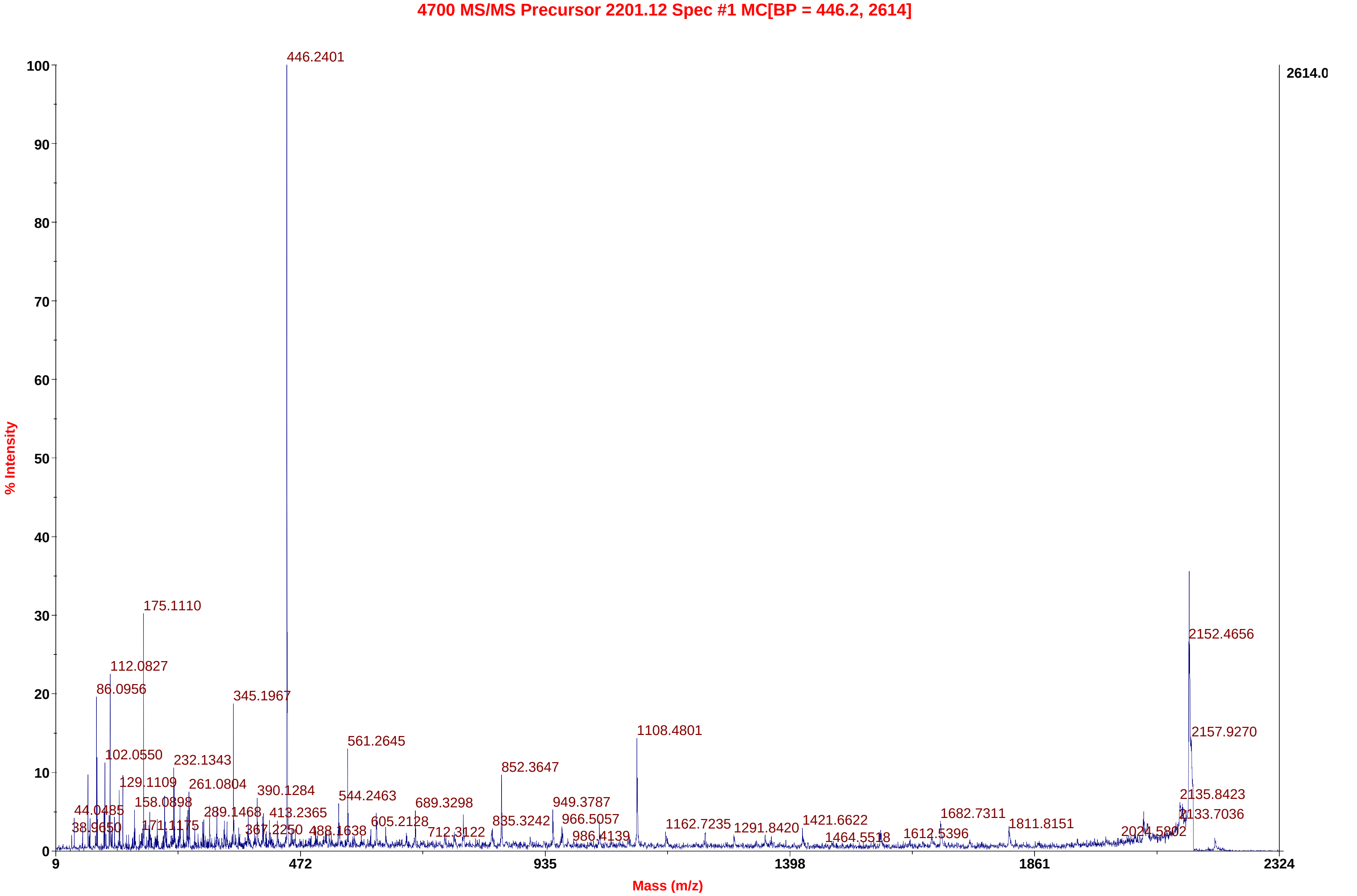
4700 MS/MS Precursor 2201.12 Spec #1 MC[BP = 446.2, 2614]
% Intensity
Mass (m/z)
2614.0
446.2401
175.1110
112.0827
86.0956
345.1967
102.0550
232.1343
129.1109
261.0804
390.1284
158.0898
44.0485
289.1468
413.2365
38.9650
171.1175
367.2250
488.1638
561.2645
544.2463
605.2128
689.3298
712.3122
852.3647
835.3242
949.3787
966.5057
986.4139
1108.4801
1162.7235
1291.8420
1421.6622
1464.5518
1612.5396
1682.7311
1811.8151
2024.5802
2152.4656
2157.9270
2135.8423
2133.7036

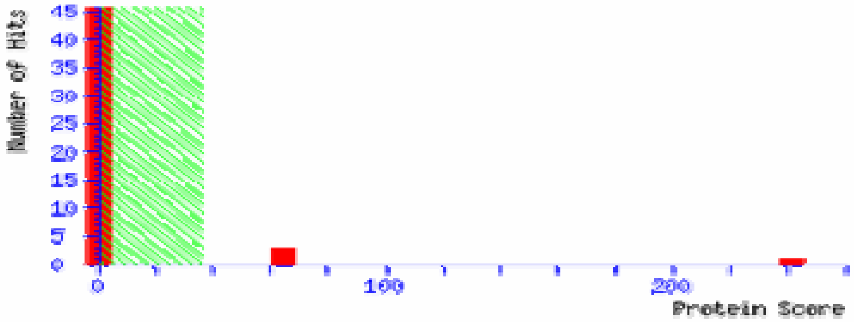

Peptide Summary Report

Format As | Peptide Summary | Help

Significance threshold p< 0.05 | Max. number of hits AUTO

Standard scoring | MudPIT scoring | Ions score or expect cut-off 0 | Show sub-sets 0

Show pop-ups | Suppress pop-ups | Sort unassigned Decreasing Score | Require bold red

Select All | Select None | Search Selected | **Error tolerant**

1. VIME_MOUSE **Mass:** 53524 **Score:** 241 **Matches:** 15(6) **Sequences:** 15(6) **emPAI:** 1.28

Vimentin.- Mus musculus (Mouse).

Check to include this hit in error tolerant search

Query	Observed	Mr(expt)	Mr(calc)	ppm	Miss	Score	Expect	Rank	Unique	Peptide
1	932.5250	931.5177	931.4610	60.9	0	37	0.071	1		K.LLEGEESR.I
4	1081.5635	1080.5562	1080.4948	56.9	1	13	12	1	U	K.QESNEYRR.Q
5	1093.5925	1092.5852	1092.5200	59.7	0	44	0.011	1	U	K.FADLSEAANR.N
6	1115.6348	1114.6275	1114.5618	58.9	0	30	0.29	1		K.VELQELNDR.F
7	1125.6698	1124.6625	1124.5978	57.5	1	27	0.32	1	U	R.FANYIDKVR.F
9	1254.6456	1253.6383	1253.5598	62.7	0	58	0.0005	1	U	R.LGDLYEEEMR.E
11	1287.7448	1286.7375	1286.6579	61.9	1	11	20	1	U	R.QVDQLTNDKAR.V
12	1295.7462	1294.7389	1294.6591	61.7	0	33	0.09	1	U	K.MALDIEIATYR.K
13	1309.6862	1308.6789	1308.5986	61.4	0	32	0.16	1	U	K.NLQEAEEWYK.S
14	1444.7992	1443.7919	1443.6994	64.1	0	29	0.29	1	U	R.SLYSSSPGGAYVTR.S
16	1557.9950	1556.9877	1556.8926	61.1	0	56	0.00014	1	U	R.ISLPLPTFSSLNLR.E
17	1587.8977	1586.8904	1586.7900	63.3	1	66	6.7e-05	1		R.TNEKVELQELNDR.F
18	1688.9358	1687.9285	1687.8199	64.4	1	23	1.5	1	U	R.VEVERDNLAEDIMR.L
19	1734.9248	1733.9175	1733.8076	63.4	1	69	4e-05	1	U	R.LQDEIQNMKEEMAR.H
57	2201.1191	2200.1118	2199.9742	62.6	0	87	6.8e-07	1	U	R.EMEENFALEAANYQDTIGR.L

Protein View

Match to: **VIME_MOUSE** Score: **241**
Vimentin.- Mus musculus (Mouse).
Found in search of ppw_C2_129990854302.txt

Nominal mass (M_r): **53524**; Calculated pI value: **5.06**
NCBI BLAST search of VIME_MOUSE against nr
Unformatted sequence string for pasting into other applications

Taxonomy: Mus musculus

Variable modifications: Carbamidomethyl (C),Deamidated (NQ),Oxidation (M)
Cleavage by Trypsin: cuts C-term side of KR unless next residue is P
Sequence Coverage: 35%

Matched peptides shown in **Bold Red**

```
  1 STRSVSSSSY RRMFGGSGTS SRPSSNRSYV TTSTRTYSLG SALRPSTSRS
 51 LYSSSPGGAY VTRSSAVRLR SSVPGVRLLQ DSVDFSLADA INTEFKNTRT
101 NEKVELQELN DRFANYIDKV RFLEQQNKIL LAELEQLKGQ GKSRLGDLYE
151 EEMRELRRQV DQLTNDKARV EVERDNLAED IMRLREKLQE EMLQREEAES
201 TLQSFRQDVD NASLARLDLE RKVESLQEEI AFLKKLHDEE IQELQAQIQE
251 QHVQIDVDVS KPDLTAALRD VRQQYESVAA KNLQEAEEWY KSKFADLSEA
301 ANRNNDALRQ AKQESNEYRR QVQSLTCEVD ALKGTNESLE RQMREMEENF
351 ALEAANYQDT IGRLQDEIQN MKEEMARHLR EYQDLLNVKM ALDIEIATYR
401 KLLEGEESRI SLPLPTFSSL NLRETNLESL PLVDTHSKRT LLIKTVETRD
451 GQVINETSQH HDDLE
```

Show predicted peptides also

Sort Peptides By ◉ Residue Number ○ Increasing Mass ○ Decreasing Mass

Start - End	Observed	Mr(expt)	Mr(calc)	ppm	Miss	Sequence	
50 - 63	1444.7992	1443.7919	1443.6994	64	0	R.SLYSSSPGGAYVTR.S	(Ions score 29)
100 - 112	1587.8977	1586.8904	1586.7900	63	1	R.TNEKVELQELNDR.F	(Ions score 66)
104 - 112	1115.6348	1114.6275	1114.5618	59	0	K.VELQELNDR.F	(Ions score 30)
113 - 121	1125.6698	1124.6625	1124.5978	58	1	R.FANYIDKVR.F	(Ions score 27)
145 - 154	1254.6456	1253.6383	1253.5598	63	0	R.LGDLYEEEMR.E	(Ions score 58)
159 - 169	1287.7448	1286.7375	1286.6579	62	1	R.QVDQLTNDKAR.V	(Ions score 11)
170 - 183	1688.9358	1687.9285	1687.8199	64	1	R.VEVERDNLAEDIMR.L	(Ions score 23)
282 - 291	1309.6862	1308.6789	1308.5986	61	0	K.NLQEAEEWYK.S	(Ions score 32)
294 - 303	1093.5925	1092.5852	1092.5200	60	0	K.FADLSEAANR.N	(Ions score 44)
313 - 320	1081.5635	1080.5562	1080.4948	57	1	K.QESNEYRR.Q	(Ions score 13)
345 - 363	2201.1191	2200.1118	2199.9742	63	0	R.EMEENFALEAANYQDTIGR.L	(Ions score [illegible]
364 - 377	1734.9248	1733.9175	1733.8076	63	1	R.LQDEIQNMKEEMAR.H	(Ions score 69)
390 - 400	1295.7462	1294.7389	1294.6591	62	0	K.MALDIEIATYR.K	(Ions score 33)
402 - 409	932.5250	931.5177	931.4610	61	0	K.LLEGEESR.I	(Ions score 37)
410 - 423	1557.9950	1556.9877	1556.8926	61	0	R.ISLPLPTFSSLNLR.E	(Ions score 56)

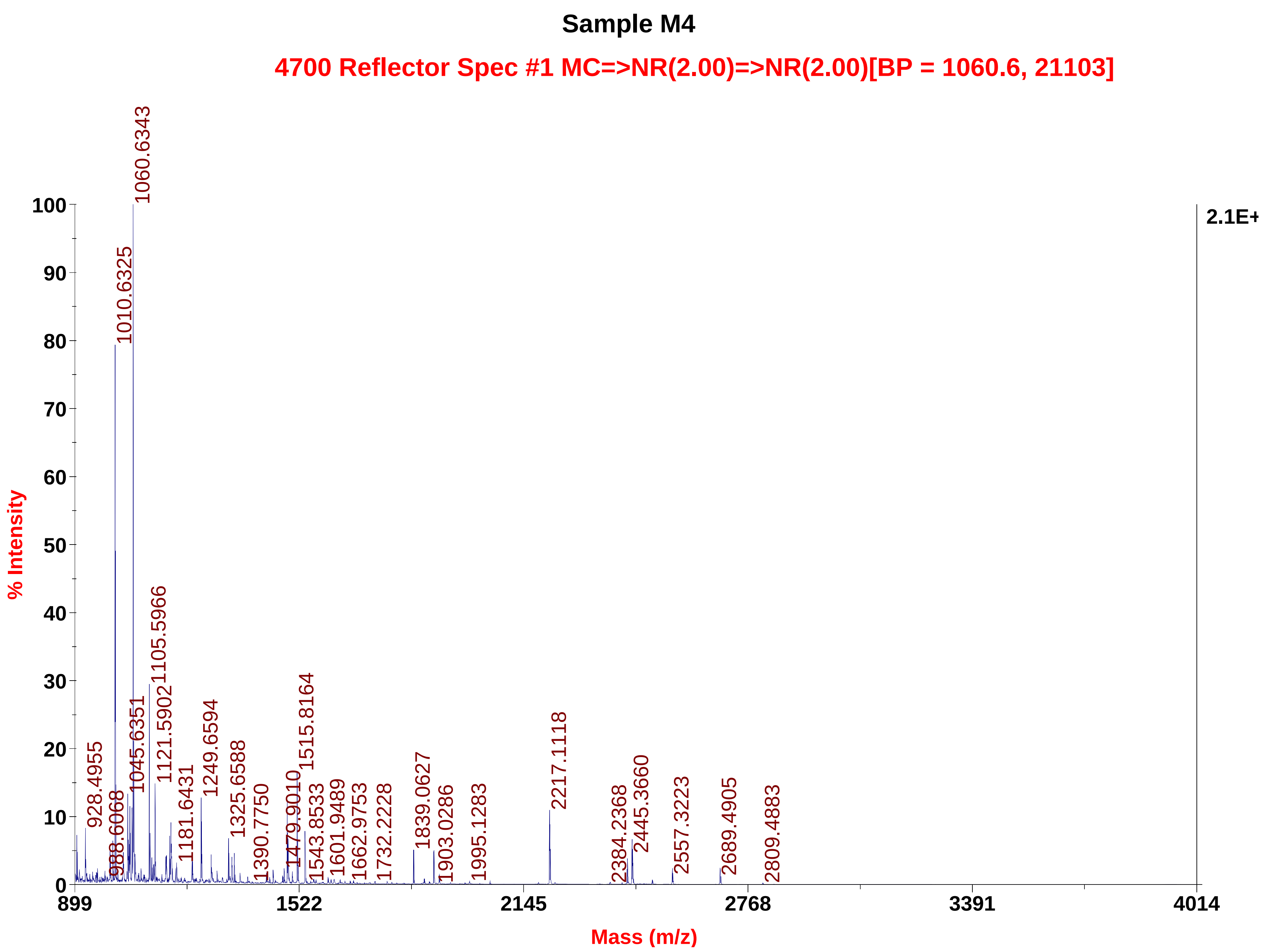

Sample M4
4700 Reflector Spec #1 MC=>NR(2.00)=>NR(2.00)[BP = 1060.6, 21103]
% Intensity
Mass (m/z)
2.1E+
1060.6343
1010.6325
1105.5966
1121.5902
1045.6351
928.4955
988.6068
1181.6431
1249.6594
1325.6588
1390.7750
1479.9010
1515.8164
1543.8533
1601.9489
1662.9753
1732.2228
1839.0627
1903.0286
1995.1283
2217.1118
2384.2368
2445.3660
2557.3223
2689.4905
2809.4883
899
1522
2145
2768
3391
4014
0
10
20
30
40
50
60
70
80
90
100

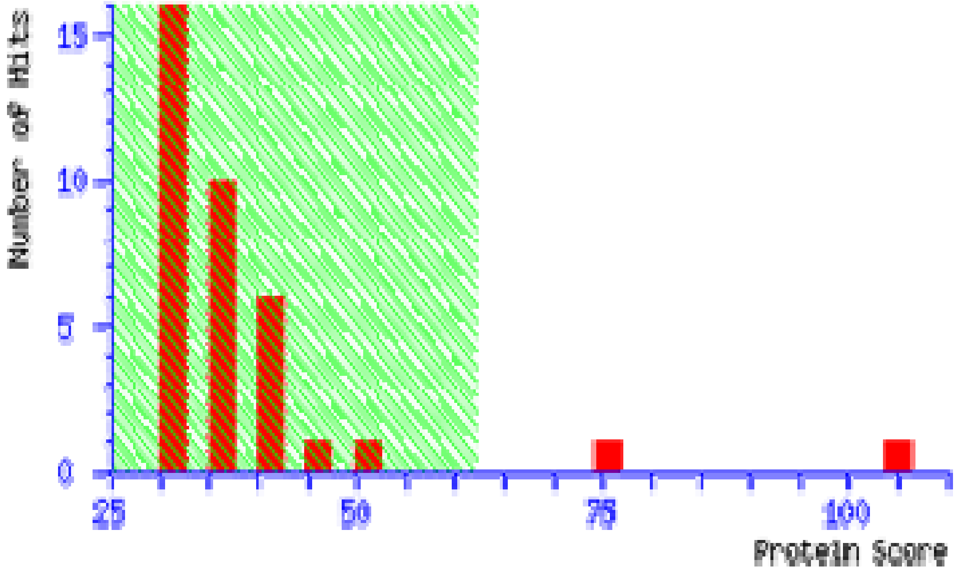

Concise Protein Summary Report

Format As | Concise Protein Summary | Help

Significance threshold p< 0.05 Max. number of hits AUTO

Re-Search All | Search Unmatched

1. AAH18158 **Mass:** 47868 **Score:** 105 **Expect:** 2.9e-06 **Matches:** 28
BC018158 NID: - Mus musculus
Q6S9I1_MOUSE **Mass:** 53172 **Score:** 94 **Expect:** 3.9e-05 **Matches:** 27
HMW kininogen-I variant (High molecular weight kininogen I isoform DeltaD5).- Mus muscu

Protein View

Match to: **AAH18158** Score: **105** Expect: **2.9e-06**
BC018158 NID: – Mus musculus
Found in search of pmf_D2_129990848803.txt

Nominal mass (M_r): **47868**; Calculated pI value: **5.74**
NCBI BLAST search of AAH18158 against nr
Unformatted sequence string for pasting into other applications

Taxonomy: Mus musculus
Links to retrieve other entries containing this sequence from NCBI Entrez:
(no taxonomy information for this entry)

Variable modifications: Carbamidomethyl (C),Deamidated (NQ),Oxidation (M)
Cleavage by Trypsin: cuts C-term side of KR unless next residue is P
Number of mass values searched: **147**
Number of mass values matched: **28**
Sequence Coverage: **56%**

Matched peptides shown in **Bold Red**

```
  1 MKLITTLLLC SGLLLTLTQG EEAQEIDCND EAVFQAVDFS LKQFNPGVKS
 51 GNQYMLHRVI EGTKTDGSPT FYSFKYLIKE GNCSAQSGLA WQDCDFKDAE
101 EAATGECTAT VGKRENEFFI VTQTCKIAPS KAPILKAYFP CIGCVHAIST
151 DSPDLEPVLK HSIEHFNNNT DHSHLFTLRK VKSAHRQVVA GLNFDITYTI
201 VQTNCSKERF PSLHGDCVAL PNGDDGECRG NLFMDINNKI ANFSQSCTLY
251 SGDDLVEALP KPCPGCPRDI PVDSPELKEV LGHSIAQLNA ENDHPFYYKI
301 DTVKKATSQV VAGTKYVIEF IARETKCSKE SNTELAEDCE IKHLGQSLDC
351 NANVYMRPWE NKVVPTVKCQ ALDMTEMARR PPGFSPFRSV TVQETKEGRT
401 RLLRACEYKG RLSKAGAEPA PERQAESSQV KQ
```

Show predicted peptides also

Sort Peptides By ◉ Residue Number ○ Increasing Mass ○ Decreasing Mass

Start – End	Observed	Mr(expt)	Mr(calc)	ppm	Miss	Sequence
50 – 58	1105.5961	1104.5888	1104.5134	68	0	K.SGNQYMLHR.V
50 – 58	1121.5890	1120.5817	1120.5084	65	0	K.SGNQYMLHR.V Oxidation (M)
65 – 75	1249.6566	1248.6493	1248.5663	67	0	K.TDGSPTFYSFK.Y
98 – 114	1765.9282	1764.9209	1764.7948	71	1	K.DAEEAATGECTATVGKR.E Carba
114 – 126	1671.9396	1670.9323	1670.8086	74	1	K.RENEFFIVTQTCK.I Carbamido
115 – 126	1515.8158	1514.8085	1514.7075	67	0	R.ENEFFIVTQTCK.I Carbamidom
137 – 160	2689.4998	2688.4925	2688.2928	74	0	K.AYFPCIGCVHAISTDSPDLEPVLK.H
161 – 180	2449.3303	2448.3230	2448.1570	68	1	K.HSIEHFNNNTDHSHLFTLRK.V 2
208 – 229	2501.2832	2500.2759	2500.0860	76	1	K.ERFPSLHGDCVALPNGDDGECR.G
208 – 229	2502.2778	2501.2705	2501.0700	80	1	K.ERFPSLHGDCVALPNGDDGECR.G
210 – 229	2216.1101	2215.1028	2214.9423	72	0	R.FPSLHGDCVALPNGDDGECR.G 2
[illegible]	[illegible]	[illegible]	[illegible]	[illegible]	[illegible]	[illegible]

M4

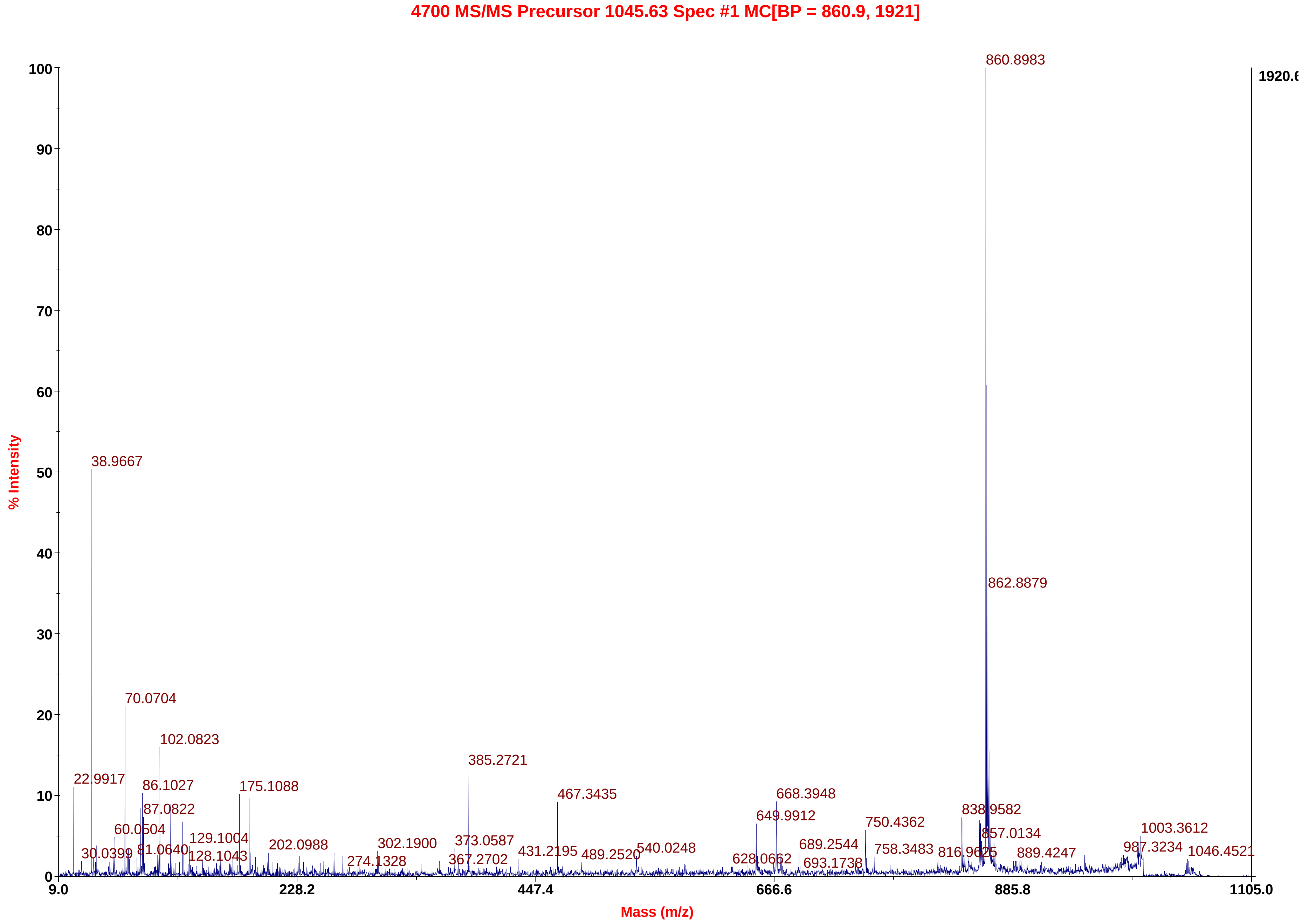
4700 MS/MS Precursor 1045.63 Spec #1 MC[BP = 860.9, 1921]
% Intensity
Mass (m/z)
1920.6
860.8983
862.8879
38.9667
70.0704
102.0823
385.2721
22.9917
86.1027
175.1088
467.3435
668.3948
87.0822
649.9912
838.9582
60.0504
750.4362
1003.3612
857.0134
129.1004
202.0988
302.1900
373.0587
689.2544
758.3483
30.0399
81.0640
128.1043
431.2195
489.2520
540.0248
987.3234
1046.4521
274.1328
367.2702
628.0662
693.1738
816.9625
889.4247
0
10
20
30
40
50
60
70
80
90
100
9.0
228.2
447.4
666.6
885.8
1105.0

M4

4700 MS/MS Precursor 1004.56 Spec #1 MC[BP = 1010.3, 2869]

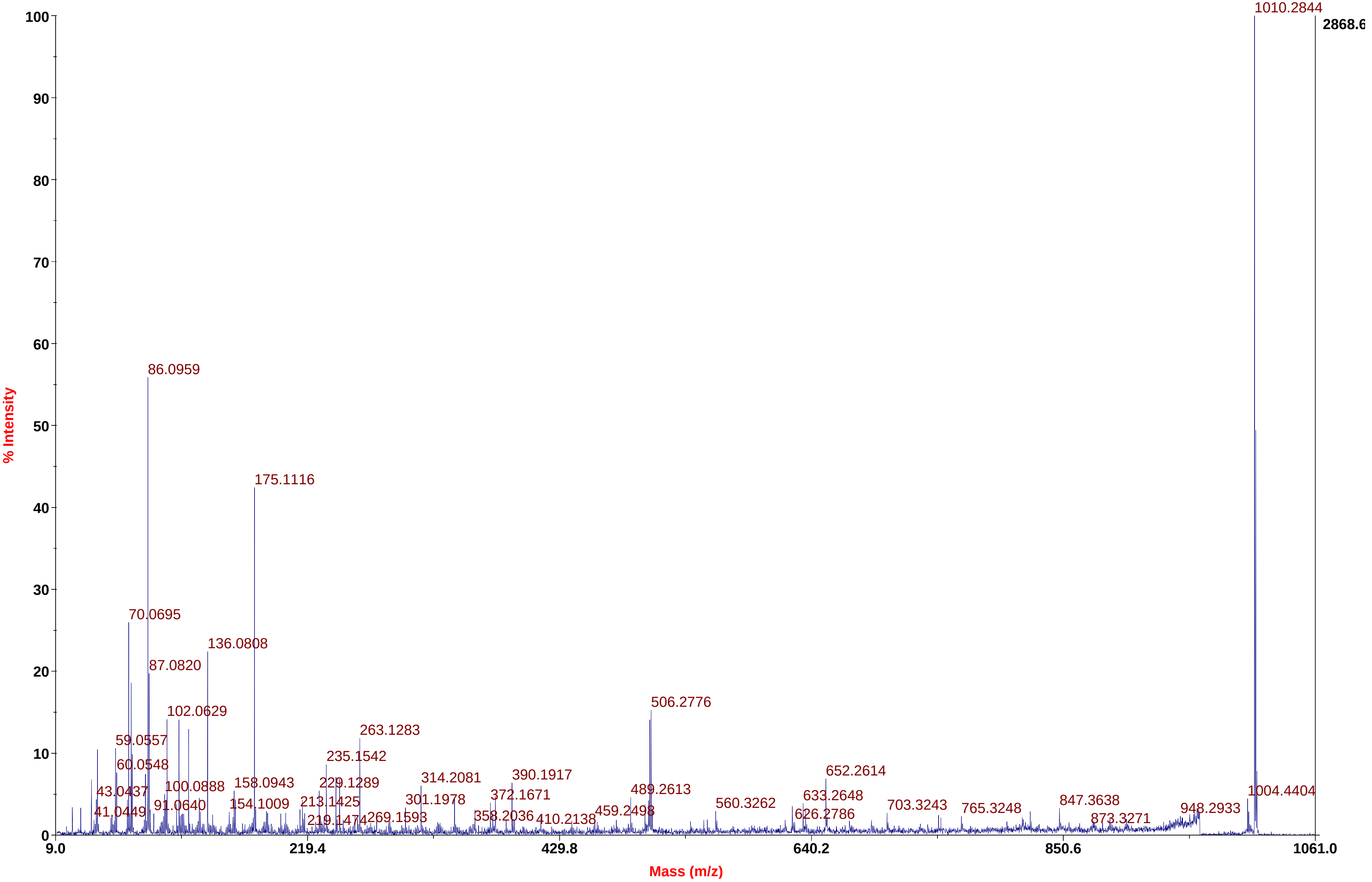

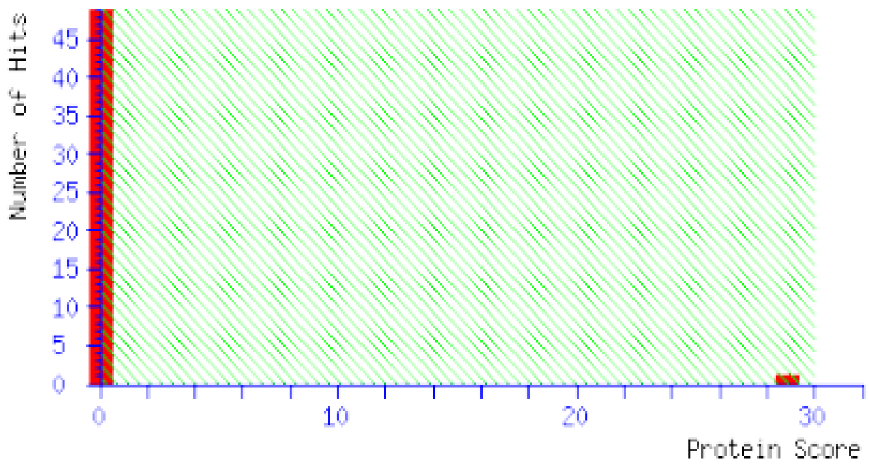

Peptide Summary Report

KNG1_MOUSE Mass: 73056 Score: 29 Matches: 5 (0) Sequences: 5(0) emPAI: 0.18

Kininogen-1 OS=Mus musculus GN=Kng1 PE=1 SV=1

☐ Check to include this hit in error tolerant search

	Query	Observed	Mr(expt)	Mr(calc)	ppm	Miss	Score	Expect	Rank	Unique	Peptide
☑	4	1010.6306	1009.6233	1009.5596	63.1	0	18	0.33	1	U	K.YVIEFIAR.E
☑	9	1112.6533	1111.6460	1111.5761	62.9	0	11	3.5	1	U	R.DIPVDSPELK.E
☑	12	1249.6566	1248.6493	1248.5663	66.5	0	18	1.4	1	U	K.TDGSPTFYSFK.Y
☑	13	1325.6593	1324.6520	1324.5574	71.5	0	17	1.4	1	U	K.CQALDMTEMAR.R + Carbamidomethyl (C)
☑	47	2216.1101	2215.1028	2214.9423	72.5	0	30	0.088	1	U	R.FPSLHGDCVALPNGDDGECR.G + 2 Carbamidomethyl (C)

Protein View

Match to: **KNG1_MOUSE** Score: **29**
Kininogen-1 OS=Mus musculus GN=Kng1 PE=1 SV=1
Found in search of E:\Dekstop 30.5.2011\Muscle\Dekstop 2.5.11\Muscle DU\Muscle data
\sample M4\ppw_D2_129990854403.txt

Nominal mass (M_r): **73056**; Calculated pI value: **6.05**

NCBI BLAST search of KNG1_MOUSE against nr
Unformatted sequence string for pasting into other applications

Taxonomy: Mus musculus

Variable modifications: Carbamidomethyl (C),Deamidated (NQ),Oxidation (M)
Cleavage by Trypsin: cuts C-term side of KR unless next residue is P
Sequence Coverage: 9%

Matched peptides shown in **Bold Red**

```
  1 MKLITTLLLC SGLLLTLTQG EEAQEIDCND EAVFQAVDFS LKQFNPGVKS
 51 GNQYMLHRVI EGTKTDGSPT FYSFKYLIKE GNCSAQSGLA WQDCDFKDAE
101 EAATGECTAT VGKRENEFFI VTQTCKIAPS KAPILKAYFP CIGCVHAIST
151 DSPDLEPVLK HSIEHFNNNT DHSHLFTLRK VKSAHRQVVA GLNFDITYTI
201 VQTNCSKERF PSLHGDCVAL PNGDDGECRG NLFMDINNKI ANFSQSCTLY
251 SGDDLVEALP KPCPGCPRDI PVDSPELKEV LGHSIAQLNA ENDHPFYYKI
301 DTVKKATSQV VAGTKYVIEF IARETKCSKE SNTELAEDCE IKHLGQSLDC
351 NANVYMRPWE NKVVPTVKCQ ALDMTEMARR PPGFSPFRSV TVQETKEGRT
401 VSPPYIAREQ EERDAETEQG PTHGHGWLHE KQIKANKNHR GHKHGHDHGH
451 WSPRRHGLGH GHQKPHGLGH GHQLKLDYLR HQREDGDDHT HTVGHGHGHG
501 HGHGHGHGHG HGHGHGHGHG HGHGKHTNKD KNSVKQTTQR TESLASSSEY
551 STTSTQMQGR TEGPTLTPPR AQPTVTSSGF QDSDFIEDVV ATTPPYDTGA
601 HDDLIPDIHV QPDSLSFKLI SDFPEATSPK CPGRPWKPAS WEDPNTETTE
651 FSDFDLLDAL S
```

Show predicted peptides also

Sort Peptides By ◉ Residue Number ○ Increasing Mass ○ Decreasing Mass

Start - End	Observed	Mr(expt)	Mr(calc)	ppm	Miss	Sequence
65 - 75	1249.6566	1248.6493	1248.5663	67	0	K.TDGSPTFYSFK.Y (Ions score 18)
210 - 229	2216.1101	2215.1028	2214.9423	72	0	R.FPSLHGDCVALPNGDDGECR.G 2 Carbamidomethyl (C) (Ions score 30)

Sample M5

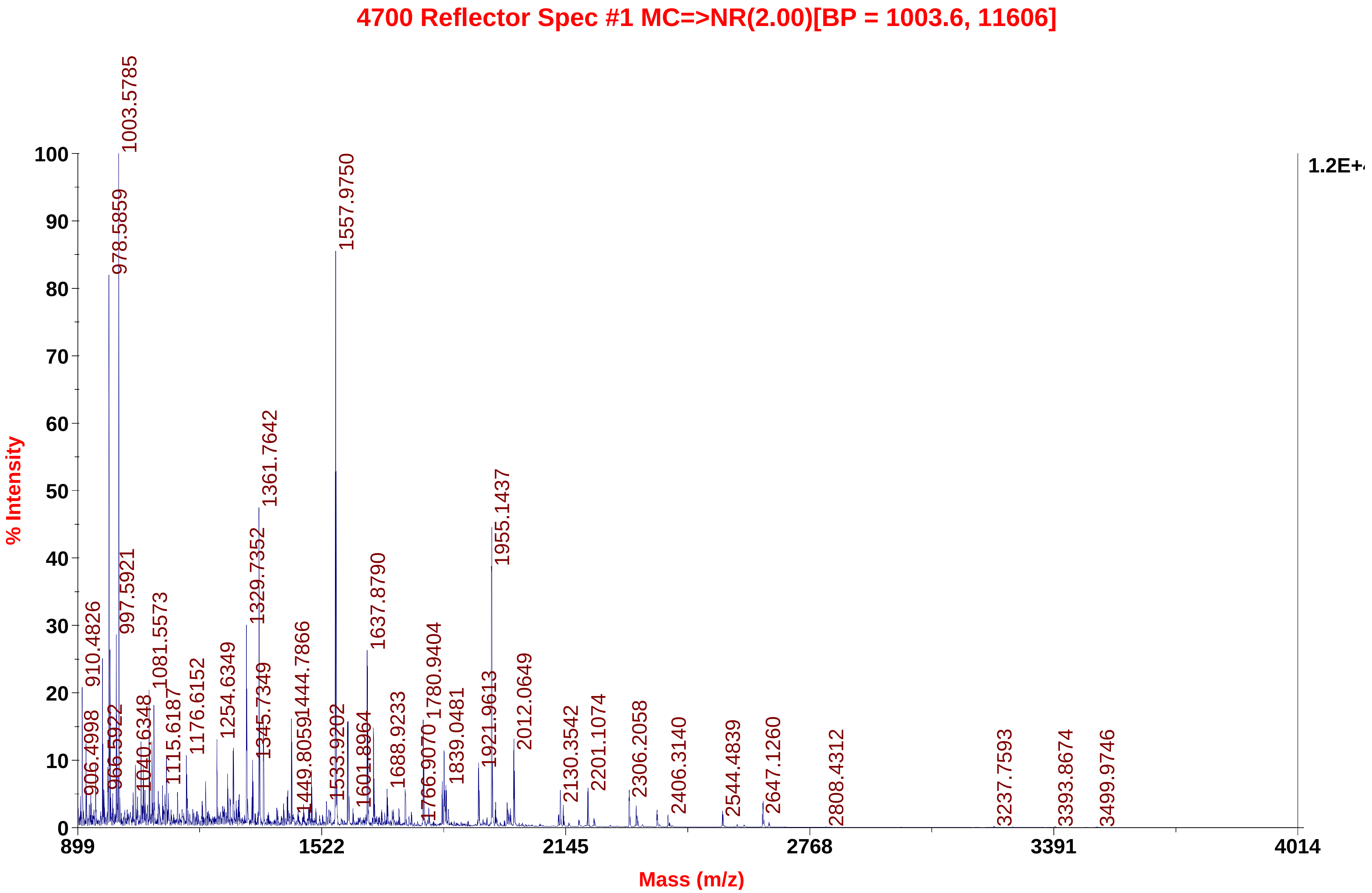

4700 Reflector Spec #1 MC=>NR(2.00)[BP = 1003.6, 11606]
% Intensity
Mass (m/z)
1.2E+4
1003.5785
978.5859
1557.9750
1361.7642
1955.1437
997.5921
1329.7352
910.4826
1081.5573
1637.8790
1444.7866
1780.9404
1254.6349
1176.6152
2012.0649
1921.9613
1115.6187
1345.7349
1839.0481
1688.9233
1040.6348
956.5922
906.4998
1449.8059
1533.9202
1601.8964
1766.9070
2130.3542
2201.1074
2306.2058
2406.3140
2544.4839
2647.1260
2808.4312
3237.7593
3393.8674
3499.9746

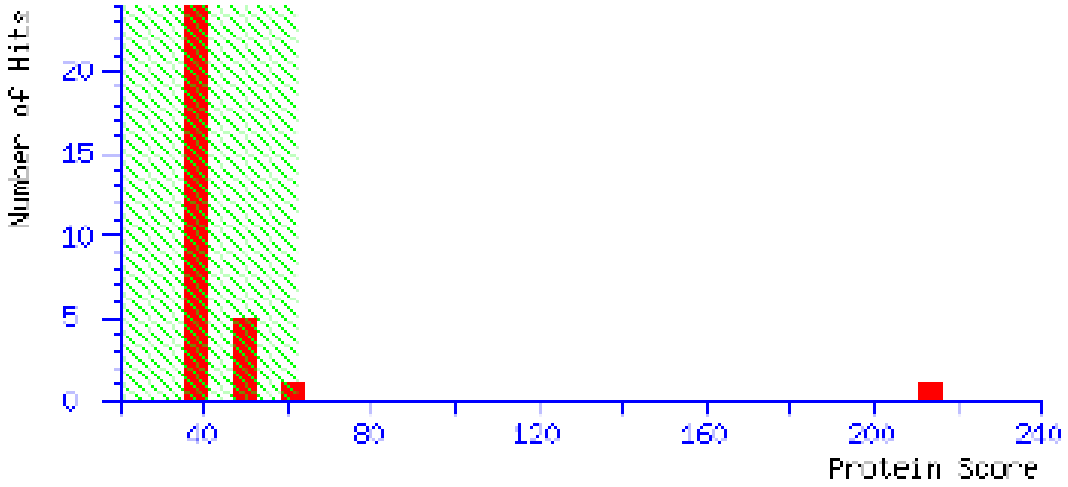

Concise Protein Summary Report

Format As | Concise Protein Summary | Help

Significance threshold p< 0.05

Max. number of hits AUTO

Re-Search All | Search Unmatched

1. CAA69019 **Mass:** 51533 **Score:** 214 **Expect:** 3.7e-17 **Matches:** 23
MMVIMENT NID: - Mus musculus
VIME_MOUSE **Mass:** 53524 **Score:** 211 **Expect:** 7.3e-17 **Matches:** 23
Vimentin.- Mus musculus (Mouse).
A43803 **Mass:** 53597 **Score:** 211 **Expect:** 7.3e-17 **Matches:** 23
vimentin - mouse
Q5FWJ3_MOUSE **Mass:** 53655 **Score:** 211 **Expect:** 7.3e-17 **Matches:** 23

Protein View

Match to: **CAA69019** Score: **214** Expect: **3.7e-17**
MMVIMENT NID: - Mus musculus
Found in search of E:\Dekstop 30.5.2011\Muscle\sample M5\sample M5 \pmf_F19_130406586900.txt

Nominal mass (M_r): **51533**; Calculated pI value: **4.96**

NCBI BLAST search of CAA69019 against nr
Unformatted sequence string for pasting into other applications

Taxonomy: Mus musculus

Cleavage by Trypsin: cuts C-term side of KR unless next residue is P
Number of mass values searched: **60**
Number of mass values matched: **23**
Sequence Coverage: **51%**

Matched peptides shown in **Bold Red**

```
  1 SSRPSSNRSY VTTSTRTYSL GSALRPSTSR SLYSSSPGGA YVTRSSAVRL
 51 RSSVPGVRLL QDSVDFSLAD AINTEFKNTR TNEKVELQEL NDRFANYIDK
101 VRFLEQQNKI LLAELEQLKG QGKSRLGDLY EEEMRELRRQ VDQLTNDKAR
151 VEVERDNLAE DIMRLREKLQ EEMLQREEAE STLQSFRQDV DNASLARLDL
201 ERKVESLQEE IAFLKKLHDE EIQELQAQIQ EQHVQIDVDV SKPDLTAALR
251 DVRQQYESVA AKNLQEAEEW YKSKFADLSE AANRNNDALR QAKQESNEYR
301 RQVQSLTCEV DALKGTNESL ERQMREMEEN FALEAANYQD TIGRLQDEIQ
351 NMKEEMARHL REYQDLLNVK MALDIEIATY RKLLEGEESR ISLPLPTFSS
401 LNLRETNLES LPLVDTHSKR TLLIKTVETR DGQVINETSQ HHDDLE
```

Show predicted peptides also

Sort Peptides By ◉ Residue Number ○ Increasing Mass ○ Decreasing Mass

Start - End	Observed	Mr(expt)	Mr(calc)	ppm	Miss	Sequence
17 - 30	1495.8473	1494.8400	1494.7790	41	0	R.TYSLGSALRPSTSR.S
31 - 44	1444.7740	1443.7667	1443.6994	47	0	R.SLYSSSPGGAYVTR.S
81 - 93	1587.8723	1586.8650	1586.7900	47	1	R.TNEKVELQELNDR.F
85 - 93	1115.6179	1114.6106	1114.5618	44	0	K.VELQELNDR.F
94 - 102	1125.6492	1124.6419	1124.5978	39	1	R.FANYIDKVR.F
126 - 135	1254.6244	1253.6171	1253.5598	46	0	R.LGDLYEEEMR.E
140 - 150	1287.7262	1286.7189	1286.6579	47	1	R.QVDQLTNDKAR.V
151 - 164	1688.9004	1687.8931	1687.8199	43	1	R.VEVERDNLAEDIMR.

M5

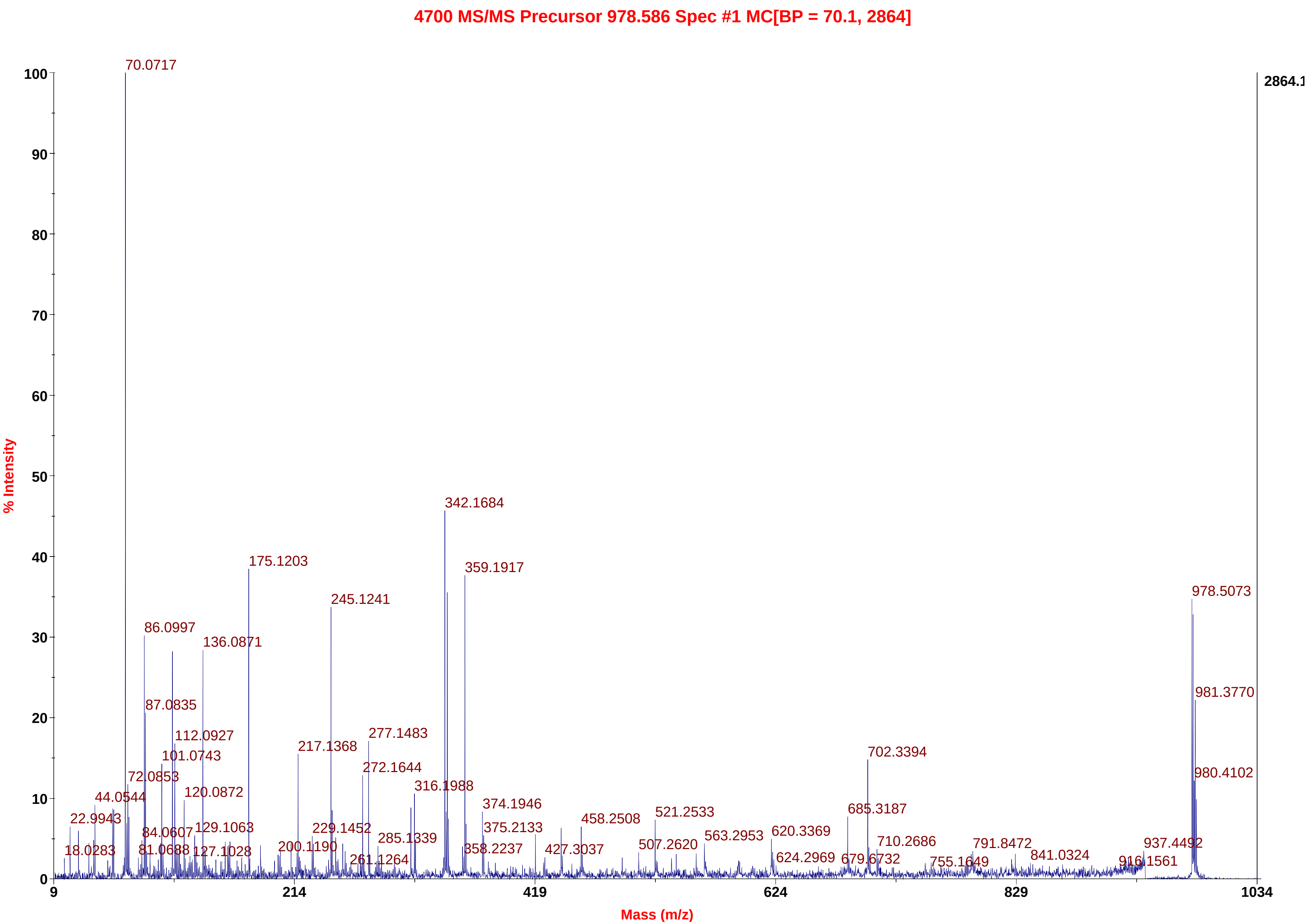

4700 MS/MS Precursor 978.586 Spec #1 MC[BP = 70.1, 2864]
% Intensity
Mass (m/z)
2864.1
70.0717
342.1684
175.1203
359.1917
245.1241
978.5073
86.0997
136.0871
981.3770
87.0835
112.0927
101.0743
217.1368
277.1483
272.1644
702.3394
72.0853
980.4102
120.0872
316.1988
44.0544
374.1946
685.3187
521.2533
458.2508
22.9943
375.2133
129.1063
229.1452
620.3369
84.0607
563.2953
285.1339
710.2686
791.8472
937.4492
200.1190
507.2620
18.0283
81.0688
127.1028
358.2237
427.3037
841.0324
624.2969
679.6732
261.1264
916.1561
755.1649

M5

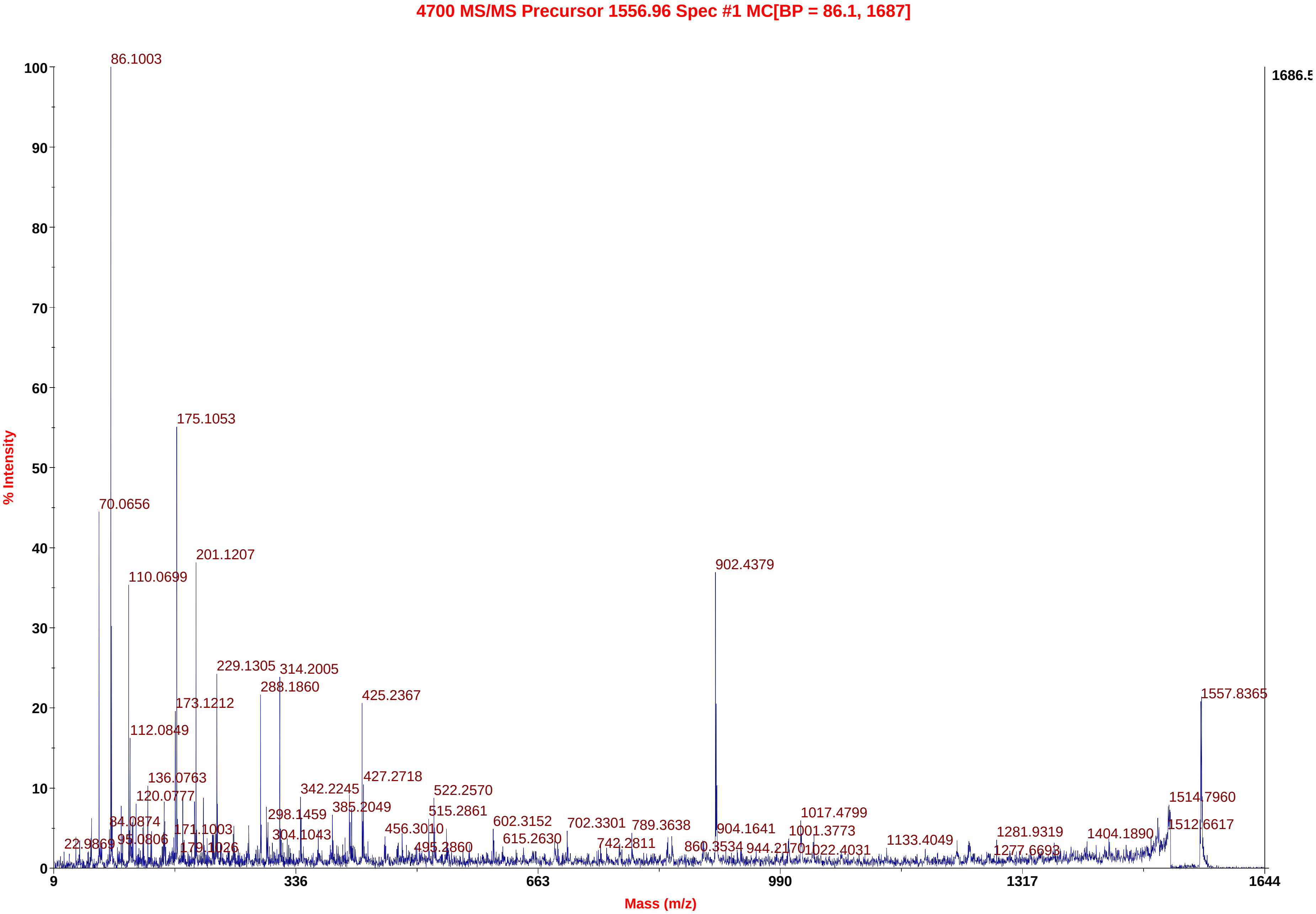

4700 MS/MS Precursor 1556.96 Spec #1 MC[BP = 86.1, 1687]
% Intensity
Mass (m/z)
86.1003
175.1053
70.0656
201.1207
110.0699
902.4379
229.1305
314.2005
288.1860
425.2367
1557.8365
173.1212
112.0849
136.0763
427.2718
342.2245
522.2570
120.0777
1514.7960
385.2049
298.1459
515.2861
1017.4799
84.0874
171.1003
602.3152
702.3301
789.3638
456.3010
904.1641
1001.3773
1512.6617
95.0806
304.1043
615.2630
1281.9319
1404.1890
22.9869
179.1026
495.2860
742.2811
860.3534
944.2170
1022.4031
1133.4049
1277.6693
1686.5

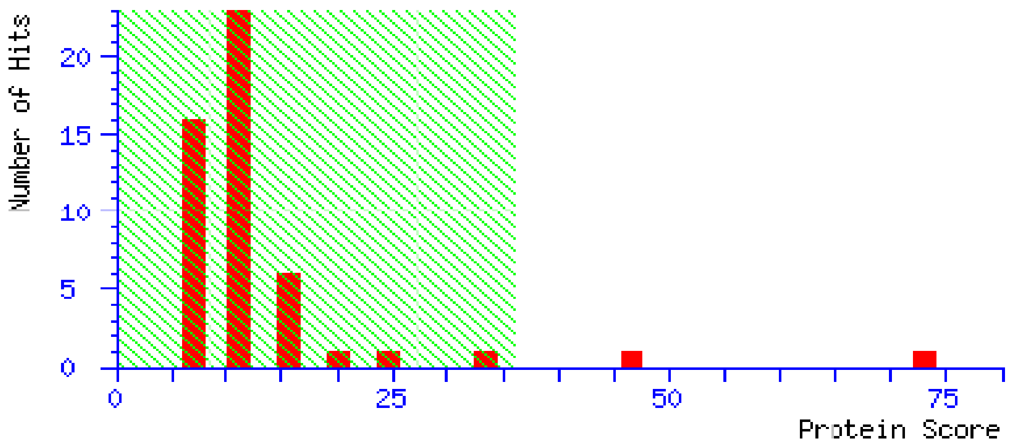

Peptide Summary Report

VIME_MOUSE **Mass:** 53524 **Score:** 73 **Matches:** 3(0) **Sequences:** 3(0)

Vimentin.- Mus musculus (Mouse).

☐ Check to include this hit in error tolerant search

	Query	Observed	Mr(expt)	Mr(calc)	ppm	Miss	Score	Expect	Rank	Unique	Peptide
☑	9	1444.7740	1443.7667	1443.6994	46.6	0	31	0.25	1	U	R.SLYSSSPGGAYVTR.S
☑	10	1557.9629	1556.9556	1556.8926	40.5	0	29	0.12	1	U	R.ISLPLPTFSSLNLR.E
☑	11	1587.8723	1586.8650	1586.7900	47.3	1	13	13	1	U	R.TNEKVELQELNDR.F

Protein View

Match to: **VIME_MOUSE** Score: **73**
Vimentin.- Mus musculus (Mouse).
Found in search of E:\Dekstop 30.5.2011\Muscle\sample M5\sample M5
\ppw_F19_130406591600.txt

Nominal mass (M_r): **53524**; Calculated pI value: **5.06**
NCBI BLAST search of VIME_MOUSE against nr
Unformatted sequence string for pasting into other applications

Taxonomy: Mus musculus

Variable modifications: Carbamidomethyl (C),Deamidated (NQ),Oxidation (M)
Cleavage by Trypsin: cuts C-term side of KR unless next residue is P
Sequence Coverage: **8%**

Matched peptides shown in **Bold Red**

```
  1 STRSVSSSSY RRMFGGSGTS SRPSSNRSYV TTSTRTYSLG SALRPSTSRS
 51 LYSSSPGGAY VTRSSAVRLR SSVPGVRLLQ DSVDFSLADA INTEFKNTRT
101 NEKVELQELN DRFANYIDKV RFLEQQNKIL LAELEQLKGQ GKSRLGDLYE
151 EEMRELRRQV DQLTNDKARV EVERDNLAED IMRLREKLQE EMLQREEAES
201 TLQSFRQDVD NASLARLDLE RKVESLQEEI AFLKKLHDEE IQELQAQIQE
251 QHVQIDVDVS KPDLTAALRD VRQQYESVAA KNLQEAEEWY KSKFADLSEA
301 ANRNNDALRQ AKQESNEYRR QVQSLTCEVD ALKGTNESLE RQMREMEENF
351 ALEAANYQDT IGRLQDEIQN MKEEMARHLR EYQDLLNVKM ALDIEIATYR
401 KLLEGEESRI SLPLPTFSSL NLRETNLESL PLVDTHSKRT LLIKTVETRD
451 GQVINETSQH HDDLE
```

Show predicted peptides also

Sort Peptides By ◉ Residue Number ○ Increasing Mass ○ Decreasing Mass

Start - End	Observed	Mr(expt)	Mr(calc)	ppm	Miss	Sequence
50 - 63	1444.7740	1443.7667	1443.6994	47	0	R.SLYSSSPGGAYVTR.S (Ions score 31)
100 - 112	1587.8723	1586.8650	1586.7900	47	1	R.TNEKVELQELNDR.F (Ions score 13)
410 - 423	1557.9629	1556.9556	1556.8926	40	0	R.ISLPLPTFSSLNLR.E (Ions score 29)

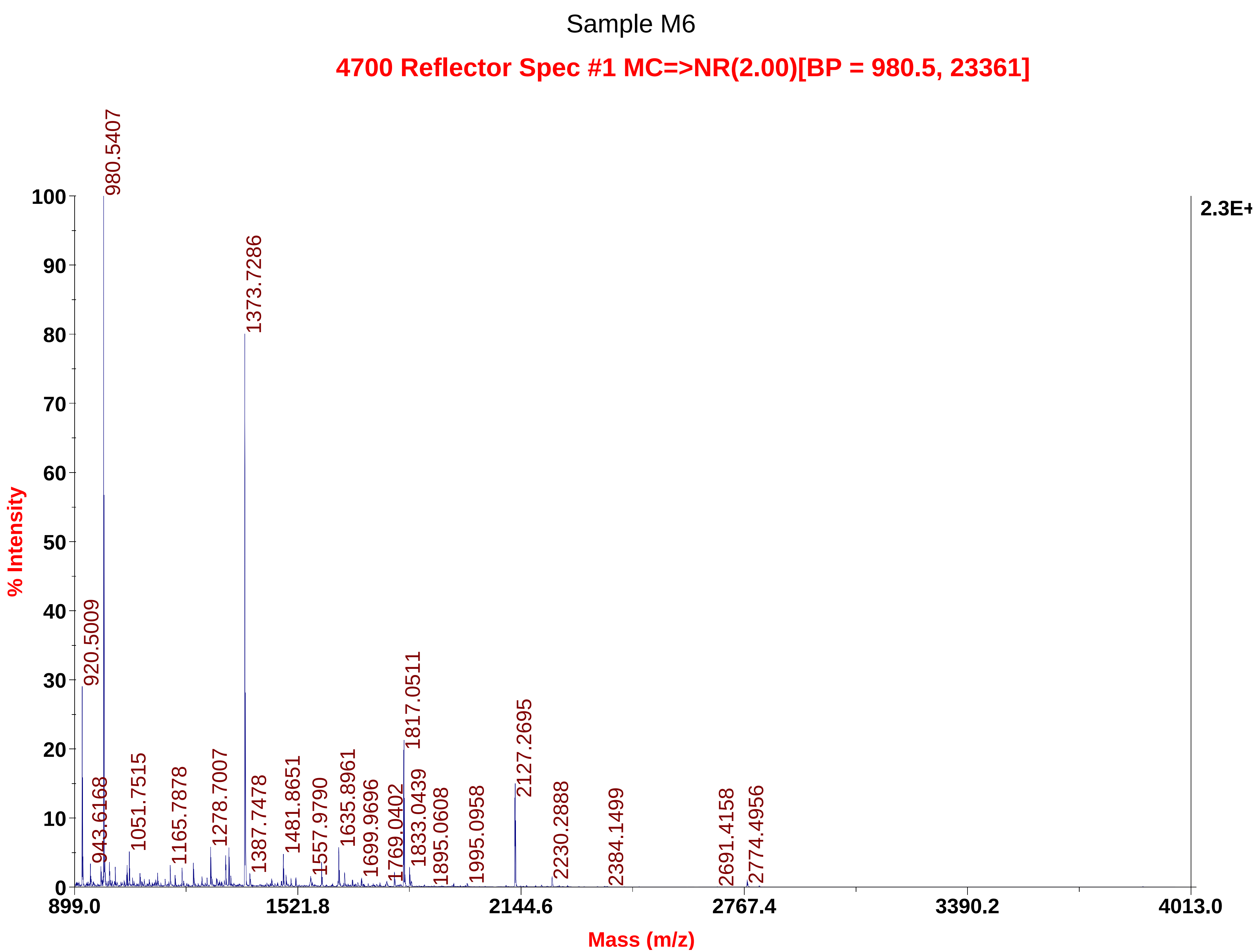
Sample M6
4700 Reflector Spec #1 MC=>NR(2.00)[BP = 980.5, 23361]
% Intensity
Mass (m/z)
2.3E+
980.5407
1373.7286
920.5009
943.6168
1051.7515
1165.7878
1278.7007
1387.7478
1481.8651
1557.9790
1635.8961
1699.9696
1769.0402
1817.0511
1833.0439
1895.0608
1995.0958
2127.2695
2230.2888
2384.1499
2691.4158
2774.4956
899.0
1521.8
2144.6
2767.4
3390.2
4013.0
0
10
20
30
40
50
60
70
80
90
100

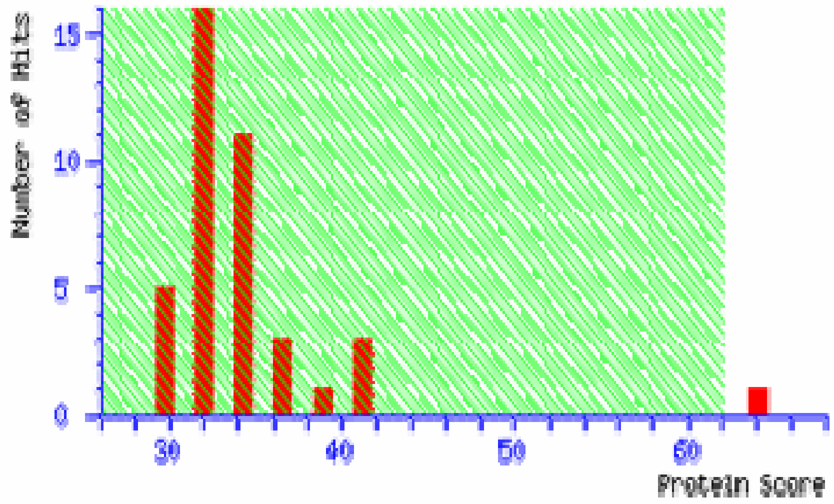

Concise Protein Summary Report

Format As | Concise Protein Summary | Help

Significance threshold p< 0.05 Max. number of hits AUTO

Re-Search All | Search Unmatched

1. Q3UBS3_MOUSE **Mass:** 38727 **Score:** 64 **Expect:** 0.036 **Matches:** 21
   Bone marrow macrophage cDNA, RIKEN full-length enriched library, clone:I830015M19 product:haptoglobin,
   Q63927_9MURI **Mass:** 38750 **Score:** 53 **Expect:** 0.48 **Matches:** 19
   Haptoglobin.- Mus sp.
   HPMS **Mass:** 38821 **Score:** 52 **Expect:** 0.54 **Matches:** 19
   haptoglobin precursor - mouse

Protein View

Match to: **Q3UBS3_MOUSE** Score: **64** Expect: **0.036**
Bone marrow macrophage cDNA, RIKEN full-length enriched library, clone:I830015M19 product:haptoglobin,
Found in search of pmf_F2_129990848905.txt

Nominal mass (M_r): **38727**; Calculated pI value: **5.88**
NCBI BLAST search of Q3UBS3_MOUSE against nr
Unformatted sequence string for pasting into other applications

Taxonomy: Mus musculus
Links to retrieve other entries containing this sequence from NCBI Entrez:
(no taxonomy information for this entry)
(no taxonomy information for this entry)
(no taxonomy information for this entry)
(no taxonomy information for this entry)
(no taxonomy information for this entry)
(no taxonomy information for this entry)
(no taxonomy information for this entry)

Variable modifications: Carbamidomethyl (C),Deamidated (NQ),Oxidation (M)
Cleavage by Trypsin: cuts C-term side of KR unless next residue is P
Number of mass values searched: **129**
Number of mass values matched: **21**
Sequence Coverage: **54%**

Matched peptides shown in **Bold Red**

```
  1 MRALGAVVTL LLWGQLFAVE LGNDAMDFED DSCPKPPEIA NGYVEHLVRY
 51 RCRQFYRLRA EGDGVYTLND EKQWVNTVAG EKLPECEAVC GKPKHPVDQV
101 QRIIGGSMDA KGSFPWQAKM ISRHGLTTGA TLISDQWLLT TAKNLFLNHS
151 ETASAKDITP TLTLYVGKNQ LVEIEKVVLH PNHSVVDIGL IKLKQRVLVT
201 ERVMPICLPS KDYIAPGRVG YVSGWGRNAN FRFTDRLKYV MLPVADQDKC
251 VVHYENSTVP EKKNLTSPVG VQPILNEHTF CAGLTKYQED TCYGDAGSAF
301 AIHDMEEDTW YAAGILSFDK SCAVAEYGVY VRATDLKDWV QETMAKN
```

Show predicted peptides also

Sort Peptides By ◉ Residue Number ○ Increasing Mass ○ Decreasing Mass

Start – End	Observed	Mr(expt)	Mr(calc)	ppm	Miss	Sequence
83 – 94	1387.7445	1386.7372	1386.6635	53	0	K.LPECEAVCGKPK.H 2 Carbamidomethyl (
95 – 102	978.5474	977.5401	977.5043	37	0	K.HPVDQVQR.I
103 – 119	1809.0271	1808.0198	1807.8927	70	1	R.IIGGSMDAKGSFPWQAK.M Oxidation (M)
112 – 119	920.5013	919.4940	919.4552	42	0	K.GSFPWQAK.M
124 – 143	2127.2664	2126.2591	2126.1372	57	0	R.HGLTTGATLISDQWLLTTAK.N
157 – 168	1320.8011	1319.7938	1319.7337	46	0	K.DITPTLTLYVGK.N
169 – 176	972.5767	971.5694	971.5287	42	0	K.NQLVEIEK.V

M6

4700 MS/MS Precursor 980.541 Spec #1 MC[BP = 136.1, 5532]

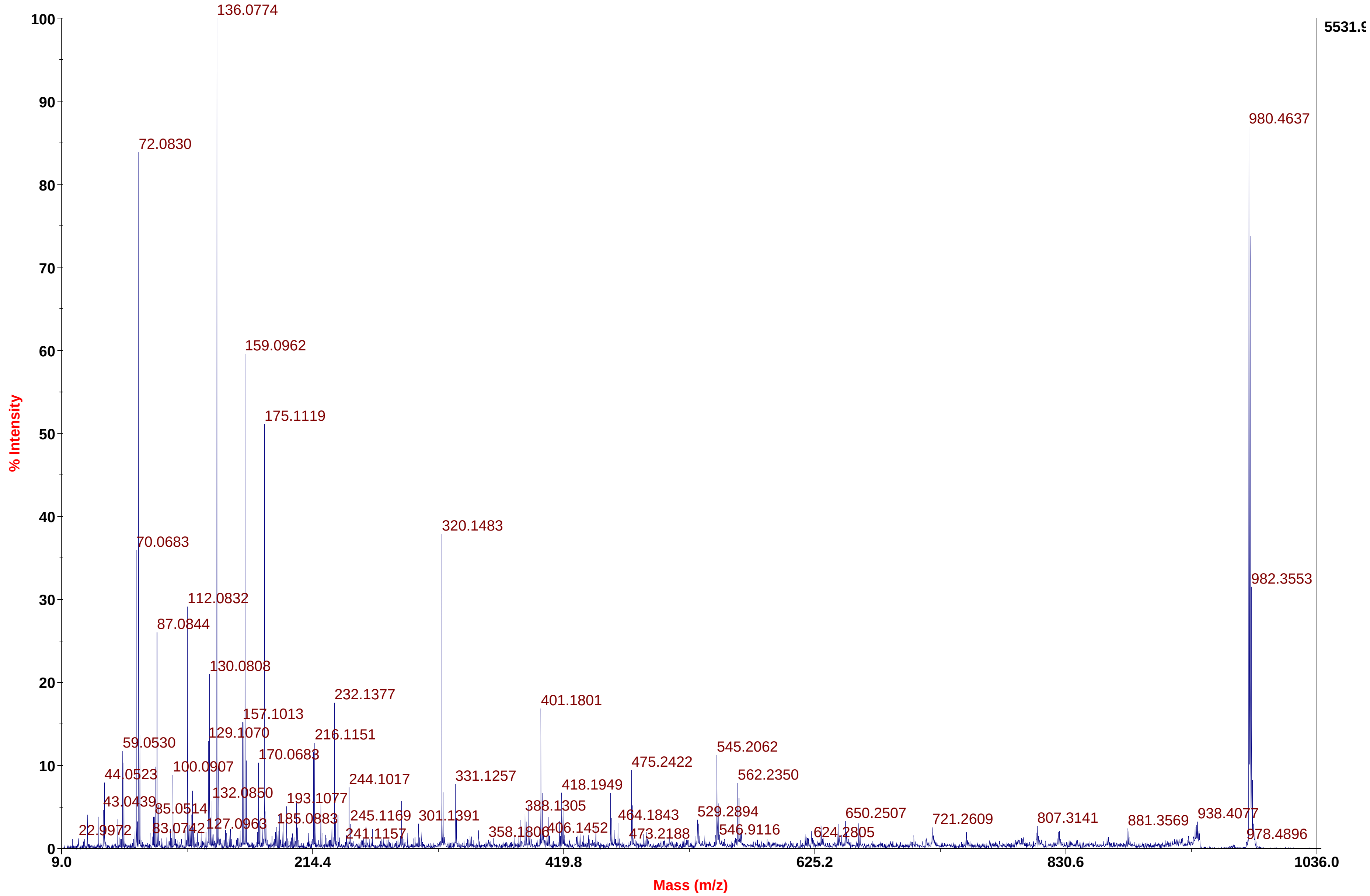

M6

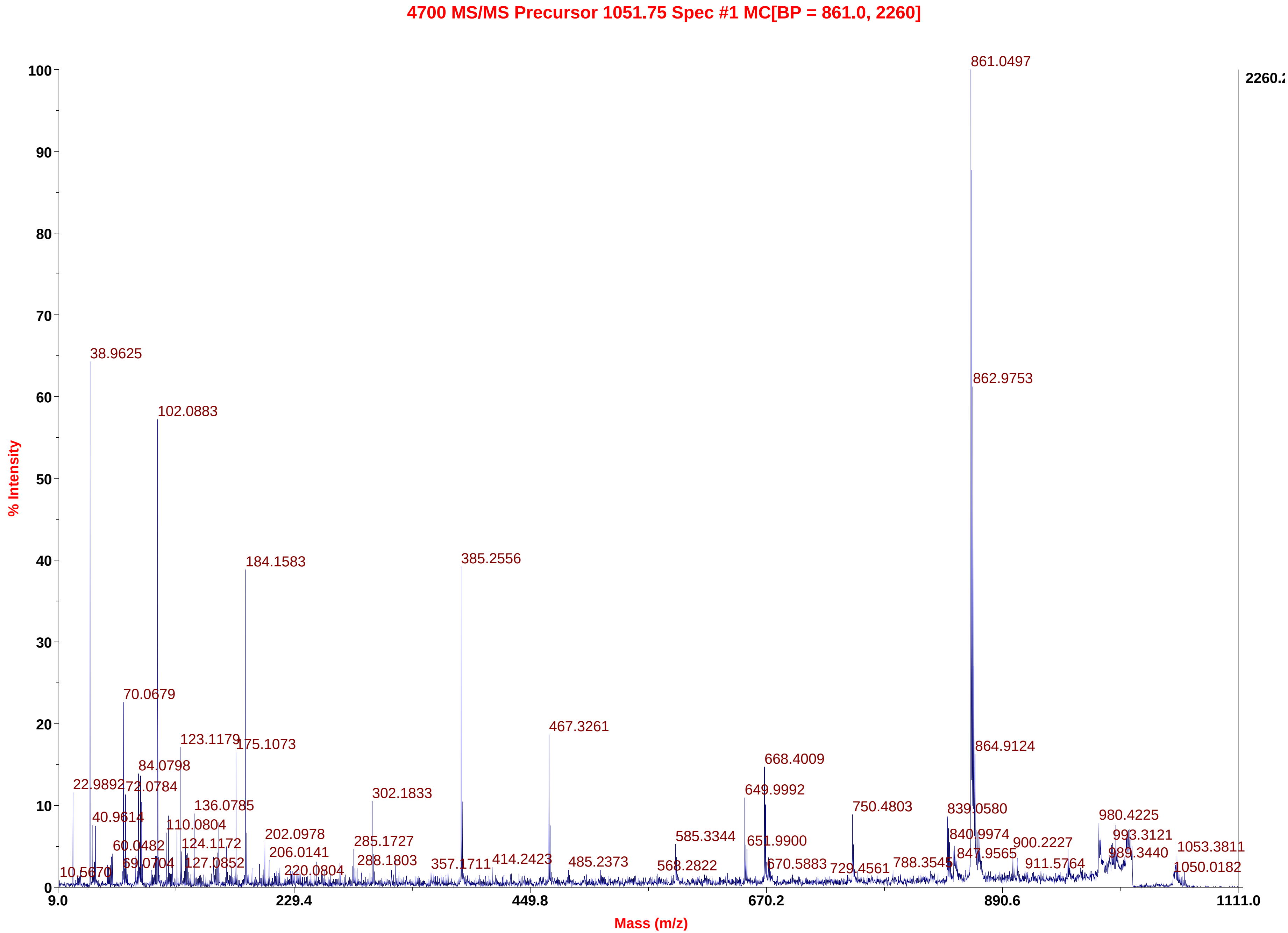
4700 MS/MS Precursor 1051.75 Spec #1 MC[BP = 861.0, 2260]
% Intensity
Mass (m/z)
861.0497
862.9753
864.9124
38.9625
102.0883
184.1583
385.2556
467.3261
70.0679
123.1179
175.1073
84.0798
22.9892
72.0784
40.9614
60.0482
69.0704
110.0804
124.1172
127.0852
136.0785
10.5670
202.0978
206.0141
220.0804
285.1727
288.1803
302.1833
357.1711
414.2423
485.2373
568.2822
585.3344
649.9992
651.9900
668.4009
670.5883
729.4561
750.4803
788.3545
839.0580
840.9974
847.9565
900.2227
911.5764
980.4225
993.3121
989.3440
1053.3811
1050.0182
2260.2

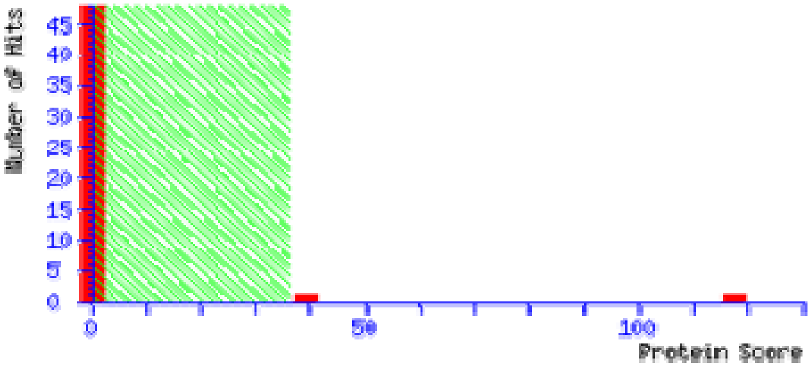

Peptide Summary Report

Format As | Peptide Summary | Help

Significance threshold p< 0.05 | Max. number of hits AUTO

Standard scoring ○ MudPIT scoring ◉ | Ions score or expect cut-off 0 | Show sub-sets 0

Show pop-ups ◉ Suppress pop-ups ○ | Sort unassigned Decreasing Score | Require bold red ☐

Select All | Select None | Search Selected | ☐ **Error tolerant**

1. HPMS **Mass**: 38821 **Score**: 117 **Matches**: 9(3) **Sequences**: 8(3) **emPAI**: 1.03
haptoglobin precursor - mouse
☐ Check to include this hit in error tolerant search

	Query	Observed	Mr(expt)	Mr(calc)	ppm	Miss	Score	Expect	Rank	Unique	Peptide
☑	1	920.5013	919.4940	919.4552	42.2	0	32	0.17	1	U	K.GSFPWQAK.M
☑	3	972.5767	971.5694	971.5287	41.8	0	24	0.94	1	U	K.NQLVEIEK.V
☑	4	980.5405	979.5333	979.4876	46.6	0	39	0.03	1	U	R.VGYVSGWGR.N
☑	10	1278.6979	1277.6906	1277.6326	45.4	0	37	0.058	1	U	K.YVMLPVADQDK.C
☑	11	1320.8011	1319.7938	1319.7337	45.6	0	23	0.66	1	U	K.DITPTLTLYVGK.N
☑	16	1635.8950	1634.8877	1634.7974	55.3	1	26	0.76	1	U	R.ATDLKDWVQETMAK.N
☑	18	1817.0505	1816.0432	1815.9375	58.2	1	52	0.0013	1	U	R.VMPICLPSKDYIAPGR.V + Car
☑	19	1833.0441	1832.0368	1831.9324	57.0	1	(22)	1.3	1	U	R.VMPICLPSKDYIAPGR.V + Car
☑	38	2127.2664	2126.2591	2126.1372	57.4	0	57	0.00022	1	U	R.HGLTTGATLISDQWLLTTAK.N

Protein View

Match to: **HPMS** Score: **117**
haptoglobin precursor - mouse
Found in search of ppw_F2_129990854705.txt

Nominal mass (M_r): **38821**; Calculated pI value: **6.08**
NCBI BLAST search of HPMS against nr
Unformatted sequence string for pasting into other applications

Taxonomy: Mus musculus

Variable modifications: Carbamidomethyl (C),Deamidated (NQ),Oxidation (M)
Cleavage by Trypsin: cuts C-term side of KR unless next residue is P
Sequence Coverage: **28%**

Matched peptides shown in **Bold Red**

1 MRALGAVVTL LLWGQLFAVE LGNDAMDFED DSCPKPPEIA NGYVEHLVRY
51 RCRQFYRLRA EGDGVYTLND EKQWVNTVAG HKLPECEAVC GKPKHPVDQV
101 QRIIGGSMDA K**GSFPWQAK**M ISR**HGLTTGA TLISDQWLLT TAK**NLFLNHS
151 ETASAK**DITP TLTLYVGKNQ LVEIEK**VVLH PNHSVVDIGL IKLKQRVLVT
201 ER**VMPICLPS KDYIAPGRVG YVSGWGR**NAN FRFTDRLK**YV MLPVADQDK**C
251 VVHYENSTVP EKKNLTSPVQ VQPILNEHTF CAGLTKYQED TCYGDAGSAF
301 AIHDMEEDTW YAAGILSFDK TCAVAEYGVY VR**ATDLKDWV QETMAK**N

Show predicted peptides also

Sort Peptides By ◉ Residue Number ○ Increasing Mass ○ Decreasing Mass

Start - End	Observed	Mr(expt)	Mr(calc)	ppm	Miss	Sequence	
112 - 119	920.5013	919.4940	919.4552	42	0	K.GSFPWQAK.M	(Ions score 32)
124 - 143	2127.2664	2126.2591	2126.1372	57	0	R.HGLTTGATLISDQWLLTTAK.N	(Ions score
157 - 168	1320.8011	1319.7938	1319.7337	46	0	K.DITPTLTLYVGK.N	(Ions score 23)
169 - 176	972.5767	971.5694	971.5287	42	0	K.NQLVEIEK.V	(Ions score 24)
203 - 218	1817.0505	1816.0432	1815.9375	58	1	R.VMPICLPSKDYIAPGR.V	Carbamidomethyl
203 - 218	1833.0441	1832.0368	1831.9324	57	1	R.VMPICLPSKDYIAPGR.V	Carbamidomethyl
219 - 227	980.5405	979.5333	979.4876	47	0	R.VGYVSGWGR.N	(Ions score 39)
239 - 249	1278.6979	1277.6906	1277.6326	45	0	K.YVMLPVADQDK.C	(Ions score 37)
333 - 346	1635.8950	1634.8877	1634.7974	55	1	R.ATDLKDWVQETMAK.N	(Ions score 26)

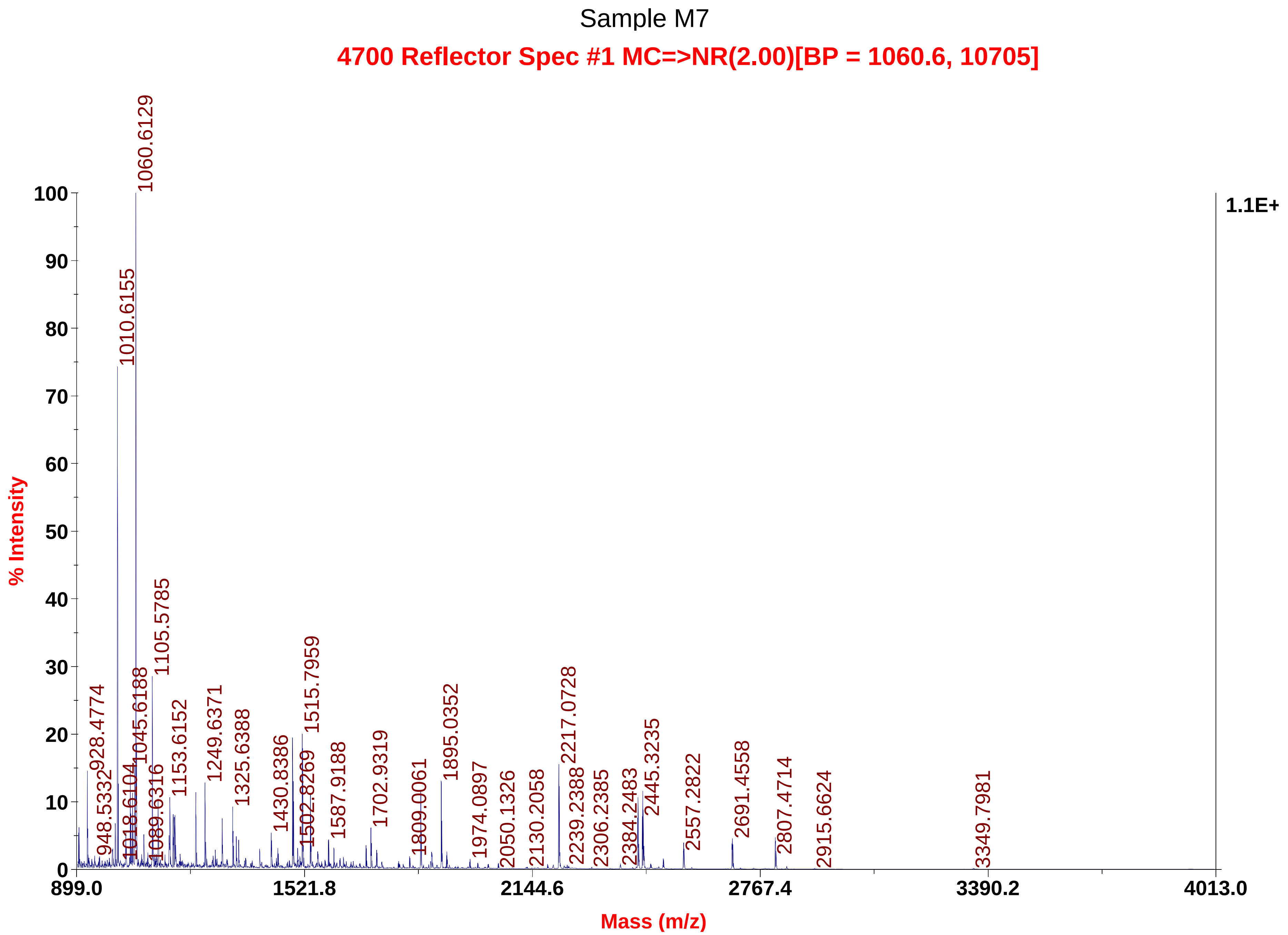
Sample M7
4700 Reflector Spec #1 MC=>NR(2.00)[BP = 1060.6, 10705]
% Intensity
Mass (m/z)
1.1E+
1060.6129
1010.6155
1105.5785
1515.7959
2217.0728
1045.6188
928.4774
1249.6371
1895.0352
1153.6152
1325.6388
2445.3235
1430.8386
1702.9319
2691.4558
1587.9188
2557.2822
1502.8269
1809.0061
2807.4714
1018.6104
1089.6316
948.5332
1974.0897
2050.1326
2130.2058
2239.2388
2306.2385
2384.2483
2915.6624
3349.7981
899.0
1521.8
2144.6
2767.4
3390.2
4013.0
0
10
20
30
40
50
60
70
80
90
100

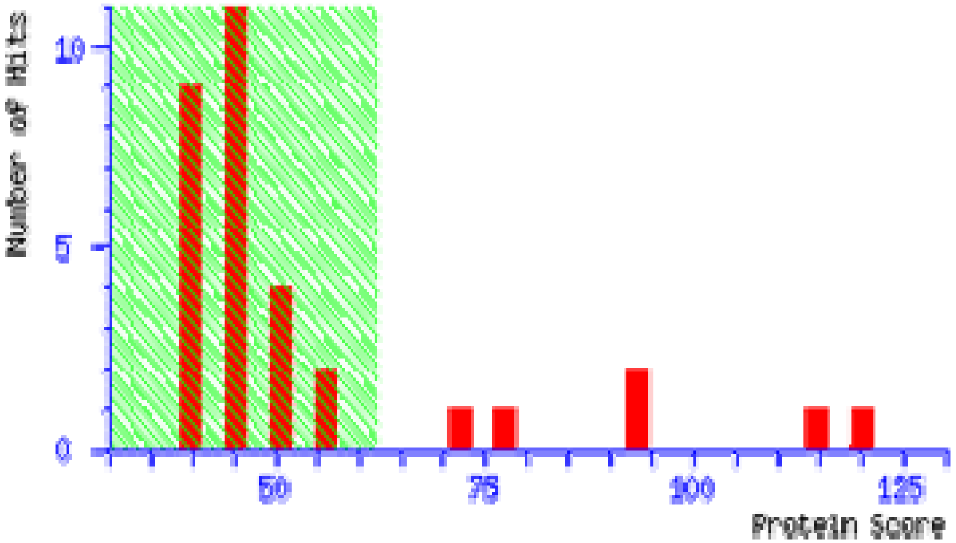

Concise Protein Summary Report

Format As | Concise Protein Summary | Help

Significance threshold p< 0.05 Max. number of hits AUTO

Re-Search All | Search Unmatched

1. Q32P18_MOUSE **Mass:** 223203 **Score:** 120 **Expect:** 9.2e-08 **Matches:** 95
   Myosin, heavy polypeptide 1, skeletal muscle, adult.- Mus musculus (Mouse).
   Q9ESW6_MOUSE **Mass:** 54545 **Score:** 40 **Expect:** 9.2 **Matches:** 31
   Myosin heavy chain IIX (Fragment).- Mus musculus (Mouse).

Protein View

Match to: Q32P18_MOUSE Score: 120 Expect: 9.2e-08
Myosin, heavy polypeptide 1, skeletal muscle, adult.- Mus musculus (Mous
Found in search of pmf_G2_1299084890б.txt

Nominal mass (M_r): 223203; Calculated pI value: 5.60
NCBI BLAST search of Q32P18_MOUSE against nr
Unformatted sequence string for pasting into other applications

Taxonomy: Mus musculus
Links to retrieve other entries containing this sequence from NCBI Entre
(no taxonomy information for this entry)
(no taxonomy information for this entry)
(no taxonomy information for this entry)

Variable modifications: Carbamidomethyl (C),Deamidated (NQ),Oxidation (M
Cleavage by Trypsin: cuts C-term side of KR unless next residue is P
Number of mass values searched: 167
Number of mass values matched: 95
Sequence Coverage: 48%

Matched peptides shown in **Bold Red**

1 **MSSDAEMAVF** **GEAAPYLRK**S EKERIEAQNK PFDAKSSVFV VDAKESFVK**A**
51 **TVQSREGGK**V TAKTEGGTTV TVKDDQVYPM NPPKYDKIED MAMMTHLHEP
101 AVLYNLKERY AAWMIYTYSG LFCVTVNPYK WLPVYNAEVV AAYRGKKRQE
151 APPHIFSISD NAYQFMLTDR ENQSILITGE SGAGKTVNTK RVIQYFATIA
201 VTGEKKKEEA TSGK**MQGTLE** **DQIISANPLL** **EAFGNAK**TVR NDNSSRFGKF
251 IRIHFGTTGK LASADIETYL LEKSR**VTFQL** **KAERSYHIFY** **QIMSNK**KPDL
301 IEMLLITTNP YDYAFVSQGE ITVPSIDDQE ELMATDSAID ILGFTSDERV
351 SIYKLTGAVM HYGNMKFKQK QREEQAEPDG TEVADK**AAYL** **QNLNSADLLK**
401 **ALCYPR**VK**VG** **NEYVTKGQTV** **QQVYNSVGAL** **AK**AVYEK**MFL** **WMVTR**INQQL
451 DTKQPRQYFI GVLDIAGFEI FDFNSLEQLC INFTNEK**LQQ** **FFNHHMFVLE**
501 **QEEYK**KEGIE WEFIDFGMDL AACIELIEKP MGIFSILEEE CMFPKATDTS
551 FKNK**LYEQHL** **GKSNNFQKPK** **PAKGK**VEAHF SLVHYAGTVD YNIAGWLDK**N**
601 **KDPLNETVVG** **LYQKSSMK**TL AYLFSGAAAA AEAESGGGGG KKGAKKKGSS
651 FQTVSALFRE NLNKLMTNLR STHPHFVRCI IPNETK**TPGA** **MEHELVLHQL**
701 **RCNGVLEGIR** ICRKGFPSR**I** **LYADFKQR**YK **VLNASAIPEG** **QFIDSKK**ASE
751 K**LLGSIDIDH** **TQYK**FGHTKV FFKAGLLGLL EEMRDDK**LAQ** **LITRTQAMCR**
801 **GYLARVEYQK** **MVER**RESIFC IQYNVR**AFMN** **VKHWPWMK**LY FKIKPLLKSA
851 ETEKEMANMK EEFEKAKENL AKAEAKRK**EL** **EEKMVALMQE** **KNDLQLQVQS**
901 **EADSLADAEE** **RCDQLIKTK**I QLEAKIKEVT ERAEDEEEIN AELTAKKRKL
951 EDECSELK**KD** **IDDLELTLAK** **VEK**EK**HATEN** **KVKNLTEEMA** **GLDETIAKLT**
1001 **K**EKK**ALQEAH** **QQTLDDLQAE** **EDKVNTLTK**A KIK**LEQQVDD** **LEGSLEQEKK**
1051 **IRMDLER**AKR KLEGDLKLAQ ESTMDVENDK QQLDEKLKK**K** **EFEMSNLQSK**
1101 **IEDEQALGMQ** **LQKK**IKELQA R**IEELEEEIE** **AER**ASRAKAE KQRSDLSREL
1151 EEISER**LEEA** **GGATSAQIEM** **NKK**REAEFQK MRR**DLEEATL** **QHEATAATLR**
1201 K**KHADSVAEL** **GEQIDNLQR**V KQKLEKEKSE MKMEIDDLAS NMEVISKSKG
1251 NLEK**MCRTLE** **DQVSELKTKE** **EEQQRLINEL** **TAQRGRLQTE** **SGEYSRQLDE**
1301 **KDSLVSQLSR** **GKQAFTQQIE** **ELKRQLEEEI** KAK**SALAHAL** **QSSRHDCDLL**
1351 **REQYEEEQEA** **KAELQRAMSK** **ANSEVAQWRT** **KYETDAIQRT** **EELEEAKK**KL
1401 AQR**LQDAEEH** **VEAVNAKCAS** **LEK**TKQR**LQN** **EVEDLMIDVE** **RTNAACAALD**
1451 **KK**QRNFDKIL AEWK**QKYEET** **HAELEASQKE** **SRSLSTELFK** **IK**NAYEESLD
1501 HLETLKRENK **NLQQEISDLT** **EQIAEGGK**RI HELEKIK**KQI** **EQEK**SELQAA
1551 LEEAEASLEH EEGKILR**IQL** **ELNQVK**SEID RK**IAEKDEEI** **DQLK**RNHIRV
1601 VESMQSTLDA EIRSRNDAIR LKKK**MEGDLN** **EMEIQLNHSN** **RMAAEALR**NY
1651 R**NTQGILKDT** **QLHLDDALRG** **QEDLKEQLAM** **VERRANLLQA** **EIEELRATLE**
1701 **QTER**SR**KIAE** **QELLDASERV** **QLLHTQNTSL** **INTKKKLETD** **ISQIQGEMED**
1751 **IVQEAR**NAEE KAKK**AITDAA** **MMAEELKK**EQ DTSAHLERMK **KNLEQTVK**DL
1801 QHRLDEAEQL ALKGGKKQIQ KLEAR**VRELE** **GEVENEQK**RN VEAIKGLRKH
1851 ERRVKELTYQ TEEDRKNVLR LQDLVDKLQS KVKAYKRQAE EAEEQSNVNL
1901 AKFR**KIQHEL** **EEAEER**ADIA ESQVNKLRVK SREVHTKIIS EE

M7

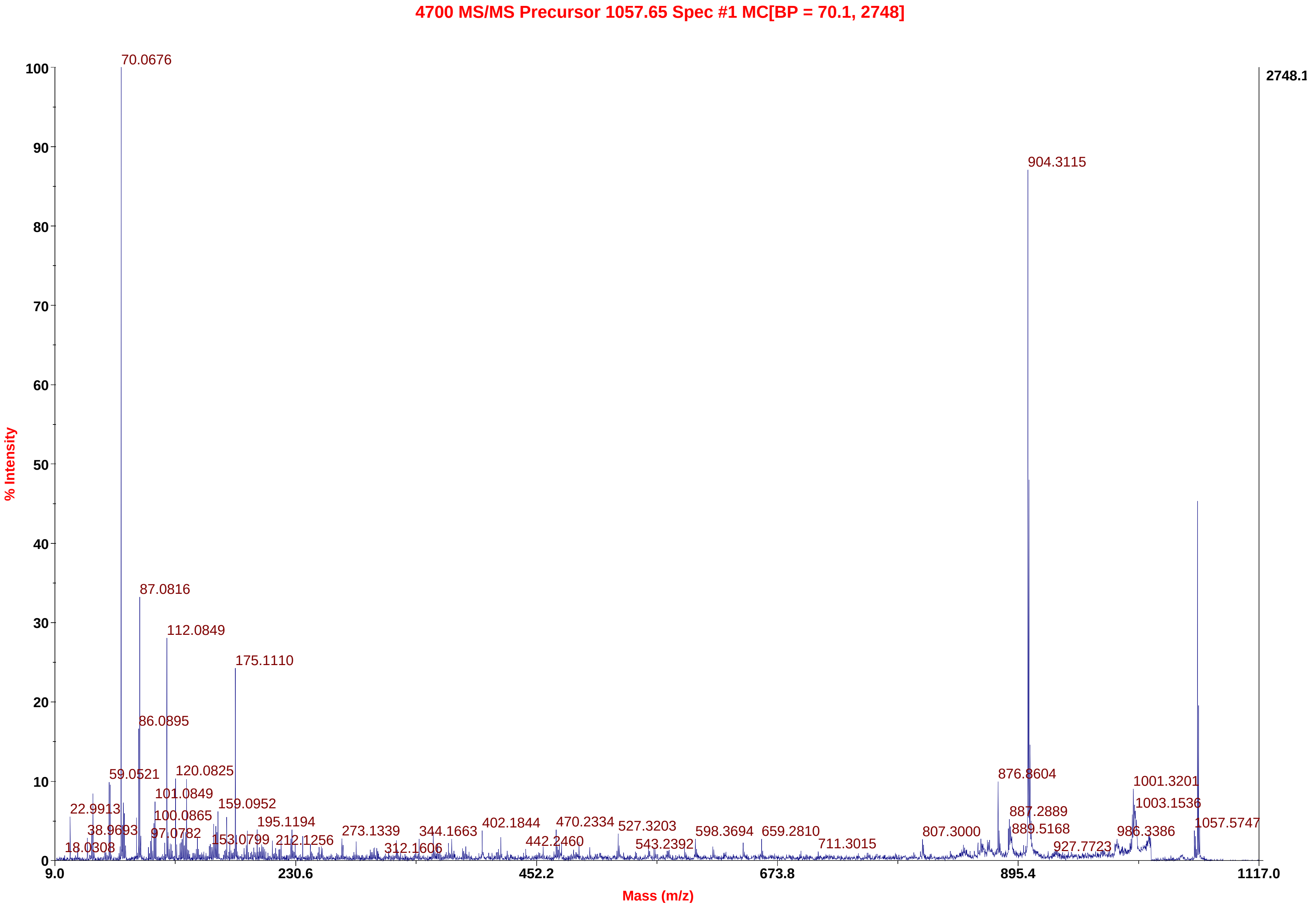
4700 MS/MS Precursor 1057.65 Spec #1 MC[BP = 70.1, 2748]
% Intensity
Mass (m/z)
2748.1
70.0676
904.3115
87.0816
112.0849
175.1110
86.0895
59.0521
120.0825
101.0849
22.9913
159.0952
100.0865
195.1194
38.9693
97.0782
18.0308
153.0799
212.1256
273.1339
312.1606
344.1663
402.1844
442.2460
470.2334
527.3203
543.2392
598.3694
659.2810
711.3015
807.3000
876.8604
887.2889
889.5168
927.7723
1001.3201
1003.1536
986.3386
1057.5747
9.0
230.6
452.2
673.8
895.4
1117.0

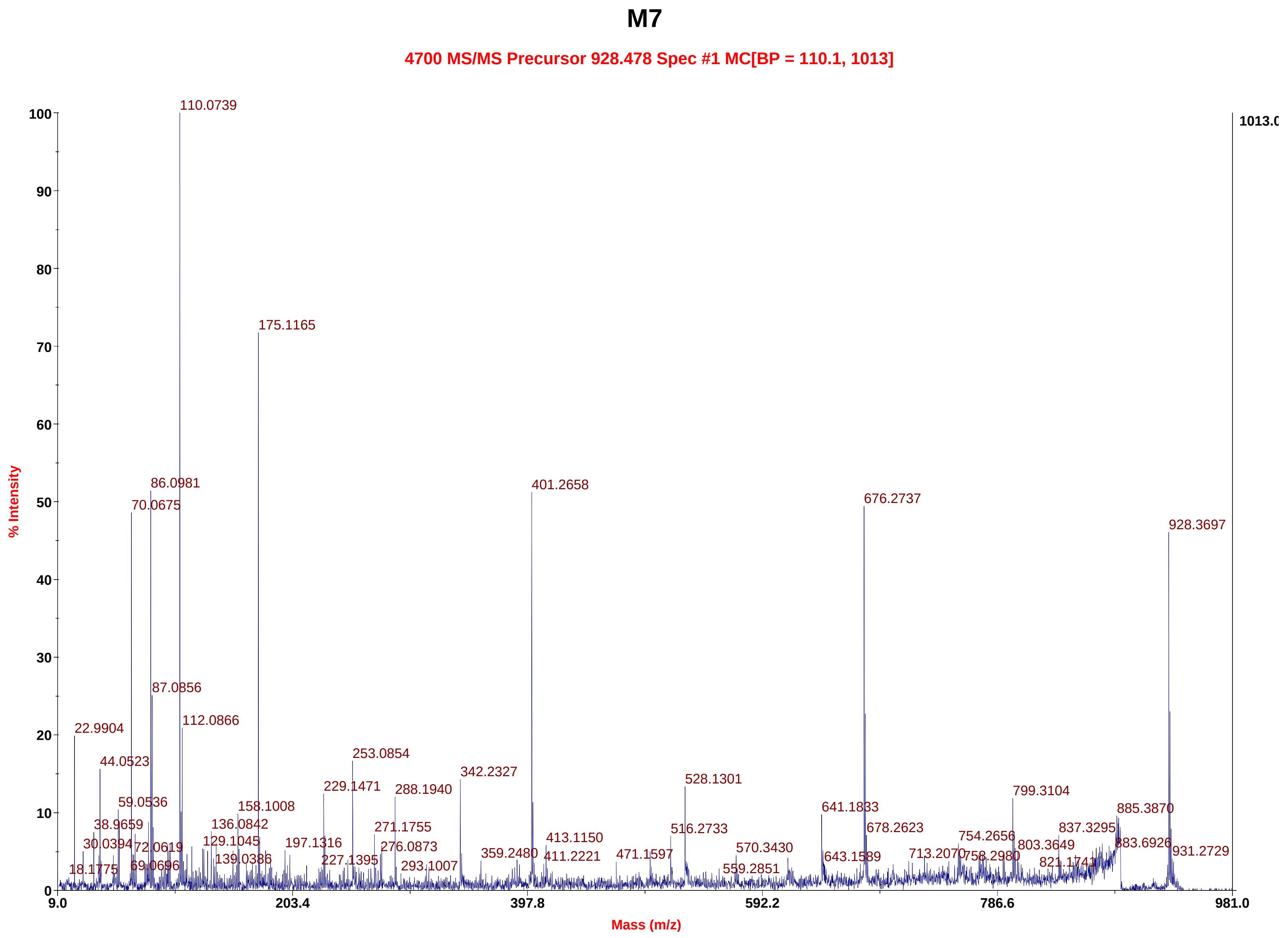
M7
4700 MS/MS Precursor 928.478 Spec #1 MC[BP = 110.1, 1013]
% Intensity
Mass (m/z)
110.0739
175.1165
86.0981
70.0675
401.2658
676.2737
928.3697
87.0856
112.0866
22.9904
44.0523
253.0854
342.2327
528.1301
229.1471
288.1940
799.3104
59.0536
158.1008
641.1833
885.3870
38.9659
136.0842
271.1755
678.2623
516.2733
837.3295
754.2656
413.1150
129.1045
30.0394
72.0619
197.1316
276.0873
570.3430
803.3649
883.6926
931.2729
359.2480
713.2070
758.2980
411.2221
471.1597
643.1589
139.0386
227.1395
821.1741
69.0696
293.1007
559.2851
18.1775
1013.0
9.0
203.4
397.8
592.2
786.6
981.0
0
10
20
30
40
50
60
70
80
90
100

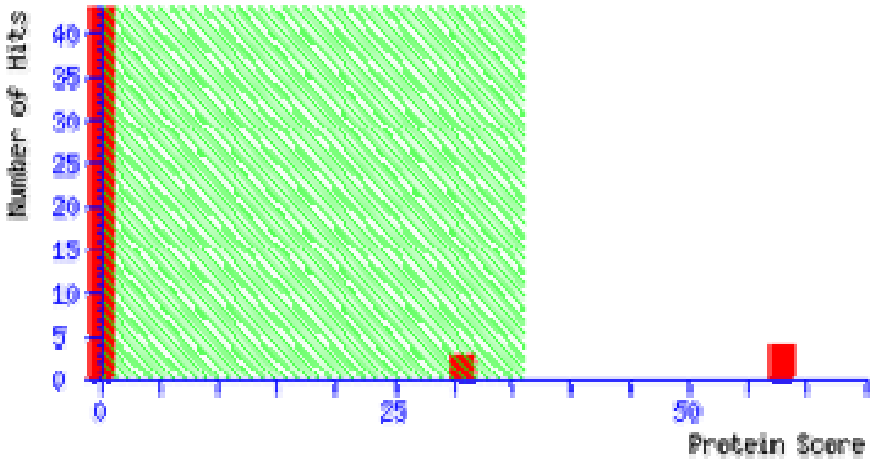

Peptide Summary Report

Format As | Peptide Summary | Help

Significance threshold p< 0.05 | Max. number of hits AUTO

Standard scoring | MudPIT scoring | Ions score or expect cut-off 0 | Show sub-sets 0

Show pop-ups | Suppress pop-ups | Sort unassigned Decreasing Score | Require bold red

Select All | Select None | Search Selected | **Error tolerant**

1. Q9JHR4_MOUSE **Mass:** 60994 **Score:** 58 **Matches:** 1(1) **Sequences:** 1(1) **emPAI:** 0.10

Myosin heavy chain IIB (Fragment).- Mus musculus (Mouse).

Check to include this hit in error tolerant search

	Query	Observed	Mr(expt)	Mr(calc)	ppm	Miss	Score	Expect	Rank	Unique	Peptide
☑	15	1702.9291	1701.9218	1701.8243	57.3	0	58	0.00046	1		R.LQNEVEDLMIDVER.S

Protein View

Match to: **Q9JHR4_MOUSE** Score: **58**
Myosin heavy chain IIB (Fragment).- Mus musculus (Mouse).
Found in search of ppw_G2_12999085480б.txt

Nominal mass (M_r): **60994**; Calculated pI value: **5.38**
NCBI BLAST search of Q9JHR4_MOUSE against nr
Unformatted sequence string for pasting into other applications

Taxonomy: Mus musculus
Links to retrieve other entries containing this sequence from NCBI Entrez:
(no taxonomy information for this entry)

Variable modifications: Carbamidomethyl (C),Deamidated (NQ),Oxidation (M)
Cleavage by Trypsin: cuts C-term side of KR unless next residue is P
Sequence Coverage: **2%**

Matched peptides shown in **Bold Red**

```
  1 SLEKTKQRLQ NEVEDLMIDV ERSNAACAAL DKKQRNFDKV LAEWKQKYEE
 51 TQAELEASQK ESRSLSTELF KVKNAYEESL DQLETLKREN KNLQQEISDL
101 TEQIAEGGKH IHELEKIKKQ IDQEKSELQA SLEEAEASLE HEEGKILRIQ
151 LELNQVKSEI DRKIAEKDEE IDQLKRNHLR VVESMQSTLD AEIRSRNDAL
201 RIKKKMEGDL NEMEIQLNHA NRQAAEAIRN LRNTQGMLKD TQLHLDDALR
251 GQDDLKEQLA MVERRANLMQ AEIEELRASL EQTERSRRVA EQELLDASER
301 VQLLHTQNTS LINTKKKLET DISQIQGEME DIVQEARNAE EKAKKAITDA
351 AMMAEELKKE QDTSAHLERM KKNMEQTVKD LQHRLDEAEQ LALKGGKKQI
401 QKLEARVREL ENEVENEQKR NIEAVKGLRK HERRVKELTY QTEEDRKNVL
451 RLQDLVDKLQ TKVKAYKRQA EEAEEQSNVN LAKFRKIQHE LEEAEERADI
501 AESQVNKLRV KSREVHTKVI SEE
```

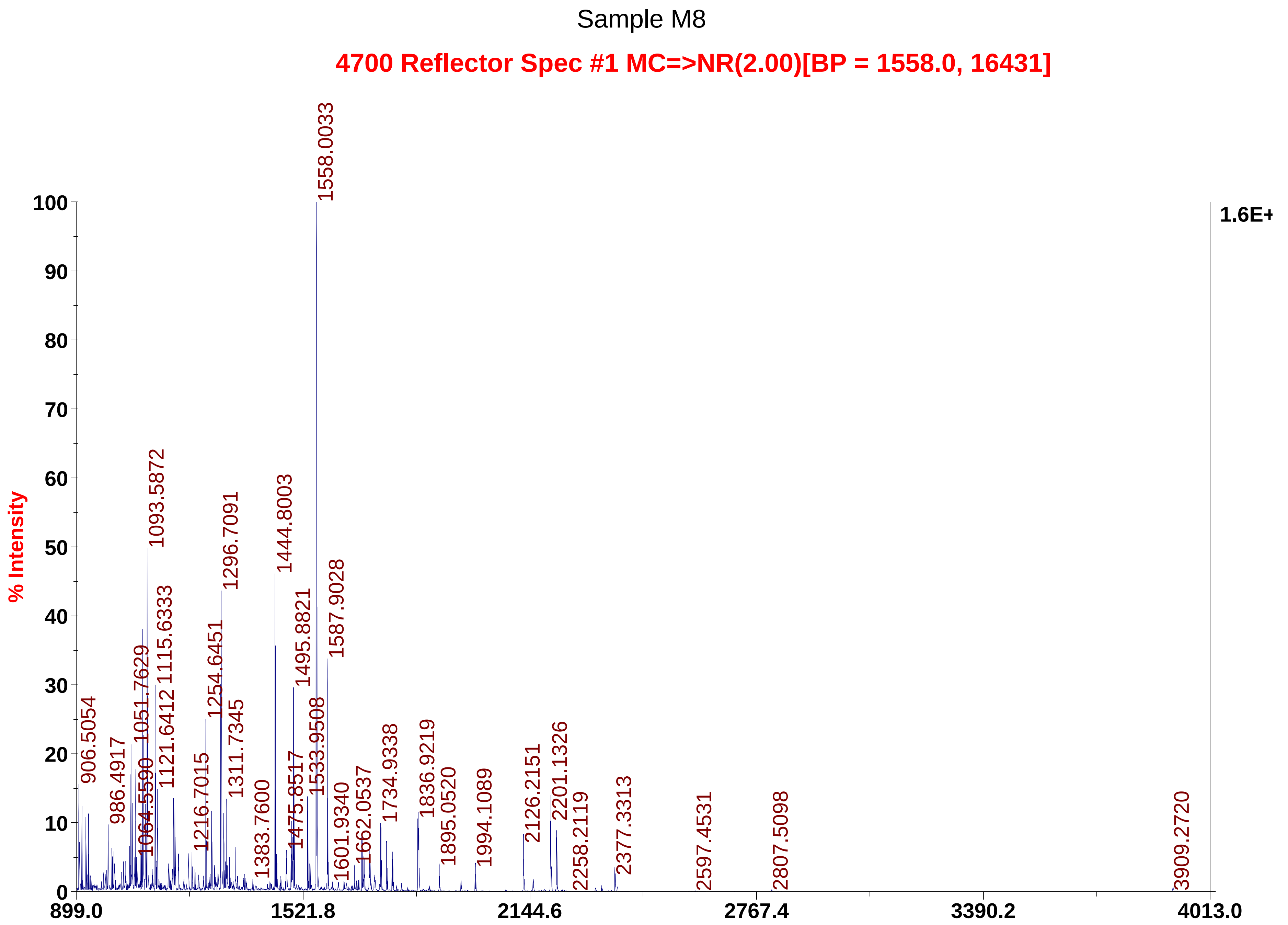

Sample M8
4700 Reflector Spec #1 MC=>NR(2.00)[BP = 1558.0, 16431]
% Intensity
Mass (m/z)
1.6E+
100
90
80
70
60
50
40
30
20
10
0
899.0
1521.8
2144.6
2767.4
3390.2
4013.0
906.5054
986.4917
1051.7629
1064.5590
1093.5872
1115.6333
1121.6412
1216.7015
1254.6451
1296.7091
1311.7345
1383.7600
1444.8003
1475.8517
1495.8821
1533.9508
1558.0033
1587.9028
1601.9340
1662.0537
1734.9338
1836.9219
1895.0520
1994.1089
2126.2151
2201.1326
2258.2119
2377.3313
2597.4531
2807.5098
3909.2720

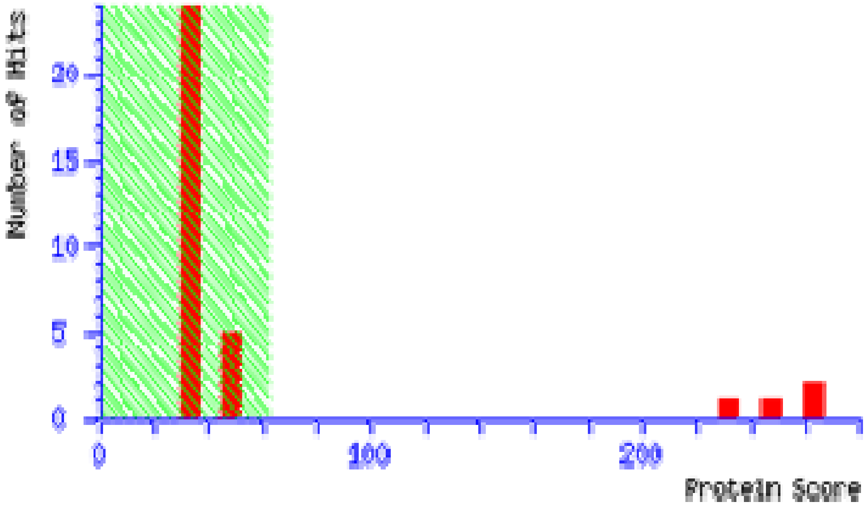

Concise Protein Summary Report

Format As | Concise Protein Summary | Help

Significance threshold p< 0.05 Max. number of hits AUTO

Re-Search All | Search Unmatched

1. CAA69019 **Mass**: 51533 **Score**: 262 **Expect**: 5.8e-22 **Matches**: 65
   MMVIMENT NID: - Mus musculus
   Q3UD36_MOUSE **Mass**: 47148 **Score**: 213 **Expect**: 4.6e-17 **Matches**: 61
   Bone marrow macrophage cDNA, RIKEN full-length enriched library, clone:I830001B16 product:vimentin,
   BAC28181 **Mass**: 17978 **Score**: 69 **Expect**: 0.011 **Matches**: 25
   AK033175 NID: - Mus musculus

Protein View

```
Match to: CAA69019 Score: 262 Expect: 5.8e-22
MMVIMENT NID:  - Mus musculus
Found in search of pmf_H2_129990848907.txt
```

Nominal mass (M_r): **51533**; Calculated pI value: **4.96**
NCBI BLAST search of CAA69019 against nr
Unformatted sequence string for pasting into other applications

Taxonomy: Mus musculus

Variable modifications: Carbamidomethyl (C),Deamidated (NQ),Oxidation (M)
Cleavage by Trypsin: cuts C-term side of KR unless next residue is P
Number of mass values searched: **162**
Number of mass values matched: **65**
Sequence Coverage: **89%**

Matched peptides shown in **Bold Red**

```
  1 SSRPSSNRSY VTTSTRTYSL GSALRPSTSR SLYSSSPGGA YVTRSSAVRL
 51 RSSVPGVRLL QDSVDFSLAD AINTEFKNTR TNEKVELQEL NDRFANYIDK
101 VRFLEQQNKI LLAELEQLKG QGKSRLGDLY EEEMRELRRQ VDQLTNDKAR
151 VEVERDNLAE DIMRLREKLQ EEMLQREEAE STLQSFRQDV DNASLARLDL
201 ERKVESLQEE IAFLKKLHDE EIQELQAQIQ EQHVQIDVDV SKPDLTAALR
251 DVRQQYESVA AKNLQEAEEW YKSKFADLSE AANRNNDALR QAKQESNEYR
301 RQVQSLTCEV DALKGTNESL ERQMREMEEN FALEAANYQD TIGRLQDEIQ
351 NMKEEMARHL REYQDLLNVK MALDIEIATY RKLLEGEESR ISLPLPTFSS
401 LNLRETNLES LPLVDTHSKR TLLIKTVETR DGQVINETSQ HHDDLE
```

Show predicted peptides also

Sort Peptides By ◉ Residue Number ○ Increasing Mass ○ Decreasing Mass

Start - End	Observed	Mr(expt)	Mr(calc)	ppm	Miss	Sequence
9 - 16	914.5060	913.4988	913.4505	53	0	R.SYVTTSTR.T
17 - 30	1495.8798	1494.8725	1494.7790	63	0	R.TYSLGSALRPSTSR.S
31 - 44	1444.7979	1443.7906	1443.6994	63	0	R.SLYSSSPGGAYVTR.S
59 - 77	2126.2109	2125.2036	2125.0579	69	0	R.LLQDSVDFSLADAINTEFK.N
81 - 93	1587.9003	1586.8930	1586.7900	65	1	R.TNEKVELQELNDR.F
85 - 93	1115.6313	1114.6240	1114.5618	56	0	K.VELQELNDR.F
94 - 102	1125.6686	1124.6613	1124.5978	56	1	R.FANYIDKVR.F
101 - 109	1162.6250	1161.6177	1161.6142	3	1	K.VRFLEQQNK.I Deamidated (NQ)
103 - 109	906.5054	905.4981	905.4607	41	0	R.FLEQQNK.I
103 - 109	908.4515	907.4443	907.4287	17	0	R.FLEQQNK.I 2 Deamidated (NQ)
110 - 119	1169.7764	1168.7691	1168.7067	53	0	K.ILLAELEQLK.G
110 - 123	1540.0065	1538.9992	1538.9032	62	1	K.ILLAELEQLKGQGK.S

M8

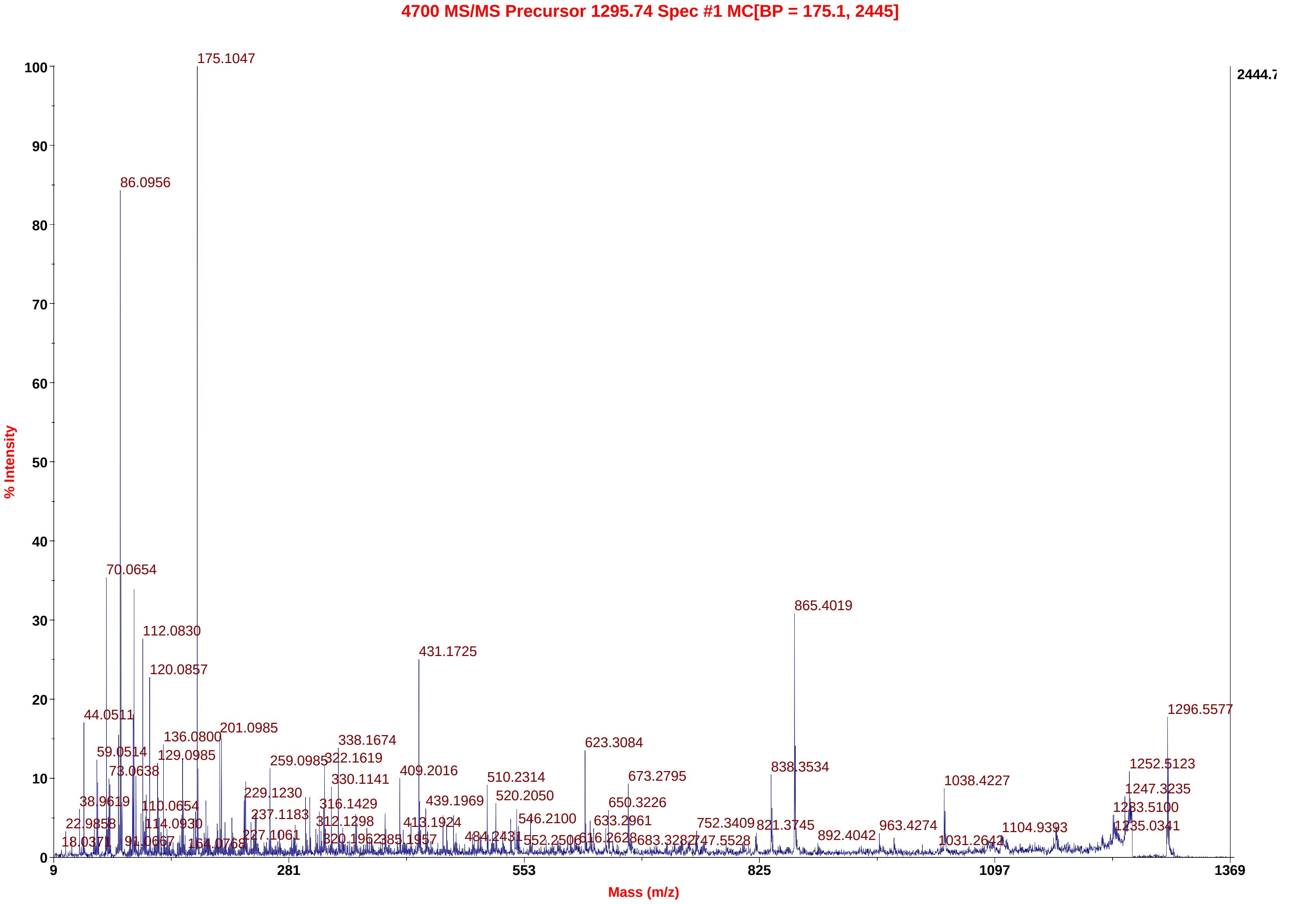

4700 MS/MS Precursor 1295.74 Spec #1 MC[BP = 175.1, 2445]
% Intensity
Mass (m/z)
2444.7
175.1047
86.0956
70.0654
112.0830
120.0857
44.0511
59.0514
73.0638
136.0800
129.0985
201.0985
38.9619
22.9858
18.0371
110.0654
114.0930
91.0667
164.0768
229.1230
237.1183
227.1061
259.0985
338.1674
322.1619
330.1141
316.1429
312.1298
320.1962
385.1957
409.2016
431.1725
439.1969
413.1924
484.2431
510.2314
520.2050
546.2100
552.2506
623.3084
673.2795
650.3226
633.2961
616.2628
683.3282
747.5528
752.3409
821.3745
838.3534
865.4019
892.4042
963.4274
1038.4227
1031.2642
1104.9393
1252.5123
1247.3235
1233.5100
1235.0341
1296.5577

M8

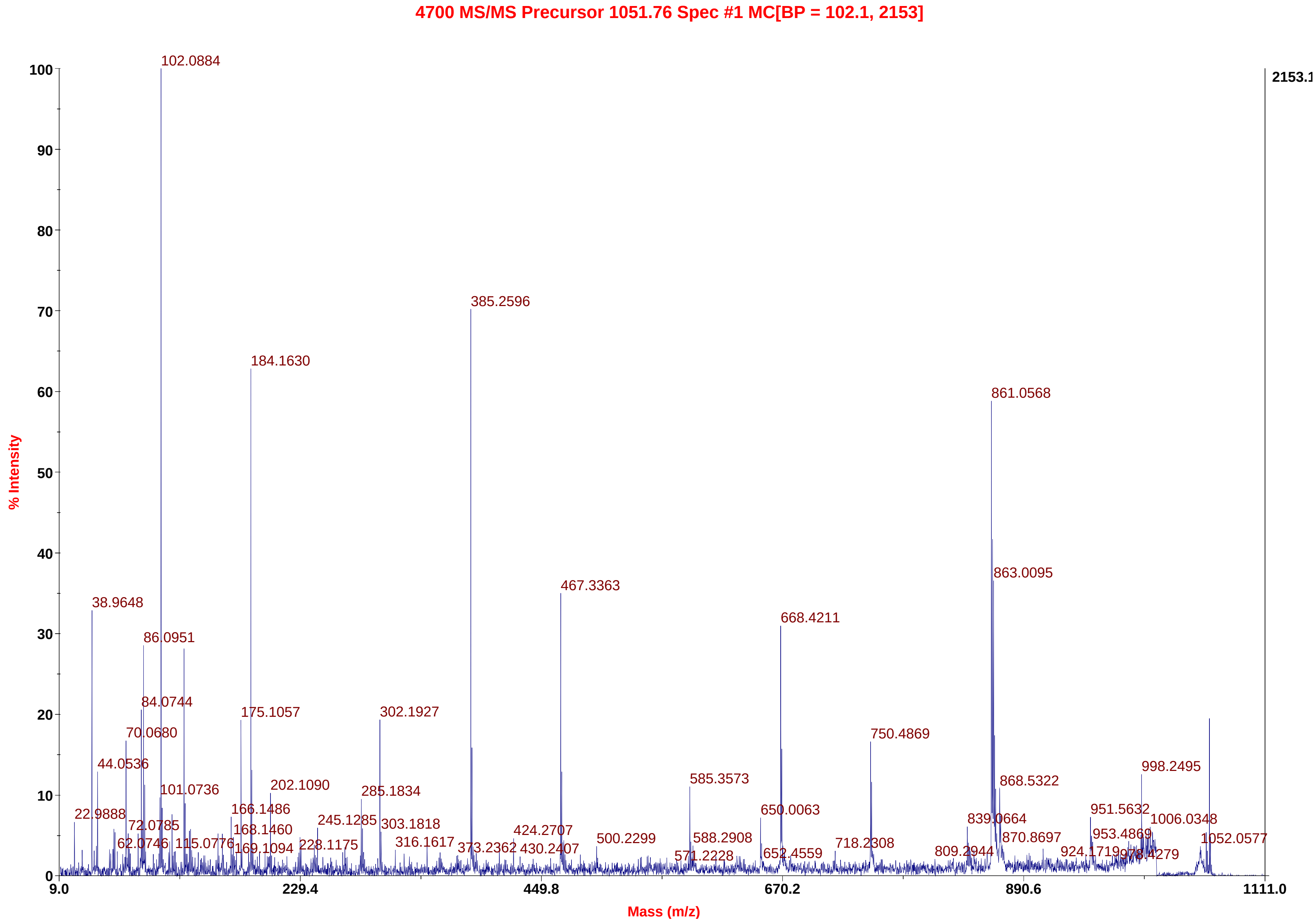
4700 MS/MS Precursor 1051.76 Spec #1 MC[BP = 102.1, 2153]
% Intensity
Mass (m/z)
2153.1
102.0884
385.2596
184.1630
861.0568
863.0095
467.3363
38.9648
668.4211
86.0951
84.0744
175.1057
302.1927
70.0680
750.4869
44.0536
998.2495
585.3573
868.5322
101.0736
202.1090
285.1834
22.9888
166.1486
650.0063
951.5632
839.0664
1006.0348
72.0785
245.1285
303.1818
168.1460
424.2707
953.4869
588.2908
500.2299
870.8697
1052.0577
62.0746
115.0776
316.1617
718.2308
169.1094
228.1175
373.2362
430.2407
809.2944
924.1719
978.4279
571.2228
652.4559
9.0
229.4
449.8
670.2
890.6
1111.0

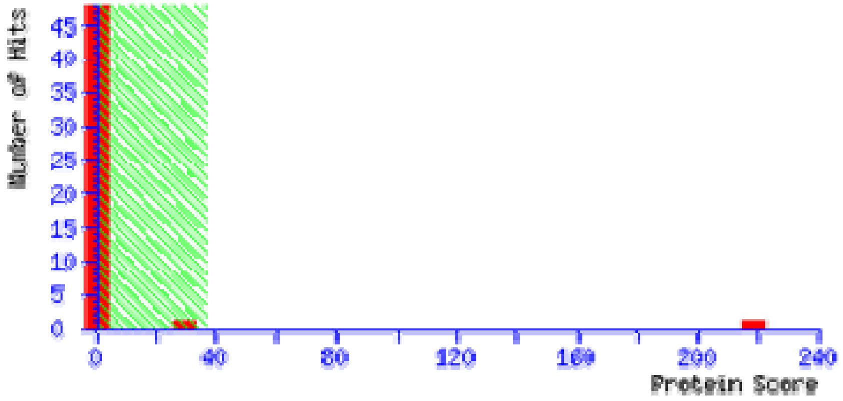

Peptide Summary Report

Format As | Peptide Summary | Help

Significance threshold p< 0.05 | Max. number of hits AUTO

Standard scoring ○ MudPIT scoring ● | Ions score or expect cut-off 0 | Show sub-sets 0

Show pop-ups ● Suppress pop-ups ○ | Sort unassigned Decreasing Score | Require bold red ☐

Select All | Select None | Search Selected | ☐ **Error tolerant**

1. VIME_MOUSE **Mass:** 53524 **Score:** 219 **Matches:** 18(9) **Sequences:** 15(8) **emPAI:** 2.82
Vimentin.- Mus musculus (Mouse).
☐ Check to include this hit in error tolerant search

	Query	Observed	Mr(expt)	Mr(calc)	ppm	Miss	Score	Expect	Rank	Unique	Peptide
☑	1	914.5060	913.4988	913.4505	52.8	0	23	1.2	1	U	R.SYVTTSTR.T
☑	3	1081.5625	1080.5552	1080.4948	55.9	1	17	4.9	1	U	K.QESNEYRR.Q
☑	4	1093.5869	1092.5796	1092.5200	54.6	0	48	0.005	1	U	K.FADLSEAANR.N
☑	5	1115.6313	1114.6240	1114.5618	55.8	0	31	0.2	1	U	K.VELQELNDR.F
☑	7	1254.6449	1253.6376	1253.5598	62.1	0	38	0.044	1	U	R.LGDLYEEEMR.E
☑	8	1270.6376	1269.6303	1269.5547	59.6	0	(12)	21	1	U	R.LGDLYEEEMR.E + Oxidation
☑	9	1295.7449	1294.7376	1294.6591	60.7	0	31	0.16	1	U	K.MALDIEIATYR.K

Protein View

Match to: **VIME_MOUSE** Score: **219**
Vimentin.- Mus musculus (Mouse).
Found in search of ppw_H2_129990854907.txt

Nominal mass (M_r): **53524**; Calculated pI value: **5.06**
NCBI BLAST search of VIME_MOUSE against nr
Unformatted sequence string for pasting into other applications

Taxonomy: Mus musculus

Variable modifications: Carbamidomethyl (C),Deamidated (NQ),Oxidation (M)
Cleavage by Trypsin: cuts C-term side of KR unless next residue is P
Sequence Coverage: 38%

Matched peptides shown in **Bold Red**

1 STRSVSSSSY RRMFGGSGTS SRPSSNR**SYV** **TTSTRTYSLG** **SALRPSTSRS**

51 **LYSSSPGGAY** **VTR**SSAVRLR SSVPGVRLLQ DSVDFSLADA INTEFKNTR**T**

101 **NEKVELQELN** **DR**FANYIDKV RFLEQQNKIL LAELEQLKGQ GKSR**LGDLYE**

151 **EEMR**ELRRQV DQLTNDKARV EVERDNLAED IMRLREKLQE EMLQREEAES

201 TLQSFRQDVD NASLARLDLE R**KVESLQEEI** **AFLK**KLHDEE IQELQAQIQE

251 QHVQIDVDVS KPDLTAALRD VRQQYESVAA KNLQEAEEWY KSK**FADLSEA**

301 **ANR**NNDALRQ AK**QESNEYRR** QVQSLTCEVD ALKGTNESLE RQMR**EMEENF**

351 **ALEAANYQDT** **IGRLQDEIQN** **MKEEMAR**HLR EYQDLLNVK**M** **ALDIEIATYR**

401 KLLEGEESR**I** **SLPLPTFSSL** **NLRETNLESL** **PLVDTHSK**RT LLIKTVETR**D**

451 **GQVINETSQH** **HDDLE**

Show predicted peptides also

Sort Peptides By ◉ Residue Number ○ Increasing Mass ○ Decreasing Mass

Start - End	Observed	Mr(expt)	Mr(calc)	ppm	Miss	Sequence
28 - 35	914.5060	913.4988	913.4505	53	0	R.SYVTTSTR.T (Ions score 23)
36 - 49	1495.8798	1494.8725	1494.7790	63	0	R.TYSLGSALRPSTSR.S (Ions score 9)
50 - 63	1444.7979	1443.7906	1443.6994	63	0	R.SLYSSSPGGAYVTR.S (Ions score 24)
100 - 112	1587.9003	1586.8930	1586.7900	65	1	R.TNEKVELQELNDR.F (Ions score 47)
104 - 112	1115.6313	1114.6240	1114.5618	56	0	K.VELQELNDR.F (Ions score 31)
145 - 154	1254.6449	1253.6376	1253.5598	62	0	R.LGDLYEEEMR.E (Ions score 38)
145 - 154	1270.6376	1269.6303	1269.5547	60	0	R.LGDLYEEEMR.E Oxidation (M) (Ions score 12)
222 - 234	1533.9507	1532.9434	1532.8450	64	1	R.KVESLQEEIAFLK.K (Ions score 40)
[illegible]	[illegible]	[illegible]	[illegible]	[illegible]	[illegible]	[illegible]

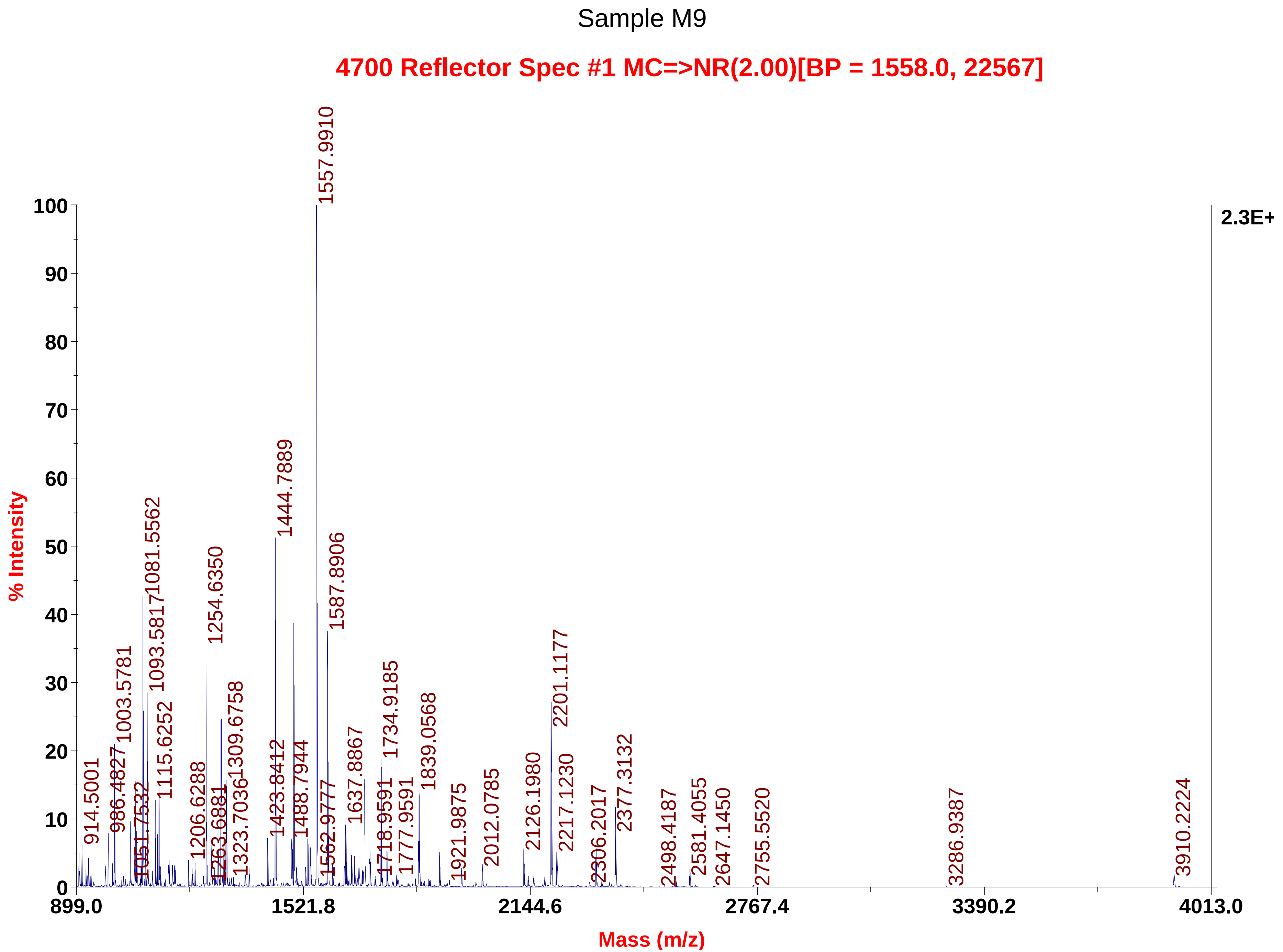
Sample M9
4700 Reflector Spec #1 MC=>NR(2.00)[BP = 1558.0, 22567]
% Intensity
Mass (m/z)
2.3E+
100
90
80
70
60
50
40
30
20
10
0
899.0
1521.8
2144.6
2767.4
3390.2
4013.0
914.5001
986.4827
1003.5781
1051.7532
1081.5562
1093.5817
1115.6252
1206.6288
1254.6350
1263.6881
1309.6758
1323.7036
1423.8412
1444.7889
1488.7944
1557.9910
1562.9777
1587.8906
1637.8867
1718.9591
1734.9185
1777.9591
1839.0568
1921.9875
2012.0785
2126.1980
2201.1177
2217.1230
2306.2017
2377.3132
2498.4187
2581.4055
2647.1450
2755.5520
3286.9387
3910.2224

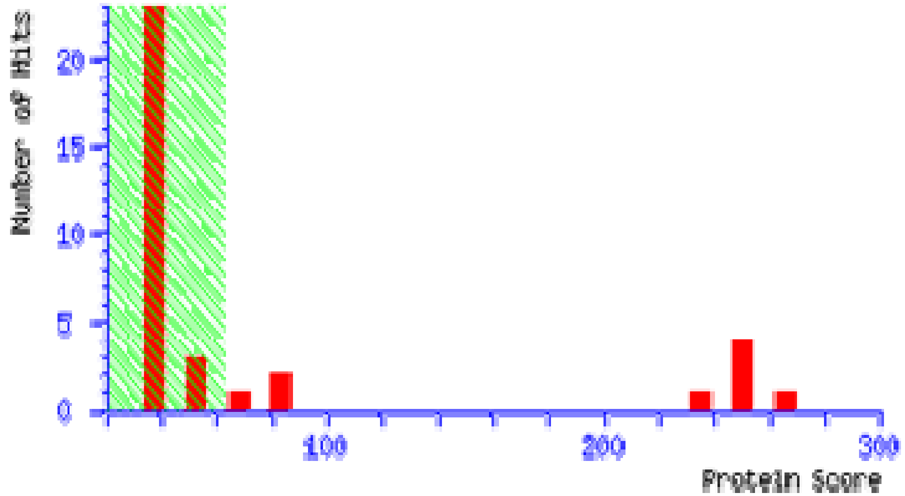

Concise Protein Summary Report

Format As | Concise Protein Summary | Help

Significance threshold p< 0.05 Max. number of hits AUTO

Re-Search All | Search Unmatched

1. Q5FWJ3_MOUSE **Mass**: 53655 **Score**: 265 **Expect**: 2.9e-22 **Matches**: 77
Vimentin (NOD-derived CD11c +ve dendritic cells cDNA, RIKEN full- length enriched library, clone:
VIME_MOUSE **Mass**: 53524 **Score**: 265 **Expect**: 2.9e-22 **Matches**: 77
Vimentin.- Mus musculus (Mouse).
CAA69019 **Mass**: 51533 **Score**: 258 **Expect**: 1.5e-21 **Matches**: 75
MMVIMENT NID: - Mus musculus
Q3TFD9_MOUSE **Mass**: 53656 **Score**: 253 **Expect**: 4.6e-21 **Matches**: 75
17 days embryo heart cDNA, RIKEN full-length enriched library, clone:I920087O13 product:vimentin,

Protein View

Match to: **Q5FWJ3_MOUSE** Score: **265** Expect: **2.9e-22**
Vimentin (NOD-derived CD11c +ve dendritic cells cDNA, RIKEN full- length enriched library,
Found in search of pmf_I2_12990848908.txt

Nominal mass (M_r): **53655**; Calculated pI value: **5.06**
NCBI BLAST search of Q5FWJ3_MOUSE against nr
Unformatted sequence string for pasting into other applications

Taxonomy: Mus musculus
Links to retrieve other entries containing this sequence from NCBI Entrez:
(no taxonomy information for this entry)
(no taxonomy information for this entry)
(no taxonomy information for this entry)
(no taxonomy information for this entry)

Variable modifications: Carbamidomethyl (C),Deamidated (NQ),Oxidation (M)
Cleavage by Trypsin: cuts C-term side of KR unless next residue is P
Number of mass values searched: **181**
Number of mass values matched: **77**
Sequence Coverage: **89%**

Matched peptides shown in **Bold Red**

```
  1 MSTRSVSSSS YRRMFGGSGT SSRPSSNRSY VTTSTRTYSL GSALRPSTSR
 51 SLYSSSPGGA YVTRSSAVRL RSSVPGVRLL QDSVDFSLAD AINTEFKNTR
101 TNEKVELQEL NDRFANYIDK VRFLEQQNKI LLAELEQLKG QGKSRLGDLY
151 EEEMRELRRQ VDQLTNDKAR VEVERDNLAE DIMRLREKLQ EEMLQREEAE
201 STLQSFRQDV DNASLARLDL ERKVESLQEE IAFLKKLHDE EIQELQAQIQ
251 EQHVQIDVDV SKPDLTAALR DVRQQYESVA AKNLQEAEEW YKSKFADLSE
301 AANRNNDALR QAKQESNEYR RQVQSLTCEV DALKGTNESL ERQMREMEEN
351 FALEAANYQD TIGRLQDEIQ NMKEEMARHL REYQDLLNVK MALDIEIATY
401 RKLLEGEESR ISLPLPTFSS LNLRETNLES LPLVDTHSKR TLLIKTVETR
451 DGQVINETSQ HHDDLE
```

Show predicted peptides also

Sort Peptides By ◉ Residue Number ○ Increasing Mass ○ Decreasing Mass

Start	–	End	Observed	Mr(expt)	Mr(calc)	ppm	Miss	Sequence	
5	–	13	1028.5667	1027.5594	1027.5047	53	1	R.SVSSSSYRR.M	
14	–	28	1543.7866	1542.7793	1542.6845	61	0	R.MFGGSGTSSRPSSNR.S	Oxi
29	–	36	914.5001	913.4928	913.4505	46	0	R.SYVTTSTR.T	
29	–	50	2391.3230	2390.3157	2390.2190	40	1	R.SYVTTSTRTYSLGSALRPSTSR	
37	–	50	1495.8713	1494.8640	1494.7790	57	0	R.TYSLGSALRPSTSR.S	
51	–	64	1444.7861	1443.7788	1443.6994	55	0	R.SLYSSSPGGAYVTR.S	
79	–	97	2126.1934	2125.1861	2125.0579	60	0	R.LLQDSVDFSLADAINTEFK.N	
79	–	100	2498.3972	2497.3899	2497.2336	63	1	R.LLQDSVDFSLADAINTEFKNTR	
101	–	113	1587.8877	1586.8804	1586.7900	57	1	R.TNEKVELQELNDR.F	
105	–	113	1115.6230	1114.6157	1114.5618	48	0	K.VELQELNDR.F	
114	–	122	1125.6603	1124.6530	1124.5978	49	1	R.FANYIDKVR.F	
121	–	129	1162.6176	1161.6103	1161.6142	-3	1	K.VRFLEQQNK.I	Deamidate

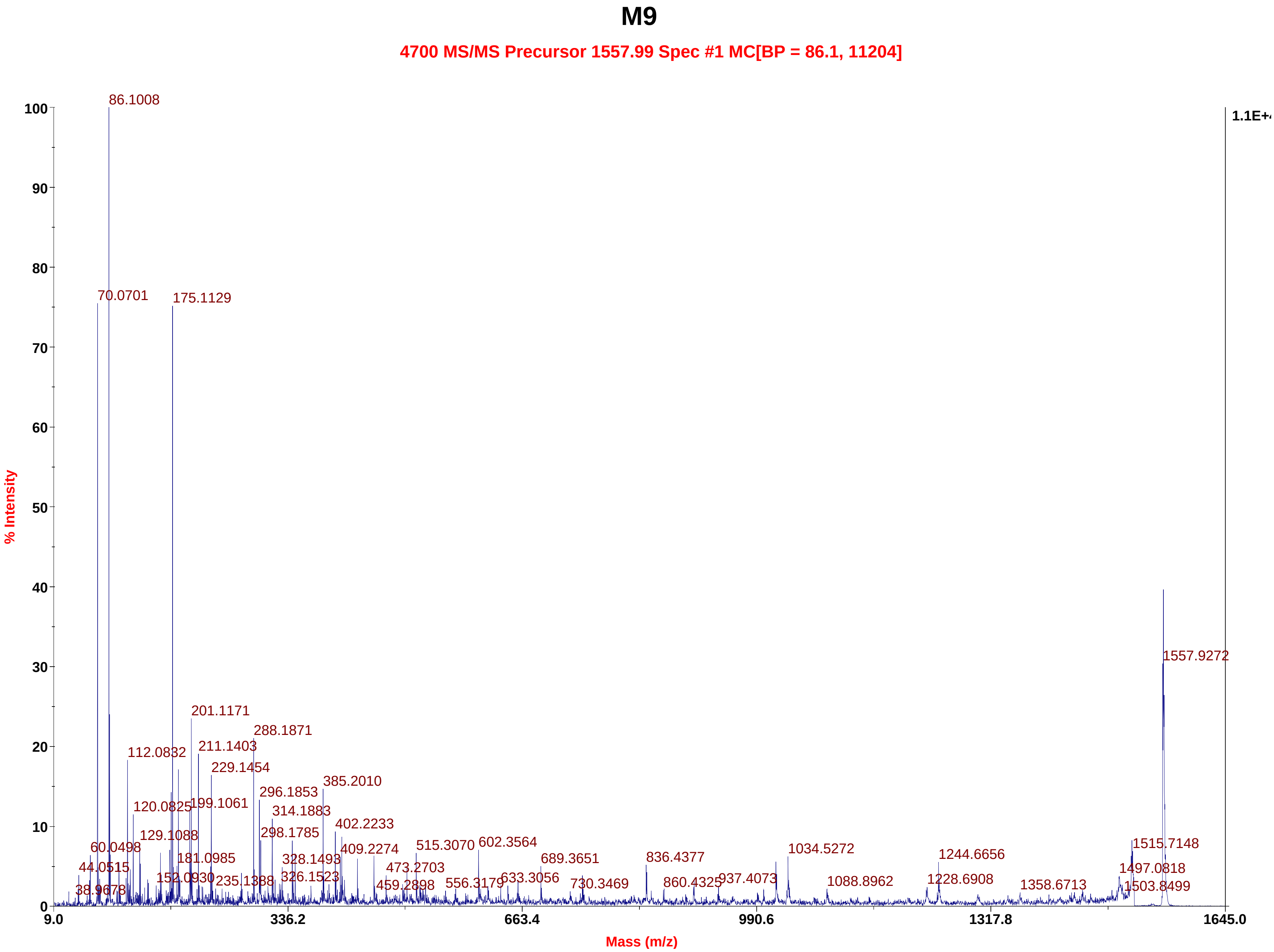
M9
4700 MS/MS Precursor 1557.99 Spec #1 MC[BP = 86.1, 11204]
% Intensity
Mass (m/z)
1.1E+4
86.1008
70.0701
175.1129
201.1171
288.1871
211.1403
112.0832
229.1454
385.2010
296.1853
120.0825
199.1061
314.1883
402.2233
298.1785
129.1088
60.0498
409.2274
515.3070
602.3564
181.0985
328.1493
689.3651
836.4377
1034.5272
1244.6656
1515.7148
1557.9272
44.0515
473.2703
1497.0818
152.0930
235.1388
326.1523
633.3056
937.4073
1088.8962
1228.6908
1358.6713
1503.8499
38.9678
459.2898
556.3179
730.3469
860.4325
9.0
336.2
663.4
990.6
1317.8
1645.0

M9

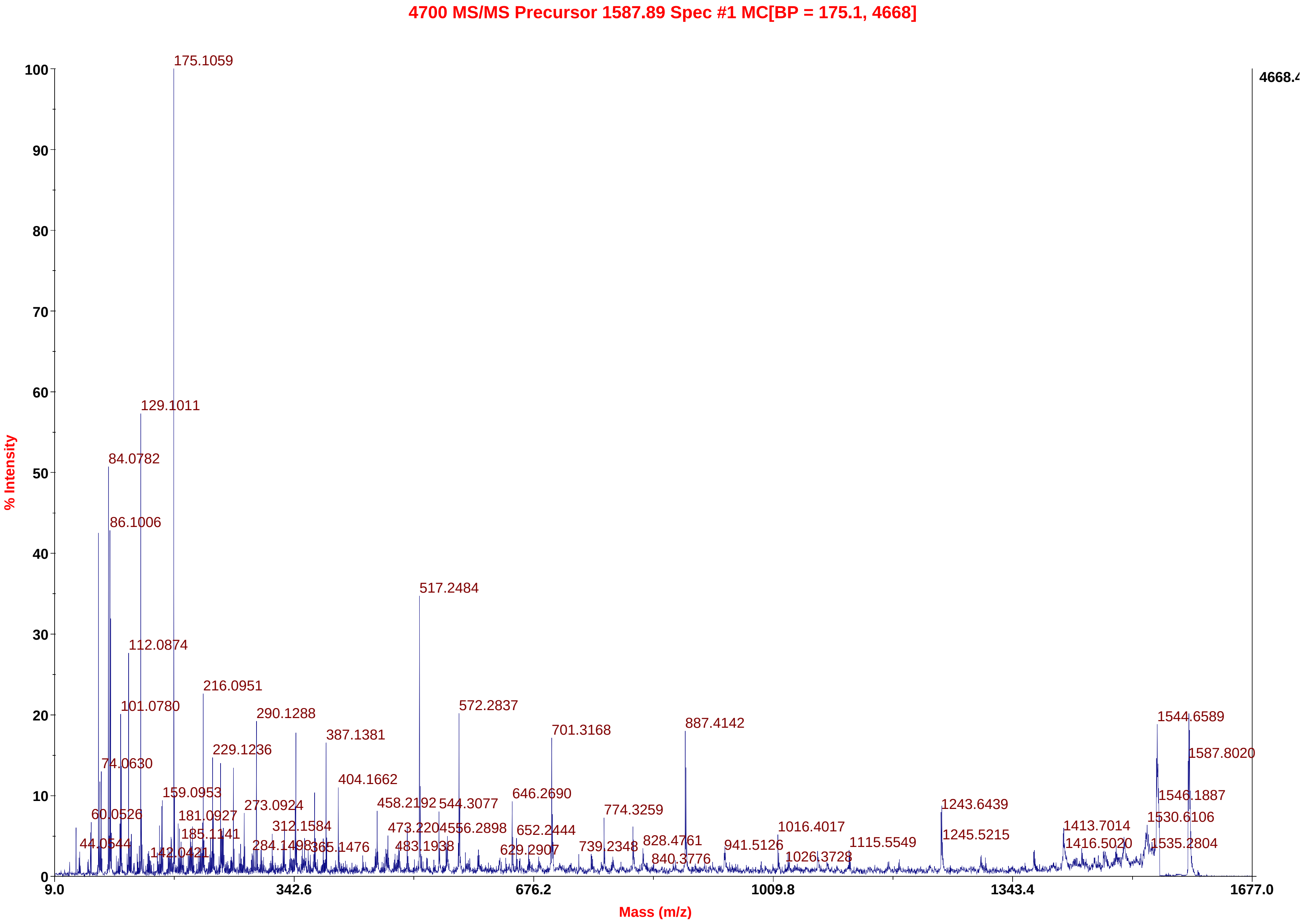
4700 MS/MS Precursor 1587.89 Spec #1 MC[BP = 175.1, 4668]
% Intensity
Mass (m/z)
4668.4
175.1059
129.1011
84.0782
86.1006
112.0874
216.0951
101.0780
290.1288
229.1236
387.1381
74.0630
404.1662
159.0953
60.0526
181.0927
185.1141
44.0544
142.0421
273.0924
312.1584
284.1498
365.1476
458.2192
473.2204
483.1938
517.2484
544.3077
556.2898
572.2837
646.2690
652.2444
629.2907
701.3168
739.2348
774.3259
828.4761
840.3776
887.4142
941.5126
1016.4017
1026.3728
1115.5549
1243.6439
1245.5215
1413.7014
1416.5020
1530.6106
1535.2804
1544.6589
1546.1887
1587.8020
0
10
20
30
40
50
60
70
80
90
100
9.0
342.6
676.2
1009.8
1343.4
1677.0

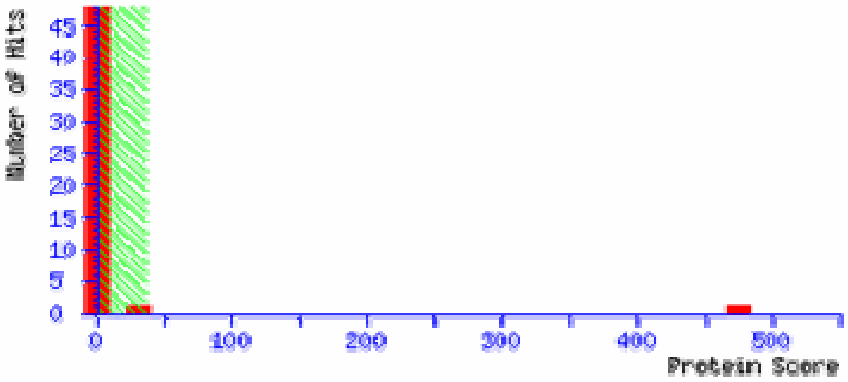

Peptide Summary Report

Format As | Peptide Summary | Help

Significance threshold p< 0.05 | Max. number of hits AUTO

Standard scoring ○ MudPIT scoring ● | Ions score or expect cut-off 0 | Show sub-sets 0

Show pop-ups ● Suppress pop-ups ○ | Sort unassigned Decreasing Score | Require bold red ☐

Select All | Select None | Search Selected | ☐ **Error tolerant**

1. VIME_MOUSE **Mass:** 53524 **Score:** 474 **Matches:** 18(12) **Sequences:** 18(12) **emPAI:** 4.20
Vimentin.- Mus musculus (Mouse).
☐ Check to include this hit in error tolerant search

	Query	Observed	Mr(expt)	Mr(calc)	ppm	Miss	Score	Expect	Rank	Unique	Peptide
☑	2	1046.5822	1045.5749	1045.5226	50.0	0	24	1.2	1	U	K.LQEEMLQR.E
☑	3	1081.5541	1080.5468	1080.4948	48.2	1	27	0.53	1	U	K.QESNEYRR.Q
☑	4	1093.5795	1092.5722	1092.5200	47.8	0	61	0.00028	1	U	K.FADLSEAANR.N
☑	5	1115.6230	1114.6157	1114.5618	48.4	0	31	0.23	1	U	K.VELQELNDR.F
☑	6	1125.6603	1124.6530	1124.5978	49.1	1	35	0.058	1	U	R.FANYIDKVR.F
☑	7	1254.6342	1253.6269	1253.5598	53.6	0	71	2.3e-05	1	U	R.LGDLYEEEMR.E
☑	8	1295.7336	1294.7263	1294.6591	51.9	0	44	0.0081	1	U	K.MALDIEIATYR.K
☑	9	1309.6732	1308.6659	1308.5986	51.5	0	46	0.0081	1	U	K.NLQEAEEWYK.S
☑	10	1444.7861	1443.7788	1443.6994	55.0	0	60	0.0003	1	U	R.SLYSSSPGGAYVTR.S

Protein View

Match to: **VIME_MOUSE** Score: **474**
Vimentin.- Mus musculus (Mouse).
Found in search of ppw_I2_129990855108.txt

Nominal mass (M_r): **53524**; Calculated pI value: **5.06**
NCBI BLAST search of VIME_MOUSE against nr
Unformatted sequence string for pasting into other applications

Taxonomy: Mus musculus

Variable modifications: Carbamidomethyl (C),Deamidated (NQ),Oxidation (M)
Cleavage by Trypsin: cuts C-term side of KR unless next residue is P
Sequence Coverage: **47%**

Matched peptides shown in **Bold Red**

```
  1 STRSVSSSSY RRMFGGSGTS SRPSSNRSYV TTSTRTYSLG SALRPSTSRS
 51 LYSSSPGGAY VTRSSAVRLR SSVPGVRLLQ DSVDFSLADA INTEFKNTRT
101 NEKVELQELN DRFANYIDKV RFLEQQNKIL LAELEQLKGQ GKSRLGDLYE
151 EEMRELRRQV DQLTNDKARV EVERDNLAED IMRLREKLQE EMLQREEAES
201 TLQSFRQDVD NASLARLDLE RKVESLQEEI AFLKKLHDEE IQELQAQIQE
251 QHVQIDVDVS KPDLTAALRD VRQQYESVAA KNLQEAEEWY KSKFADLSEA
301 ANRNNDALRQ AKQESNEYRR QVQSLTCEVD ALKGTNESLE RQMREMEENF
351 ALEAANYQDT IGRLQDEIQN MKEEMARHLR EYQDLLNVKM ALDIEIATYR
401 KLLEGEESRI SLPLPTFSSL NLRETNLESL PLVDTHSKRT LLIKTVETRD
451 GQVINETSQH HDDLE
```

Show predicted peptides also

Sort Peptides By ◉ Residue Number ○ Increasing Mass ○ Decreasing Mass

Start – End	Observed	Mr(expt)	Mr(calc)	ppm	Miss	Sequence
36 – 49	1495.8713	1494.8640	1494.7790	57	0	R.TYSLGSALRPSTSR.S (Ions score 8)
50 – 63	1444.7861	1443.7788	1443.6994	55	0	R.SLYSSSPGGAYVTR.S (Ions score 60)
78 – 96	2126.1934	2125.1861	2125.0579	60	0	R.LLQDSVDFSLADAINTEFK.N (Ions score 75)
100 – 112	1587.8877	1586.8804	1586.7900	57	1	R.TNEKVELQELNDR.F (Ions score 71)
104 – 112	1115.6230	1114.6157	1114.5618	48	0	K.VELQELNDR.F (Ions score 31)
113 – 121	1125.6603	1124.6530	1124.5978	49	1	R.FANYIDKVR.F (Ions score 35)
145 – 154	1254.6342	1253.6269	1253.5598	54	0	R.LGDLYEEEMR.E (Ions score 71)
170 – 183	1688.9269	1687.9196	1687.8199	59	1	R.VEVERDNLAEDIMR.L (Ions score 30)
188 – 195	1046.5822	1045.5749	1045.5226	50	0	K.LQEEMLQR.E (Ions score 24)
188 – 206	2324.2590	2323.2517	2323.1114	60	1	K.LQEEMLQREEAESTLQSFR.Q (Ions score 42)
282 – 291	1309.6732	1308.6659	1308.5986	51	0	K.NLQEAEEWYK.S (Ions score 46)

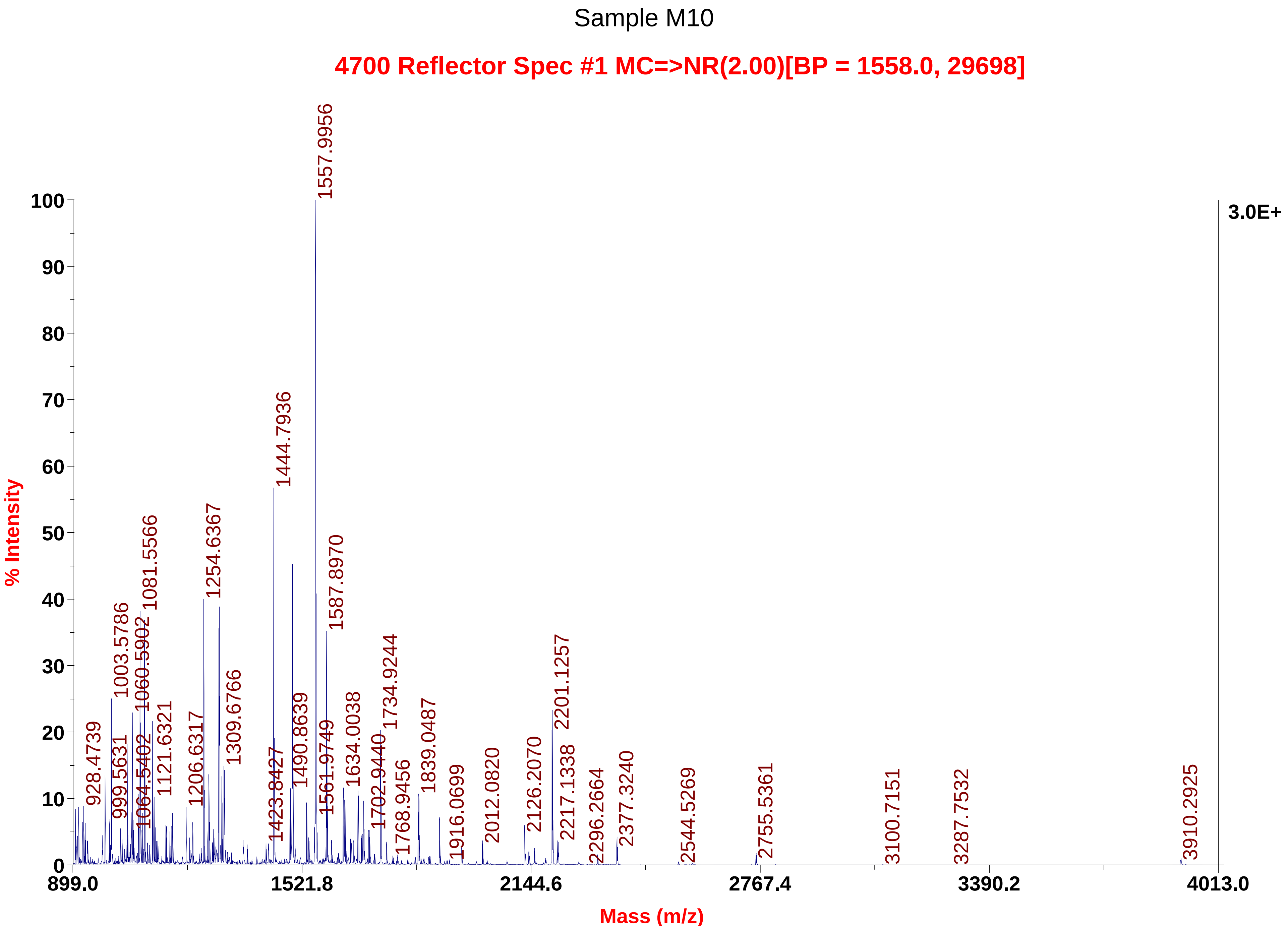
Sample M10
4700 Reflector Spec #1 MC=>NR(2.00)[BP = 1558.0, 29698]
% Intensity
Mass (m/z)
3.0E+
899.0
1521.8
2144.6
2767.4
3390.2
4013.0
1557.9956
1444.7936
1254.6367
1081.5566
1587.8970
1003.5786
1060.5902
1309.6766
1121.6321
928.4739
999.5631
1064.5402
1206.6317
1423.8427
1490.8639
1561.9749
1634.0038
1702.9440
1734.9244
1768.9456
1839.0487
1916.0699
2012.0820
2126.2070
2201.1257
2217.1338
2296.2664
2377.3240
2544.5269
2755.5361
3100.7151
3287.7532
3910.2925

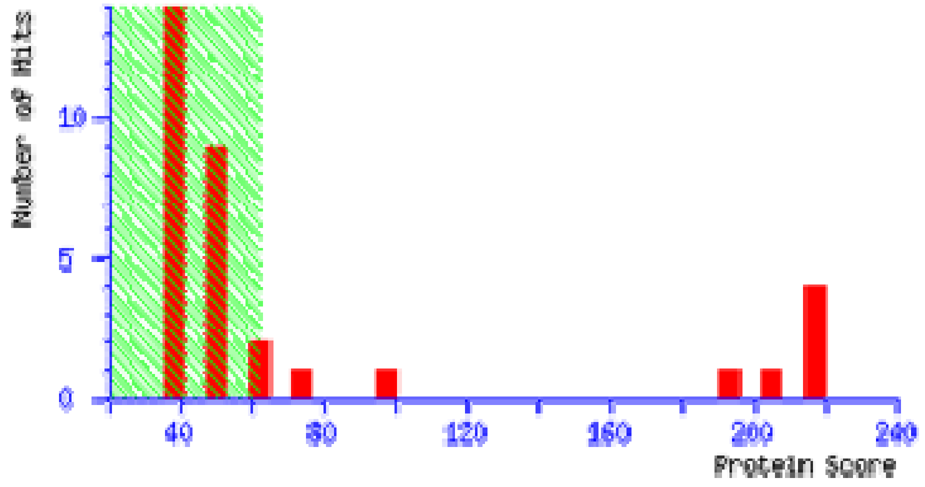

Concise Protein Summary Report

Format As | Concise Protein Summary | Help

Significance threshold p< 0.05 Max. number of hits AUTO

Re-Search All | Search Unmatched

1. CAA39807 **Mass**: 53689 **Score**: 217 **Expect**: 1.8e-17 **Matches**: 64
MMVMTM NID: - Mus musculus

---

2. VIME_MOUSE **Mass**: 53524 **Score**: 216 **Expect**: 2.3e-17 **Matches**: 65
Vimentin.- Mus musculus (Mouse).
Q5FWJ3_MOUSE **Mass**: 53655 **Score**: 216 **Expect**: 2.3e-17 **Matches**: 65
Vimentin (NOD-derived CD11c +ve dendritic cells cDNA, RIKEN full- length enriched library, [illegible]
CAA69019 **Mass**: 51533 **Score**: 210 **Expect**: 9.2e-17 **Matches**: 64
MMVIMENT NID: - Mus musculus

Protein View

Match to: **CAA39807** Score: **217** Expect: **1.8e-17**
MMVMTM NID: - Mus musculus
Found in search of pmf_J2_129990849009.txt

Nominal mass (M_r): **53689**; Calculated pI value: **5.06**
NCBI BLAST search of CAA39807 against nr
Unformatted sequence string for pasting into other applications

Taxonomy: Mus musculus

Variable modifications: Carbamidomethyl (C),Deamidated (NQ),Oxidation (M)
Cleavage by Trypsin: cuts C-term side of KR unless next residue is P
Number of mass values searched: **178**
Number of mass values matched: **64**
Sequence Coverage: **83%**

Matched peptides shown in **Bold Red**

```
  1 MSTRSVSSSS YRRMFGGSGT SSRPSSNRSY VTTSTRTYSL GSALRPSTSR
 51 SLYSSSPGGA YVTRSSAVRL RSSVPGVRLL QDSVDFSLAD AINTEFKNTR
101 TNEKVELQEL NDRFANYIDK VRFLEQQNKI LLAELEQLKG QGKSRLGDLY
151 EEEMRELRRQ VDQFTNDKAR VEVERDNLAE DIMRLREKLQ EEMLQREEAE
201 STLQSFRQDV DNASLARLDL ERKVESLQEE IAFLKKLHDE EIQELQAQIQ
251 EQHVQIDVDV SKPDLTAALR DVRQQYESVA AKNLQEAEEW YKSKFADLSE
301 AANRNNDALR QAKQESNEYR RQVQSLTCEV DALKGTNESL ERQMREMEEN
351 FALEAANYQD TIGRLQDEIQ NMKEEMARHL REYQDLLNVK MALDIEIATY
401 RKLLEGEESR ISLPLPTFSS LNLRETNLES LPLVDTHSKR TLLIKTVETR
451 DGQVINETSQ HHDDLE
```

Show predicted peptides also

Sort Peptides By ◉ Residue Number ○ Increasing Mass ○ Decreasing Mass

Start - End	Observed	Mr(expt)	Mr(calc)	ppm	Miss	Sequence
2 - 12	1216.6931	1215.6858	1215.5844	83	1	M.STRSVSSSSYR.R
5 - 13	1028.5896	1027.5823	1027.5047	76	1	R.SVSSSSYRR.M
29 - 36	914.4987	913.4914	913.4505	45	0	R.SYVTTSTR.T
29 - 50	2391.3303	2390.3230	2390.2190	44	1	R.SYVTTSTRTYSLGSALRPSTSR.S
37 - 50	1495.8760	1494.8687	1494.7790	60	0	R.TYSLGSALRPSTSR.S
51 - 64	1444.7904	1443.7831	1443.6994	58	0	R.SLYSSSPGGAYVTR.S
79 - 97	2126.2061	2125.1988	2125.0579	66	0	R.LLQDSVDFSLADAINTEFK.N
101 - 113	1587.8940	1586.8867	1586.7900	61	1	R.TNEKVELQELNDR.F
105 - 113	1115.6248	1114.6175	1114.5618	50	0	K.VELQELNDR.F

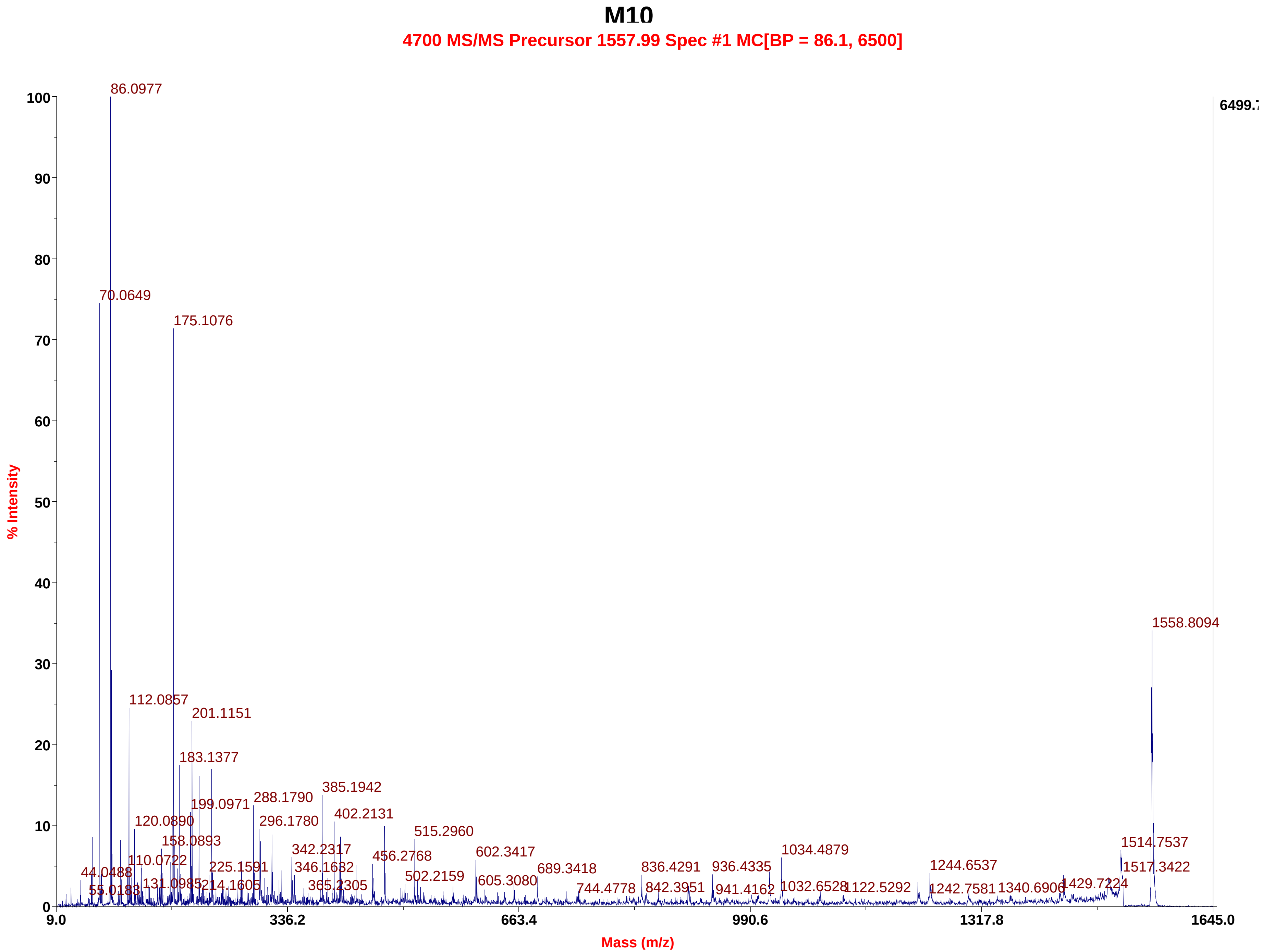
M10
4700 MS/MS Precursor 1557.99 Spec #1 MC[BP = 86.1, 6500]
% Intensity
Mass (m/z)
6499.7
86.0977
70.0649
175.1076
112.0857
201.1151
183.1377
199.0971
120.0890
158.0893
110.0722
44.0488
55.0183
131.0985
214.1605
225.1591
288.1790
296.1780
385.1942
402.2131
342.2317
346.1632
365.2305
456.2768
502.2159
515.2960
602.3417
605.3080
689.3418
744.4778
836.4291
842.3951
936.4335
941.4162
1034.4879
1032.6528
1122.5292
1244.6537
1242.7581
1340.6906
1429.7224
1514.7537
1517.3422
1558.8094

M10

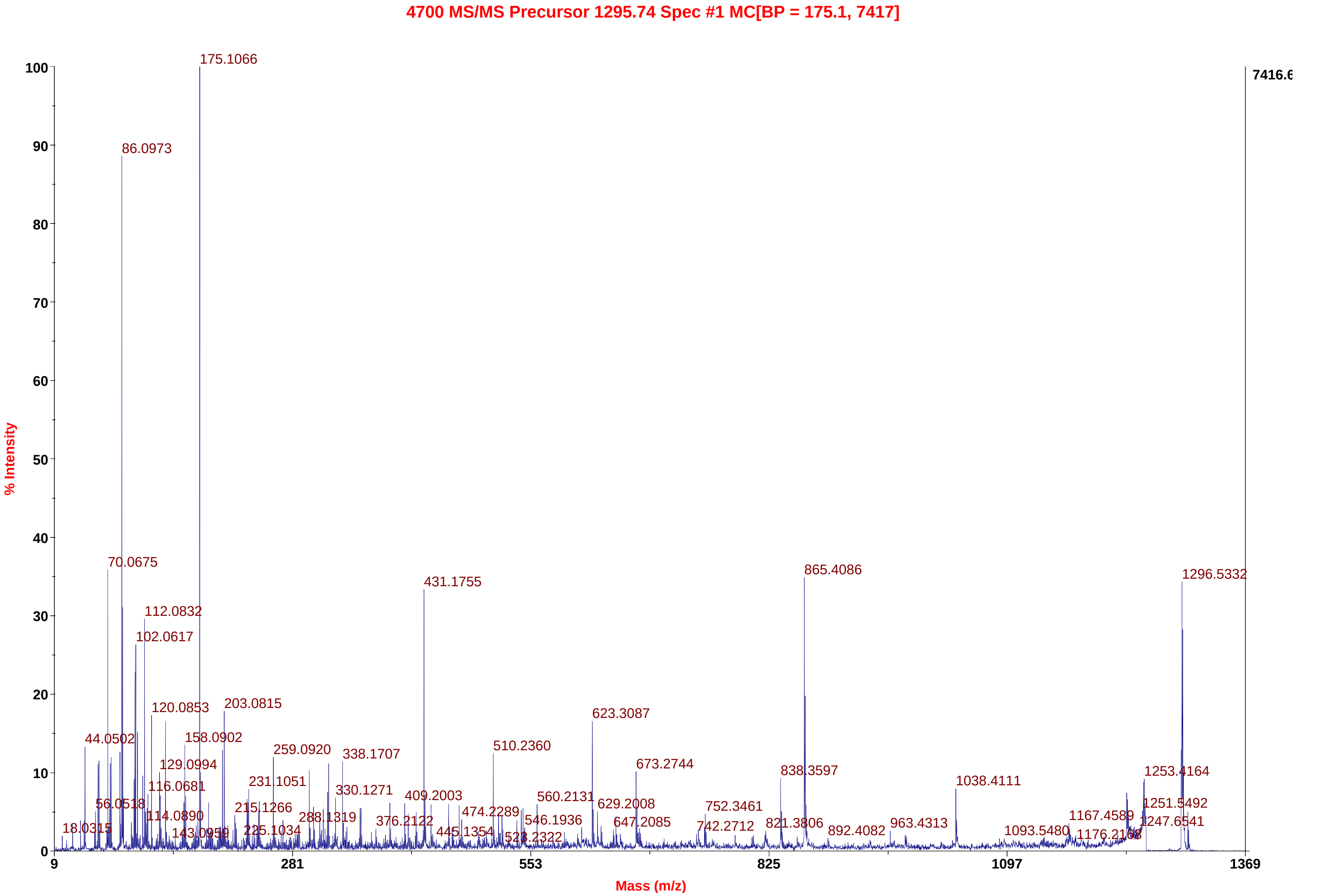
4700 MS/MS Precursor 1295.74 Spec #1 MC[BP = 175.1, 7417]
% Intensity
Mass (m/z)
7416.6
175.1066
86.0973
70.0675
112.0832
102.0617
120.0853
203.0815
44.0502
158.0902
129.0994
116.0681
56.0518
114.0890
18.0315
143.0959
215.1266
225.1034
231.1051
259.0920
288.1319
338.1707
330.1271
376.2122
409.2003
431.1755
445.1354
474.2289
510.2360
523.2322
546.1936
560.2131
623.3087
629.2008
647.2085
673.2744
742.2712
752.3461
821.3806
838.3597
865.4086
892.4082
963.4313
1038.4111
1093.5480
1167.4589
1176.2168
1247.6541
1251.5492
1253.4164
1296.5332

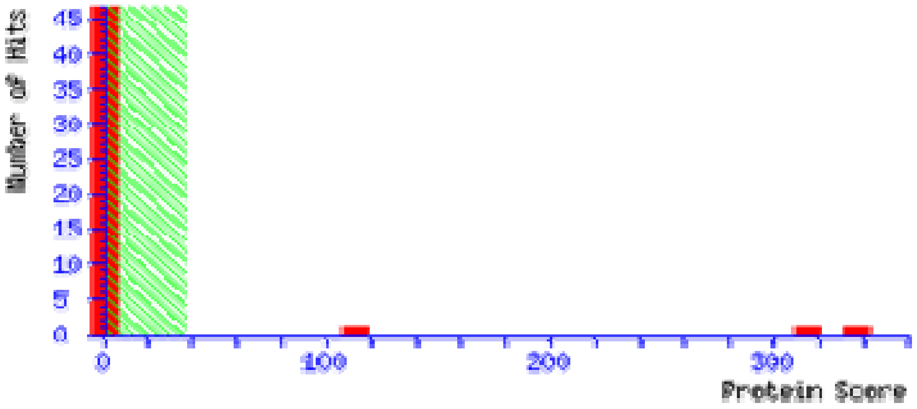

Peptide Summary Report

Format As | Peptide Summary | Help

Significance threshold p< 0.05 | Max. number of hits AUTO

Standard scoring ○ MudPIT scoring ● | Ions score or expect cut-off 0 | Show sub-sets 0

Show pop-ups ● Suppress pop-ups ○ | Sort unassigned Decreasing Score | Require bold red ☐

Select All | Select None | Search Selected | ☐ **Error tolerant**

1. VIME_MOUSE **Mass:** 53524 **Score:** 337 **Matches:** 15(9) **Sequences:** 15(9) **emPAI:** 1.53
Vimentin.- Mus musculus (Mouse).
☐ Check to include this hit in error tolerant search

	Query	Observed	Mr(expt)	Mr(calc)	ppm	Miss	Score	Expect	Rank	Unique	Peptide
☑	3	1046.5817	1045.5744	1045.5226	49.6	0	27	0.61	1		K.LQEEMLQR.E
☑	4	1081.5563	1080.5490	1080.4948	50.2	1	16	6.2	1		K.QESNEYRR.Q
☑	5	1093.5818	1092.5745	1092.5200	49.9	0	58	0.00047	1		K.FADLSEAANR.N
☑	6	1115.6248	1114.6175	1114.5618	50.0	0	32	0.18	1		K.VELQELNDR.F
☑	7	1254.6362	1253.6289	1253.5598	55.2	0	72	1.7e-05	1		R.LGDLYEEEMR.E
☑	9	1295.7373	1294.7300	1294.6591	54.8	0	36	0.055	1		K.MALDIEIATYR.K
☑	10	1309.6765	1308.6692	1308.5986	54.0	0	39	0.034	1		K.NLQEAEEWYK.S

Protein View

Match to: **VIME_MOUSE** Score: 337
Vimentin.- Mus musculus (Mouse).
Found in search of ppw_J2_129990855309.txt

Nominal mass (M_r): **53524**; Calculated pI value: **5.06**
NCBI BLAST search of VIME_MOUSE against nr
Unformatted sequence string for pasting into other applications

Taxonomy: Mus musculus

Variable modifications: Carbamidomethyl (C),Deamidated (NQ),Oxidation (M)
Cleavage by Trypsin: cuts C-term side of KR unless next residue is P
Sequence Coverage: 36%

Matched peptides shown in **Bold Red**

1 STRSVSSSSY RRMFGGSGTS SRPSSNRSYV TTSTR**TYSLG** **SALRPSTSRS**
51 **LYSSSPGGAY** **VTR**SSAVRLR SSVPGVRLLQ DSVDFSLADA INTEFKNTR**T**
101 **NEKVELQELN** **DR**FANYIDKV RFLEQQNKIL LAELEQLKGQ GKSR**LGDLYE**
151 **EEMR**ELRRQV DQLTNDKAR**V** **EVERDNLAED** **IMR**LREK**LQE** **EMLQR**EEAES
201 TLQSFRQDVD NASLARLDLE R**KVESLQEEI** **AFLK**KLHDEE IQELQAQIQE
251 QHVQIDVDVS KPDLTAALRD VRQQYESVAA K**NLQEAEEWY** **K**SK**FADLSEA**
301 **ANR**NNDALRQ AK**QESNEYRR** QVQSLTCEVD ALKGTNESLE RQMR**EMEENF**
351 **ALEAANYQDT** **IGRLQDEIQN** **MKEEMAR**HLR EYQDLLNVK**M** **ALDIEIATYR**
401 KLLEGEESR**I** **SLPLPTFSSL** **NLR**ETNLESL PLVDTHSKRT LLIKTVETRD
451 GQVINETSQH HDDLE

Show predicted peptides also

Sort Peptides By ◉ Residue Number ○ Increasing Mass ○ Decreasing Mass

Start – End	Observed	Mr(expt)	Mr(calc)	ppm	Miss	Sequence	
36 – 49	1495.8760	1494.8687	1494.7790	60	0	R.TYSLGSALRPSTSR.S	(Ions score 7)
50 – 63	1444.7904	1443.7831	1443.6994	58	0	R.SLYSSSPGGAYVTR.S	(Ions score 39)
100 – 112	1587.8940	1586.8867	1586.7900	61	1	R.TNEKVELQELNDR.F	(Ions score 66)
104 – 112	1115.6248	1114.6175	1114.5618	50	0	K.VELQELNDR.F	(Ions score 32)
145 – 154	1254.6362	1253.6289	1253.5598	55	0	R.LGDLYEEEMR.E	(Ions score 72)
170 – 183	1688.9337	1687.9264	1687.8199	63	1	R.VEVERDNLAEDIMR.L	(Ions score 14)
188 – 195	1046.5817	1045.5744	1045.5226	50	0	K.LQEEMLQR.E	(Ions score 27)
222 – 234	1533.9412	1532.9339	1532.8450	58	1	R.KVESLQEEIAFLK.K	(Ions score 44)
282 – 291	1309.6765	1308.6692	1308.5986	54	0	K.NLQEAEEWYK.S	(Ions score 39)
294 – 303	1093.5818	1092.5745	1092.5200	50	0	K.FADLSEAANR.N	(Ions score 58)
313 – 320	1081.5563	1080.5490	1080.4948	50	1	K.QESNEYRR.Q	(Ions score 16)
345 – 363	2201.1218	2200.1145	2199.9742	64	0	R.EMEENFALEAANYQDTIGR.L	(Ions score 122)
364 – 377	1734.9233	1733.9160	1733.8076	63	1	R.LQDEIQNMKEEMAR.H	(Ions score 95)
390 – 400	1295.7373	1294.7300	1294.6591	55	0	K.MALDIEIATYR.K	(Ions score 36)
410 – 423	1557.9924	1556.9851	1556.8926	59	0	R.ISLPLPTFSSLNLR.E	(Ions score 51)

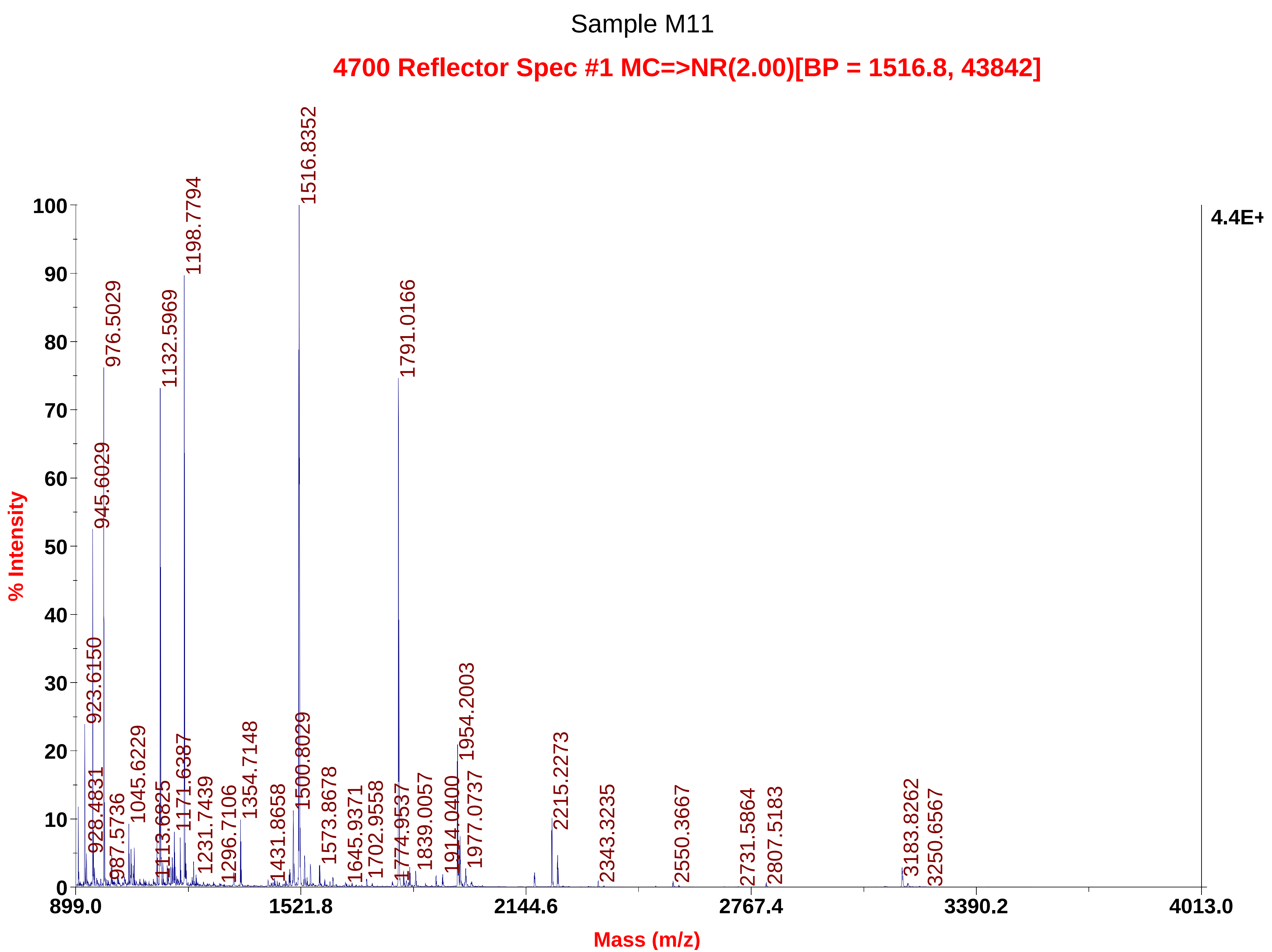

Sample M11
4700 Reflector Spec #1 MC=>NR(2.00)[BP = 1516.8, 43842]
% Intensity
Mass (m/z)
4.4E+
0
10
20
30
40
50
60
70
80
90
100
899.0
1521.8
2144.6
2767.4
3390.2
4013.0
923.6150
928.4831
945.6029
976.5029
987.5736
1045.6229
1113.6825
1132.5969
1171.6387
1198.7794
1231.7439
1296.7106
1354.7148
1431.8658
1500.8029
1516.8352
1573.8678
1645.9371
1702.9558
1774.9537
1791.0166
1839.0057
1914.0400
1954.2003
1977.0737
2215.2273
2343.3235
2550.3667
2731.5864
2807.5183
3183.8262
3250.6567

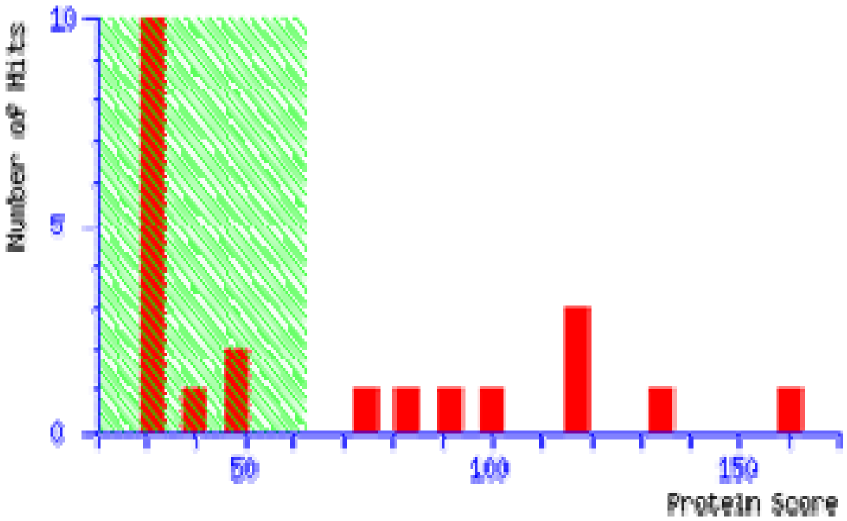

Concise Protein Summary Report

Format As | Concise Protein Summary | Help

Significance threshold p< 0.05 Max. number of hits AUTO

Re-Search All | Search Unmatched

1. Q3TVP6_MOUSE **Mass:** 41724 **Score:** 160 **Expect:** 9.2e-12 **Matches:** 37
   Osteoclast-like cell cDNA, RIKEN full-length enriched library, clone:I420044I15 product:actin, beta, c
   Q3UBP6_MOUSE **Mass:** 41742 **Score:** 136 **Expect:** 2.3e-09 **Matches:** 33
   Bone marrow macrophage cDNA, RIKEN full-length enriched library, clone:I830016H03 product:actin, beta,
   ATCHB **Mass:** 41710 **Score:** 136 **Expect:** 2.3e-09 **Matches:** 35
   actin beta - chicken
   ATHUG **Mass:** 41766 **Score:** 136 **Expect:** 2.3e-09 **Matches:** 35
   actin gamma 1 - human

Protein View

Match to: **Q3TVP6_MOUSE** Score: **160** Expect: **9.2e-12**
Osteoclast-like cell cDNA, RIKEN full-length enriched library, clone:I420044I15 product:acti
Found in search of pmf_K2_129990849010.txt

Nominal mass (M_r): **41724**; Calculated pI value: **5.30**
NCBI BLAST search of Q3TVP6_MOUSE against nr
Unformatted sequence string for pasting into other applications

Taxonomy: Mus musculus
Links to retrieve other entries containing this sequence from NCBI Entrez:
(no taxonomy information for this entry)

Variable modifications: Carbamidomethyl (C),Deamidated (NQ),Oxidation (M)
Cleavage by Trypsin: cuts C-term side of KR unless next residue is P
Number of mass values searched: **125**
Number of mass values matched: **37**
Sequence Coverage: **82%**

Matched peptides shown in **Bold Red**

```
  1 MDDDIAALVV DNGSGMCKAG FAGDDAPRAV FPSIVGRPRH QGVMVGMGQK
 51 DSYVGDEAQS KRGILTLKYP IEHGIVTNWE DMEKIWHHTF YNELRVAPEE
101 HPVLLTEAPL NPKANREKMT QIMFETFNTP AMYVAIQAVL SLYASGRTTG
151 IVMDSGDGVT HTVPIYEGYA LPHAILRLDL AGRDLTDYLM KILTERGYSF
201 TTTAEREIVR DIKEKLCYVA LDFEQEMATA ASSSSLEKSY ELPDGQVITI
251 GNERFRCPEA LFQPSFLGME SCGIHETTFN SIMKCDVDIR KDLYANTVLS
301 GGTTMYPGIA DRMQKEITAL APSTMKIKII APPERKYSVW IGGSILASLS
351 TFQQMWISKQ EYDESGPSIV HRKCF
```

Show predicted peptides also

Sort Peptides By ◉ Residue Number ○ Increasing Mass ○ Decreasing Mass

Start	–	End	Observed	Mr(expt)	Mr(calc)	ppm	Miss	Sequence	
19	–	28	976.5027	975.4955	975.4410	56	0	K.AGFAGDDAPR.A	
29	–	39	1198.7769	1197.7696	1197.6982	60	0	R.AVFPSIVGRPR.H	
40	–	50	1171.6378	1170.6305	1170.5638	57	0	R.HQGVMVGMGQK.D	
40	–	50	1173.6477	1172.6404	1172.5318	93	0	R.HQGVMVGMGQK.D	2 Deamida
40	–	50	1187.6346	1186.6273	1186.5587	58	0	R.HQGVMVGMGQK.D	Oxidation
40	–	50	1203.6329	1202.6256	1202.5536	60	0	R.HQGVMVGMGQK.D	2 Oxidati
51	–	62	1354.7119	1353.7046	1353.6161	65	1	K.DSYVGDEAQSKR.G	
69	–	84	1961.0471	1960.0398	1959.9036	69	0	K.YPIEHGIVTNWEDMEK.I	
69	–	84	1977.0839	1976.0766	1975.8986	90	0	K.YPIEHGIVTNWEDMEK.I	Oxida
85	–	95	1515.8496	1514.8423	1514.7419	66	0	K.IWHHTFYNELR.V	
85	–	95	1516.8330	1515.8257	1515.7259	66	0	K.IWHHTFYNELR.V	Deamidated
96	–	113	1954.1995	1953.1922	1953.0571	69	0	R.VAPEEHPVLLTEAPLNPK.A	
96	–	116	2295.3442	2294.3369	2294.2382	43	1	R.VAPEEHPVLLTEAPLNPKANR.E	

M11

4700 MS/MS Precursor 1515.85 Spec #1 MC[BP = 110.1, 4191]

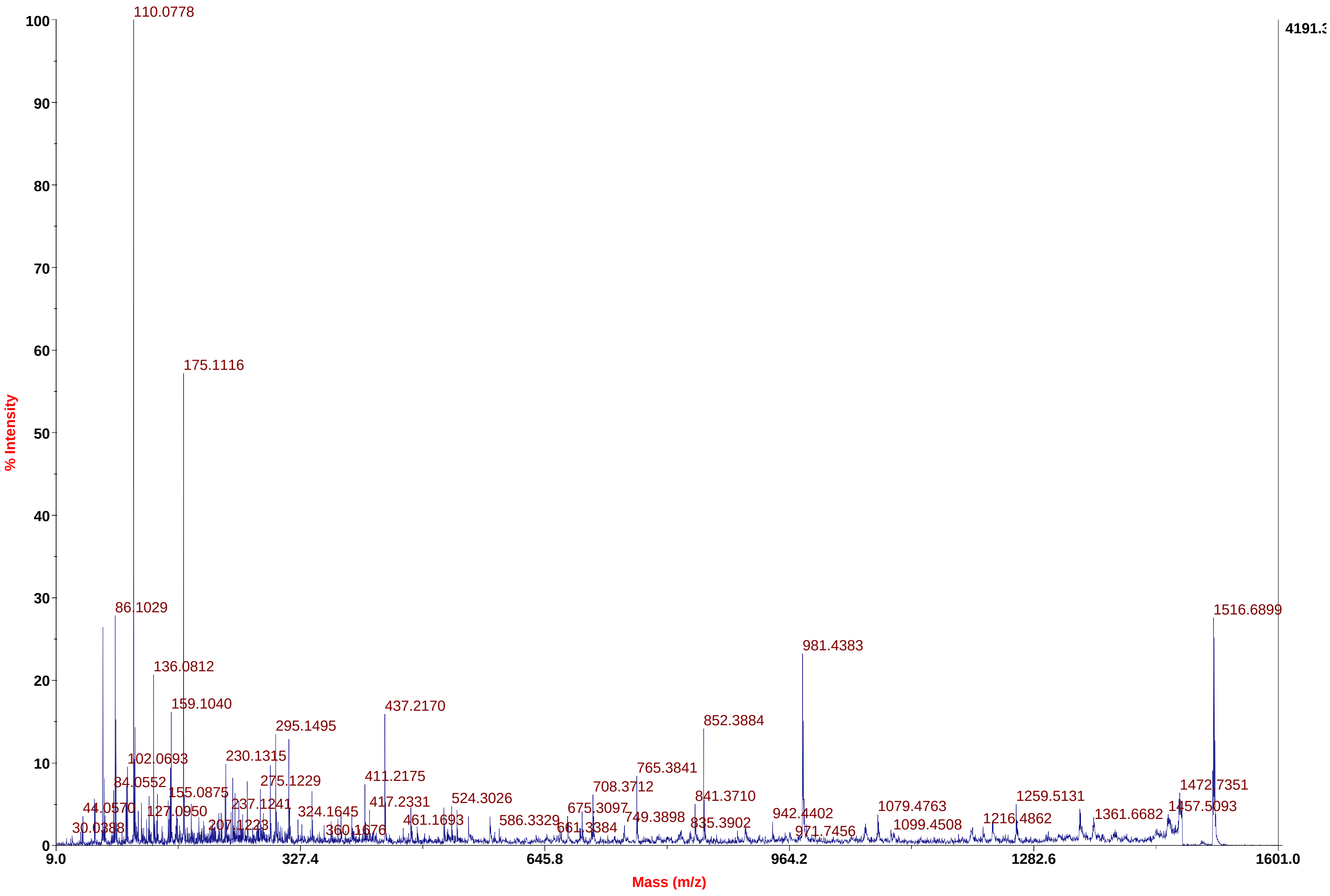

M11

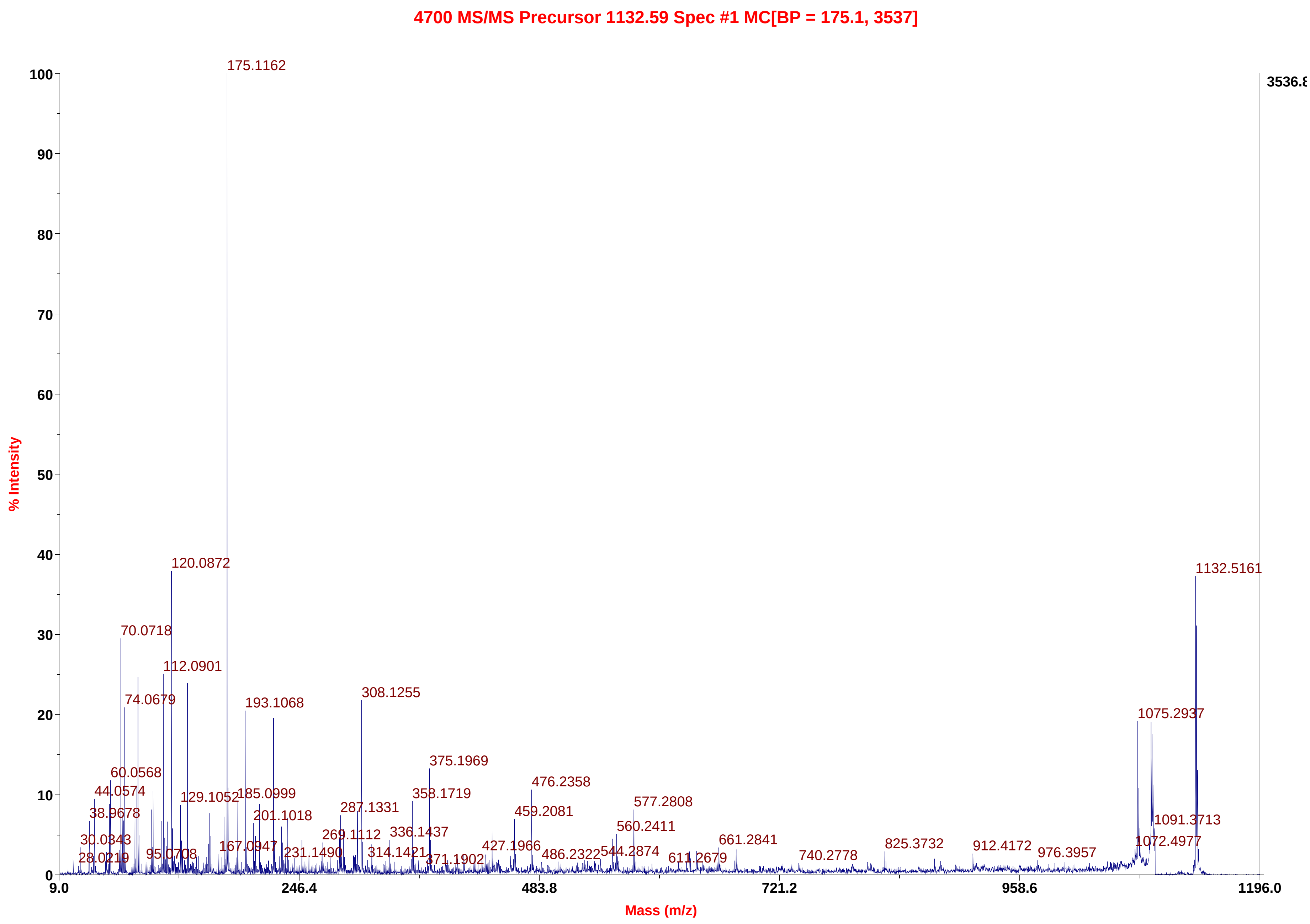
4700 MS/MS Precursor 1132.59 Spec #1 MC[BP = 175.1, 3537]
% Intensity
Mass (m/z)
3536.8
175.1162
120.0872
70.0718
112.0901
74.0679
193.1068
308.1255
1132.5161
1075.2937
375.1969
476.2358
60.0568
44.0574
129.1052
185.0999
358.1719
577.2808
38.9678
201.1018
287.1331
459.2081
560.2411
1091.3713
30.0343
269.1112
336.1437
661.2841
825.3732
912.4172
1072.4977
28.0219
95.0708
167.0947
231.1490
314.1421
371.1902
427.1966
486.2322
544.2874
611.2679
740.2778
976.3957
9.0
246.4
483.8
721.2
958.6
1196.0
0
10
20
30
40
50
60
70
80
90
100

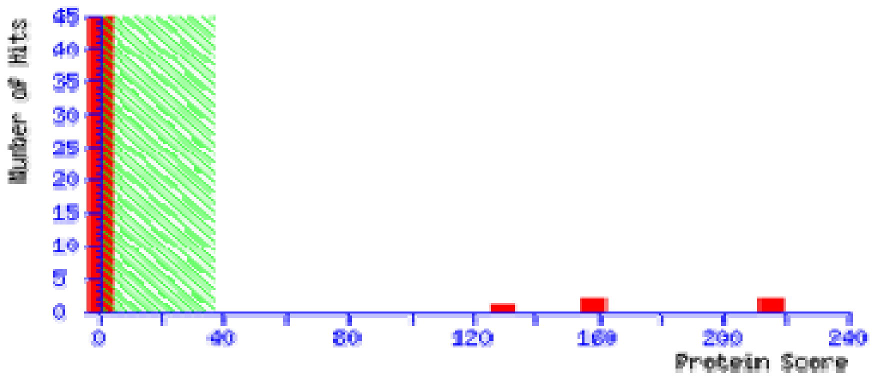

Peptide Summary Report

Format As | Peptide Summary | Help

Significance threshold p< 0.05 | Max. number of hits AUTO

Standard scoring ○ MudPIT scoring ● | Ions score or expect cut-off 0 | Show sub-sets 0

Show pop-ups ● Suppress pop-ups ○ | Sort unassigned Decreasing Score | Require bold red ☐

Select All | Select None | Search Selected | ☐ Error tolerant

1. CAA27396 **Mass:** 39161 **Score:** 215 **Matches:** 13(8) **Sequences:** 11(8) **emPAI:** 2.23
MMACTBR2 NID: - Mus musculus
☐ Check to include this hit in error tolerant search

	Query	Observed	Mr(expt)	Mr(calc)	ppm	Miss	Score	Expect	Rank	Unique	Peptide
☑	1	923.6166	922.6093	922.5600	53.5	1	18	0.64	1		K.IIAPPERK.Y
☑	2	945.6028	944.5956	944.5444	54.2	0	37	0.028	1	U	R.AVFPSIVGR.S
☑	4	998.5252	997.5179	997.4790	39.0	0	8	37	1		R.DLTDYLMK.I
☑	8	1132.5944	1131.5871	1131.5197	59.6	0	58	0.00054	1		R.GYSFTTTAER.E
☑	10	1171.6378	1170.6305	1170.5638	57.0	0	81	2.1e-06	1		R.HQGVMVGMGQK.D
☑	11	1187.6346	1186.6273	1186.5587	57.8	0	(18)	5.4	1		R.HQGVMVGMGQK.D + Oxida
☑	13	1354.7119	1353.7046	1353.6161	65.4	1	39	0.037	1		K.DSYVGDEAQSKR.G
☑	15	1515.8496	1514.8423	1514.7419	66.3	0	27	0.45	1		K.IWHHTFYNELR.V
☑	16	1791.0154	1790.0081	1789.8846	69.0	0	60	0.00021	1		K.SYELPDGQVITIGNER.F
☑	24	1954.1995	1953.1922	1953.0571	69.2	0	42	0.0051	1		R.VAPEEHPVLLTEAPLNPK.A

Protein View

Match to: **CAA27396** Score: **215**
MMACTBR2 NID: - Mus musculus
Found in search of ppw_K2_129990855410.txt

Nominal mass (M_r): **39161**; Calculated pI value: **5.78**
NCBI BLAST search of CAA27396 against nr
Unformatted sequence string for pasting into other applications

Taxonomy: Mus musculus
Links to retrieve other entries containing this sequence from NCBI Entrez:
(no taxonomy information for this entry)

Variable modifications: Carbamidomethyl (C),Deamidated (NQ),Oxidation (M)
Cleavage by Trypsin: cuts C-term side of KR unless next residue is P
Sequence Coverage: **44%**

Matched peptides shown in **Bold Red**

```
  1 PRAVFPSIVG RSRHQGVMVG MGQKDSYVGD EAQSKRGILT LKYPIEHGIV
 51 TNWDDMEKIW HHTFYNELRV APEEHPVLLT EAPLNPKANR EKMTQIMFET
101 FNTPAMYVAI QAVLSLYASG RTTGIVMDSG DGVTHTVPIY EGYALPHAIL
151 RLDLAGRDLT DYLMKILTER GYSFTTTAER EIVRDIKEKL CYVALDFEQE
201 MATAASSSSL EKSYELPDGQ VITIGNERFR CPEALFQPSF LGMESCGIHE
251 TTFNSIMKCD VDIRKDLYAN TVLSGGTTMY PGIADRMQKE ITALAPSTMK
301 IKIIAPPERK YSVWIGGSIL ASLSTFQQMW ISKQEYDESG PSIVHRKCF
```

Show predicted peptides also

Sort Peptides By ◉ Residue Number ○ Increasing Mass ○ Decreasing Mass

Start - End	Observed	Mr(expt)	Mr(calc)	ppm	Miss	Sequence
3 - 11	945.6028	944.5956	944.5444	54	0	R.AVFPSIVGR.S (Ions score 37)
14 - 24	1171.6378	1170.6305	1170.5638	57	0	R.HQGVMVGMGQK.D (Ions score 81)
14 - 24	1187.6346	1186.6273	1186.5587	58	0	R.HQGVMVGMGQK.D Oxidation (M) (Ions score
25 - 36	1354.7119	1353.7046	1353.6161	65	1	K.DSYVGDEAQSKR.G (Ions score 39)
59 - 69	1515.8496	1514.8423	1514.7419	66	0	K.IWHHTFYNELR.V (Ions score 27)
70 - 87	1954.1995	1953.1922	1953.0571	69	0	R.VAPEEHPVLLTEAPLNPK.A (Ions score 42)
122 - 151	3183.8391	3182.8318	3182.6071	71	0	R.TTGIVMDSGDGVTHTVPIYEGYALPHAILR.L (Ions s
158 - 165	998.5252	997.5179	997.4790	39	0	R.DLTDYLMK.I (Ions score 8)
171 - 180	1132.5944	1131.5871	1131.5197	60	0	R.GYSFTTTAER.E (Ions score 58)
213 - 228	1791.0154	1790.0081	1789.8846	69	0	K.SYELPDGQVITIGNER.F (Ions score 60)

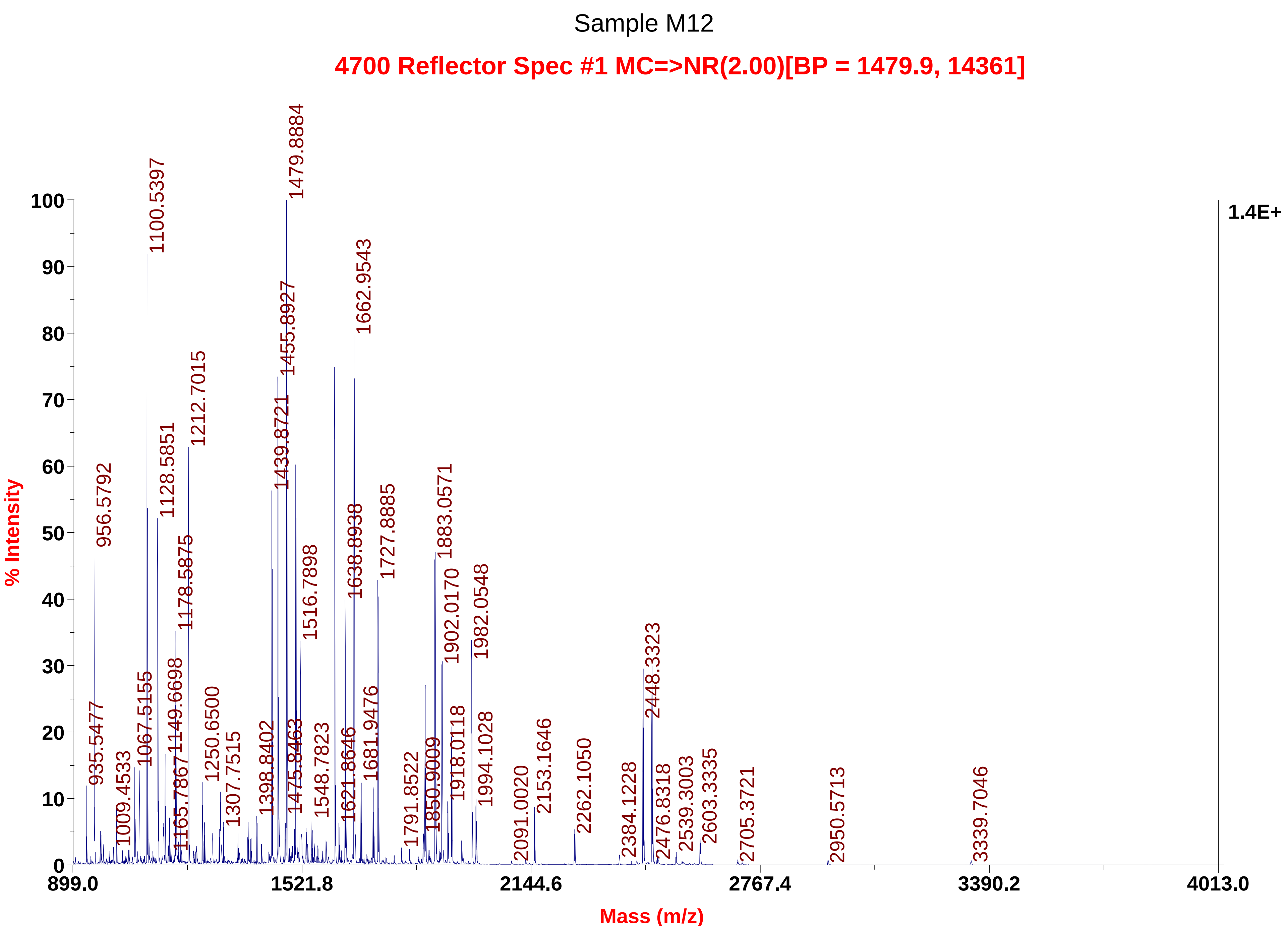

Sample M12
4700 Reflector Spec #1 MC=>NR(2.00)[BP = 1479.9, 14361]
% Intensity
Mass (m/z)
1.4E+
100
90
80
70
60
50
40
30
20
10
0
899.0
1521.8
2144.6
2767.4
3390.2
4013.0
935.5477
956.5792
1009.4533
1067.5155
1100.5397
1128.5851
1149.6698
1165.7867
1178.5875
1212.7015
1250.6500
1307.7515
1398.8402
1439.8721
1455.8927
1475.8463
1479.8884
1516.7898
1548.7823
1621.8646
1638.8938
1662.9543
1681.9476
1727.8885
1791.8522
1850.9009
1883.0571
1902.0170
1918.0118
1982.0548
1994.1028
2091.0020
2153.1646
2262.1050
2384.1228
2448.3323
2476.8318
2539.3003
2603.3335
2705.3721
2950.5713
3339.7046

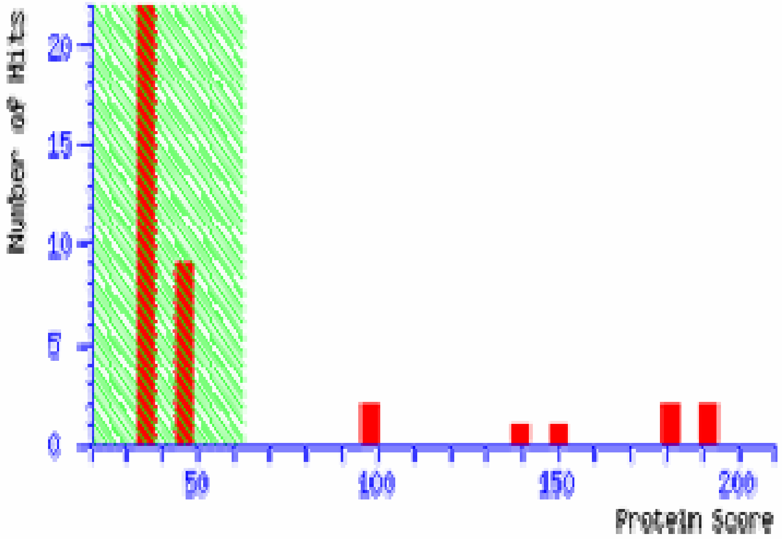

Concise Protein Summary Report

Format As | Concise Protein Summary | Help

Significance threshold p< 0.05 Max. number of hits AUTO

Re-Search All | Search Unmatched

1. **Mixture 1** **Total score:** 192 **Expect:** 5.8e-15 **Matches:** 76

Components (only one family member shown for each component):

Q8C7C7_MOUSE **Mass:** 64961 **Score:** 154 **Expect:** 3.7e-11 **Matches:** 54

2 days neonate thymus thymic cells cDNA, RIKEN full-length enriched library, clone:C920028B14 product:albumin 1,

AAB49490 **Mass:** 50514 **Score:** 98 **Expect:** 1.4e-05 **Matches:** 27

MMU89889 NID: - Mus musculus

Protein View

Match to: **Q8C7C7_MOUSE** Score: **154** Expect: **3.7e-11**
2 days neonate thymus thymic cells cDNA, RIKEN full-length enriched library, clone:C920028B14 pro
Found in search of pmf_L2_129990849011.txt

Nominal mass (M_r): **64961**; Calculated pI value: **5.49**
NCBI BLAST search of Q8C7C7_MOUSE against nr
Unformatted sequence string for pasting into other applications

Taxonomy: Mus musculus
Links to retrieve other entries containing this sequence from NCBI Entrez:
(no taxonomy information for this entry)

Variable modifications: Carbamidomethyl (C),Deamidated (NQ),Oxidation (M)
Cleavage by Trypsin: cuts C-term side of KR unless next residue is P
Number of mass values searched: **164**
Number of mass values matched: **54**
Sequence Coverage: **71%**

Matched peptides shown in **Bold Red**

```
  1 NRYNDLGEQH FKGLVLIAFS QYLQKCSYDE HAKLVQEVTD FAKTCVADES
 51 AANCDKSLHT LFGDKLCAIP NLRENYGELA DCCTKQEPER NECFLQHKDD
101 NPSLPPFERP EAEAMCTSFK ENPTTFMGHY LHEVARRHPY FYAPELLYYA
151 EQYNEILTQC CAEADKESCL TPKLDGVKEK ALVSSVRQRM KCSSMQKFGE
201 RAFKAWAVAR LSQTFPNADF AEITKLATDL TKVNKECCHG DLLECADDRA
251 ELAKYMCENQ ATISSKLQTC CDKPLLKKAH CLSEVEHDTM PADLPAIAAD
301 FVEDQEVCKN YAEAKDVFLG TFLYEYSRRH PDYSVSLLLR LAKKYEATLE
351 KCCAEANPPA CYGTVLAEFQ PLVEEPKNLV KTNCDLYEKL GEYGFQNAIL
401 VRYTQKAPQV STPTLVEAAR NLGRVGTKCC TLPEDQRLPC VEDYLSAILN
451 RVCLLHEKTP VSEHVTKCCS GSLVERRPCF SALTVDETYV PKEFKAETFT
501 FHSDICTLPE KEKQIKKQTA LAELVKHKPK ATAEQLKTVM DDFAQFLDTC
551 CKAADKDTCF STEGPNLVTR CKDALA
```

Show predicted peptides also

Sort Peptides By ◉ Residue Number ○ Increasing Mass ○ Decreasing Mass

Start - End	Observed	Mr(expt)	Mr(calc)	ppm	Miss	Sequence
1 - 12	1520.8607	1519.8534	1519.7168	90	1	-.NRYNDLGEQHFK.G
3 - 12	1250.6466	1249.6393	1249.5727	53	0	R.YNDLGEQHFK.G
3 - 25	2711.5913	2710.5840	2710.4119	64	1	R.YNDLGEQHFKGLVLIAFSQYLQK.C
13 - 25	1479.8870	1478.8797	1478.8497	20	0	K.GLVLIAFSQYLQK.C
13 - 33	2470.3696	2469.3623	2469.2362	51	1	K.GLVLIAFSQYLQKCSYDEHAK.L Carb
13 - 33	2472.3184	2471.3111	2471.2042	43	1	K.GLVLIAFSQYLQKCSYDEHAK.L Carb
26 - 33	1009.4545	1008.4472	1008.3971	50	0	K.CSYDEHAK.L Carbamidomethyl (
34 - 43	1149.6699	1148.6626	1148.6077	48	0	K.LVQEVTDFAK.T
57 - 65	1017.5802	1016.5729	1016.5291	43	0	K.SLHTLFGDK.L
57 - 73	1898.0306	1897.0233	1897.0244	-1	1	K.SLHTLFGDKLCAIPNLR.E

Protein View

Match to: **AAB49490** Score: **98** Expect: **1.4e-05**
MMU89889 NID: - Mus musculus
Found in search of pmf_L2_129990849011.txt

Nominal mass (M_r): **50514**; Calculated pI value: **7.32**
NCBI BLAST search of AAB49490 against nr
Unformatted sequence string for pasting into other applications

Taxonomy: Mus musculus

Variable modifications: Carbamidomethyl (C),Deamidated (NQ),Oxidation (M)
Cleavage by Trypsin: cuts C-term side of KR unless next residue is P
Number of mass values searched: **164**
Number of mass values matched: **27**
Sequence Coverage: **56%**

Matched peptides shown in **Bold Red**

1 ALNILVLLGL CWSLAVASPL PTANGRVAEV ENGTKPDSDV PEHCLDTWSF
51 DAATMDHNGT MLFFKGEFVW RGHSGTRELI SAR**WKNPITS** **VDAAFRGPDS**
101 **VFLIKEDKVW** **VYPPEKK**ENG YPK**LFQEEFP** **GIPYPPDAAV** **ECHRGECQSE**
151 **GVLFFQGNRK** **WFWDFATR**TQ KERSWSTVGN CTAALRWLER **YYCFQGNK**FL
201 R**FNPVTGEVP** **PRYPLDAR**DY FVSCPGQGHG RPRNGTAHGN STHPMHSR**CS**
251 **PDPGLTALLS** **DHRGATYAFT** **GSHYWR**LDSS RDGWHSWPIA HHWPQGPSTV
301 DAAFSWDDK**V** **YLIQGTQVYV** **FLTKGGNNLV** **SGYPK**RLEKE **LGSPPGISLE**
351 **TIDAAFSCPG** **SSR**LYVSSGR **RLWWLDLKSG** **AQATWTEVSW** **PHEK**VDGALC
401 LDK**SLGPNTC** **SSNGSSLYFI** **HGPNLYCYSS** **IDKLNAAKSL** **PQPQKVSILG**
451 **CSQ**

Show predicted peptides also

Sort Peptides By ◉ Residue Number ○ Increasing Mass ○ Decreasing Mass

Start - End	Observed	Mr(expt)	Mr(calc)	ppm	Miss	Sequence	
84 - 96	1504.8777	1503.8704	1503.7834	58	1	R.WKNPITSVDAAFR.G	
86 - 96	1190.6776	1189.6703	1189.6091	51	0	K.NPITSVDAAFR.G	
97 - 105	975.5921	974.5848	974.5437	42	0	R.GPDSVFLIK.E	
97 - 108	1347.7872	1346.7799	1346.7082	53	1	R.GPDSVFLIKEDK.V	
109 - 116	1017.5802	1016.5729	1016.5331	39	0	K.VWVYPPEK.K	
109 - 117	1145.6559	1144.6486	1144.6281	18	1	K.VWVYPPEKK.E	
124 - 144	2472.3184	2471.3111	2471.1580	62	0	K.LFQEEFPGIPYPPDAAVECHR.G	Carbami
145 - 159	1727.8855	1726.8782	1726.7733	61	0	R.GECQSEGVLFFQGNR.K	Carbamidometh
145 - 160	1855.9944	1854.9871	1854.8683	64	1	R.GECQSEGVLFFQGNRK.W	Carbamidomet
160 - 168	1256.6888	1255.6815	1255.6138	54	1	R.KWFWDFATR.T	
161 - 168	1128.5824	1127.5751	1127.5189	50	0	K.WFWDFATR.T	
191 - 198	1079.5121	1078.5048	1078.4542	47	0	R.YYCFQGNK.F	Carbamidomethyl (C)

M12

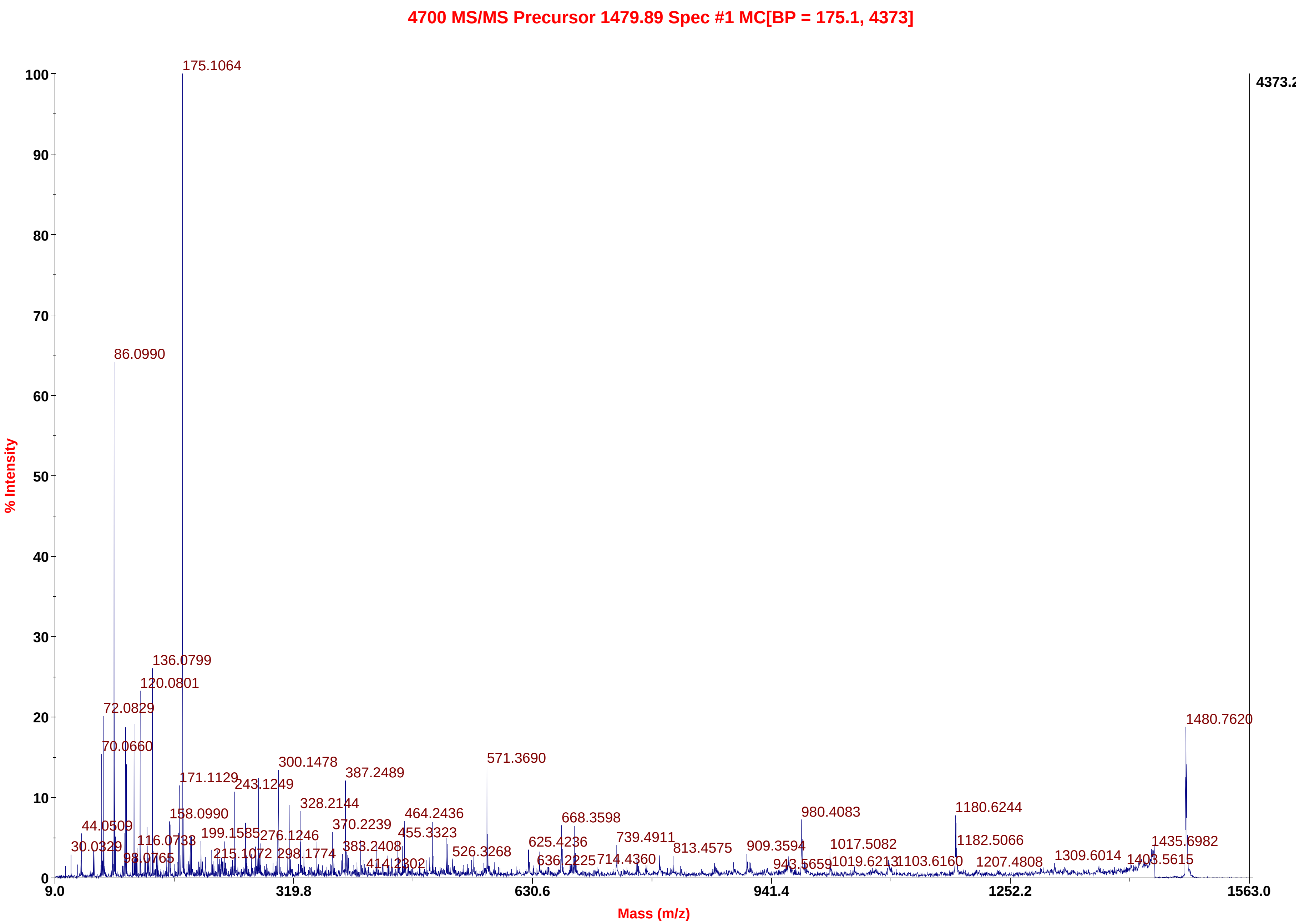

4700 MS/MS Precursor 1479.89 Spec #1 MC[BP = 175.1, 4373]
% Intensity
Mass (m/z)
4373.2
175.1064
86.0990
136.0799
120.0801
72.0829
70.0660
44.0509
30.0329
116.0733
98.0765
158.0990
171.1129
199.1585
215.1072
243.1249
276.1246
298.1774
300.1478
328.2144
370.2239
383.2408
387.2489
414.2302
455.3323
464.2436
526.3268
571.3690
625.4236
636.2225
668.3598
714.4360
739.4911
813.4575
909.3594
943.5659
980.4083
1017.5082
1019.6213
1103.6160
1180.6244
1182.5066
1207.4808
1309.6014
1403.5615
1435.6982
1480.7620

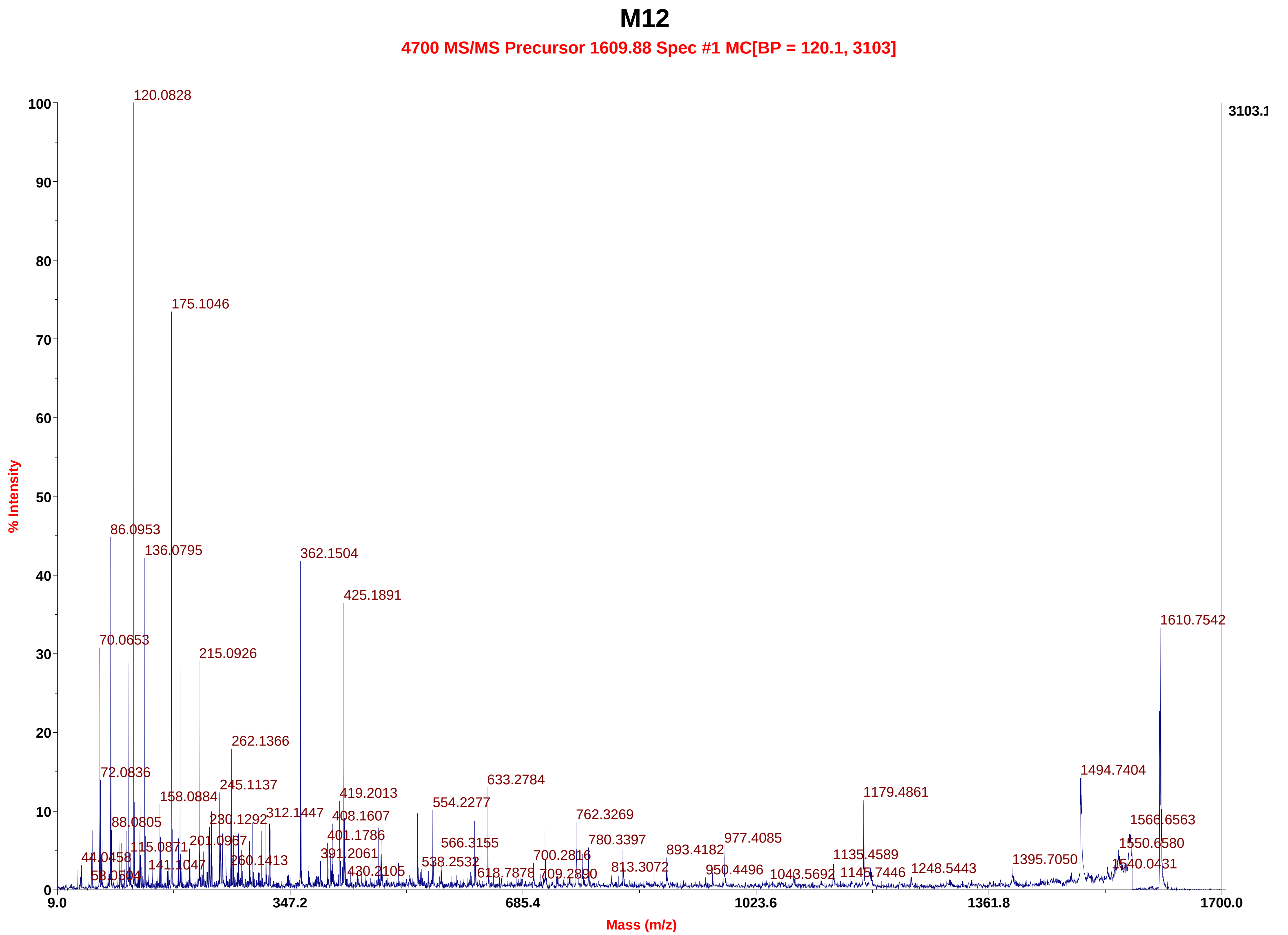
M12
4700 MS/MS Precursor 1609.88 Spec #1 MC[BP = 120.1, 3103]
% Intensity
Mass (m/z)
120.0828
175.1046
86.0953
136.0795
362.1504
425.1891
1610.7542
70.0653
215.0926
262.1366
72.0836
245.1137
158.0884
633.2784
1494.7404
419.2013
1179.4861
554.2277
312.1447
408.1607
762.3269
230.1292
1566.6563
88.0805
401.1786
977.4085
201.0967
780.3397
566.3155
1550.6580
115.0871
893.4182
391.2061
1135.4589
1395.7050
700.2816
44.0458
260.1413
538.2532
813.3072
141.1047
1540.0431
1248.5443
430.2105
950.4496
1145.7446
618.7878
709.2890
53.0504
1043.5692
3103.1
9.0
347.2
685.4
1023.6
1361.8
1700.0

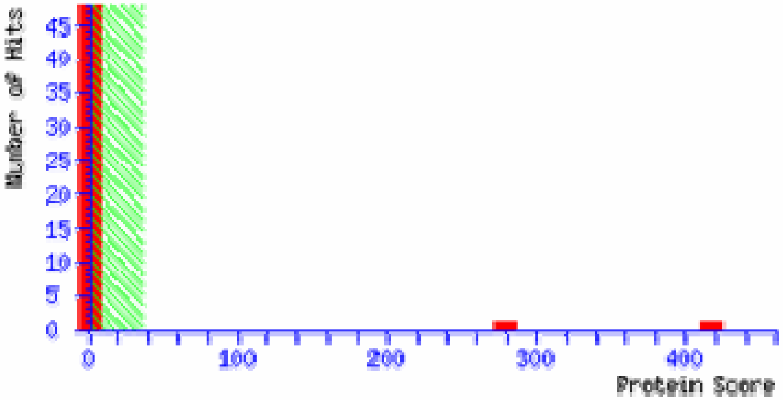

Peptide Summary Report

Format As | Peptide Summary | Help

Significance threshold p< 0.05 Max. number of hits AUTO

Standard scoring ○ MudPIT scoring ● Ions score or expect cut-off 0 Show sub-sets 0

Show pop-ups ● Suppress pop-ups ○ Sort unassigned Decreasing Score Require bold red ☐

Select All | Select None | Search Selected | ☐ **Error tolerant**

1. Q3UKP2_MOUSE **Mass:** 51285 **Score:** 417 **Matches:** 10(9) **Sequences:** 10(9) **emPAI:** 1.72
14 days embryo liver cDNA, RIKEN full-length enriched library, clone:I530016F13 product:hemopexin, full insert s
☐ Check to include this hit in error tolerant search

	Query	Observed	Mr(expt)	Mr(calc)	ppm	Miss	Score	Expect	Rank	Unique	Peptide
☑	2	1100.5388	1099.5315	1099.4757	50.8	0	45	0.01	1	U	R.DYFVSCPGR.G + Carbamidomethyl (C)
☑	3	1128.5824	1127.5751	1127.5189	49.9	0	18	4.3	1	U	K.WFWDFATR.T
☑	5	1212.7012	1211.6939	1211.6299	52.9	0	65	5.1e-05	1	U	R.FNPVTGEVPPR.Y
☑	9	1504.8777	1503.8704	1503.7834	57.9	1	56	0.00054	1	U	R.WKNPITSVDAAFR.G
☑	10	1516.7863	1515.7790	1515.6895	59.1	0	53	0.0016	1	U	R.GATYAFTGSHYWR.L
☑	12	1638.8927	1637.8854	1637.7832	62.4	0	79	3.4e-06	1	U	R.CSPDPGLTALLSDHR.G + Carbamidometh
☑	14	1727.8855	1726.8782	1726.7733	60.7	0	112	2e-09	1	U	R.GECQSEGVLFFQGNR.K + Carbamidometh

Protein View

Match to: **Q3UKP2_MOUSE** Score: **417**
14 days embryo liver cDNA, RIKEN full-length enriched library, clone:I530016F13 product:hemopexin, full inse
Found in search of ppw_L2_129990855611.txt

Nominal mass (M_r): **51285**; Calculated pI value: **7.92**
NCBI BLAST search of Q3UKP2_MOUSE against nr
Unformatted sequence string for pasting into other applications

Taxonomy: Mus musculus
Links to retrieve other entries containing this sequence from NCBI Entrez:
(no taxonomy information for this entry)

Variable modifications: Carbamidomethyl (C),Deamidated (NQ),Oxidation (M)
Cleavage by Trypsin: cuts C-term side of KR unless next residue is P
Sequence Coverage: **28%**

Matched peptides shown in **Bold Red**

```
  1 MARTAVALNI LVLLGLCWSL AVASPLPTAN GRVAEVENGT KPDSDVPEHC
 51 LDTWSFDAAT MDHNGTMLFF KGEFVWRGHS GTRELISARW KNPITSVDAA
101 FRGPDSVFLI KEDKVWVYPP EKKENGYPKL FQEEFPGIPY PPDAAVECHR
151 GECQSEGVLF FQGNRKWFWD FATRTQKERS WSTVGNCTAA LRWLERYYCF
201 QGNKFLRFNP VTGEVPPRYP LDARDYFVSC PGRGHGRPRN GTAHGNSTHP
251 MHSRCSPDPG LTALLSDHRG ATYAFTGSHY WRLDSSRDGW HSWPIAHHWP
301 QGPSTVDAAF SWDDKVYLIQ GTQVYVFLTK GGNNLVSGYP KRLEKELGSP
351 PGISLETIDA AFSCPGSSRL YVSSGRRLWW LDLKSGAQAT WTEVSWPHEK
401 VDGALCLDKS LGPNTCSSNG SSLYFIHGPN LYCYSSIDKL NAAKSLPQPQ
451 KVNSILGCSQ
```

Show predicted peptides also

Sort Peptides By ◉ Residue Number ○ Increasing Mass ○ Decreasing Mass

Start – End	Observed	Mr(expt)	Mr(calc)	ppm	Miss	Sequence
90 – 102	1504.8777	1503.8704	1503.7834	58	1	R.WKNPITSVDAAFR.G (Ions score 56)
130 – 150	2472.3184	2471.3111	2471.1580	62	0	K.LFQEEFPGIPYPPDAAVECHR.G Carbamidomethy
151 – 165	1727.8855	1726.8782	1726.7733	61	0	R.GECQSEGVLFFQGNR.K Carbamidomethyl (C)
151 – 166	1855.9944	1854.9871	1854.8683	64	1	R.GECQSEGVLFFQGNRK.W Carbamidomethyl (C)
167 – 174	1128.5824	1127.5751	1127.5189	50	0	K.WFWDFATR.T (Ions score 18)
208 – 218	1212.7012	1211.6939	1211.6299	53	0	R.FNPVTGEVPPR.Y (Ions score 65)
225 – 233	1100.5388	1099.5315	1099.4757	51	0	R.DYFVSCPGR.G Carbamidomethyl (C) (Ions s
255 – 269	1638.8927	1637.8854	1637.7832	62	0	R.CSPDPGLTALLSDHR.G Carbamidomethyl (C)
270 – 282	1516.7863	1515.7790	1515.6895	59	0	R.GATYAFTGSHYWR.L (Ions score 53)
346 – 369	2448.3303	2447.3230	2447.1638	65	0	K.ELGSPPGISLETIDAAFSCPGSSR.L Carbamidomet

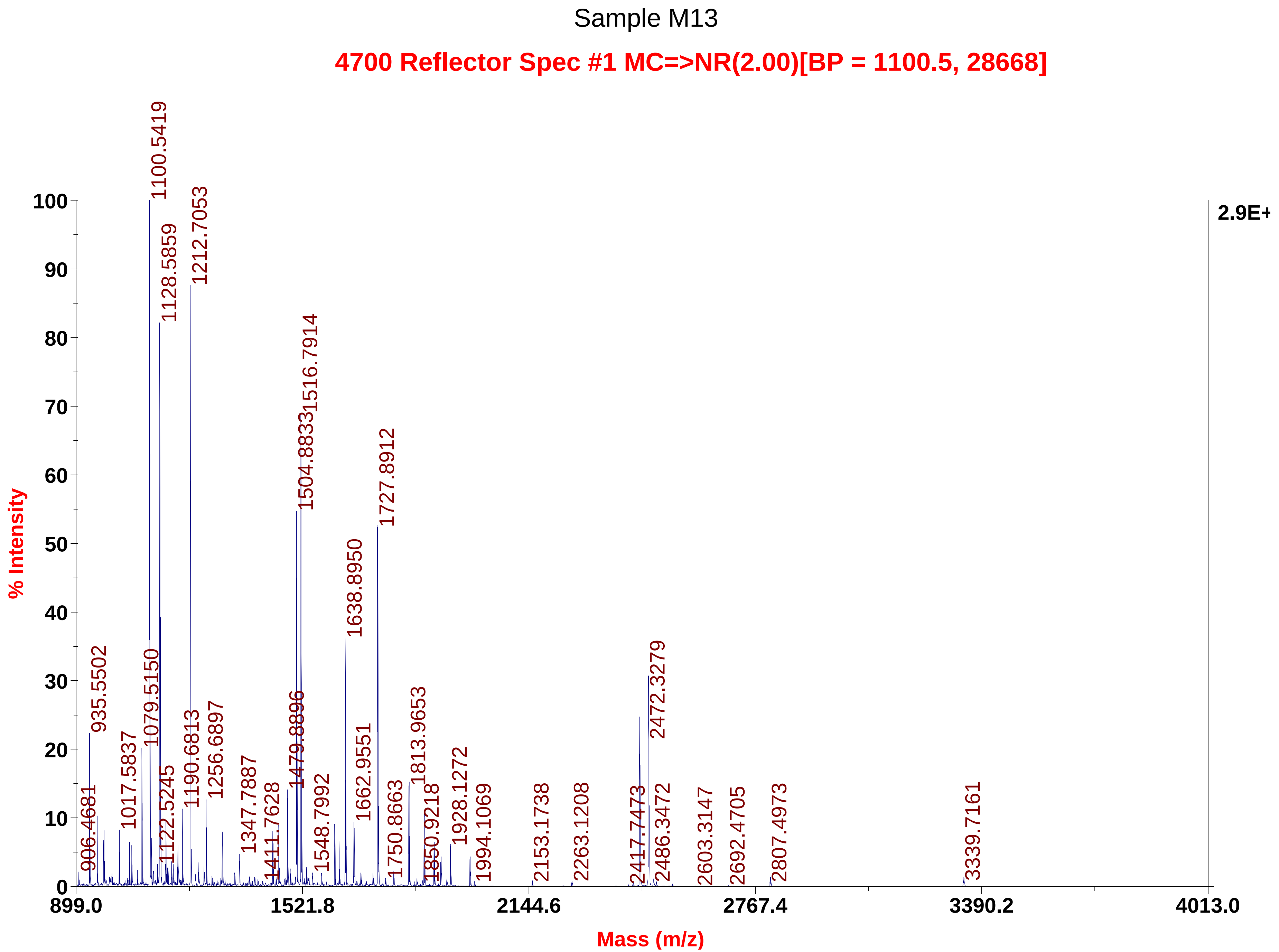

Sample M13
4700 Reflector Spec #1 MC=>NR(2.00)[BP = 1100.5, 28668]
% Intensity
Mass (m/z)
2.9E+
100
90
80
70
60
50
40
30
20
10
0
899.0
1521.8
2144.6
2767.4
3390.2
4013.0
1100.5419
1128.5859
1212.7053
1516.7914
1504.8833
1727.8912
1638.8950
2472.3279
935.5502
1079.5150
1479.8896
1813.9653
1017.5837
1122.5245
1190.6813
1256.6897
1347.7887
1411.7628
1548.7992
1662.9551
1750.8663
1850.9218
1928.1272
1994.1069
2153.1738
2263.1208
2417.7473
2486.3472
2603.3147
2692.4705
2807.4973
3339.7161
906.4681

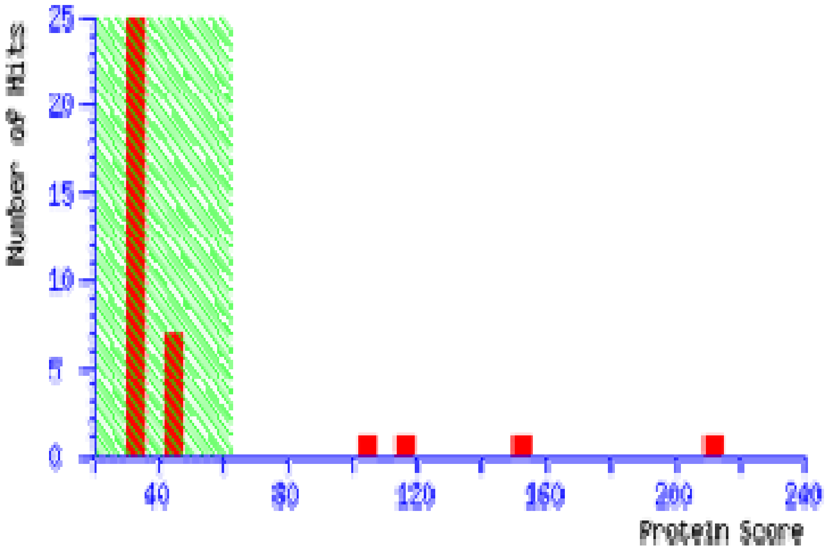

Concise Protein Summary Report

Format As | Concise Protein Summary | Help

Significance threshold p< 0.05 Max. number of hits AUTO

Re-Search All | Search Unmatched

1. **Mixture 1** **Total score**: 212 **Expect**: 5.8e-17 **Matches**: 70

Components (only one family member shown for each component):

Q3UKP2_MOUSE **Mass**: 51285 **Score**: 152 **Expect**: 5.8e-11 **Matches**: 31

14 days embryo liver cDNA, RIKEN full-length enriched library, clone:I530016F13 product:hemopexin, full inse

Q8C7C7_MOUSE **Mass**: 64961 **Score**: 114 **Expect**: 3.7e-07 **Matches**: 46

2 days neonate thymus thymic cells cDNA, RIKEN full-length enriched library, clone:C920028B14 product:albumi

Protein View

Match to: **Q3UKP2_MOUSE** Score: **152** Expect: **5.8e-11**
14 days embryo liver cDNA, RIKEN full-length enriched library, clone:I530016F13 product:hemopexin,
Found in search of pmf_M2_129990849012.txt

Nominal mass (M_r): **51285**; Calculated pI value: **7.92**
NCBI BLAST search of Q3UKP2_MOUSE against nr
Unformatted sequence string for pasting into other applications

Taxonomy: Mus musculus
Links to retrieve other entries containing this sequence from NCBI Entrez:
(no taxonomy information for this entry)

Variable modifications: Carbamidomethyl (C),Deamidated (NQ),Oxidation (M)
Cleavage by Trypsin: cuts C-term side of KR unless next residue is P
Number of mass values searched: **126**
Number of mass values matched: **31**
Sequence Coverage: **63%**

Matched peptides shown in **Bold Red**

```
  1 MARTAVALNI LVLLGLCWSL AVASPLPTAN GRVAEVENGT KPDSDVPEHC
 51 LDTWSFDAAT MDHNGTMLFF KGEFVWRGHS GTRELISARW KNPITSVDAA
101 FRGPDSVFLI KEDKVWVYPP EKKENGYPKL FQEEFPGIPY PPDAAVECHR
151 GECQSEGVLF FQGNRKWFWD FATRTQKERS WSTVGNCTAA LRWLERYYCF
201 QGNKFLRFNP VTGEVPPRYP LDARDYFVSC PGRGHGRPRN GTAHGNSTHP
251 MHSRCSPDPG LTALLSDHRG ATYAFTGSHY WRLDSSRDGW HSWPIAHHWP
301 QGPSTVDAAF SWDDKVYLIQ GTQVYVFLTK GGNNLVSGYP KRLEKELGSP
351 PGISLETIDA AFSCPGSSRL YVSSGRRLWW LDLKSGAQAT WTEVSWPHEK
401 VDGALCLDKS LGPNTCSSNG SSLYFIHGPN LYCYSSIDKL NAAKSLPQPQ
451 KVNSILGCSQ
```

Show predicted peptides also

Sort Peptides By ◉ Residue Number ○ Increasing Mass ○ Decreasing Mass

Start – End	Observed	Mr(expt)	Mr(calc)	ppm	Miss	Sequence
90 – 102	1504.8826	1503.8753	1503.7834	61	1	R.WKNPITSVDAAFR.G
92 – 102	1190.6791	1189.6718	1189.6091	53	0	K.NPITSVDAAFR.G
103 – 111	975.5937	974.5864	974.5437	44	0	R.GPDSVFLIK.E
103 – 114	1347.7883	1346.7810	1346.7082	54	1	R.GPDSVFLIKEDK.V
112 – 122	1389.7819	1388.7746	1388.6976	55	1	K.EDKVWVYPPEK.K
115 – 122	1017.5838	1016.5765	1016.5331	43	0	K.VWVYPPEK.K
115 – 123	1145.6945	1144.6872	1144.6281	52	1	K.VWVYPPEKK.E

Protein View

Match to: Q8C7C7_MOUSE Score: **114** Expect: **3.7e-07**
2 days neonate thymus thymic cells cDNA, RIKEN full-length enriched library, clone:C92002
Found in search of pmf_M2_129990849012.txt

Nominal mass (M_r): **64961**; Calculated pI value: **5.49**
NCBI BLAST search of Q8C7C7_MOUSE against nr
Unformatted sequence string for pasting into other applications

Taxonomy: Mus musculus
Links to retrieve other entries containing this sequence from NCBI Entrez:
(no taxonomy information for this entry)

Variable modifications: Carbamidomethyl (C),Deamidated (NQ),Oxidation (M)
Cleavage by Trypsin: cuts C-term side of KR unless next residue is P
Number of mass values searched: **126**
Number of mass values matched: **46**
Sequence Coverage: **69%**

Matched peptides shown in **Bold Red**

```
  1 NRYNDLGEQH FKGLVLIAFS QYLQKCSYDE HAKLVQEVTD FAKTCVADES
 51 AANCDKSLHT LFGDKLCAIP NLRENYGELA DCCTKQEPER NECFLQHKDD
101 NPSLPPFERP EAEAMCTSFK ENPTTFMGHY LHEVARRHPY FYAPELLYYA
151 EQYNEILTQC CAEADKESCL TPKLDGVKEK ALVSSVRQRM KCSSMQKFGE
201 RAFKAWAVAR LSQTFPNADF AEITKLATDL TKVNKECCHG DLLECADDRA
251 ELAKYMCENQ ATISSKLQTC CDKPLLKKAH CLSEVEHDTM PADLPAIAAD
301 FVEDQEVCKN YAEAKDVFLG TFLYEYSRRH PDYSVSLLLR LAKKYEATLE
351 KCCAEANPPA CYGTVLAEFQ PLVEEPKNLV KTNCDLYEKL GEYGFQNAIL
401 VRYTQKAPQV STPTLVEAAR NLGRVGTKCC TLPEDQRLPC VEDYLSAILN
451 RVCLLHEKTP VSEHVTKCCS GSLVERRPCF SALTVDETYV PKEFKAETFT
501 FHSDICTLPE KEKQIKKQTA LAELVKHKPK ATAEQLKTVM DDFAQFLDTC
551 CKAADKDTCF STEGPNLVTR CKDALA
```

Show predicted peptides also

Sort Peptides By ◉ Residue Number ○ Increasing Mass ○ Decreasing Mass

Start - End	Observed	Mr(expt)	Mr(calc)	ppm	Miss	Sequence
1 - 12	1520.8362	1519.8289	1519.7168	74	1	-.NRYNDLGEQHFK.G
13 - 25	1479.8887	1478.8814	1478.8497	21	0	K.GLVLIAFSQYLQK.C
13 - 33	2415.3196	2414.3123	2414.1828	54	1	K.GLVLIAFSQYLQKCSYDEHAK
13 - 33	2470.3232	2469.3159	2469.2362	32	1	K.GLVLIAFSQYLQKCSYDEHAK
13 - 33	2472.3269	2471.3196	2471.2042	47	1	K.GLVLIAFSQYLQKCSYDEHAK
34 - 43	1149.6681	1148.6608	1148.6077	46	0	K.LVQEVTDFAK.T
34 - 56	2457.3489	2456.3416	2456.1199	90	1	K.LVQEVTDFAKTCVADESAANC
57 - 65	1017.5838	1016.5765	1016.5291	47	0	K.SLHTLFGDK.L

M13

4700 MS/MS Precursor 1212.7 Spec #1 MC[BP = 352.2, 4991]

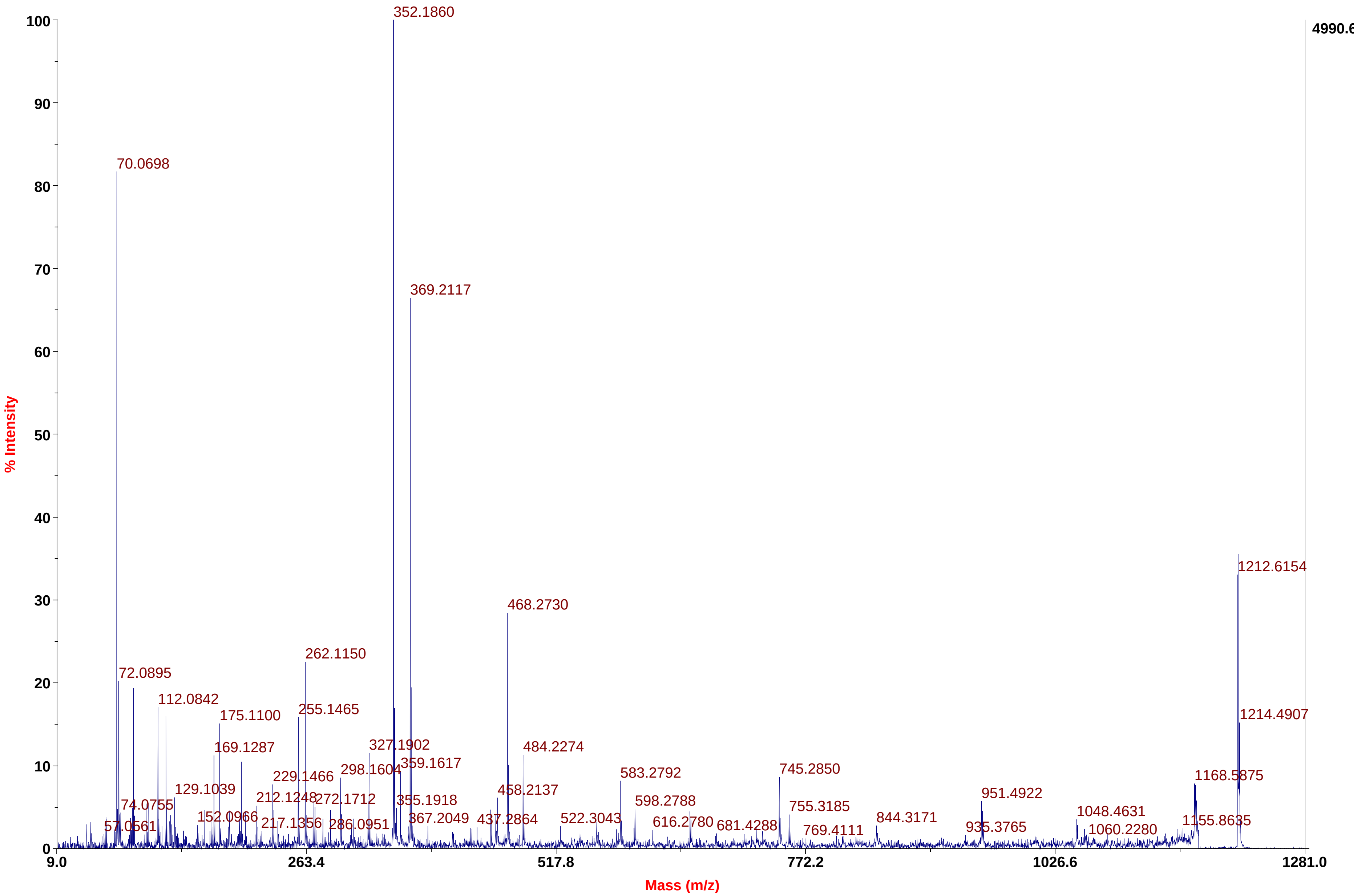

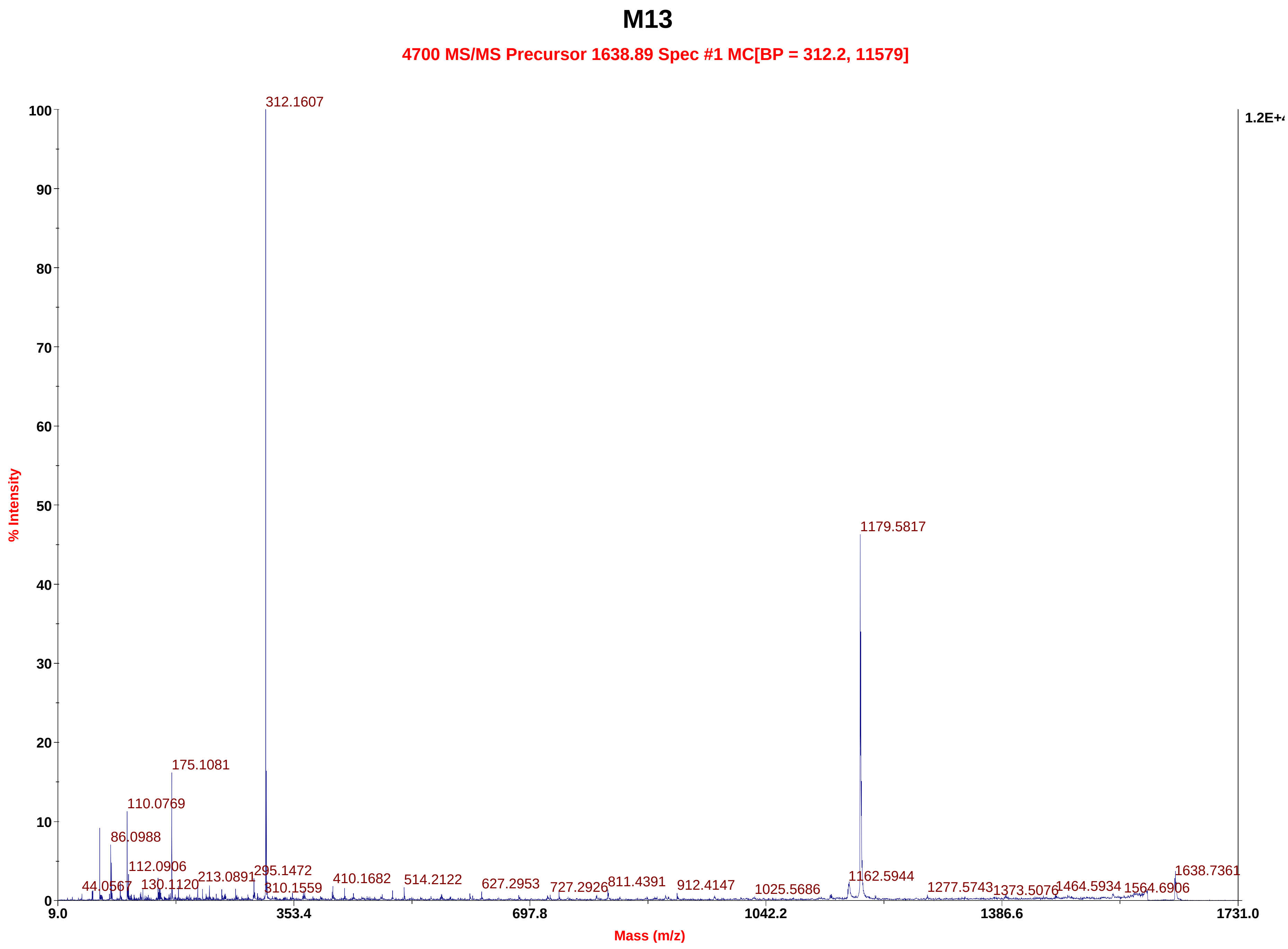
M13
4700 MS/MS Precursor 1638.89 Spec #1 MC[BP = 312.2, 11579]
% Intensity
Mass (m/z)
312.1607
1179.5817
175.1081
110.0769
86.0988
112.0906
44.0567
130.1120
213.0891
295.1472
310.1559
410.1682
514.2122
627.2953
727.2926
811.4391
912.4147
1025.5686
1162.5944
1277.5743
1373.5076
1464.5934
1564.6906
1638.7361
9.0
353.4
697.8
1042.2
1386.6
1731.0

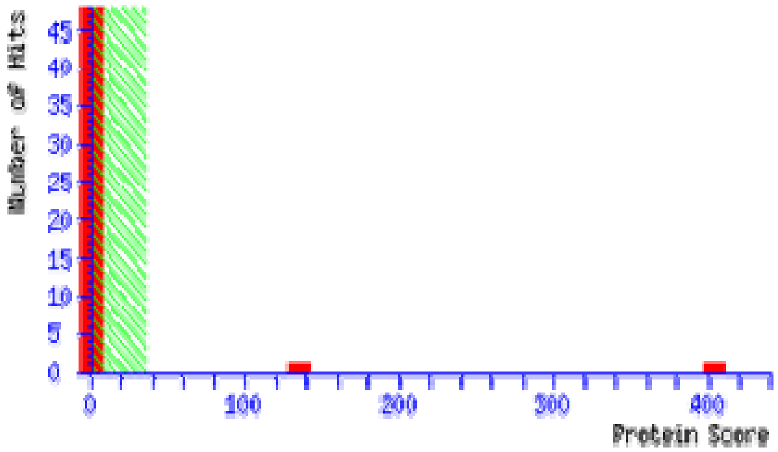

Peptide Summary Report

Format As | Peptide Summary | Help

Significance threshold p< 0.05 Max. number of hits AUTO

Standard scoring ○ MudPIT scoring ● Ions score or expect cut-off 0 Show sub-sets 0

Show pop-ups ● Suppress pop-ups ○ Sort unassigned Decreasing Score Require bold red ☐

Select All | Select None | Search Selected | ☐ **Error tolerant**

1. Q3UKP2_MOUSE **Mass:** 51285 **Score:** 404 **Matches:** 15(9) **Sequences:** 15(9) **emPAI:** 2.80

14 days embryo liver cDNA, RIKEN full-length enriched library, clone:I530016F13 product:hemopexin, full insert s

☐ Check to include this hit in error tolerant search

	Query	Observed	Mr(expt)	Mr(calc)	ppm	Miss	Score	Expect	Rank	Unique	Peptide
☑	2	1079.5145	1078.5072	1078.4542	49.2	0	49	0.0041	1	U	R.YYCFQGNK.F + Carbamidomethyl (C)
☑	3	1100.5393	1099.5320	1099.4757	51.2	0	36	0.067	1	U	R.DYFVSCPGR.G + Carbamidomethyl (C)
☑	4	1128.5852	1127.5779	1127.5189	52.4	0	21	2.5	1	U	K.WFWDFATR.T

Protein View

Match to: **Q3UKP2_MOUSE** Score: **404**
14 days embryo liver cDNA, RIKEN full-length enriched library, clone:I530016F13 product:hemopexin, full insert sequen
Found in search of ppw_M2_129990855812.txt

Nominal mass (M_r): **51285**; Calculated pI value: **7.92**
NCBI BLAST search of Q3UKP2_MOUSE against nr
Unformatted sequence string for pasting into other applications

Taxonomy: Mus musculus
Links to retrieve other entries containing this sequence from NCBI Entrez:
(no taxonomy information for this entry)

Variable modifications: Carbamidomethyl (C),Deamidated (NQ),Oxidation (M)
Cleavage by Trypsin: cuts C-term side of KR unless next residue is P
Sequence Coverage: **35%**

Matched peptides shown in **Bold Red**

```
  1 MARTAVALNI LVLLGLCWSL AVASPLPTAN GRVAEVENGT KPDSDVPEHC
 51 LDTWSFDAAT MDHNGTMLFF KGEFVWRGHS GTRELISARW KNPITSVDAA
101 FRGPDSVFLI KEDKVWVYPP EKKENGYPKL FQEEFPGIPY PPDAAVECHR
151 GECQSEGVLF FQGNRKWFWD FATRTQKERS WSTVGNCTAA LRWLERYYCF
201 QGNKFLRFNP VTGEVPPRYP LDARDYFVSC PGRGHGRPRN GTAHGNSTHP
251 MHSRCSPDPG LTALLSDHRG ATYAFTGSHY WRLDSSRDGW HSWPIAHHWP
301 QGPSTVDAAF SWDDKVYLIQ GTQVYVFLTK GGNNLVSGYP KRLEKELGSP
351 PGISLETIDA AFSCPGSSRL YVSSGRRLWW LDLKSGAQAT WTEVSWPHEK
401 VDGALCLDKS LGPNTCSSNG SSLYFIHGPN LYCYSSIDKL NAAKSLPQPQ
451 KVNSILGCSQ
```

Show predicted peptides also

Sort Peptides By ◉ Residue Number ○ Increasing Mass ○ Decreasing Mass

Start – End	Observed	Mr(expt)	Mr(calc)	ppm	Miss	Sequence
90 – 102	1504.8826	1503.8753	1503.7834	61	1	R.WKNPITSVDAAFR.G (Ions score 69)
92 – 102	1190.6791	1189.6718	1189.6091	53	0	K.NPITSVDAAFR.G (Ions score 44)
115 – 123	1145.6945	1144.6872	1144.6281	52	1	K.VWVYPPEKK.E (Ions score 6)
130 – 150	2472.3269	2471.3196	2471.1580	65	0	K.LFQEEFPGIPYPPDAAVECHR.G Carbamidomethyl (C) (Ion
151 – 165	1727.8903	1726.8830	1726.7733	64	0	R.GECQSEGVLFFQGNR.K Carbamidomethyl (C) (Ions scor
151 – 166	1855.9941	1854.9868	1854.8683	64	1	R.GECQSEGVLFFQGNRK.W Carbamidomethyl (C) (Ions sco
166 – 174	1256.6886	1255.6813	1255.6138	54	1	R.KWFWDFATR.T (Ions score 19)
167 – 174	1128.5852	1127.5779	1127.5189	52	0	K.WFWDFATR.T (Ions score 21)
197 – 204	1079.5145	1078.5072	1078.4542	49	0	R.YYCFQGNK.F Carbamidomethyl (C) (Ions score 49)
208 – 218	1212.7028	1211.6955	1211.6299	54	0	R.FNPVTGEVPPR.Y (Ions score 39)

Sample M14

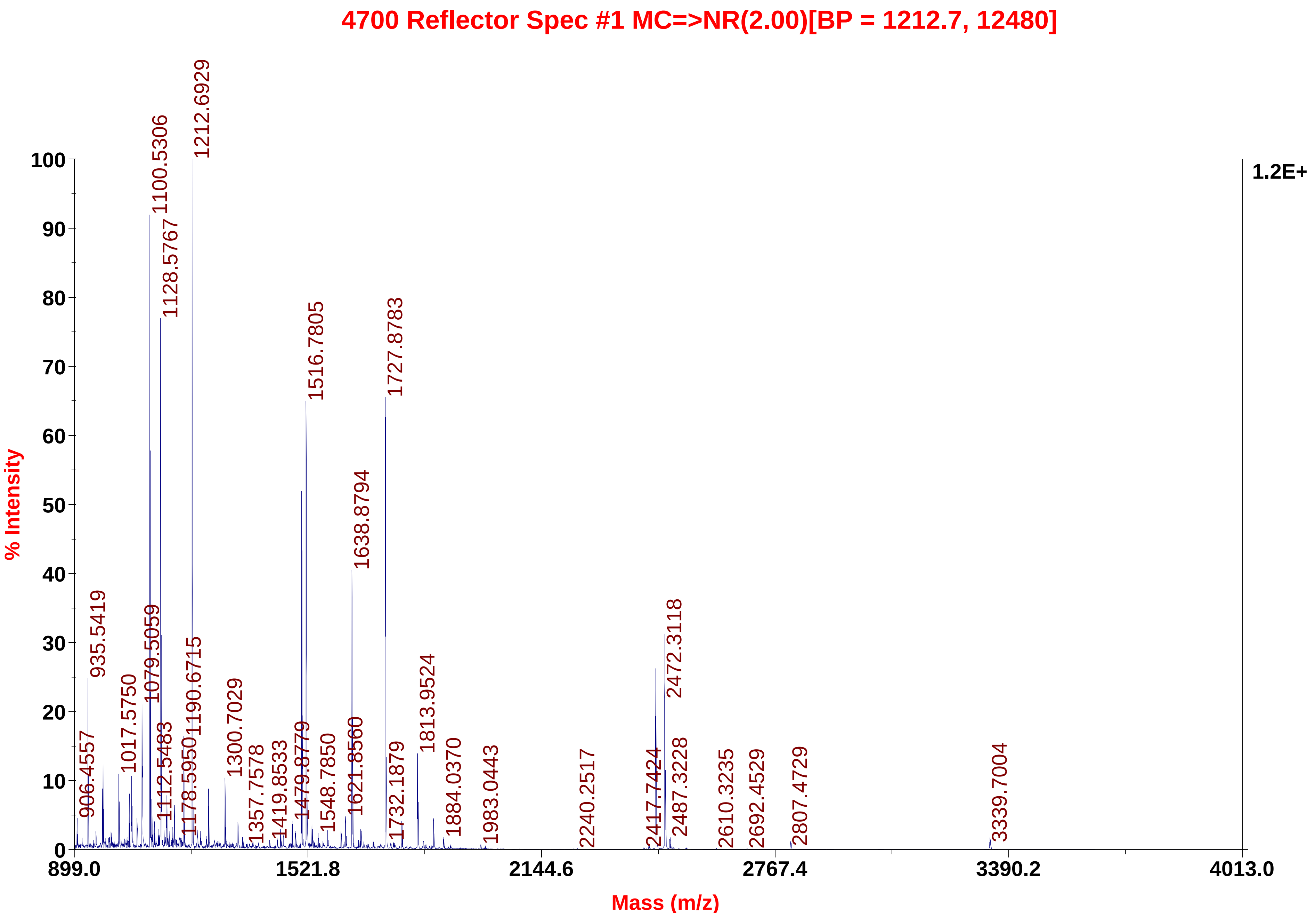

4700 Reflector Spec #1 MC=>NR(2.00)[BP = 1212.7, 12480]
% Intensity
Mass (m/z)
1.2E+
100
90
80
70
60
50
40
30
20
10
0
899.0
1521.8
2144.6
2767.4
3390.2
4013.0
906.4557
935.5419
1017.5750
1079.5059
1100.5306
1112.5483
1128.5767
1178.5950
1190.6715
1212.6929
1300.7029
1357.7578
1419.8533
1479.8779
1516.7805
1548.7850
1621.8560
1638.8794
1727.8783
1732.1879
1813.9524
1884.0370
1983.0443
2240.2517
2417.7424
2472.3118
2487.3228
2610.3235
2692.4529
2807.4729
3339.7004

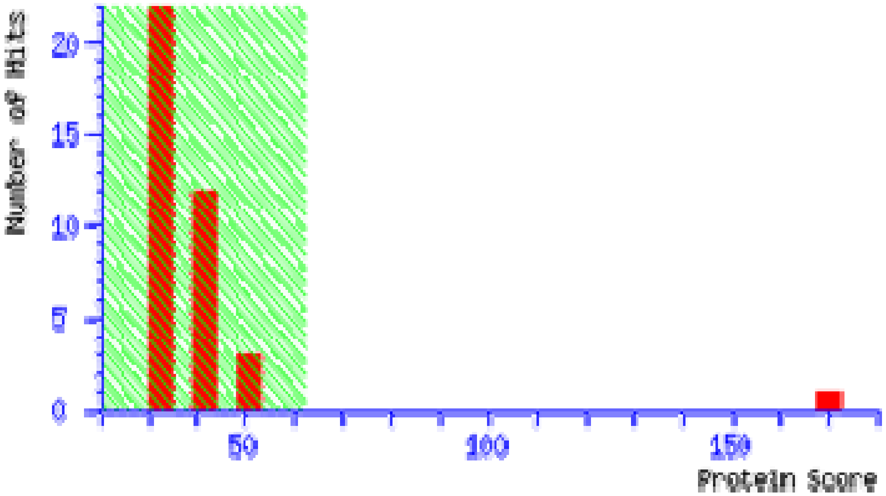

Concise Protein Summary Report

Format As | Concise Protein Summary | Help

Significance threshold p< 0.05 Max. number of hits AUTO

Re-Search All | Search Unmatched

1. Q3UKP2_MOUSE **Mass**: 51285 **Score**: 170 **Expect**: 9.2e-13 **Matches**: 25
14 days embryo liver cDNA, RIKEN full-length enriched library, clone:I530016F13 product:hemopexi
AAH11246 **Mass**: 51308 **Score**: 170 **Expect**: 9.2e-13 **Matches**: 25
BC011246 NID: - Mus musculus
AAB49490 **Mass**: 50514 **Score**: 157 **Expect**: 1.8e-11 **Matches**: 24
MMU89889 NID: - Mus musculus
Q8K1U6_MOUSE **Mass**: 31027 **Score**: 110 **Expect**: 9.2e-07 **Matches**: 15
Hemopexin (Fragment).- Mus musculus (Mouse).

Protein View

Match to: **Q3UKP2_MOUSE** Score: **170** Expect: **9.2e-13**
14 days embryo liver cDNA, RIKEN full-length enriched library, clone:I530016F13 product:hemopexin, f
Found in search of pmf_N2_129990849013.txt

Nominal mass (M_r): **51285**; Calculated pI value: **7.92**
NCBI BLAST search of Q3UKP2_MOUSE against nr
Unformatted sequence string for pasting into other applications

Taxonomy: Mus musculus
Links to retrieve other entries containing this sequence from NCBI Entrez:
(no taxonomy information for this entry)

Variable modifications: Carbamidomethyl (C),Deamidated (NQ),Oxidation (M)
Cleavage by Trypsin: cuts C-term side of KR unless next residue is P
Number of mass values searched: **108**
Number of mass values matched: **25**
Sequence Coverage: **51%**

Matched peptides shown in **Bold Red**

```
  1 MARTAVALNI LVLLGLCWSL AVASPLPTAN GRVAEVENGT KPDSDVPEHC
 51 LDTWSFDAAT MDHNGTMLFF KGEFVWRGHS GTRELISARW KNPITSVDAA
101 FRGPDSVFLI KEDKVWVYPP EKKENGYPKL FQEEFPGIPY PPDAAVECHR
151 GECQSEGVLF FQGNRKWFWD FATRTQKERS WSTVGNCTAA LRWLERYYCF
201 QGNKFLRFNP VTGEVPPRYP LDARDYFVSC PGRGHGRPRN GTAHGNSTHP
251 MHSRCSPDPG LTALLSDHRG ATYAFTGSHY WRLDSSRDGW HSWPIAHHWP
301 QGPSTVDAAF SWDDKVYLIQ GTQVYVFLTK GGNNLVSGYP KRLEKELGSP
351 PGISLETIDA AFSCPGSSRL YVSSGRRLWW LDLKSGAQAT WTEVSWPHEK
401 VDGALCLDKS LGPNTCSSNG SSLYFIHGPN LYCYSSIDKL NAAKSLPQPQ
451 KVNSILGCSQ
```

Show predicted peptides also

Sort Peptides By ◉ Residue Number ○ Increasing Mass ○ Decreasing Mass

Start – End	Observed	Mr(expt)	Mr(calc)	ppm	Miss	Sequence	
90 – 102	1504.8705	1503.8632	1503.7834	53	1	R.WKNPITSVDAAFR.G	
92 – 102	1190.6692	1189.6619	1189.6091	44	0	K.NPITSVDAAFR.G	
103 – 111	975.5863	974.5790	974.5437	36	0	R.GPDSVFLIK.E	
103 – 114	1347.7714	1346.7641	1346.7082	42	1	R.GPDSVFLIKEDK.V	
115 – 122	1017.5754	1016.5681	1016.5331	34	0	K.VWVYPPEK.K	
115 – 123	1145.6865	1144.6792	1144.6281	45	1	K.VWVYPPEKK.E	
130 – 150	2472.3081	2471.3008	2471.1580	58	0	K.LFQEEFPGIPYPPDAAVECHR.G	Carbami
151 – 165	1670.8501	1669.8428	1669.7519	54	0	R.GECQSEGVLFFQGNR.K	
151 – 165	1727.8776	1726.8703	1726.7733	56	0	R.GECQSEGVLFFQGNR.K	Carbamidometh
151 – 166	1855.9836	1854.9763	1854.8683	58	1	R.GECQSEGVLFFQGNRK.W	Carbamidomet
166 – 174	1256.6799	1255.6726	1255.6138	47	1	R.KWFWDFATR.T	

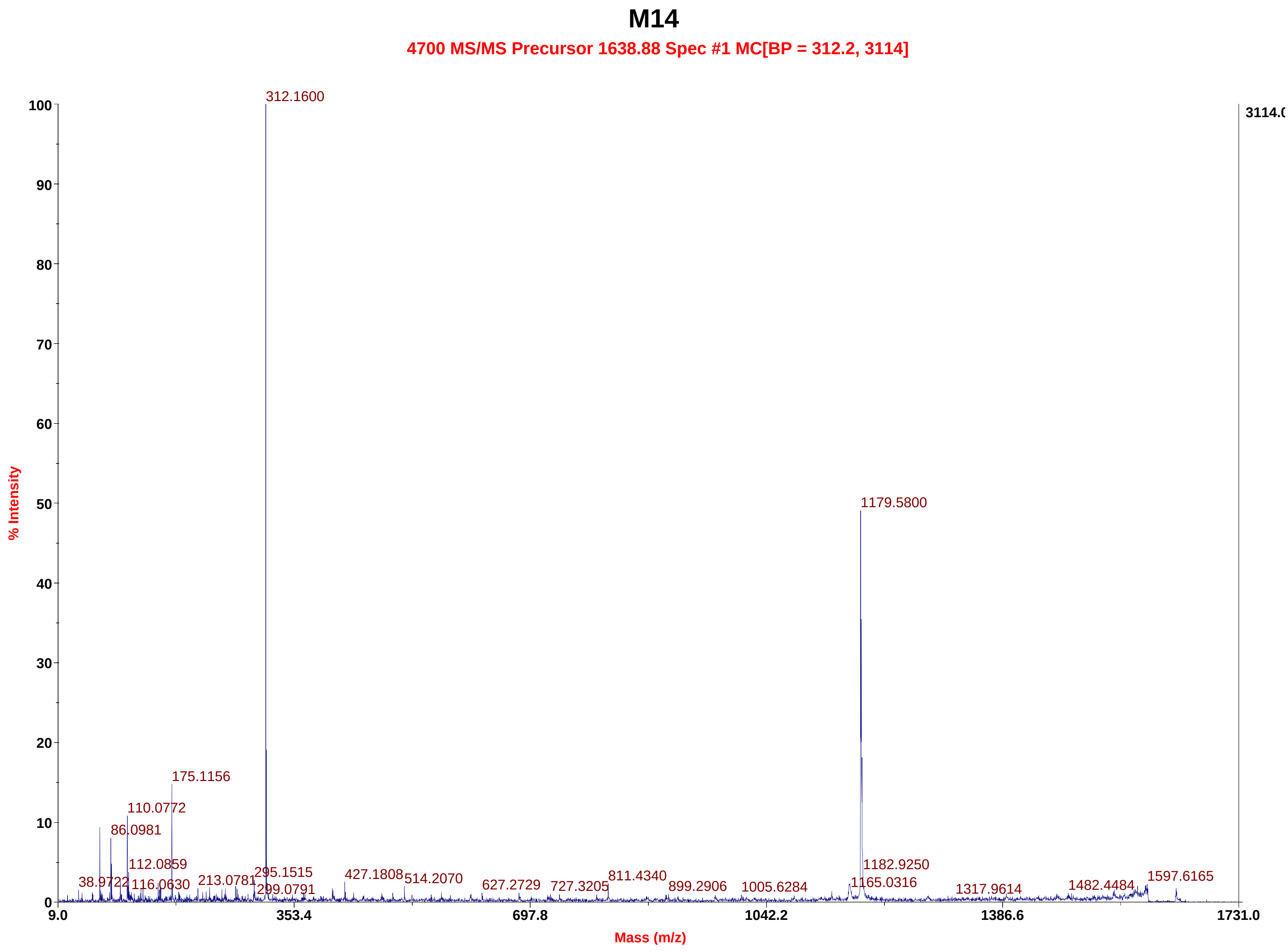

M14
4700 MS/MS Precursor 1638.88 Spec #1 MC[BP = 312.2, 3114]
% Intensity
Mass (m/z)
312.1600
1179.5800
175.1156
110.0772
86.0981
112.0859
38.9722
116.0630
213.0781
295.1515
299.0791
427.1808
514.2070
627.2729
727.3205
811.4340
899.2906
1005.6284
1182.9250
1165.0316
1317.9614
1482.4484
1597.6165
9.0
353.4
697.8
1042.2
1386.6
1731.0

M14

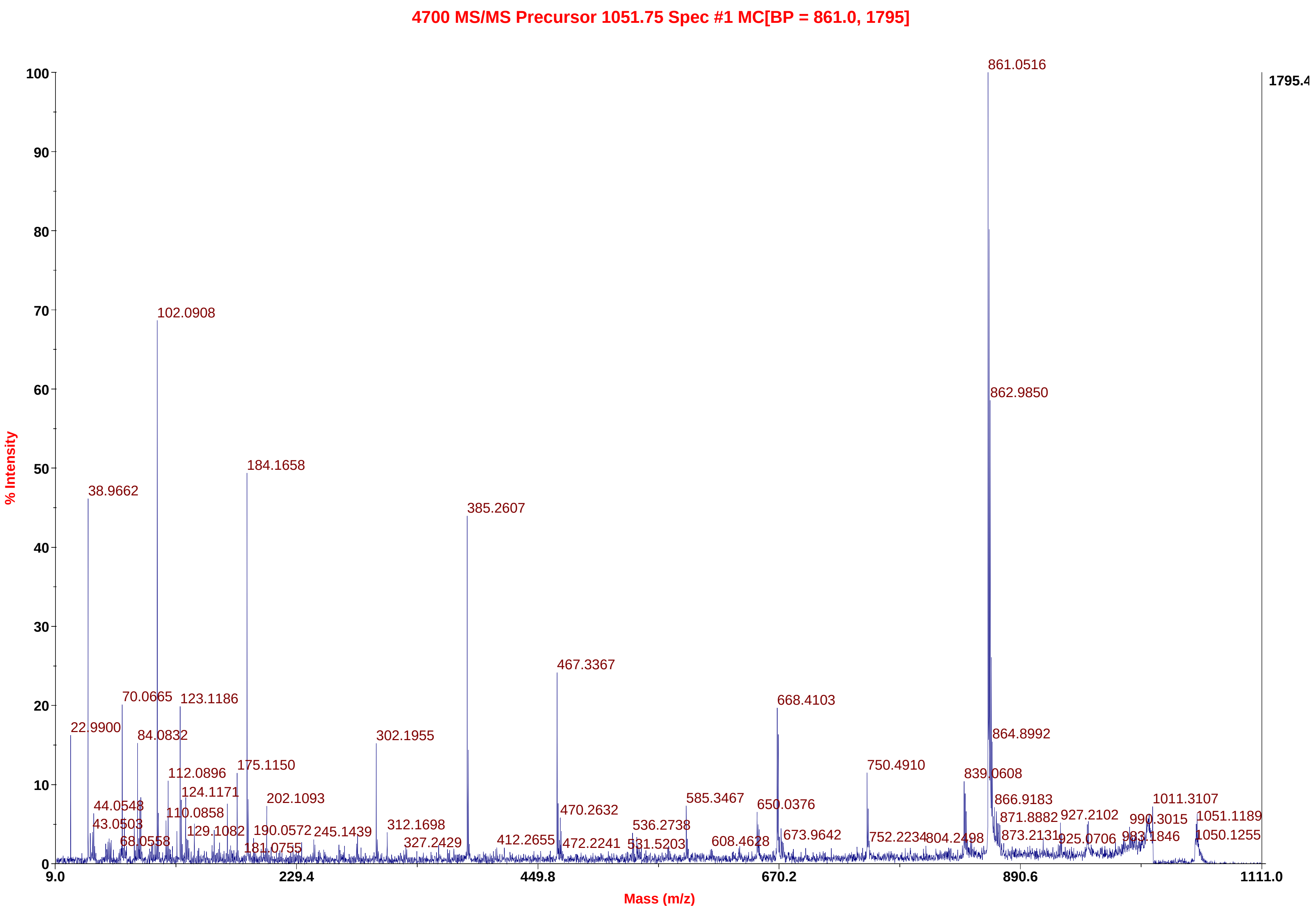
4700 MS/MS Precursor 1051.75 Spec #1 MC[BP = 861.0, 1795]
% Intensity
Mass (m/z)
861.0516
1795.4
102.0908
862.9850
184.1658
38.9662
385.2607
467.3367
70.0665
123.1186
668.4103
22.9900
84.0832
302.1955
864.8992
112.0896
175.1150
750.4910
839.0608
124.1171
202.1093
585.3467
1011.3107
44.0548
866.9183
650.0376
110.0858
470.2632
927.2102
871.8882
990.3015
1051.1189
43.0503
312.1698
536.2738
129.1082
190.0572
245.1439
673.9642
873.2131
983.1846
1050.1255
68.0558
327.2429
412.2655
752.2234
804.2498
925.0706
472.2241
608.4628
531.5203
181.0755

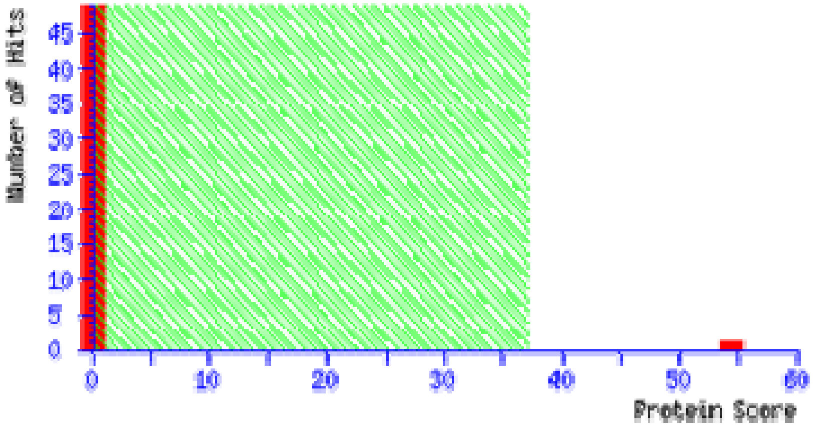

Peptide Summary Report

Format As | Peptide Summary | Help

Significance threshold p< 0.05 | Max. number of hits AUTO

Standard scoring ○ MudPIT scoring ● | Ions score or expect cut-off 0 | Show sub-sets 0

Show pop-ups ● Suppress pop-ups ○ | Sort unassigned Decreasing Score | Require bold red ☐

Select All | Select None | Search Selected | ☐ **Error tolerant**

1. Q3UKP2_MOUSE **Mass:** 51285 **Score:** 55 **Matches:** 15(1) **Sequences:** 15(1) **emPAI:** 0.56
14 days embryo liver cDNA, RIKEN full-length enriched library, clone:I530016F13 product:hemopexin, full inser
☐ Check to include this hit in error tolerant search

	Query	Observed	Mr(expt)	Mr(calc)	ppm	Miss	Score	Expect	Rank	Unique	Peptide
☑	2	975.5863	974.5790	974.5437	36.3	0	34	0.095	1	U	R.GPDSVFLIK.E
☑	3	1017.5754	1016.5681	1016.5331	34.4	0	18	4.7	1	U	K.VWVYPPEK.K
☑	6	1079.5037	1078.4964	1078.4542	39.1	0	25	1	1	U	R.YYCFQGNK.F + Carbamidomethyl
☑	7	1100.5300	1099.5227	1099.4757	42.8	0	27	0.59	1	U	R.DYFVSCPGR.G + Carbamidomethyl
☑	8	1128.5742	1127.5669	1127.5189	42.6	0	4	1.2e+02	1	U	K.WFWDFATR.T

Protein View

Match to: **Q3UKP2_MOUSE** Score: **55**
14 days embryo liver cDNA, RIKEN full-length enriched library, clone:I530016F13 product:hemopexin, full i
Found in search of ppw_N2_129990855913.txt

Nominal mass (M_r): **51285**; Calculated pI value: **7.92**
NCBI BLAST search of Q3UKP2_MOUSE against nr
Unformatted sequence string for pasting into other applications

Taxonomy: Mus musculus
Links to retrieve other entries containing this sequence from NCBI Entrez:
(no taxonomy information for this entry)

Variable modifications: Carbamidomethyl (C),Deamidated (NQ),Oxidation (M)
Cleavage by Trypsin: cuts C-term side of KR unless next residue is P
Sequence Coverage: **34%**

Matched peptides shown in **Bold Red**

```
  1 MARTAVALNI LVLLGLCWSL AVASPLPTAN GRVAEVENGT KPDSDVPEHC
 51 LDTWSFDAAT MDHNGTMLFF KGEFVWRGHS GTRELISARW KNPITSVDAA
101 FRGPDSVFLI KEDKVWVYPP EKKENGYPKL FQEEFPGIPY PPDAAVECHR
151 GECQSEGVLF FQGNRKWFWD FATRTQKERS WSTVGNCTAA LRWLERYYCF
201 QGNKFLRFNP VTGEVPPRYP LDARDYFVSC PGRGHGRPRN GTAHGNSTHP
251 MHSRCSPDPG LTALLSDHRG ATYAFTGSHY WRLDSSRDGW HSWPIAHHWP
301 QGPSTVDAAF SWDDKVYLIQ GTQVYVFLTK GGNNLVSGYP KRLEKELGSP
351 PGISLETIDA AFSCPGSSRL YVSSGRRLWW LDLKSGAQAT WTEVSWPHEK
401 VDGALCLDKS LGPNTCSSNG SSLYFIHGPN LYCYSSIDKL NAAKSLPQPQ
451 KVNSILGCSQ
```

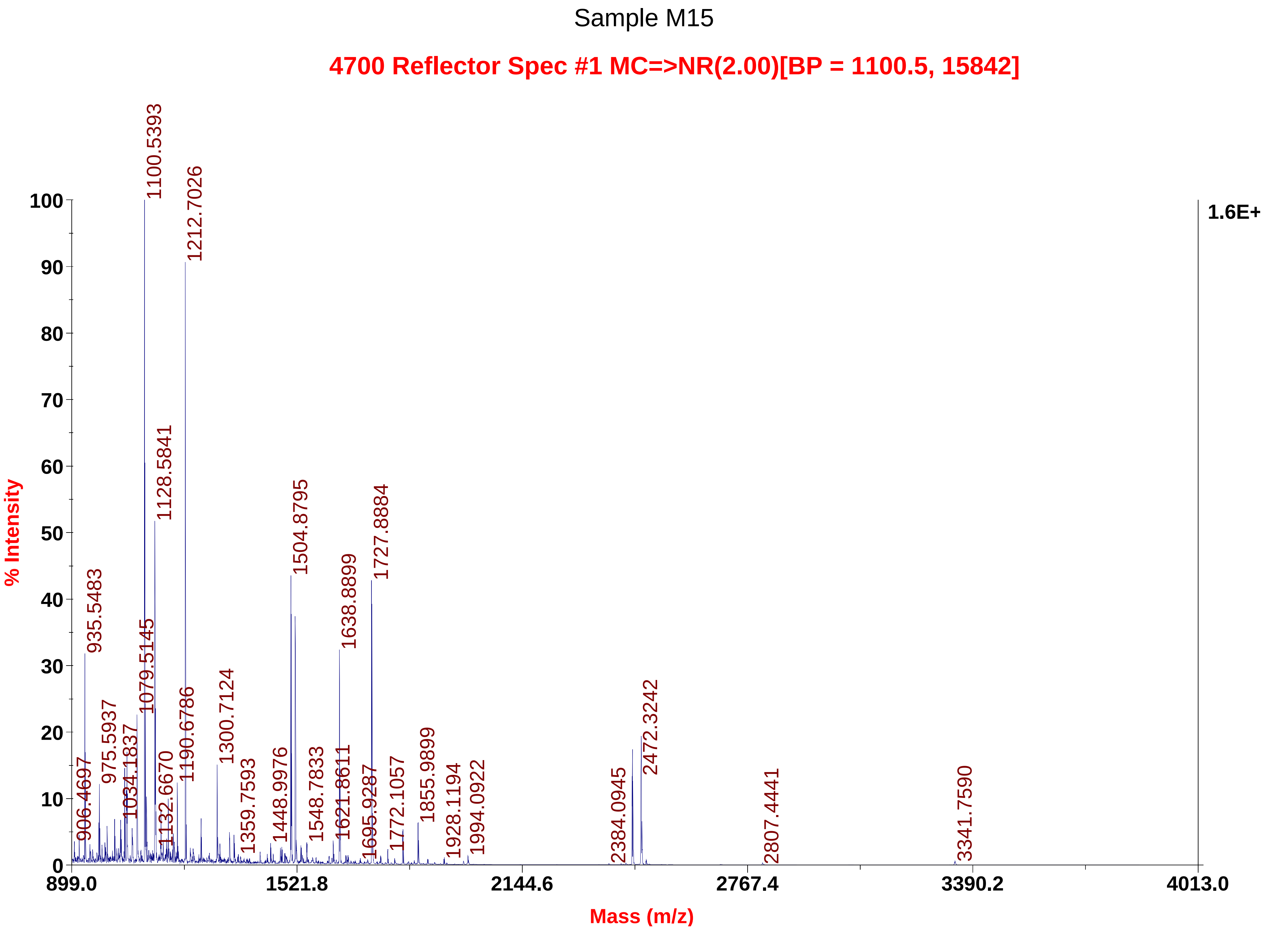

Sample M15
4700 Reflector Spec #1 MC=>NR(2.00)[BP = 1100.5, 15842]
% Intensity
Mass (m/z)
1.6E+
100
90
80
70
60
50
40
30
20
10
0
899.0
1521.8
2144.6
2767.4
3390.2
4013.0
1100.5393
1212.7026
1128.5841
1504.8795
1727.8884
1638.8899
935.5483
1079.5145
1300.7124
975.5937
1034.1837
1190.6786
1132.6670
906.4697
1359.7593
1448.9976
1548.7833
1621.8611
1695.9287
1772.1057
1855.9899
1928.1194
1994.0922
2384.0945
2472.3242
2807.4441
3341.7590

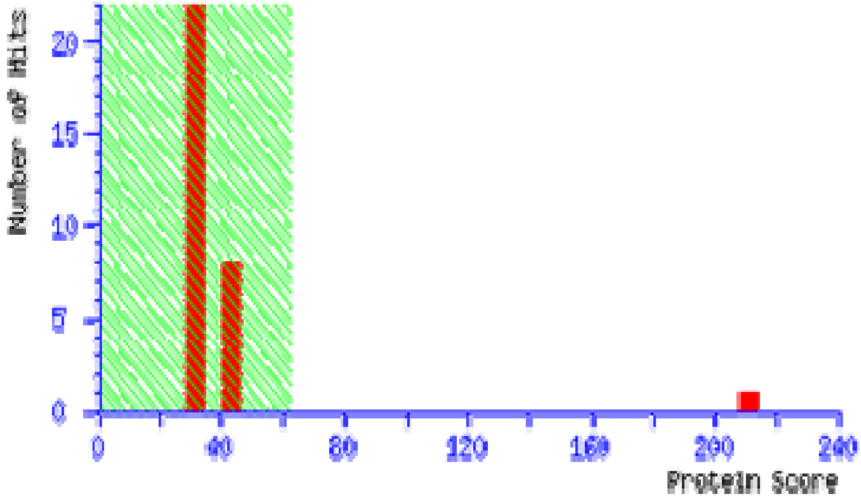

Concise Protein Summary Report

Format As | Concise Protein Summary | Help

Significance threshold p< 0.05 Max. number of hits AUTO

Re-Search All | Search Unmatched

1. Q3UKP2_MOUSE **Mass:** 51285 **Score:** 211 **Expect:** 7.3e-17 **Matches:** 29
14 days embryo liver cDNA, RIKEN full-length enriched library, clone:I530016F13 product:hemopexin, fu
AAH11246 **Mass:** 51308 **Score:** 211 **Expect:** 7.3e-17 **Matches:** 29
BC011246 NID: - Mus musculus
AAB49490 **Mass:** 50514 **Score:** 196 **Expect:** 2.3e-15 **Matches:** 27
MMU89889 NID: - Mus musculus
Q8K1U6_MOUSE **Mass:** 31027 **Score:** 134 **Expect:** 3.7e-09 **Matches:** 17
Hemopexin (Fragment).- Mus musculus (Mouse).

Protein View

Match to: **Q3UKP2_MOUSE** Score: **211** Expect: **7.3e-17**
14 days embryo liver cDNA, RIKEN full-length enriched library, clone:I530016F13 product:hemopexin
Found in search of pmf_o2_129990849114.txt

Nominal mass (M_r): **51285**; Calculated pI value: **7.92**
NCBI BLAST search of Q3UKP2_MOUSE against nr
Unformatted sequence string for pasting into other applications

Taxonomy: Mus musculus
Links to retrieve other entries containing this sequence from NCBI Entrez:
(no taxonomy information for this entry)

Variable modifications: Carbamidomethyl (C),Deamidated (NQ),Oxidation (M)
Cleavage by Trypsin: cuts C-term side of KR unless next residue is P
Number of mass values searched: **111**
Number of mass values matched: **29**
Sequence Coverage: **58%**

Matched peptides shown in **Bold Red**

```
  1 MARTAVALNI LVLLGLCWSL AVASPLPTAN GRVAEVENGT KPDSDVPEHC
 51 LDTWSFDAAT MDHNGTMLFF KGEFVWRGHS GTRELISARW KNPITSVDAA
101 FRGPDSVFLI KEDKVWVYPP EKKENGYPKL FQEEFPGIPY PPDAAVECHR
151 GECQSEGVLF FQGNRKWFWD FATRTQKERS WSTVGNCTAA LRWLERYYCF
201 QGNKFLRFNP VTGEVPPRYP LDARDYFVSC PGRGHGRPRN GTAHGNSTHP
251 MHSRCSPDPG LTALLSDHRG ATYAFTGSHY WRLDSSRDGW HSWPIAHHWP
301 QGPSTVDAAF SWDDKVYLIQ GTQVYVFLTK GGNNLVSGYP KRLEKELGSP
351 PGISLETIDA AFSCPGSSRL YVSSGRRLWW LDLKSGAQAT WTEVSWPHEK
401 VDGALCLDKS LGPNTCSSNG SSLYFIHGPN LYCYSSIDKL NAAKSLPQPQ
451 KVNSILGCSQ
```

Show predicted peptides also

Sort Peptides By ◉ Residue Number ○ Increasing Mass ○ Decreasing Mass

Start - End	Observed	Mr(expt)	Mr(calc)	ppm	Miss	Sequence
90 - 102	1504.8770	1503.8697	1503.7834	57	1	R.WKNPITSVDAAFR.G
92 - 102	1190.6782	1189.6709	1189.6091	52	0	K.NPITSVDAAFR.G
103 - 111	975.5938	974.5865	974.5437	44	0	R.GPDSVFLIK.E
103 - 114	1347.7842	1346.7769	1346.7082	51	1	R.GPDSVFLIKEDK.V
115 - 122	1017.5829	1016.5756	1016.5331	42	0	K.VWVYPPEK.K
115 - 123	1145.6919	1144.6846	1144.6281	49	1	K.VWVYPPEKK.E
130 - 150	2472.3220	2471.3147	2471.1580	63	0	K.LFQEEFPGIPYPPDAAVECHR.G Carb

M15

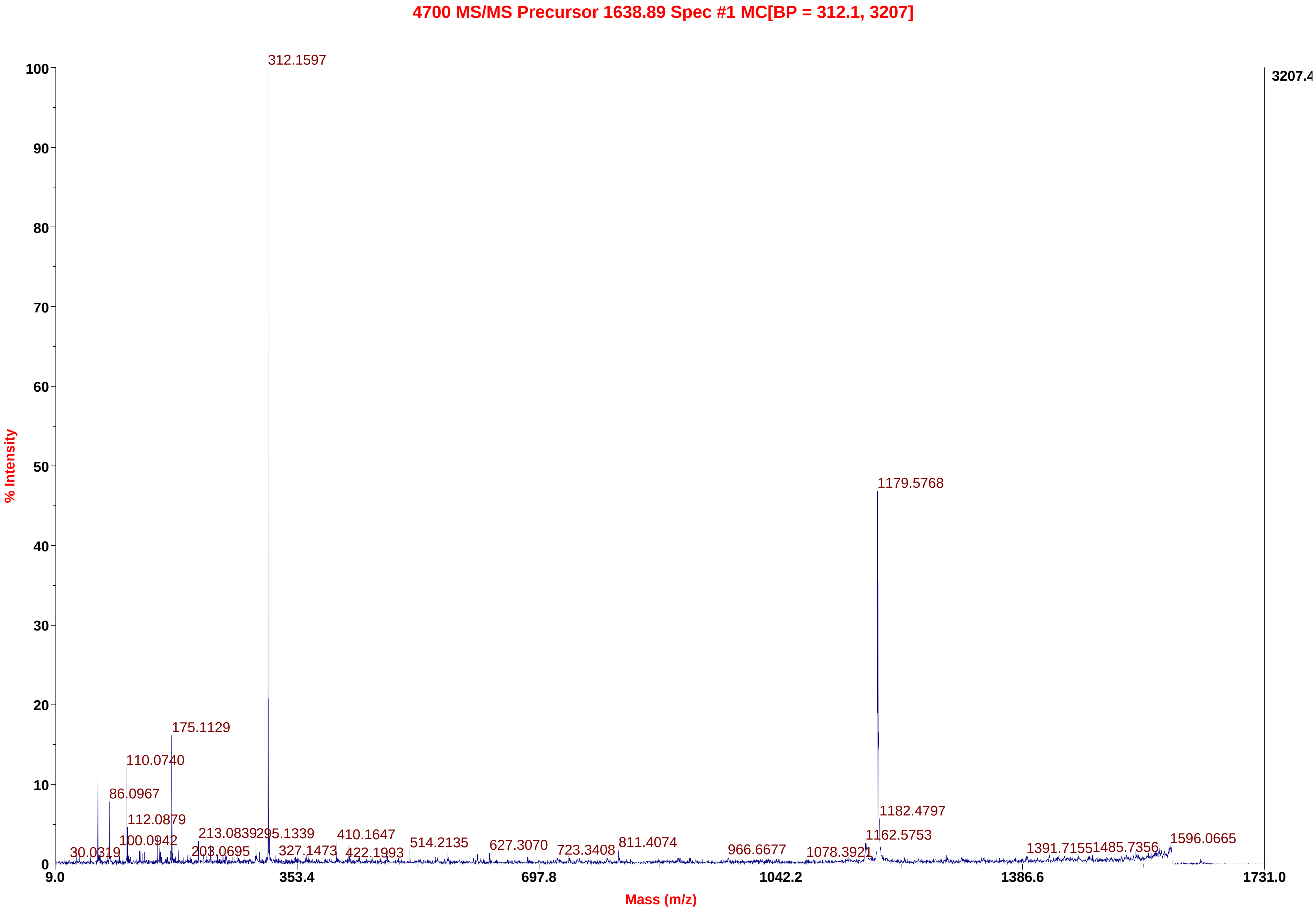
4700 MS/MS Precursor 1638.89 Spec #1 MC[BP = 312.1, 3207]
% Intensity
Mass (m/z)
3207.4
312.1597
1179.5768
175.1129
110.0740
86.0967
112.0879
100.0942
30.0319
213.0839
203.0695
295.1339
327.1473
410.1647
422.1993
514.2135
627.3070
723.3408
811.4074
966.6677
1078.3921
1162.5753
1182.4797
1391.7155
1485.7356
1596.0665
9.0
353.4
697.8
1042.2
1386.6
1731.0

M15

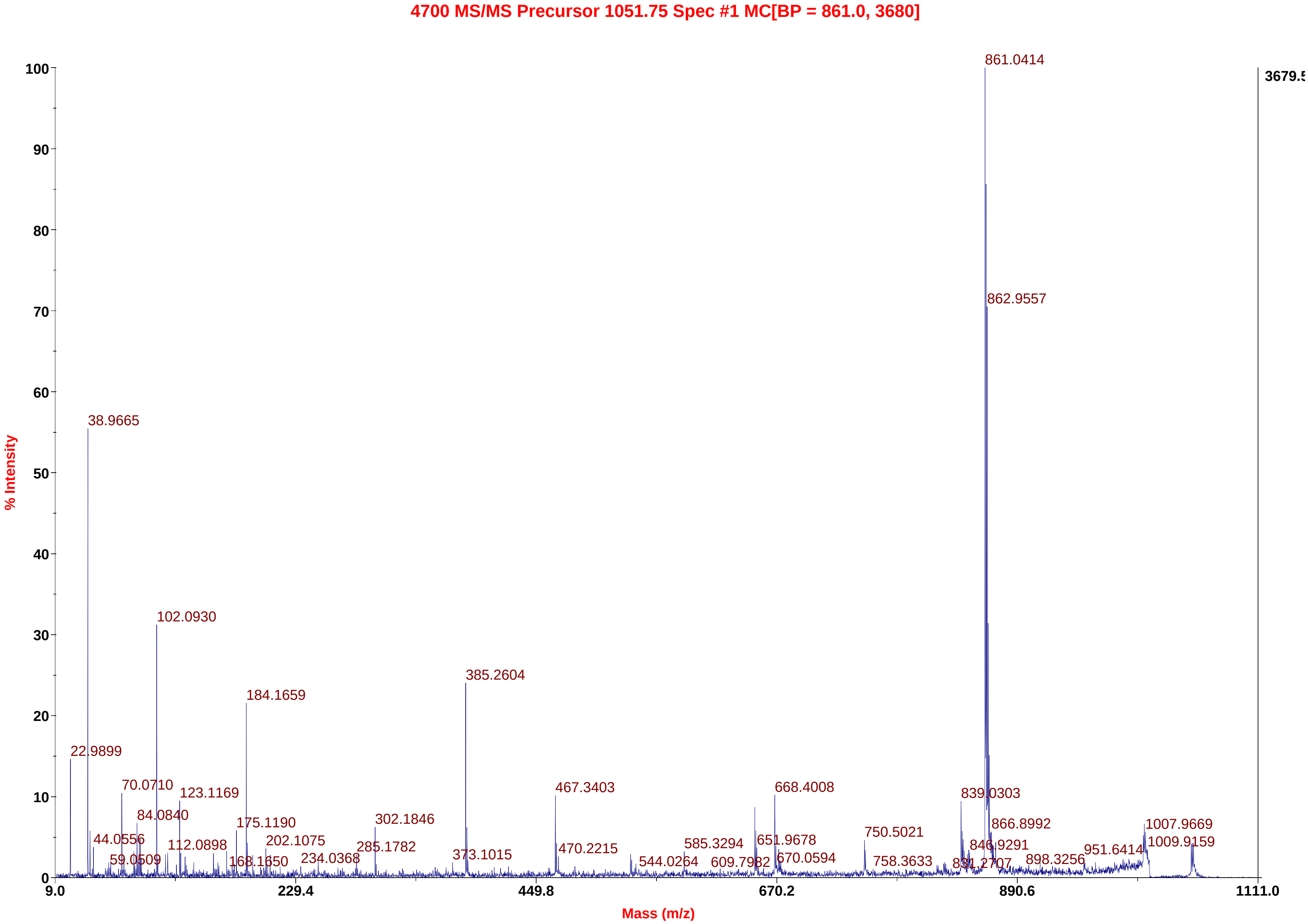

4700 MS/MS Precursor 1051.75 Spec #1 MC[BP = 861.0, 3680]
% Intensity
Mass (m/z)
3679.5
861.0414
862.9557
38.9665
102.0930
385.2604
184.1659
22.9899
70.0710
123.1169
84.0840
44.0556
59.0509
112.0898
168.1650
175.1190
202.1075
234.0368
285.1782
302.1846
373.1015
467.3403
470.2215
544.0264
585.3294
609.7982
651.9678
668.4008
670.0594
750.5021
758.3633
839.0303
831.2707
846.9291
866.8992
898.3256
951.6414
1007.9669
1009.9159

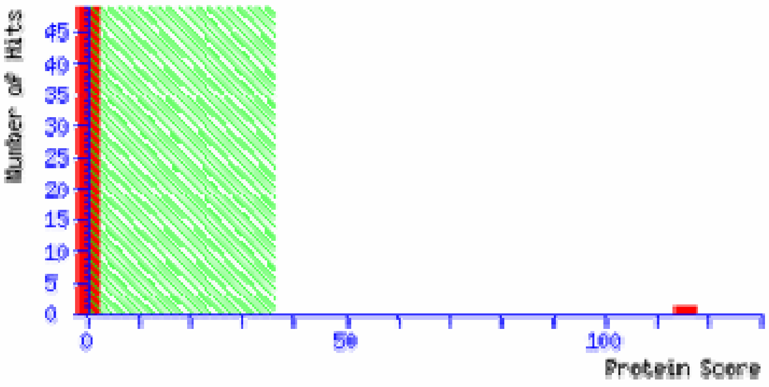

Peptide Summary Report

Format As: Peptide Summary   Help

Significance threshold p< 0.05   Max. number of hits AUTO

Standard scoring ○ MudPIT scoring ◉   Ions score or expect cut-off 0   Show sub-sets 0

Show pop-ups ◉ Suppress pop-ups ○   Sort unassigned Decreasing Score   Require bold red ☐

Select All   Select None   Search Selected   ☐ **Error tolerant**

1. Q3UKP2_MOUSE **Mass:** 51285 **Score:** 115 **Matches:** 14(3) **Sequences:** 14(3) **emPAI:** 0.95

14 days embryo liver cDNA, RIKEN full-length enriched library, clone:I530016F13 product:hemopexin, full insert sec

☐ Check to include this hit in error tolerant search

	Query	Observed	Mr(expt)	Mr(calc)	ppm	Miss	Score	Expect	Rank	Unique	Peptide
	2	975.5938	974.5865	974.5437	43.9	0	25	0.62	3	U	R.GPDSVFLIK.E
	3	1017.5829	1016.5756	1016.5331	41.8	0	14	13	3	U	K.VWVYPPEK.K
☑	6	1079.5121	1078.5048	1078.4542	46.9	0	25	1.1	1	U	R.YYCFQGNK.F + Carbamidomethyl (C)
☑	7	1100.5388	1099.5315	1099.4757	50.8	0	22	1.8	1	U	R.DYFVSCPGR.G + Carbamidomethyl (C)
☑	8	1128.5817	1127.5744	1127.5189	49.3	0	4	1.3e+02	1	U	K.WFWDFATR.T
	9	1145.6919	1144.6846	1144.6281	49.4	1	6	46	2	U	K.VWVYPPEKK.E
☑	11	1190.6782	1189.6709	1189.6091	51.9	0	31	0.22	1	U	K.NPITSVDAAFR.G
☑	12	1212.7021	1211.6948	1211.6299	53.6	0	35	0.052	1	U	R.FNPVTGEVPPR.Y
☑	14	1504.8770	1503.8697	1503.7834	57.4	1	12	11	1	U	R.WKNPITSVDAAFR.G

Protein View

Match to: **Q3UKP2_MOUSE** Score: **115**
14 days embryo liver cDNA, RIKEN full-length enriched library, clone:I530016F13 product:hemopexin, full
Found in search of ppw_o2_12990856114.txt

Nominal mass (M_r): **51285**; Calculated pI value: **7.92**
NCBI BLAST search of Q3UKP2_MOUSE against nr
Unformatted sequence string for pasting into other applications

Taxonomy: Mus musculus
Links to retrieve other entries containing this sequence from NCBI Entrez:
(no taxonomy information for this entry)

Variable modifications: Carbamidomethyl (C),Deamidated (NQ),Oxidation (M)
Cleavage by Trypsin: cuts C-term side of KR unless next residue is P
Sequence Coverage: 33%

Matched peptides shown in **Bold Red**

```
  1 MARTAVALNI LVLLGLCWSL AVASPLPTAN GRVAEVENGT KPDSDVPEHC
 51 LDTWSFDAAT MDHNGTMLFF KGEFVWRGHS GTRELISARW KNPITSVDAA
101 FRGPDSVFLI KEDKVWVYPP EKKENGYPKL FQEEFPGIPY PPDAAVECHR
151 GECQSEGVLF FQGNRKWFWD FATRTQKERS WSTVGNCTAA LRWLERYYCF
201 QGNKFLRFNP VTGEVPPRYP LDARDYFVSC PGRGHGRPRN GTAHGNSTHP
251 MHSRCSPDPG LTALLSDHRG ATYAFTGSHY WRLDSSRDGW HSWPIAHHWP
301 QGPSTVDAAF SWDDKVYLIQ GTQVYVFLTK GGNNLVSGYP KRLEKELGSP
351 PGISLETIDA AFSCPGSSRL YVSSGRRLWW LDLKSGAQAT WTEVSWPHEK
401 VDGALCLDKS LGPNTCSSNG SSLYFIHGPN LYCYSSIDKL NAAKSLPQPQ
451 KVNSILGCSQ
```

Show predicted peptides also

Sort Peptides By ◉ Residue Number ○ Increasing Mass ○ Decreasing Mass

Start - End	Observed	Mr(expt)	Mr(calc)	ppm	Miss	Sequence
90 - 102	1504.8770	1503.8697	1503.7834	57	1	R.WKNPITSVDAAFR.G (Ions score 12)
92 - 102	1190.6782	1189.6709	1189.6091	52	0	K.NPITSVDAAFR.G (Ions score 31)
103 - 111	975.5938	974.5865	974.5437	44	0	R.GPDSVFLIK.E (Ions score 25)
115 - 122	1017.5829	1016.5756	1016.5331	42	0	K.VWVYPPEK.K (Ions score 14)
115 - 123	1145.6919	1144.6846	1144.6281	49	1	K.VWVYPPEKK.E (Ions score 6)
130 - 150	2472.3220	2471.3147	2471.1580	63	0	K.LFQEEFPGIPYPPDAAVECHR.G Carbamidom
151 - 165	1727.8873	1726.8800	1726.7733	62	0	R.GECQSEGVLFFQGNR.K Carbamidomethyl
167 - 174	1128.5817	1127.5744	1127.5189	49	0	K.WFWDFATR.T (Ions score 4)
197 - 204	1079.5121	1078.5048	1078.4542	47	0	R.YYCFQGNK.F Carbamidomethyl (C) (Io

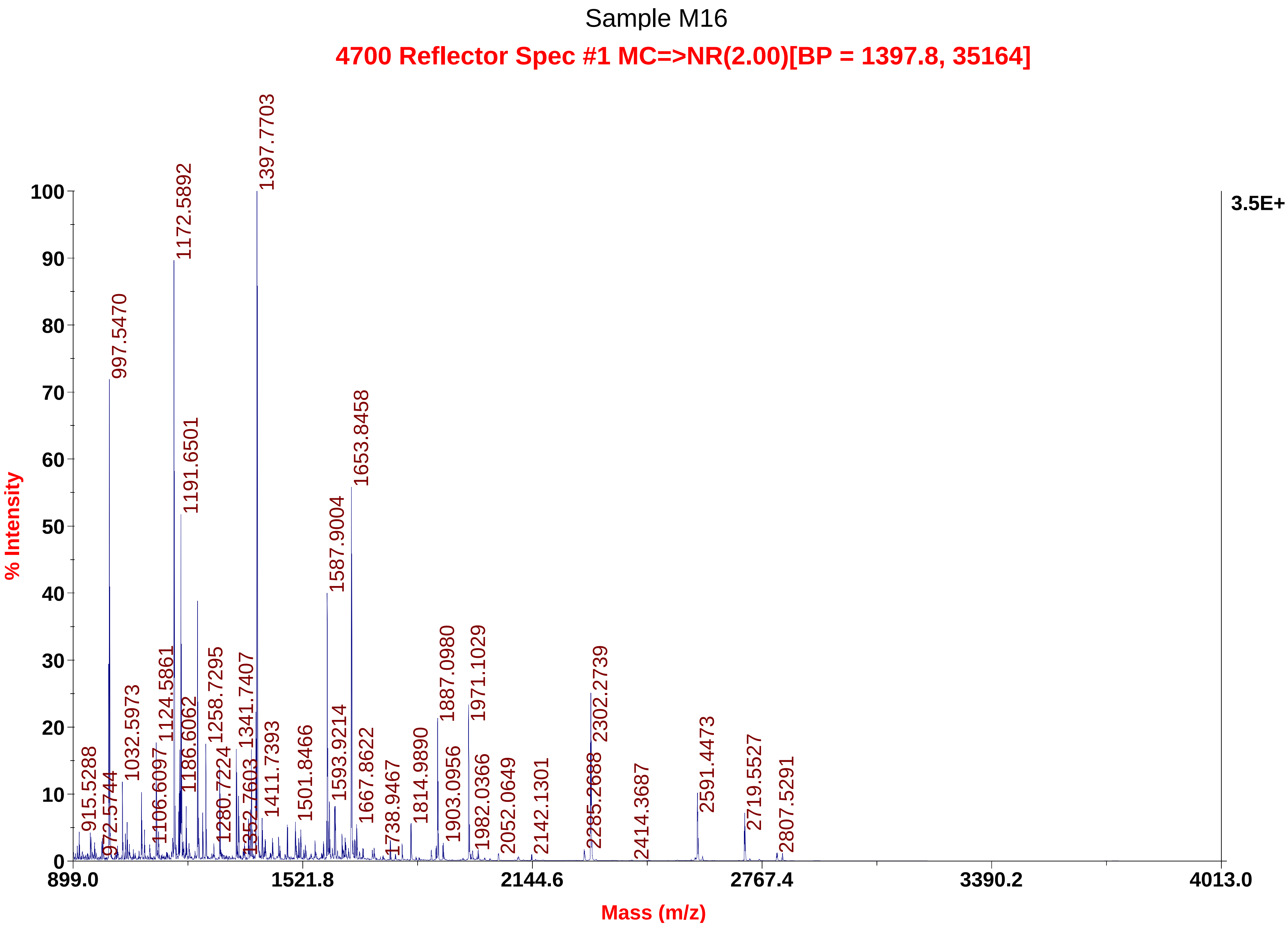
Sample M16
4700 Reflector Spec #1 MC=>NR(2.00)[BP = 1397.8, 35164]
% Intensity
Mass (m/z)
3.5E+
100
90
80
70
60
50
40
30
20
10
0
899.0
1521.8
2144.6
2767.4
3390.2
4013.0
1397.7703
1172.5892
997.5470
1191.6501
1653.8458
1587.9004
1887.0980
1971.1029
2302.2739
1124.5861
1258.7295
1341.7407
1032.5973
1186.6062
1593.9214
1411.7393
1501.8466
1667.8622
1814.9890
2591.4473
2719.5527
915.5288
972.5744
1106.6097
1280.7224
1352.7603
1738.9467
1903.0956
1982.0366
2052.0649
2142.1301
2285.2688
2414.3687
2807.5291

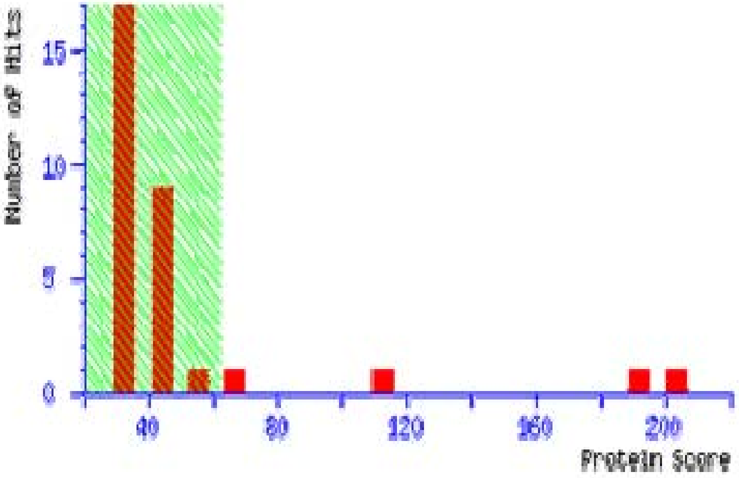

Concise Protein Summary Report

Format As | Concise Protein Summary | Help

Significance threshold p< 0.05 Max. number of hits AUTO

Re-Search All | Search Unmatched

1. Q8C2F4_MOUSE **Mass**: 56590 **Score**: 203 **Expect**: 4.6e-16 **Matches**: 45
   2 days neonate thymus thymic cells cDNA, RIKEN full-length enriched library, clone:E430024F21 product:glucose regulated
   Q99LF6_MOUSE **Mass**: 56643 **Score**: 203 **Expect**: 4.6e-16 **Matches**: 45
   Protein disulfide isomerase associated 3.- Mus musculus (Mouse).
   Q3TEI9_MOUSE **Mass**: 55393 **Score**: 190 **Expect**: 9.2e-15 **Matches**: 44
   2 days neonate thymus thymic cells cDNA, RIKEN full-length enriched library, clone:E430002E15 product:glucose regulated
   Q3UZK8_MOUSE **Mass**: 55446 **Score**: 190 **Expect**: 9.2e-15 **Matches**: 44
   8 days embryo whole body cDNA, RIKEN full-length enriched library, clone:5730420K02 product:glucose regulated protein,
   AAA39944 **Mass**: 56586 **Score**: 151 **Expect**: 7.3e-11 **Matches**: 42
   [illegible]

Protein View

Match to: **Q8C2F4_MOUSE** Score: **203** Expect: **4.6e-16**
2 days neonate thymus thymic cells cDNA, RIKEN full-length enriched library, clone:E430024F21 product:glucose regulated pr
Found in search of pmf_P2_129990849115.txt

Nominal mass (M_r): **56590**; Calculated pI value: **5.78**
NCBI BLAST search of Q8C2F4_MOUSE against nr
Unformatted sequence string for pasting into other applications

Taxonomy: Mus musculus
Links to retrieve other entries containing this sequence from NCBI Entrez:
(no taxonomy information for this entry)

Variable modifications: Carbamidomethyl (C),Deamidated (NQ),Oxidation (M)
Cleavage by Trypsin: cuts C-term side of KR unless next residue is P
Number of mass values searched: **170**
Number of mass values matched: **45**
Sequence Coverage: **69%**

Matched peptides shown in **Bold Red**

```
  1 MRFSCLALLP GVALLLASAC LAAASDVLEL TDENFESRVS DTGSAGLMLV
 51 EFFAPWCGHC KRLAPEYEAA ATRLKGIVPL AKVDCTANTN TCNKYGVSGY
101 PTLKIFRDGE EAGAYDGPRT ADGIVSHLKK QAGPASVPLR TEEEFKKFIS
151 DKDASVVGFF RDLFSDGHSE FLKAASNLRD NYRFAHTNIE SLVKEYDDNG
201 EGITIFRPLH LANKFEDKTV AYTEKKMTSG KIKKFIQDSI FGLCPHMTED
251 NKDLIQGKDL LTAYYDVDYE KNAKGSNYWR NRVMMVAKKF LDAGHKLNFA
301 VASRKTFSHE LSDFGLESTT GEVPVVAIRT AKGEKFVMQE EFSRDGKALE
351 QFLQEYFDGN LKRYLKSEPI PESNEGPVKV VVAENFDDIV NEEDKDVLIE
401 FYAPWCGHCK NLEPKYKELG EKLSKDPNIV IAKMDATAND VPSPYEVKGF
451 PTIYFSPANK KLTPKKYEGG RELNDFISYL QREATNPPII QEEKPKKKKK
501 AQEDL
```

Show predicted peptides also

Sort Peptides By ◉ Residue Number ○ Increasing Mass ○ Decreasing Mass

Start - End	Observed	Mr(expt)	Mr(calc)	ppm	Miss	Sequence
39 - 61	2585.3071	2584.2998	2584.1549	56	0	R.VSDTGSAGLMLVEFFAPWCGHCK.R 2 Carbamidomethyl (C); Oxid
62 - 73	1347.7603	1346.7530	1346.6942	44	1	K.RLAPEYEAAATR.L
63 - 73	1191.6493	1190.6420	1190.5931	41	0	R.LAPEYEAAATR.L
95 - 104	1084.6094	1083.6021	1083.5601	39	0	K.YGVSGYPTLK.I
95 - 107	1500.7830	1499.7757	1499.8136	-25	1	K.YGVSGYPTLKIFR.D
105 - 119	1652.8489	1651.8416	1651.7590	50	1	K.IFRDGEEAGAYDGPR.T
108 - 119	1236.5663	1235.5590	1235.5054	43	0	R.DGEEAGAYDGPR.T
120 - 129	1040.6093	1039.6020	1039.5662	34	0	R.TADGIVSHLK.K
120 - 130	1168.7041	1167.6968	1167.6612	31	1	R.TADGIVSHLKK.Q
130 - 140	1123.6980	1122.6907	1122.6509	35	1	K.KQAGPASVPLR.T

M16

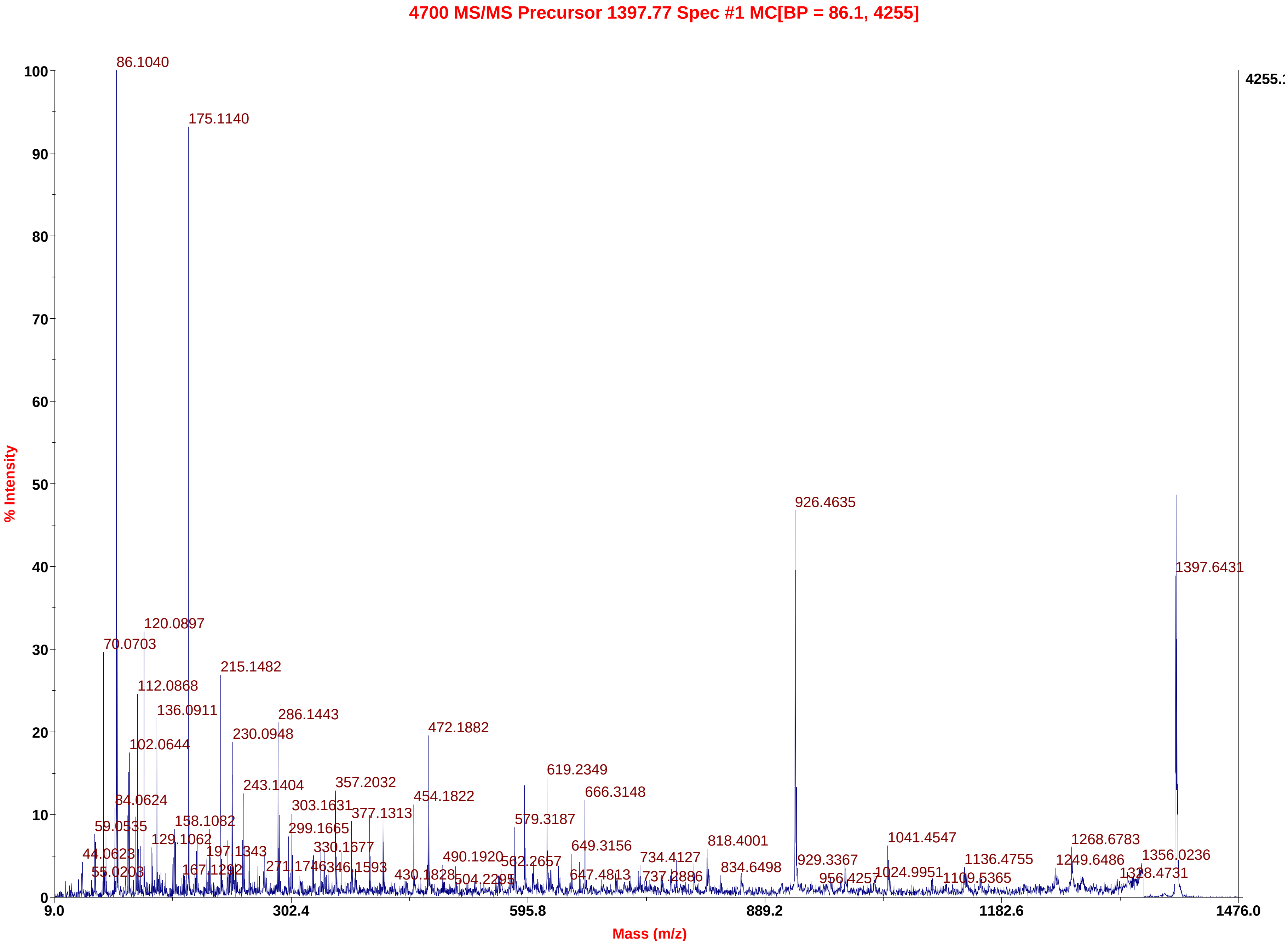
4700 MS/MS Precursor 1397.77 Spec #1 MC[BP = 86.1, 4255]
% Intensity
Mass (m/z)
86.1040
175.1140
926.4635
1397.6431
120.0897
70.0703
215.1482
112.0868
136.0911
286.1443
472.1882
230.0948
102.0644
619.2349
243.1404
357.2032
666.3148
84.0624
454.1822
303.1631
158.1082
377.1313
579.3187
59.0535
299.1665
129.1062
818.4001
1041.4547
1268.6783
649.3156
330.1677
44.0623
197.1343
490.1920
734.4127
1356.0236
929.3367
562.2657
1136.4755
1249.6486
271.1746
346.1593
834.6498
55.0203
167.1292
1024.9951
1328.4731
430.1828
647.4813
737.2886
956.4257
1109.5365
504.2295
4255.

M16

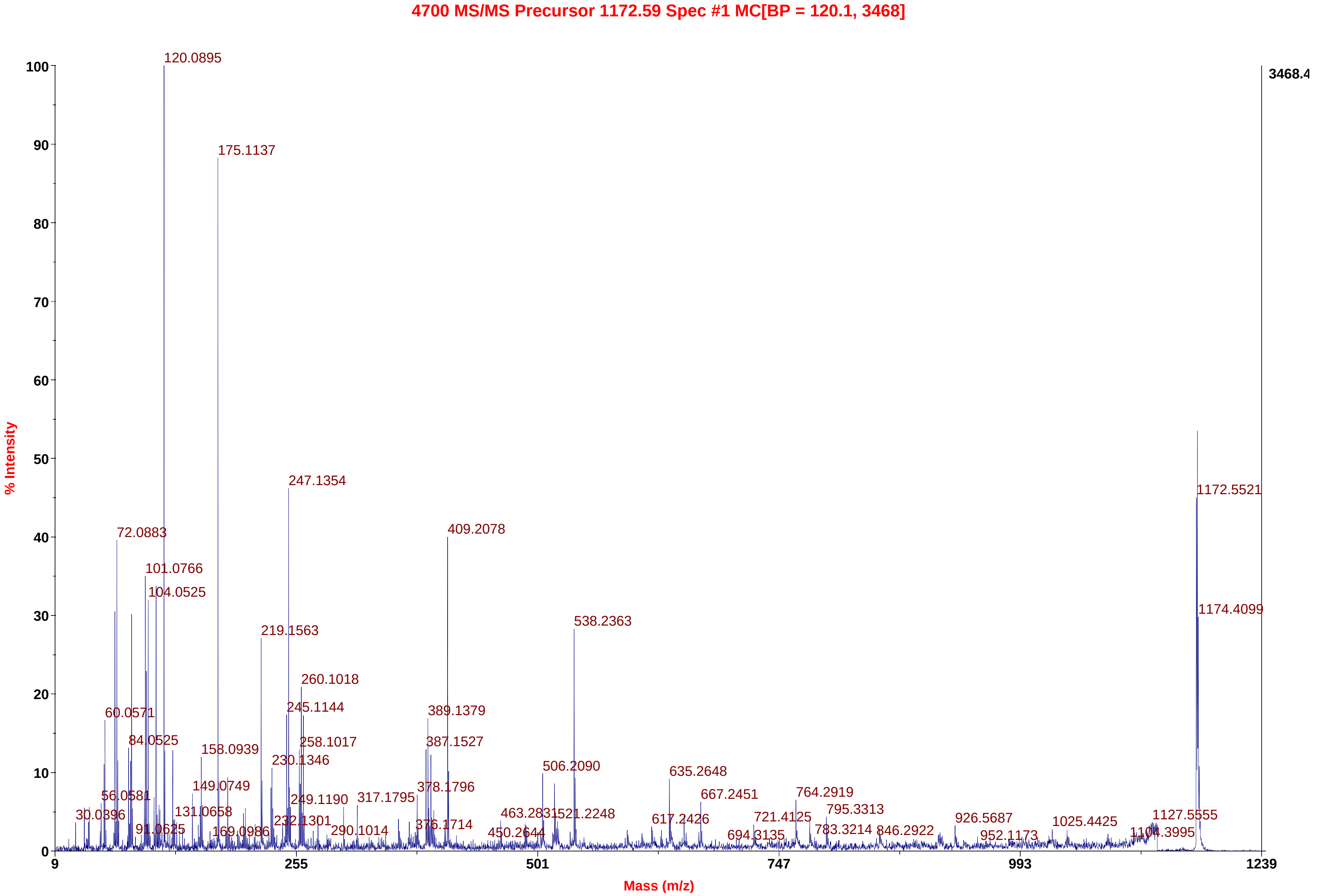

4700 MS/MS Precursor 1172.59 Spec #1 MC[BP = 120.1, 3468]
% Intensity
Mass (m/z)
3468.4
120.0895
175.1137
247.1354
409.2078
1172.5521
1174.4099
538.2363
219.1563
72.0883
101.0766
104.0525
260.1018
245.1144
389.1379
60.0571
84.0525
387.1527
258.1017
158.0939
230.1346
506.2090
635.2648
149.0749
378.1796
667.2451
764.2919
56.0581
249.1190
317.1795
795.3313
131.0658
30.0396
463.2831
521.2248
1127.5555
617.2426
721.4125
1025.4425
926.5687
232.1301
376.1714
91.0625
290.1014
783.3214
846.2922
169.0986
450.2644
1104.3995
694.3135
952.1173

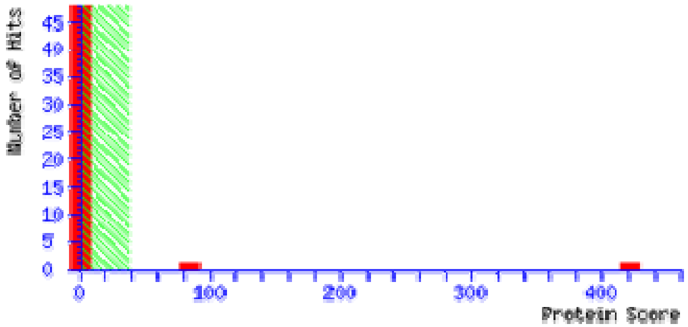

Peptide Summary Report

Format As | Peptide Summary | Help

Significance threshold p< 0.05 | Max. number of hits AUTO

Standard scoring ○ MudPIT scoring ● | Ions score or expect cut-off 0 | Show sub-sets 0

Show pop-ups ● Suppress pop-ups ○ | Sort unassigned Decreasing Score | Require bold red ☐

Select All | Select None | Search Selected | ☐ Error tolerant

1. Q3TEI9_MOUSE **Mass:** 55393 **Score:** 421 **Matches:** 15(11) **Sequences:** 15(11) **emPAI:** 3.06

2 days neonate thymus thymic cells cDNA, RIKEN full-length enriched library, clone:E430002E15 product:glucose regulated protein,

☐ Check to include this hit in error tolerant search

	Query	Observed	Mr(expt)	Mr(calc)	ppm	Miss	Score	Expect	Rank	Unique	Peptide
☑	1	997.5465	996.5392	996.5029	36.5	0	45	0.0075	1	U	K.DASVVGFFR.D
☑	3	1084.6094	1083.6021	1083.5601	38.8	0	27	0.43	1	U	K.YGVSGYPTLK.I
☑	5	1172.5880	1171.5807	1171.5332	40.6	0	41	0.023	1	U	K.FVMQEEFSR.D
☑	6	1191.6493	1190.6420	1190.5931	41.1	0	43	0.014	1	U	R.LAPEYEAAATR.L
☑	7	1236.5663	1235.5590	1235.5054	43.4	0	72	1.7e-05	1	U	R.DGEEAGAYDGPR.T
☑	9	1258.7286	1257.7213	1257.6717	39.4	0	70	2.6e-05	1	U	R.FAHTNIESLVK.E
☑	10	1341.7379	1340.7306	1340.6765	40.4	0	29	0.32	1	U	K.GFPTIYFSPANK.K
☑	11	1382.7344	1381.7271	1381.6725	39.5	0	17	5.5	1	U	K.SEPIPESNEGPVK.V
☑	12	1397.7692	1396.7619	1396.6987	45.3	0	58	0.0004	1	U	R.ELNDFISYLQR.E
☑	13	1587.8975	1586.8902	1586.8093	51.0	1	59	0.00029	1	U	K.FISDKDASVVGFFR.D
☑	14	1607.8287	1606.8214	1606.7403	50.5	0	32	0.22	1	U	K.DLLTAYYDVDYEK.N
☑	29	1971.0990	1970.0917	1969.9897	51.8	1	68	4.4e-05	1	U	K.ALEQFLQEYFDGNLKR.Y
☑	50	2302.2703	2301.2630	2301.1389	53.9	0	75	7e-06	1	U	K.EYDDNGEGITIFRPLHLANK.F
☑	74	2591.4419	2590.4346	2590.2915	55.2	0	98	3.5e-08	1	U	K.TFSHELSDFGLESTTGEVPVVAIR.T
☑	79	2719.5447	2718.5374	2718.3865	55.5	1	79	2.4e-06	1	U	R.KTFSHELSDFGLESTTGEVPVVAIR.T

Protein View

Match to: **Q3TEI9_MOUSE** Score: **421**
2 days neonate thymus thymic cells cDNA, RIKEN full-length enriched library, clone:E430002E15 product:gl
Found in search of ppw_P2_129990856215.txt

Nominal mass (M_r): **55393**; Calculated pI value: **5.53**
NCBI BLAST search of Q3TEI9_MOUSE against nr
Unformatted sequence string for pasting into other applications

Taxonomy: Mus musculus
Links to retrieve other entries containing this sequence from NCBI Entrez:
(no taxonomy information for this entry)

Variable modifications: Carbamidomethyl (C),Deamidated (NQ),Oxidation (M)
Cleavage by Trypsin: cuts C-term side of KR unless next residue is P
Sequence Coverage: **35%**

Matched peptides shown in **Bold Red**

```
  1 MRFSCLALLP GVALLLASAC LAAASDVLEL TDENFESRVS DTGSAGLMLV
 51 EFFAPWCGHC KRLAPEYEAA ATRLKGIVPL AKVDCTANTN TCNKYGVSGY
101 PTLKIFRDGE EAGAYDGPRT ADGIVSHLKK QAGPASVPLR TEEEFKKFIS
151 DKDASVVGFF RDLFSDGHSE FLKAASNLRD NYRFAHTNIE SLVKEYDDNG
201 EGITIFRPLH LANKFEDKTV AYTEKKMTSG KIKKFIQDSI FGLCPHMTED
251 NKDLIQGKDL LTAYYDVDYE KNAKGSNYWR NRVMMVAKKF LDAGHKLNFA
301 VASRKTFSHE LSDFGLESTT GEVPVVAIRT AKGEKFVMQE EFSRDGKALE
351 QFLQEYFDGN LKRYLKSEPI PESNEGPVKV VVAENFDDIV NEEDKDVLIE
401 FYAPWCGHCK NLEPKYKELG EKLSKDPNIV IAKMDATAND VPSPYEVKGF
451 PTIYFSPANK KLTPKKYEGG RELNDFISYL QREATNPPII QEEKP
```

Show predicted peptides also

Sort Peptides By ( ) Residue Number ( ) Increasing Mass ( ) Decreasing Mass

Start - End	Observed	Mr(expt)	Mr(calc)	ppm	Miss	Sequence
63 - 73	1191.6493	1190.6420	1190.5931	41	0	R.LAPEYEAAATR.L (Ions score 43)
95 - 104	1084.6094	1083.6021	1083.5601	39	0	K.YGVSGYPTLK.I (Ions score 27)
108 - 119	1236.5663	1235.5590	1235.5054	43	0	R.DGEEAGAYDGPR.T (Ions score 72)
148 - 161	1587.8975	1586.8902	1586.8093	51	1	K.FISDKDASVVGFFR.D (Ions score 59)

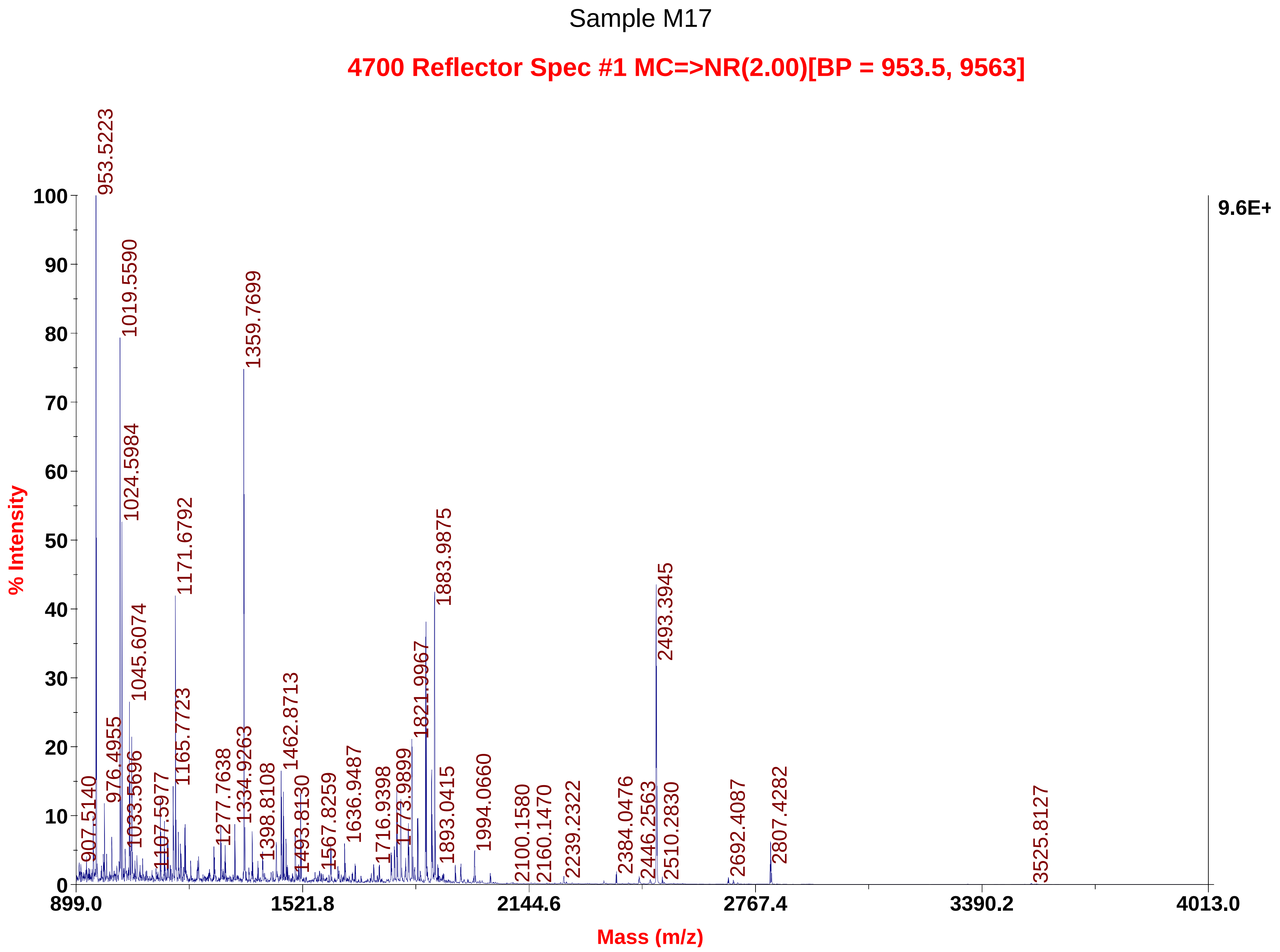

Sample M17
4700 Reflector Spec #1 MC=>NR(2.00)[BP = 953.5, 9563]
% Intensity
Mass (m/z)
9.6E+
953.5223
1019.5590
1359.7699
1024.5984
1171.6792
1883.9875
2493.3945
1045.6074
1821.9967
1165.7723
1462.8713
976.4955
1033.5696
1334.9263
907.5140
1107.5977
1277.7638
1398.8108
1493.8130
1567.8259
1636.9487
1716.9398
1773.9899
1893.0415
1994.0660
2100.1580
2160.1470
2239.2322
2384.0476
2446.2563
2510.2830
2692.4087
2807.4282
3525.8127
899.0
1521.8
2144.6
2767.4
3390.2
4013.0

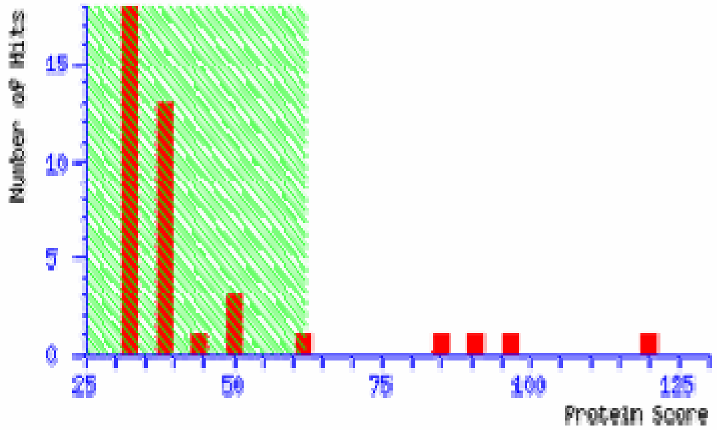

Concise Protein Summary Report

Format As | Concise Protein Summary | Help

Significance threshold p< 0.05 Max. number of hits AUTO

Re-Search All | Search Unmatched

1. Q3U1X3_MOUSE **Mass**: 43138 **Score**: 120 **Expect**: 9.2e-08 **Matches**: 35
   B6-derived CD11 +ve dendritic cells cDNA, RIKEN full-length enriched library, clone:F730110C17 product:pyruvate kinase, mu

---

2. Q4VC20_MOUSE **Mass**: 57808 **Score**: 99 **Expect**: 1.1e-05 **Matches**: 38
   Pyruvate kinase 3.- Mus musculus (Mouse).
   Q3TBW5_MOUSE **Mass**: 57807 **Score**: 99 **Expect**: 1.1e-05 **Matches**: 38
   NOD-derived CD11c +ve dendritic cells cDNA, RIKEN full-length enriched library, clone:F630218O07 product:pyruvate kinase,
   Q3TC59_MOUSE **Mass**: 57809 **Score**: 99 **Expect**: 1.1e-05 **Matches**: 37
   NOD-derived CD11c +ve dendritic cells cDNA, RIKEN full-length enriched library, clone:F630207O21 product:pyruvate kinase,
   Q3U5P6_MOUSE **Mass**: 57820 **Score**: 99 **Expect**: 1.1e-05 **Matches**: 36
   Bone marrow macrophage cDNA, RIKEN full-length enriched library, clone:I830166F06 product:pyruvate kinase, muscle, full in
   Q3TBV8_MOUSE **Mass**: 57750 **Score**: 90 **Expect**: 8.4e-05 **Matches**: 37
   NOD-derived CD11c +ve dendritic cells cDNA, RIKEN full-length enriched library, clone:F630220E24 product:pyruvate kinase,

Protein View

Match to: **Q3U1X3_MOUSE** Score: **120** Expect: **9.2e-08**
B6-derived CD11 +ve dendritic cells cDNA, RIKEN full-length enriched library, clone:F730110C17 product:pyruvate kinase, muscle
Found in search of pmf_A6_129990866000.txt

Nominal mass (M_r): **43138**; Calculated pI value: **5.88**
NCBI BLAST search of Q3U1X3_MOUSE against nr
Unformatted sequence string for pasting into other applications

Taxonomy: Mus musculus
Links to retrieve other entries containing this sequence from NCBI Entrez:
(no taxonomy information for this entry)

Variable modifications: Carbamidomethyl (C),Deamidated (NQ),Oxidation (M)
Cleavage by Trypsin: cuts C-term side of KR unless next residue is P
Number of mass values searched: **159**
Number of mass values matched: **35**
Sequence Coverage: **83%**

Matched peptides shown in **Bold Red**

```
  1 MPKPHSEAGT AFIQTQQLHA AMADTFLEHM CRLDIDSAPI TARNTGIICT
 51 IGPASRSVEM LKEMIKSGMN VARLNFSHGT HEYHAETIKN VREATESFAS
101 DPILYRPVAV ALDTKGPEIR TGLIKGSGTA EVELKKGATL KITLDNAYME
151 KCDENILWLD YKNICKVVEV GSKIYVDDGL ISLQVKEKGA DFLVTEVENG
201 GSLGSKKGVN LPGAAVDLPA VSEKDIQDLK FGVEQDVDMV FASFIRKAAD
251 VHEVRKVLGE KGKNIKIISK IENHEGVRRF DEILEASDGI MVARGDLGIE
301 IPAEKVFLAQ KMMIGRCNRA GKPVICATQM LESMIKKPRP TRAEGSDVAN
351 AVLDGADCIM LSGETAKGDY PLEAVRMQHL IAREAEAAMF HRLLF
```

Show predicted peptides also

Sort Peptides By ◉ Residue Number ○ Increasing Mass ○ Decreasing Mass

Start - End	Observed	Mr(expt)	Mr(calc)	ppm	Miss	Sequence
2 - 32	3523.7729	3522.7656	3522.6595	30	0	M.PKPHSEAGTAFIQTQQLHAAMADTFLEHMCR.L Carbamidomethyl (C)
33 - 43	1171.6792	1170.6719	1170.6244	41	0	R.LDIDSAPITAR.N
44 - 56	1302.7417	1301.7344	1301.6762	45	0	R.NTGIICTIGPASR.S
44 - 56	1359.7695	1358.7622	1358.6976	48	0	R.NTGIICTIGPASR.S Carbamidomethyl (C)
44 - 62	1990.0870	1989.0797	1989.0387	21	1	R.NTGIICTIGPASRSVEMLK.E
63 - 73	1235.6343	1234.6270	1234.6162	9	1	K.EMIKSGMNVAR.L
74 - 89	1883.9849	1882.9776	1882.8962	43	0	R.LNFSHGTHEYHAETIK.N
93 - 115	2493.3906	2492.3833	2492.2798	42	0	R.EATESFASDPILYRPVAVALDTK.G
126 - 136	1118.6117	1117.6044	1117.5979	6	1	K.GSGTAEVELKK.G
142 - 151	1197.6309	1196.6236	1196.5747	41	0	K.ITLDNAYMEK.C
142 - 151	1213.6306	1212.6233	1212.5696	44	0	K.ITLDNAYMEK.C Oxidation (M)
[illegible]	[illegible]	[illegible]	[illegible]	[illegible]	[illegible]	[illegible]

M17

4700 MS/MS Precursor 1051.74 Spec #1 MC[BP = 861.0, 4180]

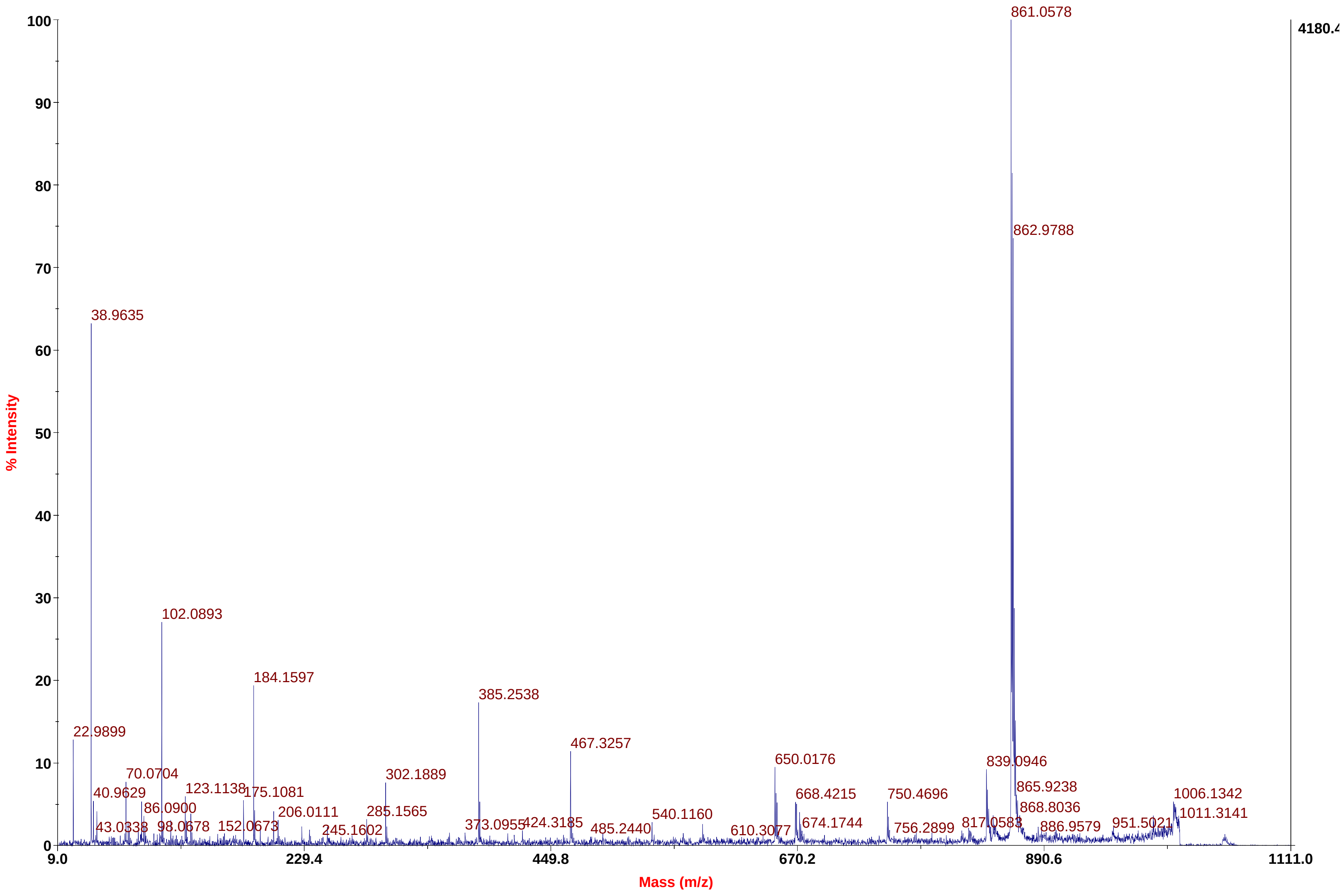

M17

4700 MS/MS Precursor 976.496 Spec #1 MC[BP = 343.2, 1047]

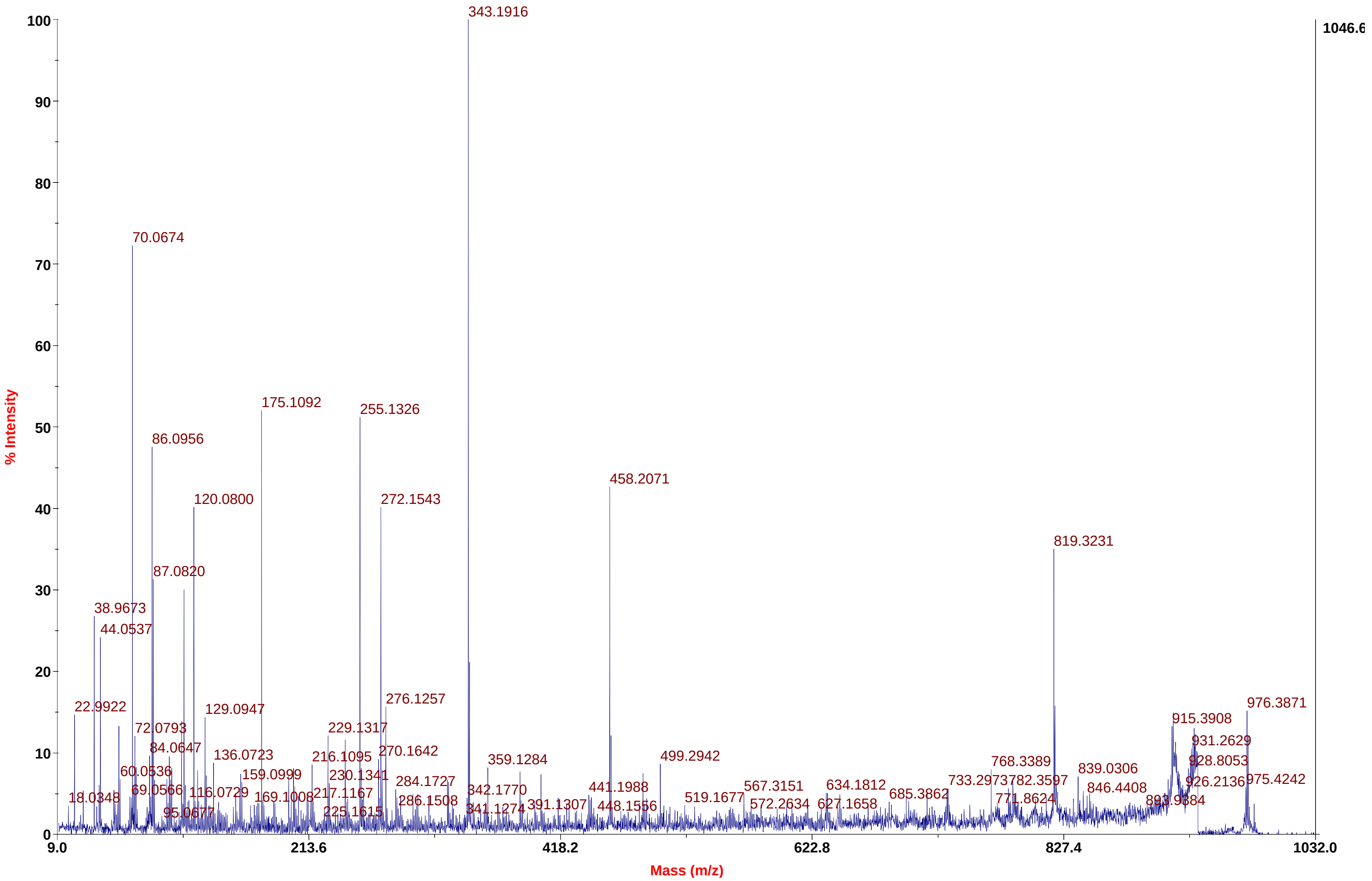

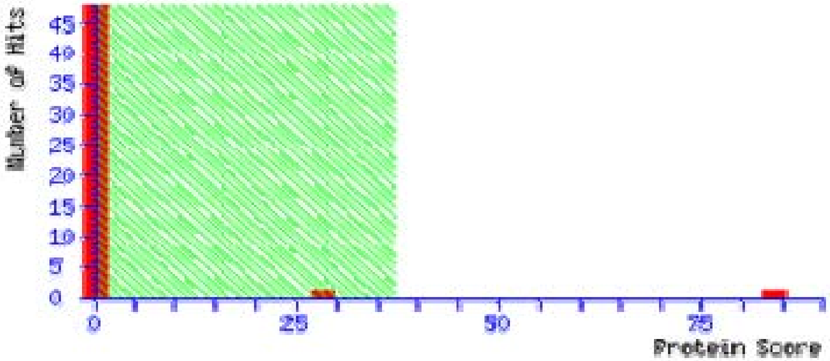

Peptide Summary Report

Format As: Peptide Summary — Help

Significance threshold p< 0.05 — Max. number of hits AUTO

Standard scoring ○ MudPIT scoring ● — Ions score or expect cut-off 0 — Show sub-sets 0

Show pop-ups ● Suppress pop-ups ○ — Sort unassigned Decreasing Score — Require bold red ☐

Select All — Select None — Search Selected — ☐ **Error tolerant**

1. KPYM_MOUSE **Mass:** 57719 **Score:** 84 **Matches:** 10(3) **Sequences:** 10(3) **emPAI:** 0.98
Pyruvate kinase isozyme M2 (EC 2.7.1.40).- Mus musculus (Mouse).
☐ Check to include this hit in error tolerant search

	Query	Observed	Mr(expt)	Mr(calc)	ppm	Miss	Score	Expect	Rank	Unique	Peptide
☑	1	953.5208	952.5135	952.4726	42.9	0	37	0.037	1	U	K.IENHEGVR.R
☑	3	1019.5587	1018.5514	1018.5083	42.3	0	31	0.23	1	U	K.GDYPLEAVR.M
	7	1141.6431	1140.6358	1140.6026	29.1	0	22	1.4	3		R.GDLGIEIPAEK.V
☑	8	1171.6792	1170.6719	1170.6244	40.6	0	39	0.029	1	U	R.LDIDSAPITAR.N
☑	9	1359.7695	1358.7622	1358.6976	47.5	0	34	0.11	1	U	R.NTGIICTIGPASR.S + Carbamid
☑	10	1462.8705	1461.8632	1461.8079	37.9	0	34	0.06	1	U	K.IYVDDGLISLQVK.E
☑	13	1779.9463	1778.9390	1778.8687	39.6	0	46	0.0075	1	U	K.GADFLVTEVENGGSLGSK.K
☑	18	1821.9973	1820.9900	1820.9091	44.5	1	23	1.6	1	U	R.RFDEILEASDGIMVAR.G
☑	22	1859.9863	1858.9790	1858.8924	46.6	0	16	7.9	1	U	K.FGVEQDVDMVFASFIR.K
☑	24	1883.9849	1882.9776	1882.8962	43.2	0	26	0.72	1	U	R.LNFSHGTHEYHAETIK.N

Protein View

Match to: **KPYM_MOUSE** Score: **84**
Pyruvate kinase isozyme M2 (EC 2.7.1.40).- Mus musculus (Mouse).
Found in search of ppw_A6_129990871300.txt

Nominal mass (M_r): **57719**; Calculated pI value: **7.42**
NCBI BLAST search of KPYM_MOUSE against nr
Unformatted sequence string for pasting into other applications

Taxonomy: Mus musculus

Variable modifications: Carbamidomethyl (C),Deamidated (NQ),Oxidation (M)
Cleavage by Trypsin: cuts C-term side of KR unless next residue is P
Sequence Coverage: **24%**

Matched peptides shown in **Bold Red**

```
  1 PKPHSEAGTA FIQTQQLHAA MADTFLEHMC RLDIDSAPIT ARNTGIICTI
 51 GPASRSVEML KEMIKSGMNV ARLNFSHGTH EYHAETIKNV REATESFASD
101 PILYRPVAVA LDTKGPEIRT GLIKGSGTAE VELKKGATLK ITLDNAYMEK
151 CDENILWLDY KNICKVVEVG SKIYVDDGLI SLQVKEKGAD FLVTEVENGG
201 SLGSKKGVNL PGAAVDLPAV SEKDIQDLKF GVEQDVDMVF ASFIRKAADV
251 HEVRKVLGEK GKNIKIISKI ENHEGVRRFD EILEASDGIM VARGDLGIEI
301 PAEKVFLAQK MMIGRCNRAG KPVICSTQML EIMIKKPRPT RAEGSDVANA
351 VLDGADCIML SGETAKGDYP LEAVRMQHLI AREAEAAIYH LQLFEELRRL
401 APITSDPTEA AAVGAVEASF KCCSGAIIVL TKSGRSAHQV ARYRPRAPII
451 AVTRNPQTAR QAHLYRGIFP VLCKDAVLNA WAEDVDLRVN LAMDVGKARG
501 FFKKGDVVIV LTGWRPGSGF TNTMRVVPVP
```

Show predicted peptides also

Sort Peptides By ◉ Residue Number ○ Increasing Mass ○ Decreasing Mass

Start – End	Observed	Mr(expt)	Mr(calc)	ppm	Miss	Sequence
32 – 42	1171.6792	1170.6719	1170.6244	41	0	R.LDIDSAPITAR.N (Ions score 39)
43 – 55	1359.7695	1358.7622	1358.6976	48	0	R.NTGIICTIGPASR.S Carbamidomethyl (C)
73 – 88	1883.9849	1882.9776	1882.8962	43	0	R.LNFSHGTHEYHAETIK.N (Ions score 26)
173 – 185	1462.8705	1461.8632	1461.8079	38	0	K.IYVDDGLISLQVK.E (Ions score 34)
188 – 205	1779.9463	1778.9390	1778.8687	40	0	K.GADFLVTEVENGGSLGSK.K (Ions score 46)
230 – 245	1859.9863	1858.9790	1858.8924	47	0	K.FGVEQDVDMVFASFIR.K (Ions score 16)
270 – 277	953.5208	952.5135	952.4726	43	0	K.IENHEGVR.R (Ions score 37)
278 – 293	1821.9973	1820.9900	1820.9091	44	1	R.RFDEILEASDGIMVAR.G (Ions score 23)
294 – 304	1141.6431	1140.6358	1140.6026	29	0	R.GDLGIEIPAEK.V (Ions score 22)
367 – 375	1019.5587	1018.5514	1018.5083	42	0	K.GDYPLEAVR.M (Ions score 31)

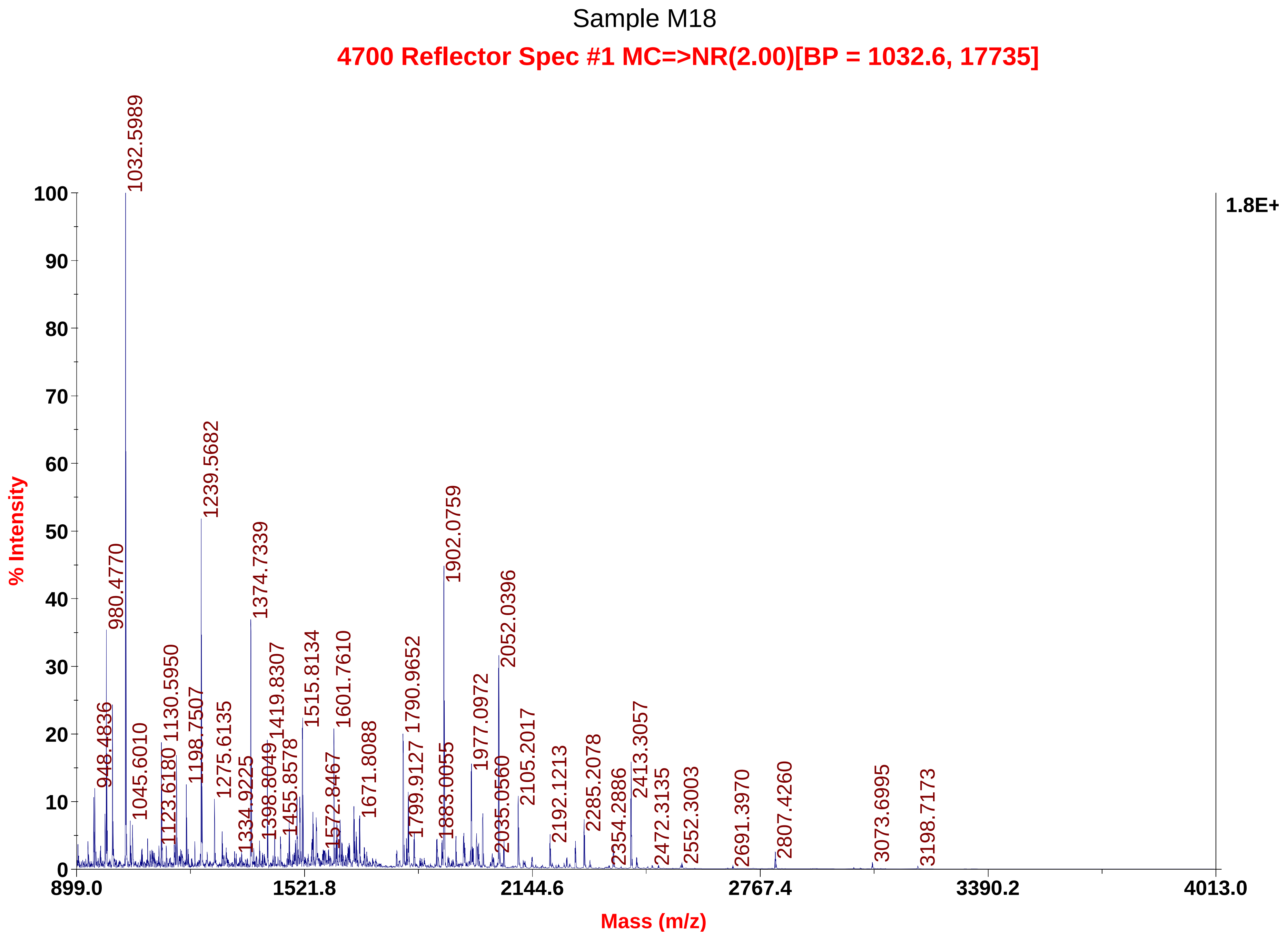

Sample M18
4700 Reflector Spec #1 MC=>NR(2.00)[BP = 1032.6, 17735]
% Intensity
Mass (m/z)
1.8E+
100
90
80
70
60
50
40
30
20
10
0
899.0
1521.8
2144.6
2767.4
3390.2
4013.0
948.4836
980.4770
1032.5989
1045.6010
1123.6180
1130.5950
1198.7507
1239.5682
1275.6135
1334.9225
1374.7339
1398.8049
1419.8307
1455.8578
1515.8134
1572.8467
1601.7610
1671.8088
1790.9652
1799.9127
1883.0055
1902.0759
1977.0972
2035.0560
2052.0396
2105.2017
2192.1213
2285.2078
2354.2886
2413.3057
2472.3135
2552.3003
2691.3970
2807.4260
3073.6995
3198.7173

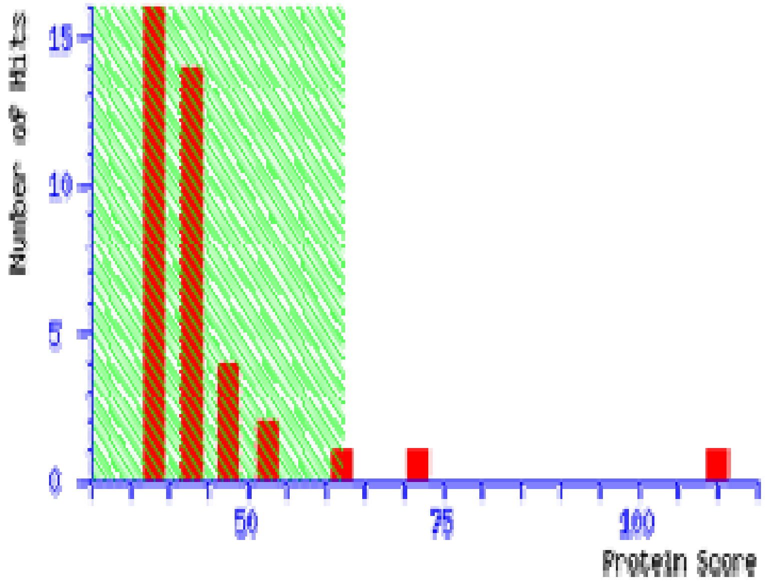

Concise Protein Summary Report

Format As | Concise Protein Summary | Help

Significance threshold p< 0.05 Max. number of hits AUTO

Re-Search All | Search Unmatched

1. Q3TGR2_MOUSE **Mass**: 54718 **Score**: 110 **Expect**: 9.2e-07 **Matches**: 42
17 days pregnant adult female amnion cDNA, RIKEN full-length enriched library, clone:I920039H17 product:fibrinogen,
AAL02225 **Mass**: 27037 **Score**: 71 **Expect**: 0.0072 **Matches**: 26
AF413205 NID: - Mus musculus

Protein View

Match to: **Q3TGR2_MOUSE** Score: **110** Expect: **9.2e-07**
17 days pregnant adult female amnion cDNA, RIKEN full-length enriched library, clone:I920039H17 product:fibrinogen
Found in search of pmf_B6_129990866001.txt

Nominal mass (M_r): **54718**; Calculated pI value: **6.68**
NCBI BLAST search of Q3TGR2_MOUSE against nr
Unformatted sequence string for pasting into other applications

Taxonomy: Mus musculus
Links to retrieve other entries containing this sequence from NCBI Entrez:
(no taxonomy information for this entry)
(no taxonomy information for this entry)
(no taxonomy information for this entry)
(no taxonomy information for this entry)

Variable modifications: Carbamidomethyl (C),Deamidated (NQ),Oxidation (M)
Cleavage by Trypsin: cuts C-term side of KR unless next residue is P
Number of mass values searched: **182**
Number of mass values matched: **42**
Sequence Coverage: **66%**

Matched peptides shown in **Bold Red**

```
  1 MRHLWLLLLL CVFSVQTQAA DDDYDEPTDS LDARGHRPVD RRKEEPPSLR
 51 PAPPPISGGG YRARPAKATA NQKKVERRPP DAGGCLHADT DMGVLCPTGC
101 TLQQTLLNQE RPIKSSIAEL NNNIQSVSDT SSVTFQYLTL LKDMWKKKQA
151 QVKENENVIN EYSSILEDQR LYIDETVNDN IPLNLRVLRS ILEDLRSKIQ
201 KLESDISAQM EYCRTPCTVS CNIPVVSGKE CEEIIRKGGE TSEMYLIQPD
251 TSIKPYRVYC DMKTENGGWT VIQNRQDGSV DFGRKWDPYK KGFGNIATNE
301 DAKKYCGLPG EYWLGNDKIS QLTRMGPTEL LIEMEDWKGD KVKAHYGGFT
351 VQNEASKYQV SVNKYKGTAG NALMDGASQL VGENRTMTIH NGMFFSTYDR
401 DNDGWVTTDP RKQCSKEDGG GWWYNRCHAA NPNGRYYWGG LYSWDMSKHG
451 TDDGVVWMNW KGSWYSMRRM SMKIRPFFPQ Q
```

Show predicted peptides also

Sort Peptides By ◉ Residue Number ○ Increasing Mass ○ Decreasing Mass

Start - End	Observed	Mr(expt)	Mr(calc)	ppm	Miss	Sequence
43 - 62	2105.2007	2104.1934	2104.1065	41	1	R.KEEPPSLRPAPPPISGGGYR.A
44 - 62	1977.0981	1976.0908	1976.0116	40	0	K.EEPPSLRPAPPPISGGGYR.A
115 - 142	3072.7024	3071.6951	3071.5663	42	0	K.SSIAELNNNIQSVSDTSSVTFQYLTLLK.D
154 - 170	2052.0364	2051.0291	2050.9443	41	0	K.ENENVINEYSSILEDQR.L
171 - 186	1902.0759	1901.0686	1900.9894	42	0	R.LYIDETVNDNIPLNLR.V
199 - 214	1971.0605	1970.0532	1969.9237	66	1	K.IQKLESDISAQMEYCR.T Carbamidomethyl (C)
202 - 214	1601.7582	1600.7509	1600.6861	40	0	K.LESDISAQMEYCR.T Carbamidomethyl (C)

M18

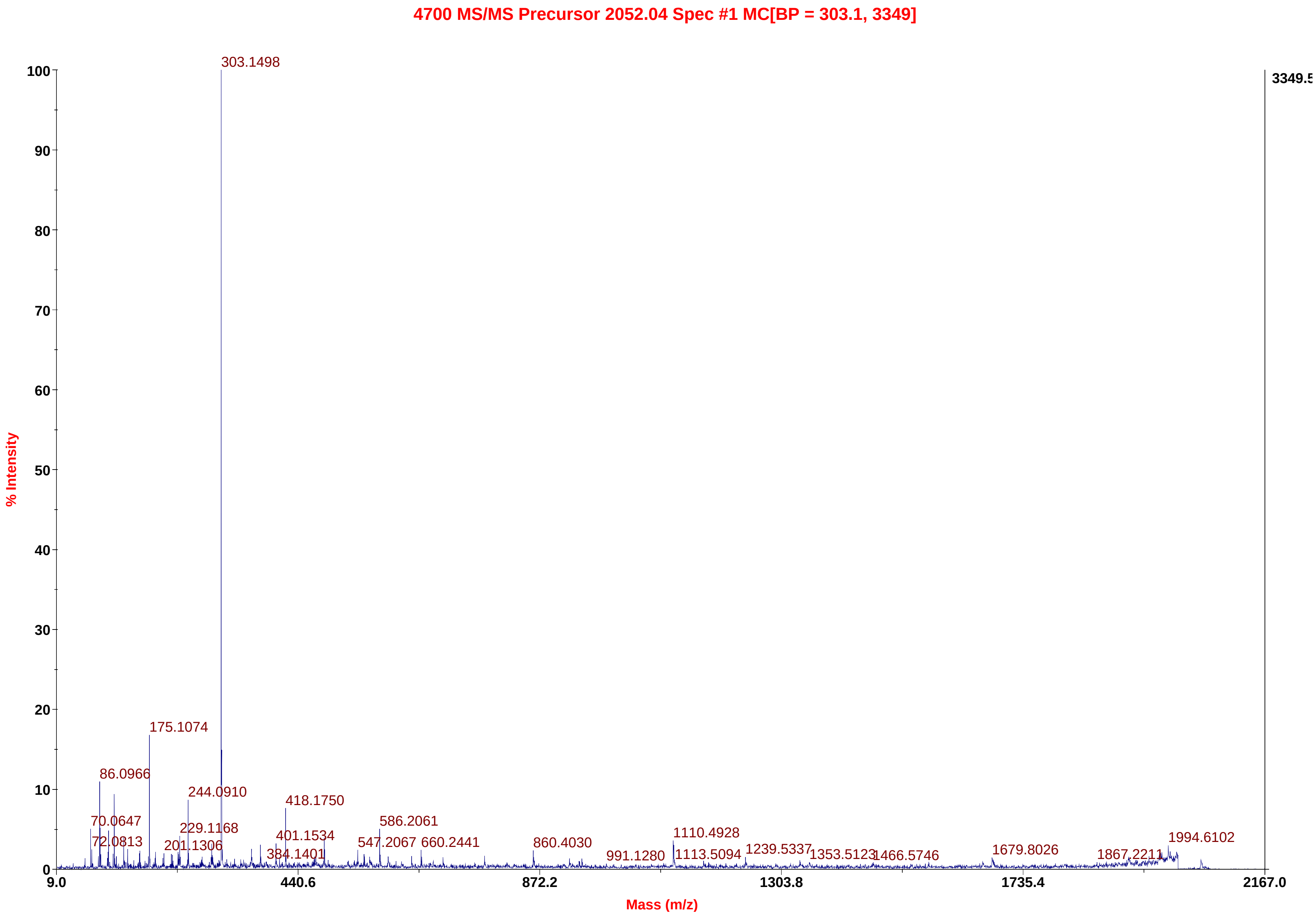
4700 MS/MS Precursor 2052.04 Spec #1 MC[BP = 303.1, 3349]
% Intensity
Mass (m/z)
303.1498
175.1074
86.0966
244.0910
418.1750
70.0647
229.1168
586.2061
72.0813
201.1306
401.1534
547.2067
660.2441
384.1401
860.4030
991.1280
1110.4928
1113.5094
1239.5337
1353.5123
1466.5746
1679.8026
1867.2211
1994.6102
3349.5
9.0
440.6
872.2
1303.8
1735.4
2167.0
0
10
20
30
40
50
60
70
80
90
100

M18

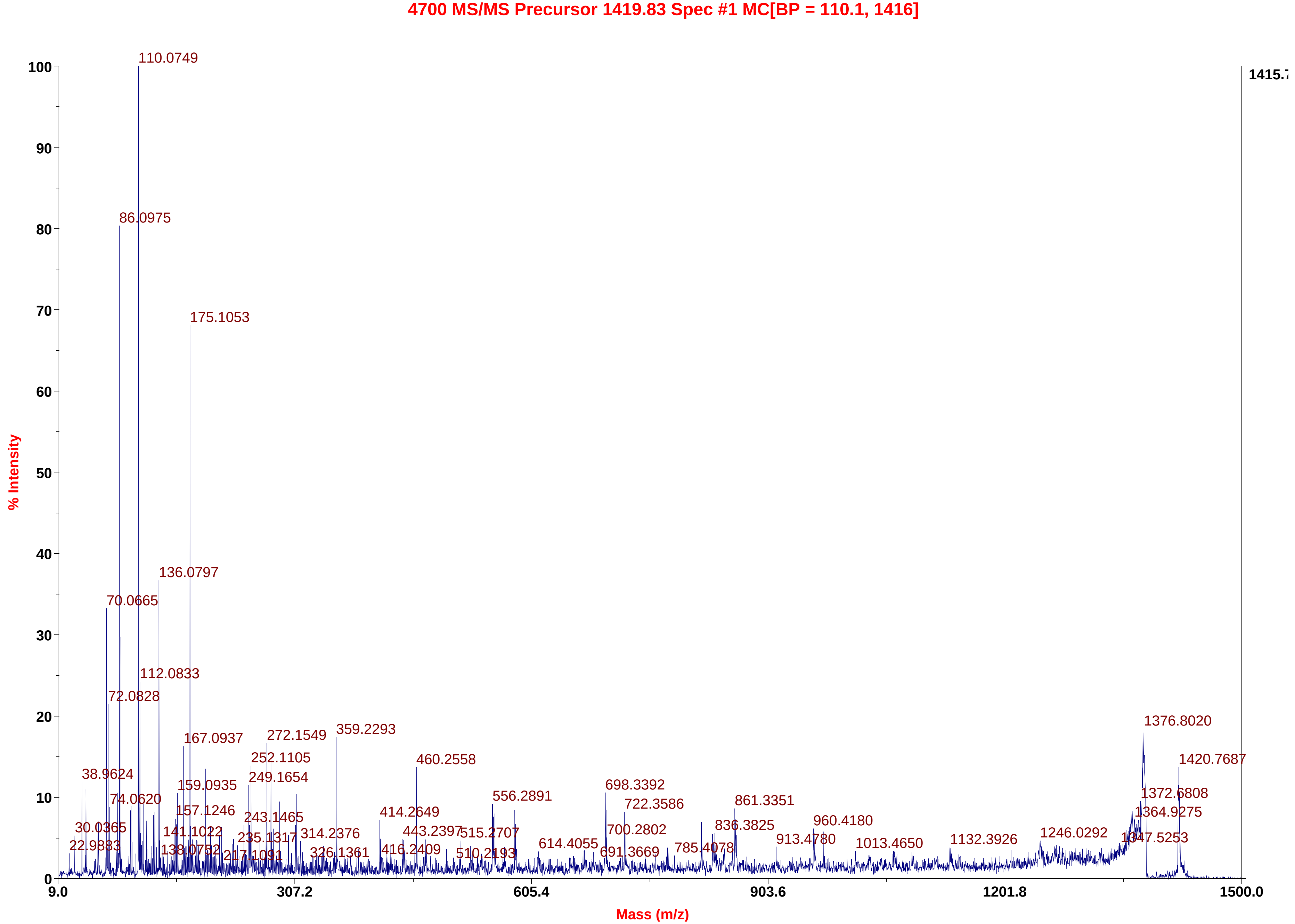
4700 MS/MS Precursor 1419.83 Spec #1 MC[BP = 110.1, 1416]
% Intensity
Mass (m/z)
110.0749
86.0975
175.1053
136.0797
70.0665
112.0833
72.0828
167.0937
272.1549
359.2293
252.1105
249.1654
159.0935
38.9624
74.0620
157.1246
243.1465
30.0365
141.1022
235.1317
314.2376
22.9883
138.0752
217.1091
326.1361
460.2558
414.2649
443.2397
416.2409
515.2707
510.2193
556.2891
614.4055
698.3392
722.3586
700.2802
691.3669
785.4078
836.3825
861.3351
913.4780
960.4180
1013.4650
1132.3926
1246.0292
1347.5253
1364.9275
1372.6808
1376.8020
1420.7687
1415.7

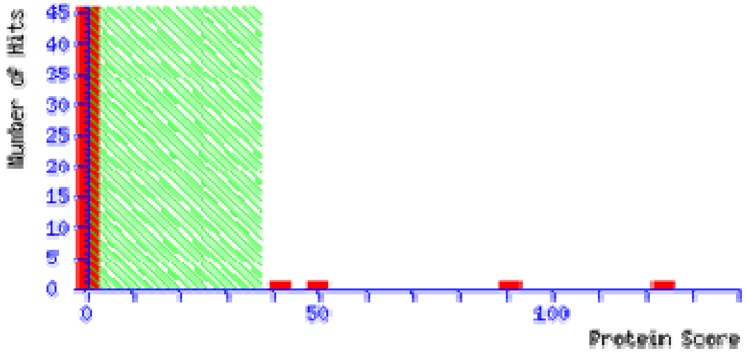

Peptide Summary Report

Format As | Peptide Summary | Help

Significance threshold p< 0.05 | Max. number of hits AUTO

Standard scoring ○ MudPIT scoring ● | Ions score or expect cut-off 0 | Show sub-sets 0

Show pop-ups ● Suppress pop-ups ○ | Sort unassigned Decreasing Score | Require bold red ☐

Select All | Select None | Search Selected | ☐ **Error tolerant**

1. Q3TGR2_MOUSE **Mass:** 54718 **Score:** 124 **Matches:** 12(3) **Sequences:** 12(3) **emPAI:** 0.68
17 days pregnant adult female amnion cDNA, RIKEN full-length enriched library, clone:I920039H17 product:fibrinogen,
☐ Check to include this hit in error tolerant search

	Query	Observed	Mr(expt)	Mr(calc)	ppm	Miss	Score	Expect	Rank	Unique	Peptide
☑	1	980.4769	979.4697	979.4359	34.4	0	27	0.52	1	U	R.QDGSVDFGR.K
☑	2	996.4788	995.4715	995.4355	36.2	0	33	0.11	1	U	R.CHAANPNGR.Y + Carbamidomethyl (C)
☑	3	1032.5969	1031.5896	1031.5553	33.3	0	18	5	1	U	K.IRPFFPQQ.-
	7	1239.5681	1238.5608	1238.5105	40.6	0	6	65	4	U	K.EDGGGWWYNR.C
☑	8	1374.7316	1373.7243	1373.6688	40.4	0	22	2.2	1	U	K.TENGGWTVIQNR.Q
☑	12	1601.7582	1600.7509	1600.6861	40.5	0	15	9.6	1	U	K.LESDISAQMEYCR.T + Carbamidomethyl (C
☑	13	1671.8080	1670.8007	1670.7399	36.4	0	15	11	1	U	K.YCGLPGEYWLGNDK.I + Carbamidomethyl (
☑	23	1902.0759	1901.0686	1900.9894	41.7	0	42	0.015	1	U	R.LYIDETVNDNIPLNLR.V
☑	29	1977.0981	1976.0908	1976.0116	40.1	0	17	6.1	1	U	K.EEPPSLRPAPPPISGGGYR.A
☑	32	2052.0364	2051.0291	2050.9443	41.4	0	51	0.0023	1	U	K.ENENVINEYSSILEDQR.L
☑	36	2105.2007	2104.1934	2104.1065	41.3	1	19	2.6	1	U	R.KEEPPSLRPAPPPISGGGYR.A
☑	57	2413.3040	2412.2967	2412.1995	40.3	1	70	2.6e-05	1	U	R.KGGETSEMYLIQPDTSIKPYR.V

Protein View

Match to: **Q3TGR2_MOUSE** Score: **124**
17 days pregnant adult female amnion cDNA, RIKEN full-length enriched library, clone:I920039H17 product:fibrinogen, B beta
Found in search of ppw_B6_129990871401.txt

Nominal mass (M_r): **54718**; Calculated pI value: **6.68**
NCBI BLAST search of Q3TGR2_MOUSE against nr
Unformatted sequence string for pasting into other applications

Taxonomy: Mus musculus
Links to retrieve other entries containing this sequence from NCBI Entrez:
(no taxonomy information for this entry)
(no taxonomy information for this entry)
(no taxonomy information for this entry)
(no taxonomy information for this entry)

Variable modifications: Carbamidomethyl (C),Deamidated (NQ),Oxidation (M)
Cleavage by Trypsin: cuts C-term side of KR unless next residue is P
Sequence Coverage: 30%

Matched peptides shown in **Bold Red**

```
  1 MRHLWLLLLL CVFSVQTQAA DDDYDEPTDS LDARGHRPVD RRKEEPPSLR
 51 PAPPPISGGG YRARPAKATA NQKKVERRPP DAGGCLHADT DMGVLCPTGC
101 TLQQTLLNQE RPIKSSIAEL NNNIQSVSDT SSVTFQYLTL LKDMWKKKQA
151 QVKENENVIN EYSSILEDQR LYIDETVNDN IPLNLRVLRS ILEDLRSKIQ
201 KLESDISAQM EYCRTPCTVS CNIPVVSGKE CEEIIRKGGE TSEMYLIQPD
251 TSIKPYRVYC DMKTENGGWT VIQNRQDGSV DFGRKWDPYK KGFGNIATNE
301 DAKKYCGLPG EYWLGNDKIS QLTRMGPTEL LIEMEDWKGD KVKAHYGGFT
351 VQNEASKYQV SVNKYKGTAG NALMDGASQL VGENRTMTIH NGMFFSTYDR
401 DNDGWVTTDP RKQCSKEDGG GWWYNRCHAA NPNGRYYWGG LYSWDMSKHG
451 TDDGVVWMNW KGSWYSMRRM SMKIRPFFPQ Q
```

Show predicted peptides also

Sort Peptides By ◉ Residue Number ○ Increasing Mass ○ Decreasing Mass

Start – End	Observed	Mr(expt)	Mr(calc)	ppm	Miss	Sequence
43 – 62	2105.2007	2104.1934	2104.1065	41	1	R.KEEPPSLRPAPPPISGGGYR.A (Ions score 19)
44 – 62	1977.0981	1976.0908	1976.0116	40	0	K.EEPPSLRPAPPPISGGGYR.A (Ions score 17)
154 – 170	2052.0364	2051.0291	2050.9443	41	0	K.ENENVINEYSSILEDQR.L (Ions score 51)
171 – 186	1902.0759	1901.0686	1900.9894	42	0	R.LYIDETVNDNIPLNLR.V (Ions score 42)
202 – 214	1601.7582	1600.7509	1600.6861	40	0	K.LESDISAQMEYCR.T Carbamidomethyl (C) (Ions score 15)
237 – 257	2413.3040	2412.2967	2412.1995	40	1	R.KGGETSEMYLIQPDTSIKPYR.V (Ions score 70)
264 – 275	1374.7316	1373.7243	1373.6688	40	0	K.TENGGWTVIQNR.Q (Ions score 22)
276 – 284	980.4769	979.4697	979.4359	34	0	R.QDGSVDFGR.K (Ions score 27)
305 – 318	1671.8080	1670.8007	1670.7399	36	0	K.YCGLPGEYWLGNDK.I Carbamidomethyl (C) (Ions score 15)
417 – 426	1239.5681	1238.5608	1238.5105	41	0	K.EDGGGWWYNR.C (Ions score 6)
427 – 435	996.4788	995.4715	995.4355	36	0	R.CHAANPNGR.Y Carbamidomethyl (C) (Ions score 33)
474 – 481	1032.5969	1031.5896	1031.5553	33	0	K.IRPFFPQQ.- (Ions score 18)

Sample M19

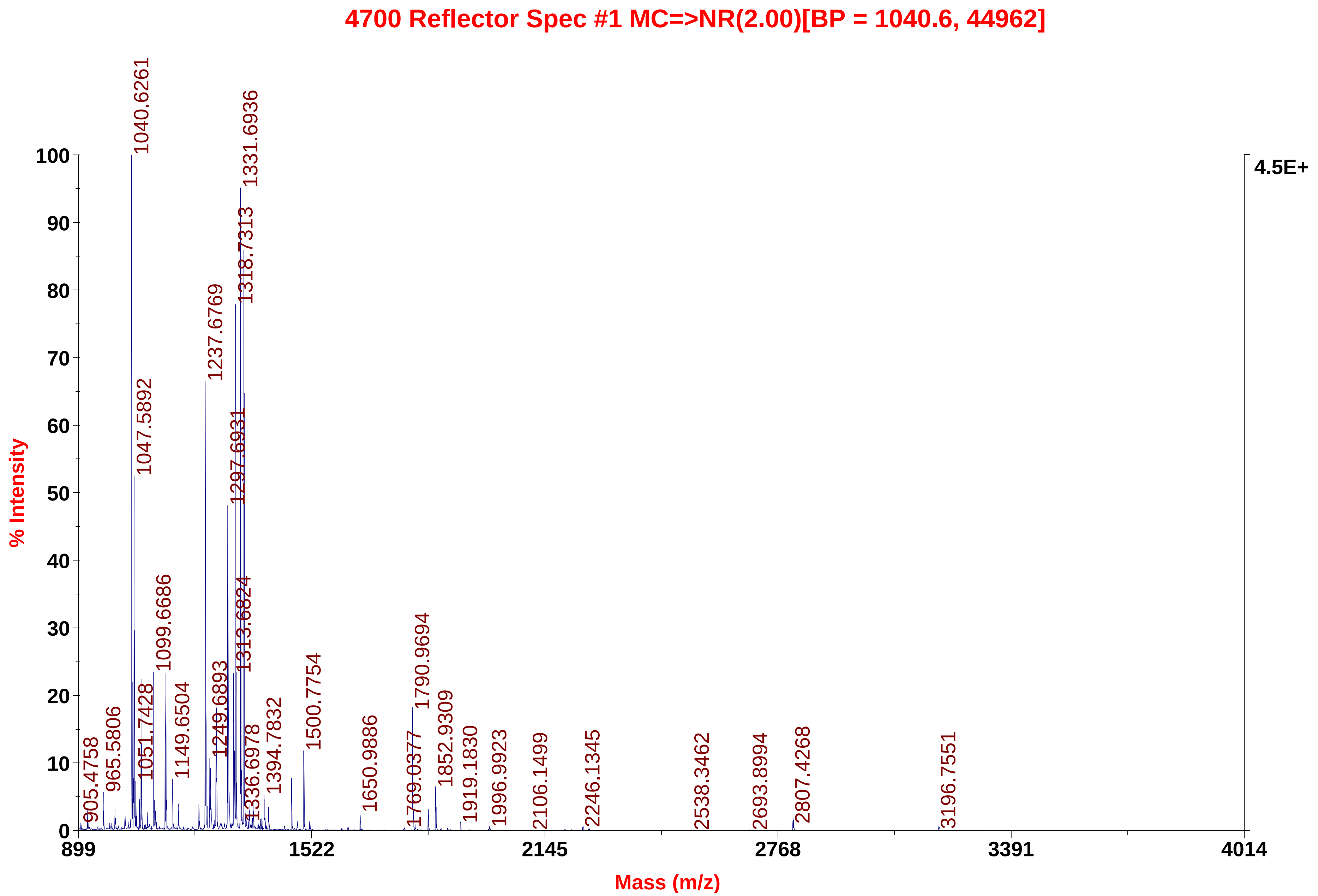

4700 Reflector Spec #1 MC=>NR(2.00)[BP = 1040.6, 44962]
4.5E+
% Intensity
Mass (m/z)
905.4758
965.5806
1040.6261
1047.5892
1051.7428
1099.6686
1149.6504
1237.6769
1249.6893
1297.6931
1313.6824
1318.7313
1331.6936
1336.6978
1394.7832
1500.7754
1650.9886
1769.0377
1790.9694
1852.9309
1919.1830
1996.9923
2106.1499
2246.1345
2538.3462
2693.8994
2807.4268
3196.7551

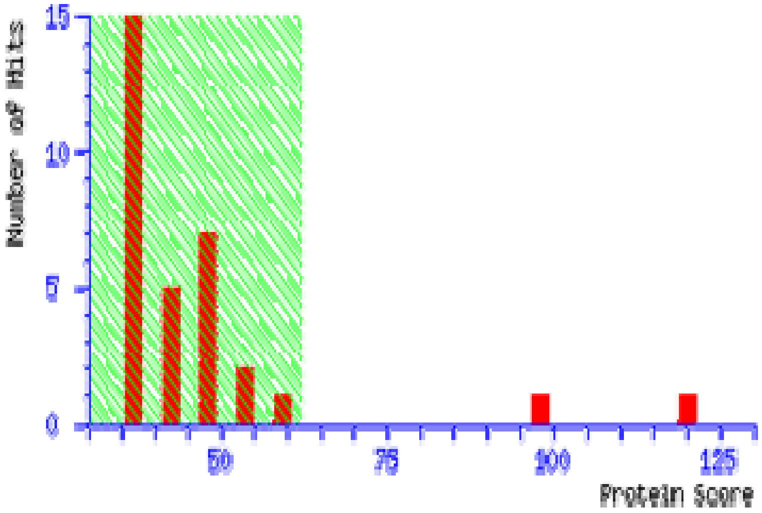

Concise Protein Summary Report

Format As | Concise Protein Summary | Help

Significance threshold p< 0.05 Max. number of hits AUTO

Re-Search All | Search Unmatched

1. Q3V2G1_MOUSE **Mass**: 30666 **Score**: 120 **Expect**: 9.2e-08 **Matches**: 24
   Adult male small intestine cDNA, RIKEN full-length enriched library, clone:2010012G12 product:apolipoprotein A-I,
   JC1237 **Mass**: 30358 **Score**: 113 **Expect**: 4.6e-07 **Matches**: 22
   apolipoprotein A-I precursor - mouse
   Q8BPD5_MOUSE **Mass**: 30597 **Score**: 110 **Expect**: 9.2e-07 **Matches**: 23
   18 days pregnant adult female placenta and extra embryonic tissue cDNA, RIKEN full-length enriched library, clone:
   S22420 **Mass**: 30569 **Score**: 110 **Expect**: 9.2e-07 **Matches**: 22
   apolipoprotein A-I precursor - mouse

Protein View

Match to: Q3V2G1_MOUSE Score: **120** Expect: **9.2e-08**
Adult male small intestine cDNA, RIKEN full-length enriched library, clone:2010012G12 product:apolipoprotein
Found in search of pmf_C6_129990866002.txt

Nominal mass (M_r): **30666**; Calculated pI value: **5.64**
NCBI BLAST search of Q3V2G1_MOUSE against nr
Unformatted sequence string for pasting into other applications

Taxonomy: Mus musculus
Links to retrieve other entries containing this sequence from NCBI Entrez:
(no taxonomy information for this entry)

Variable modifications: Carbamidomethyl (C),Deamidated (NQ),Oxidation (M)
Cleavage by Trypsin: cuts C-term side of KR unless next residue is P
Number of mass values searched: **147**
Number of mass values matched: **24**
Sequence Coverage: **60%**

Matched peptides shown in **Bold Red**

```
  1 MKAVVLAVAL VFLTGRQAWH VWQQDEPQSQ WDKVKDFANV YVDAVKDSGR
 51 DYVSQFESSS LGQQLNLNLL ENWDTLGSTV SQLQERLGPL TRDFWDNLEK
101 ETDWVRQEMN KDLEEVKQKV QPYLDEFQKK WKEDVELYRQ KVAPLGAELQ
151 ESARQKLQEL QGRLSPVAEE FRDRMRTHVD SLRTQLAPHS EQMRESLAQR
201 LAELKSNPTL NEYHTRAKTH LKTLGEKARP ALEDLRHSLM PMLETLKTQV
251 QSVIDKASET LTAQ
```

Show predicted peptides also

Sort Peptides By ◉ Residue Number ○ Increasing Mass ○ Decreasing Mass

Start – End	Observed	Mr(expt)	Mr(calc)	ppm	Miss	Sequence	
17 – 33	2198.1162	2197.1089	2196.9501	72	0	R.QAWHVWQQDEPQSQWDK.V	2 Deamidated (NQ)
34 – 46	1467.8397	1466.8324	1466.7769	38	1	K.VKDFANVYVDAVK.D	
36 – 46	1240.6721	1239.6648	1239.6136	41	0	K.DFANVYVDAVK.D	
36 – 50	1655.8489	1654.8416	1654.7951	28	1	K.DFANVYVDAVKDSGR.D	
93 – 100	1066.5186	1065.5113	1065.4767	32	0	R.DFWDNLEK.E	
93 – 106	1852.9277	1851.9204	1851.8428	42	1	R.DFWDNLEKETDWVR.Q	
107 – 117	1362.7241	1361.7168	1361.6496	49	1	R.QEMNKDLEEVK.Q	
107 – 117	1378.7196	1377.7123	1377.6446	49	1	R.QEMNKDLEEVK.Q	Oxidation (M)
120 – 129	1266.6832	1265.6759	1265.6292	37	0	K.VQPYLDEFQK.K	
120 – 130	1394.7819	1393.7746	1393.7242	36	1	K.VQPYLDEFQKK.W	
131 – 139	1237.6746	1236.6673	1236.6139	43	1	K.WKEDVELYR.Q	
133 – 139	923.4918	922.4845	922.4396	49	0	K.EDVELYR.Q	

M19

4700 MS/MS Precursor 1040.63 Spec #1 MC[BP = 884.4, 8838]

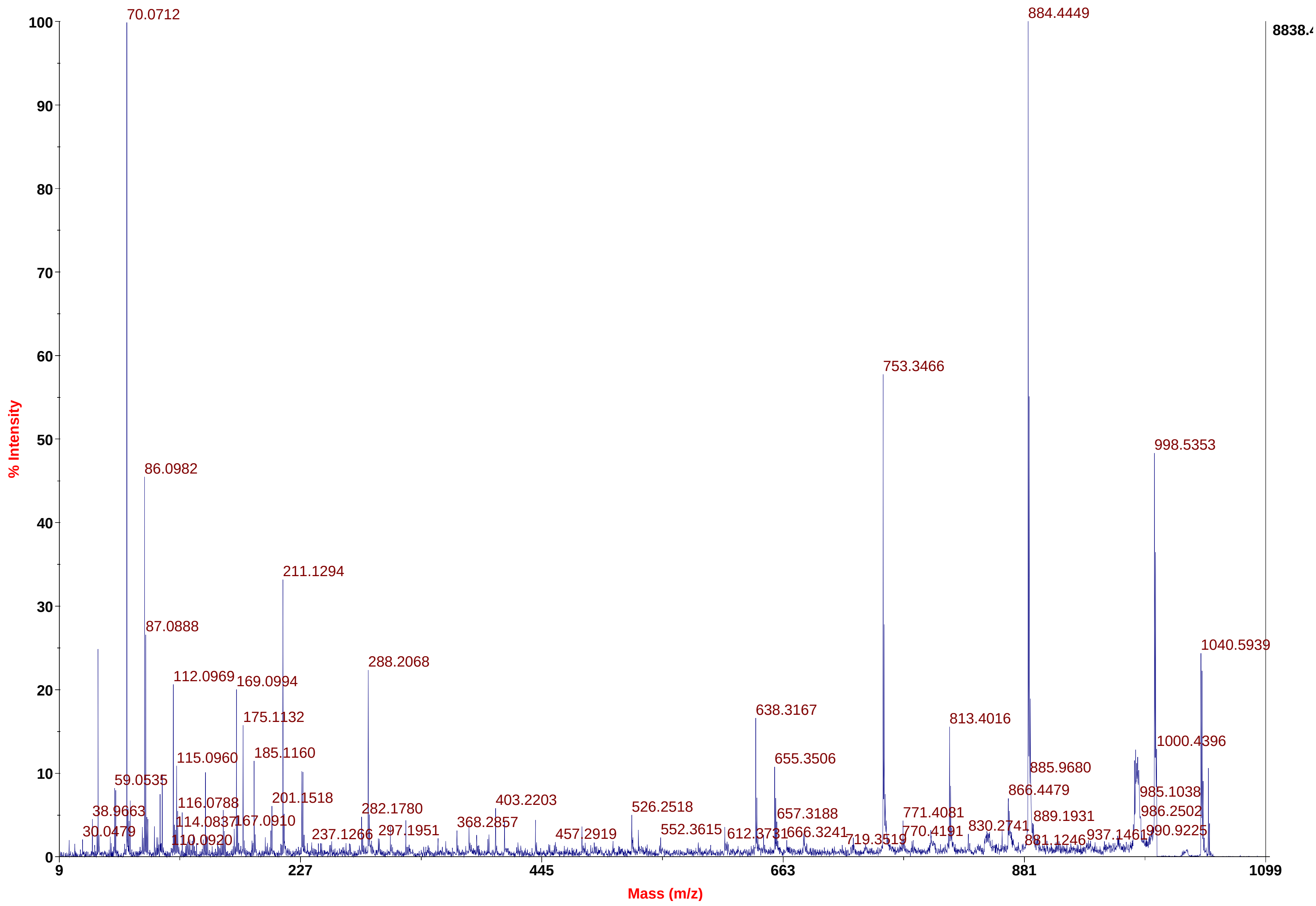

M19

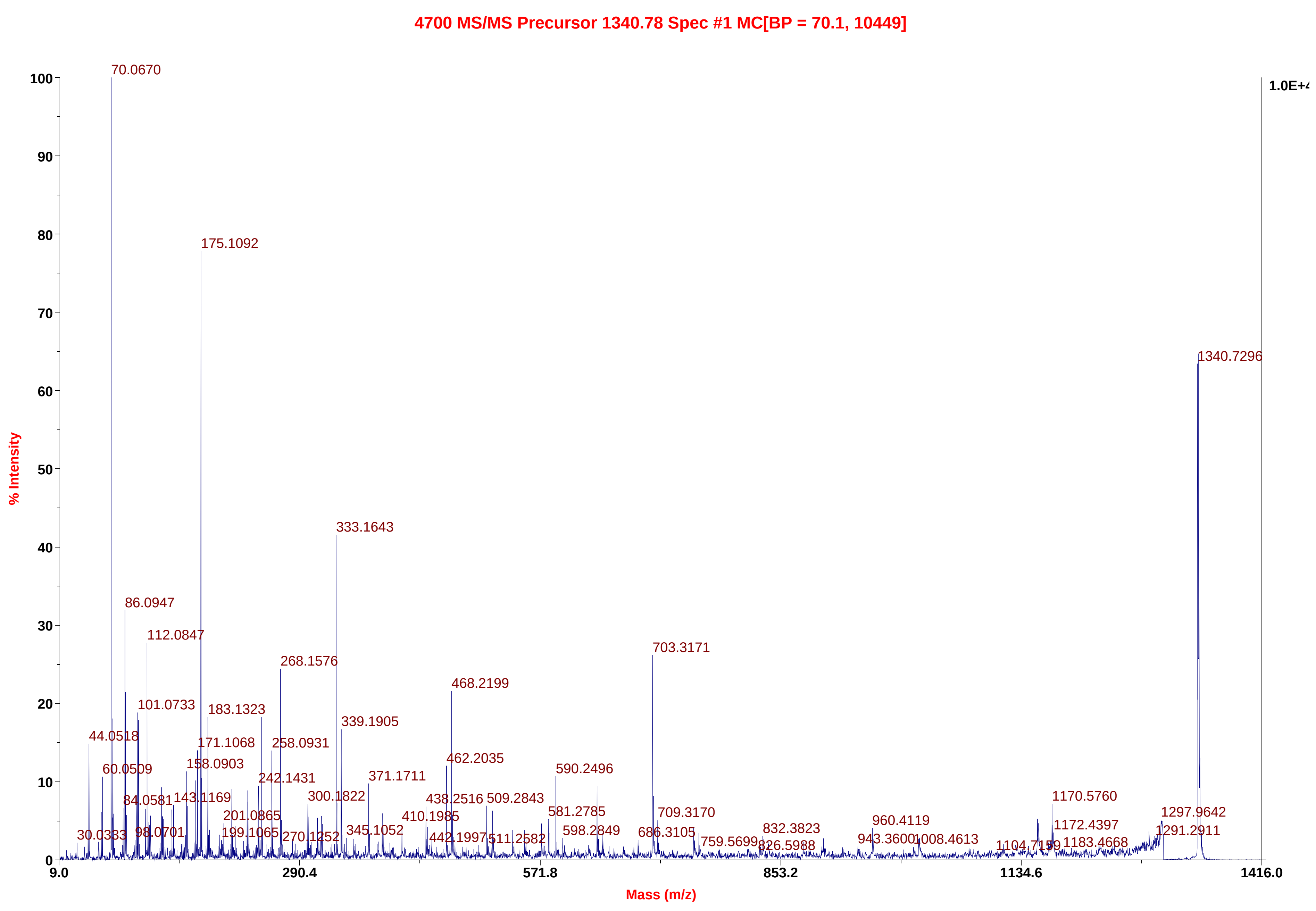
4700 MS/MS Precursor 1340.78 Spec #1 MC[BP = 70.1, 10449]
% Intensity
Mass (m/z)
1.0E+4
70.0670
175.1092
333.1643
86.0947
112.0847
268.1576
703.3171
468.2199
101.0733
183.1323
339.1905
44.0518
171.1068
258.0931
60.0509
158.0903
462.2035
590.2496
242.1431
371.1711
84.0581
143.1169
300.1822
438.2516
509.2843
1170.5760
201.0865
410.1985
581.2785
709.3170
1297.9642
960.4119
1172.4397
30.0333
98.0701
199.1065
270.1252
345.1052
442.1997
511.2582
598.2849
686.3105
832.3823
759.5699
826.5988
943.3600
1008.4613
1104.7159
1183.4668
1291.2911
1340.7296
9.0
290.4
571.8
853.2
1134.6
1416.0

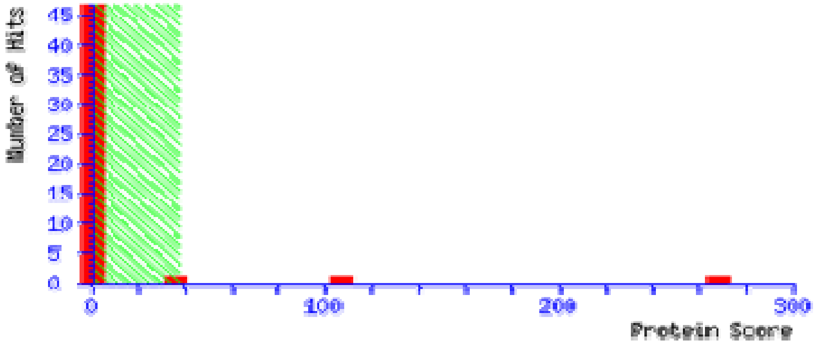

Peptide Summary Report

Format As: Peptide Summary — Help

Significance threshold p< 0.05 — Max. number of hits AUTO

Standard scoring — MudPIT scoring — Ions score or expect cut-off 0 — Show sub-sets 0

Show pop-ups — Suppress pop-ups — Sort unassigned Decreasing Score — Require bold red

Select All — Select None — Search Selected — Error tolerant

1. Q3V2G1_MOUSE **Mass:** 30666 **Score:** 268 **Matches:** 12(9) **Sequences:** 12(9) **emPAI:** 5.37

Adult male small intestine cDNA, RIKEN full-length enriched library, clone:2010012G12 product:apolipoprotein

Check to include this hit in error tolerant search

Query	Observed	Mr(expt)	Mr(calc)	ppm	Miss	Score	Expect	Rank	Unique	Peptide
2	1040.6257	1039.6184	1039.5774	39.5	0	17	2.7	1	U	K.ARPALEDLR.H
3	1047.5887	1046.5814	1046.5396	39.9	0	31	0.24	1	U	R.LSPVAEEFR.D
4	1099.6685	1098.6612	1098.6145	42.5	1	39	0.019	1	U	R.QKLQELQGR.L
8	1237.6746	1236.6673	1236.6139	43.2	1	64	0.0001	1	U	K.WKEDVELYR.Q
10	1266.6832	1265.6759	1265.6292	36.9	0	40	0.029	1	U	K.VQPYLDEFQK.K
11	1297.6907	1296.6834	1296.6245	45.5	0	45	0.01	1	U	R.TQLAPHSEQMR.E
12	1318.7311	1317.7238	1317.6677	42.6	1	23	1.6	1	U	R.LSPVAEEFRDR.M
13	1331.6930	1330.6857	1330.6266	44.5	0	56	0.00071	1	U	K.SNPTLNEYHTR.A
14	1340.7750	1339.7677	1339.7095	43.4	0	48	0.0033	1	U	K.VAPLGAELQESAR.Q
16	1394.7819	1393.7746	1393.7242	36.2	1	38	0.041	1	U	K.VQPYLDEFQKK.W
17	1467.8397	1466.8324	1466.7769	37.8	1	86	5.6e-07	1	U	K.VKDFANVYVDAVK.D
23	1852.9277	1851.9204	1851.8428	41.9	1	80	3.4e-06	1	U	R.DFWDNLEKETDWVR.Q

Protein View

Match to: Q3V2G1_MOUSE Score: 268
Adult male small intestine cDNA, RIKEN full-length enriched library, clone:2010012G12 product:apolipoprotein A-I,
Found in search of ppw_C6_129990871602.txt

Nominal mass (M_r): **30666**; Calculated pI value: **5.64**
NCBI BLAST search of Q3V2G1_MOUSE against nr
Unformatted sequence string for pasting into other applications

Taxonomy: Mus musculus
Links to retrieve other entries containing this sequence from NCBI Entrez:
(no taxonomy information for this entry)

Variable modifications: Carbamidomethyl (C),Deamidated (NQ),Oxidation (M)
Cleavage by Trypsin: cuts C-term side of KR unless next residue is P
Sequence Coverage: **42%**

Matched peptides shown in **Bold Red**

```
  1 MKAVVLAVAL VFLTGRQAWH VWQQDEPQSQ WDKVKDFANV YVDAVKDSGR
 51 DYVSQFESSS LGQQLNLNLL ENWDTLGSTV SQLQERLGPL TRDFWDNLEK
101 ETDWVRQEMN KDLEEVKQKV QPYLDEFQKK WKEDVELYRQ KVAPLGAELQ
151 ESARQKLQEL QGRLSPVAEE FRDRMRTHVD SLRTQLAPHS EQMRESLAQR
201 LAELKSNPTL NEYHTRAKTH LKTLGEKARP ALEDLRHSLM PMLETLKTQV
251 QSVIDKASET LTAQ
```

Show predicted peptides also

Sort Peptides By ◉ Residue Number ○ Increasing Mass ○ Decreasing Mass

Start - End	Observed	Mr(expt)	Mr(calc)	ppm	Miss	Sequence
34 - 46	1467.8397	1466.8324	1466.7769	38	1	K.VKDFANVYVDAVK.D (Ions score 86)
93 - 106	1852.9277	1851.9204	1851.8428	42	1	R.DFWDNLEKETDWVR.Q (Ions score 80)
120 - 129	1266.6832	1265.6759	1265.6292	37	0	K.VQPYLDEFQK.K (Ions score 40)
120 - 130	1394.7819	1393.7746	1393.7242	36	1	K.VQPYLDEFQKK.W (Ions score 38)
131 - 139	1237.6746	1236.6673	1236.6139	43	1	K.WKEDVELYR.Q (Ions score 64)
142 - 154	1340.7750	1339.7677	1339.7095	43	0	K.VAPLGAELQESAR.Q (Ions score 48)
155 - 163	1099.6685	1098.6612	1098.6145	43	1	R.QKLQELQGR.L (Ions score 39)
164 - 172	1047.5887	1046.5814	1046.5396	40	0	R.LSPVAEEFR.D (Ions score 31)
164 - 174	1318.7311	1317.7238	1317.6677	43	1	R.LSPVAEEFRDR.M (Ions score 23)
184 - 194	1297.6907	1296.6834	1296.6245	45	0	R.TQLAPHSEQMR.E (Ions score 45)
206 - 216	1331.6930	1330.6857	1330.6266	44	0	K.SNPTLNEYHTR.A (Ions score 56)
228 - 236	1040.6257	1039.6184	1039.5774	39	0	K.ARPALEDLR.H (Ions score 17)

Sample M20

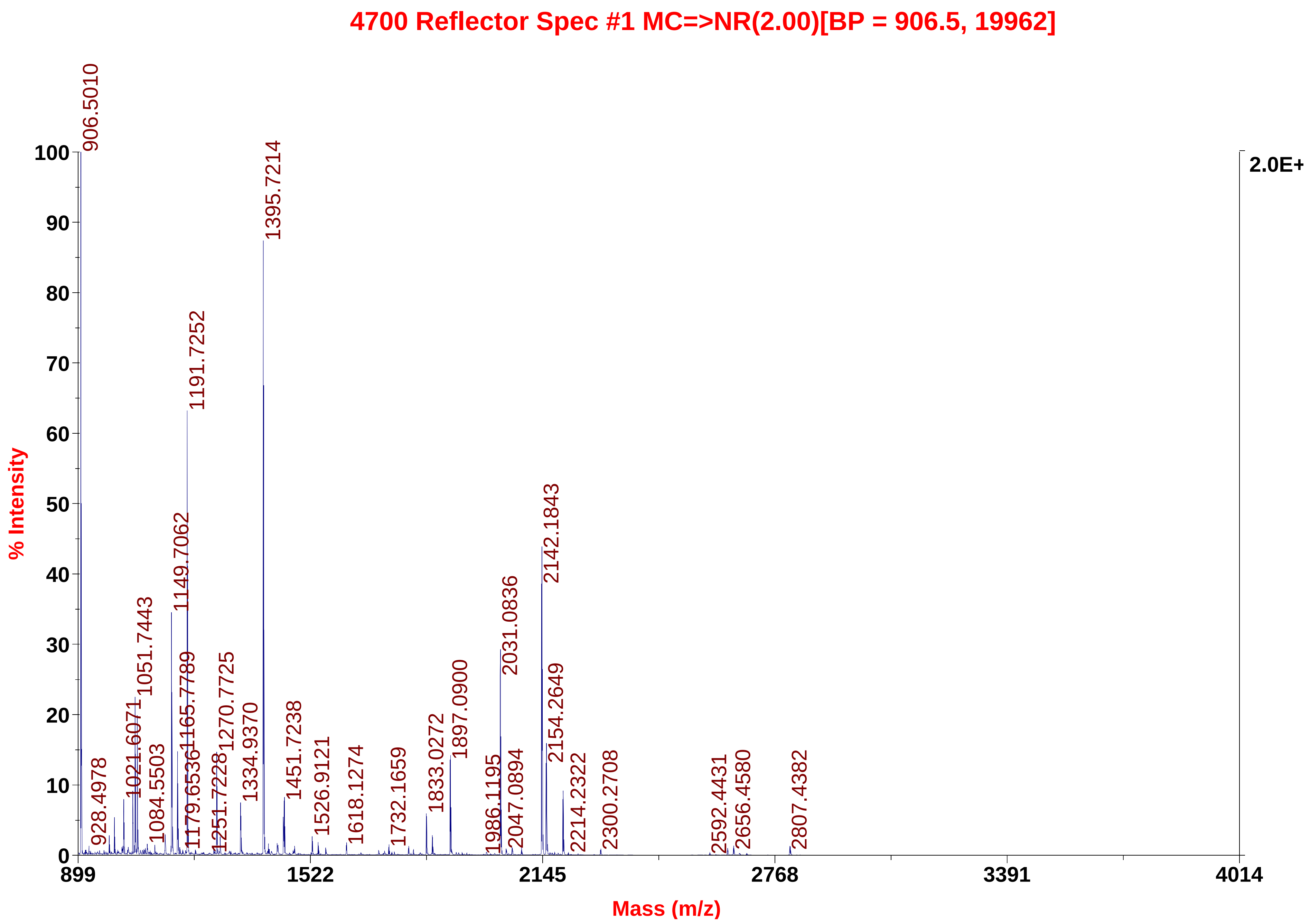

4700 Reflector Spec #1 MC=>NR(2.00)[BP = 906.5, 19962]
% Intensity
Mass (m/z)
2.0E+
906.5010
928.4978
1021.6071
1051.7443
1084.5503
1149.7062
1165.7789
1179.6536
1191.7252
1251.7228
1270.7725
1334.9370
1395.7214
1451.7238
1526.9121
1618.1274
1732.1659
1833.0272
1897.0900
1986.1195
2031.0836
2047.0894
2142.1843
2154.2649
2214.2322
2300.2708
2592.4431
2656.4580
2807.4382

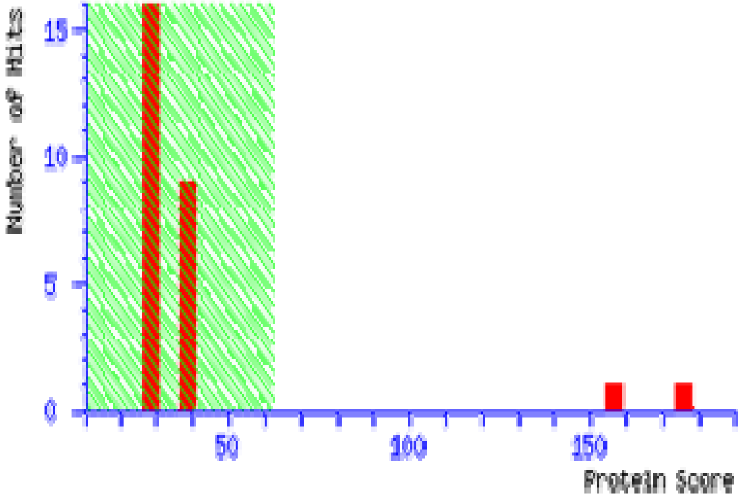

Concise Protein Summary Report

Format As | Concise Protein Summary | Help

Significance threshold p< 0.05 Max. number of hits AUTO

Re-Search All | Search Unmatched

1. PRDX6_MOUSE **Mass:** 24724 **Score:** 176 **Expect:** 2.3e-13 **Matches:** 30
Peroxiredoxin-6 (EC 1.11.1.15) (Antioxidant protein 2) (1-Cys peroxiredoxin) (1-Cys PRX) (Acidic calcium-independent p
Q53ZU7_MOUSE **Mass:** 24855 **Score:** 176 **Expect:** 2.3e-13 **Matches:** 30
Peroxiredoxin 6 (CRL-1722 L5178Y-R cDNA, RIKEN full-length enriched library, clone:I730069G09 product:peroxiredoxin 6,
AAD03716 **Mass:** 24825 **Score:** 162 **Expect:** 5.8e-12 **Matches:** 28
MMCYSP4 NID: - Mus musculus
AAC67553 **Mass:** 24885 **Score:** 148 **Expect:** 1.5e-10 **Matches:** 28
AF093853 NID: - Mus musculus

Protein View

Match to: **PRDX6_MOUSE** Score: **176** Expect: **2.3e-13**
Peroxiredoxin-6 (EC 1.11.1.15) (Antioxidant protein 2) (1-Cys peroxiredoxin) (1-Cys PRX) (Acidic calci
Found in search of pmf_D6_129990866103.txt

Nominal mass (M_r): **24724**; Calculated pI value: **5.72**
NCBI BLAST search of PRDX6_MOUSE against nr
Unformatted sequence string for pasting into other applications

Taxonomy: Mus musculus

Variable modifications: Carbamidomethyl (C),Deamidated (NQ),Oxidation (M)
Cleavage by Trypsin: cuts C-term side of KR unless next residue is P
Number of mass values searched: **119**
Number of mass values matched: **30**
Sequence Coverage: **88%**

Matched peptides shown in **Bold Red**

```
  1 PGGLLLGDEA PNFEANTTIG RIRFHDFLGD SWGILFSHPR DFTPVCTTEL
 51 GRAAKLAPEF AKRNVKLIAL SIDSVEDHLA WSKDINAYNG ETPTEKLPFP
101 IIDDKGRDLA ILLGMLDPVE KDDNNMPVTA RVVFIFGPDK KLKLSILYPA
151 TTGRNFDEIL RVVDSLQLTG TKPVATPVDW KKGESVMVVP TLSEEEAKQC
201 FPKGVFTKEL PSGKKYLRYT PQP
```

Show predicted peptides also

Sort Peptides By ◉ Residue Number ○ Increasing Mass ○ Decreasing Mass

Start - End	Observed	Mr(expt)	Mr(calc)	ppm	Miss	Sequence	
1 - 21	2142.1814	2141.1741	2141.0753	46	0	-.PGGLLLGDEAPNFEANTTIGR.I	
22 - 40	2300.2776	2299.2703	2299.1651	46	1	R.IRFHDFLGDSWGILFSHPR.D	
24 - 40	2031.0808	2030.0735	2029.9799	46	0	R.FHDFLGDSWGILFSHPR.D	
41 - 52	1338.7070	1337.6997	1337.6286	53	0	R.DFTPVCTTELGR.A	
41 - 52	1395.7209	1394.7136	1394.6500	46	0	R.DFTPVCTTELGR.A	Carbamidomethyl (C
53 - 62	1045.6050	1044.5977	1044.5967	1	1	R.AAKLAPEFAK.R	
64 - 83	2239.2366	2238.2293	2238.1896	18	1	R.NVKLIALSIDSVEDHLAWSK.D	Deamidated
67 - 83	1897.0906	1896.0833	1895.9993	44	0	K.LIALSIDSVEDHLAWSK.D	
84 - 96	1451.7247	1450.7174	1450.6576	41	0	K.DINAYNGETPTEK.L	
97 - 105	1057.6310	1056.6237	1056.5855	36	0	K.LPFPIIDDK.G	
97 - 107	1270.7704	1269.7631	1269.7081	43	1	K.LPFPIIDDKGR.D	
106 - 121	1740.0472	1739.0399	1738.9651	43	1	K.GRDLAILLGMLDPVEK.D	
108 - 121	1526.9110	1525.9037	1525.8425	40	0	R.DLAILLGMLDPVEK.D	
108 - 121	1542.9082	1541.9009	1541.8375	41	0	R.DLAILLGMLDPVEK.D	Oxidation (M)

M20

4700 MS/MS Precursor 906.502 Spec #1 MC[BP = 175.1, 3389]

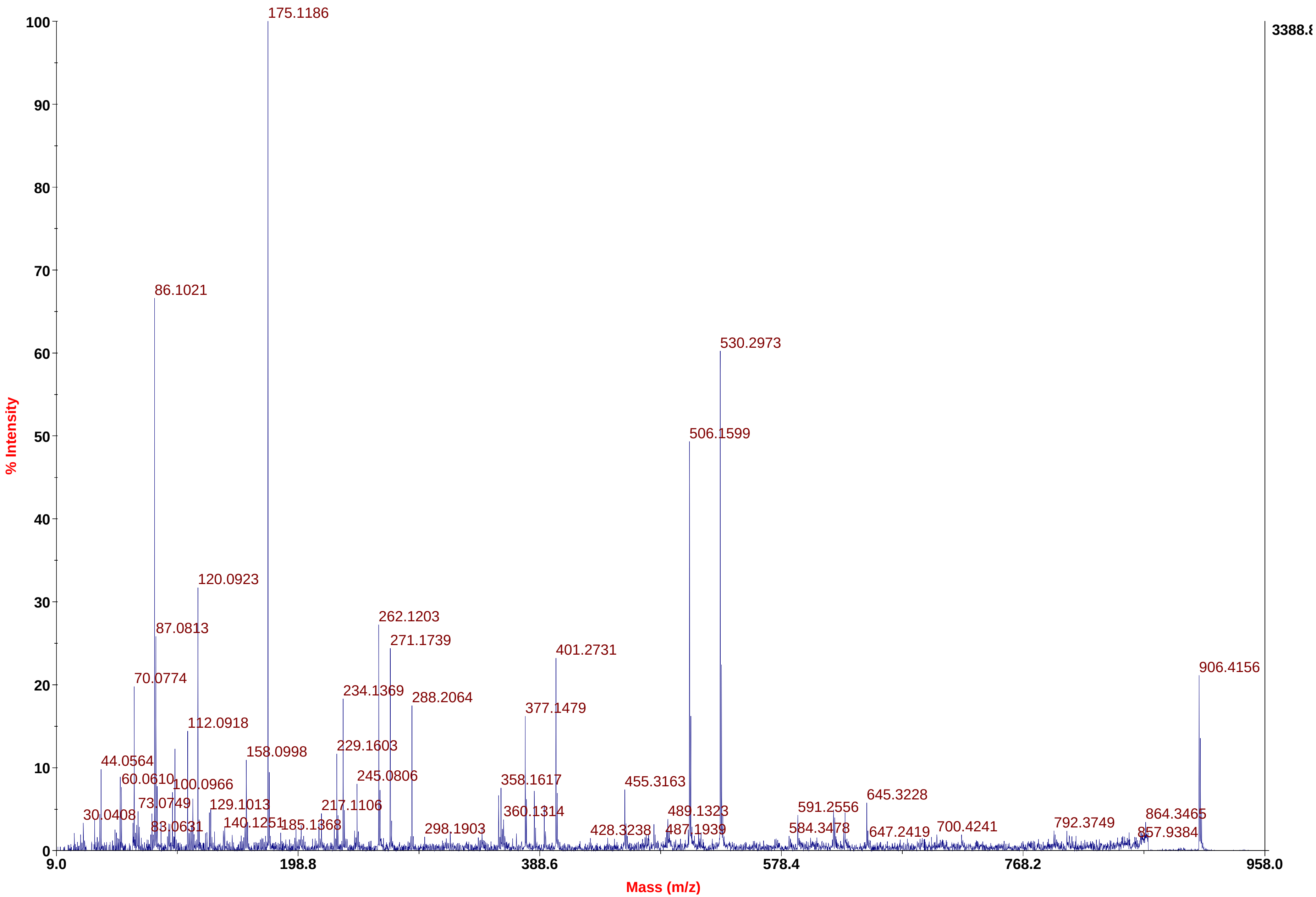

M20

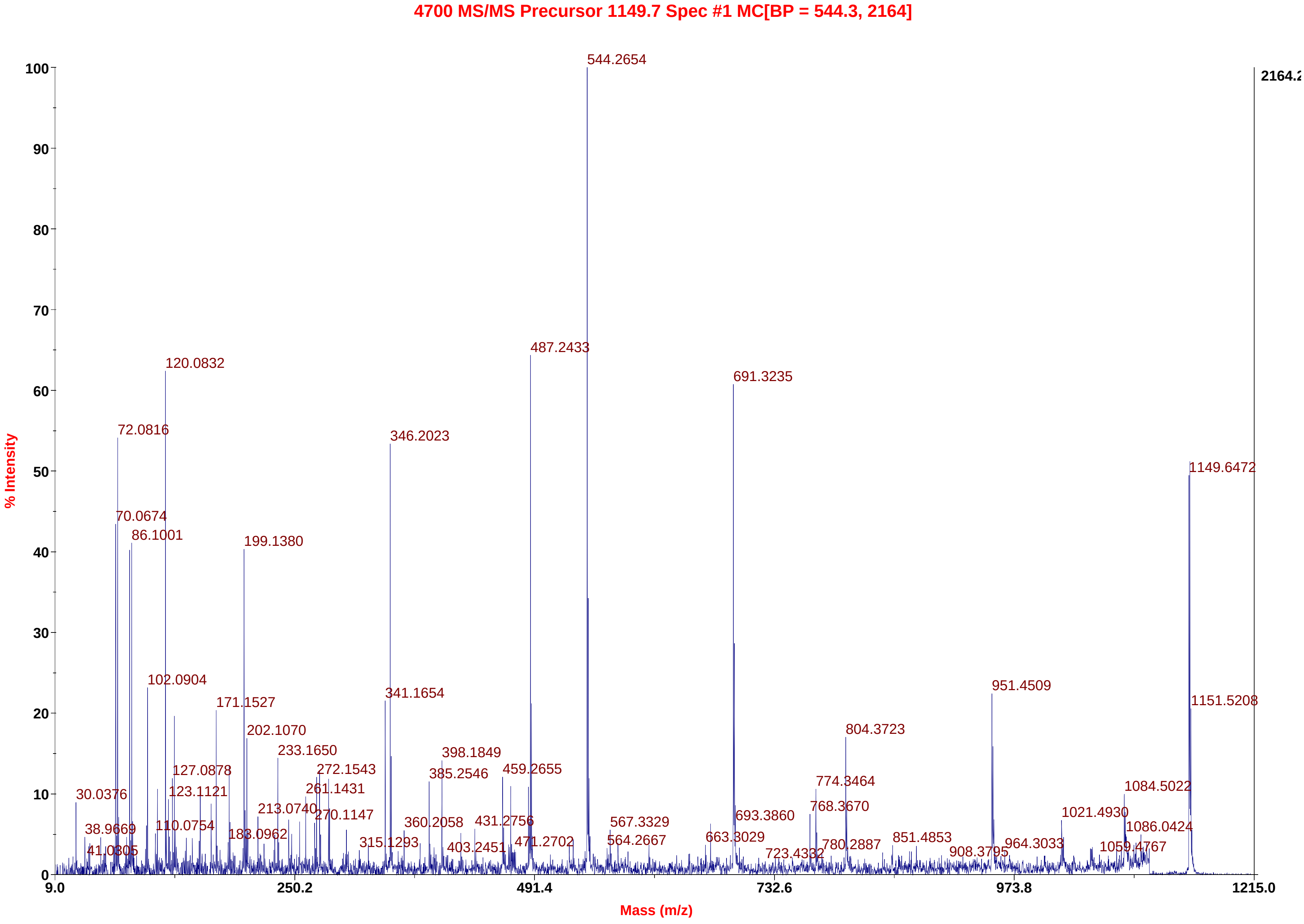
4700 MS/MS Precursor 1149.7 Spec #1 MC[BP = 544.3, 2164]
% Intensity
Mass (m/z)
2164.2
544.2654
487.2433
120.0832
691.3235
72.0816
346.2023
1149.6472
70.0674
86.1001
199.1380
102.0904
341.1654
951.4509
171.1527
1151.5208
202.1070
804.3723
233.1650
398.1849
127.0878
272.1543
459.2655
385.2546
774.3464
1084.5022
123.1121
261.1431
30.0376
213.0740
768.3670
1021.4930
270.1147
693.3860
360.2058
431.2756
567.3329
38.9669
110.0754
1086.0424
183.0962
663.3029
851.4853
315.1293
471.2702
564.2667
780.2887
964.3033
403.2451
1059.4767
908.3795
41.0305
723.4332
9.0
250.2
491.4
732.6
973.8
1215.0

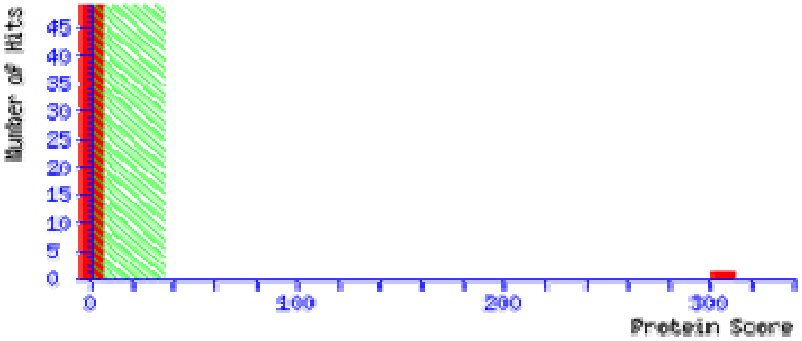

Peptide Summary Report

Format As | Peptide Summary | Help

Significance threshold p< 0.05 | Max. number of hits AUTO

Standard scoring | MudPIT scoring | Ions score or expect cut-off 0 | Show sub-sets 0

Show pop-ups | Suppress pop-ups | Sort unassigned Decreasing Score | Require bold red

Select All | Select None | Search Selected | Error tolerant

1. PRDX6_MOUSE **Mass:** 24724 **Score:** 306 **Matches:** 13(11) **Sequences:** 13(11) **emPAI:** 17.46

Peroxiredoxin-6 (EC 1.11.1.15) (Antioxidant protein 2) (1-Cys peroxiredoxin) (1-Cys PRX) (Acidic calcium-inde

Check to include this hit in error tolerant search

Query	Observed	Mr(expt)	Mr(calc)	ppm	Miss	Score	Expect	Rank	Unique	Peptide
1	906.5024	905.4952	905.4607	38.1	0	40	0.031	1	U	R.NFDEILR.V
4	1021.6072	1020.5999	1020.5644	34.8	0	43	0.0088	1	U	R.VVFIFGPDK.K
7	1057.6310	1056.6237	1056.5855	36.1	0	59	0.00024	1	U	K.LPFPIIDDK.G
8	1149.7048	1148.6975	1148.6594	33.2	1	35	0.044	1	U	R.VVFIFGPDKK.L
9	1191.7252	1190.7179	1190.6659	43.7	0	36	0.034	1	U	K.LSILYPATTGR.N
10	1270.7704	1269.7631	1269.7081	43.3	1	42	0.0098	1	U	K.LPFPIIDDKGR.D
12	1395.7209	1394.7136	1394.6500	45.6	0	71	2.3e-05	1	U	R.DFTPVCTTELGR.A + Carbamidometh
13	1451.7247	1450.7174	1450.6576	41.3	0	45	0.0095	1	U	K.DINAYNGETPTEK.L
15	1833.0288	1832.0215	1831.9237	53.4	1	24	0.94	1	U	K.KGESVMVVPTLSEEEAK.Q
19	1897.0906	1896.0833	1895.9993	44.3	0	72	1.2e-05	1	U	K.LIALSIDSVEDHLAWSK.D
26	2031.0808	2030.0735	2029.9799	46.1	0	30	0.28	1	U	R.FHDFLGDSWGILFSHPR.D
40	2142.1814	2141.1741	2141.0753	46.2	0	71	2.2e-05	1	U	-.PGGLLLGDEAPNFEANTTIGR.I
42	2154.2661	2153.2588	2153.1732	39.7	0	55	0.00047	1	U	R.VVDSLQLTGTKPVATPVDWK.K

Protein View

Match to: **PRDX6_MOUSE** Score: **306**
Peroxiredoxin-6 (EC 1.11.1.15) (Antioxidant protein 2) (1-Cys peroxiredoxin) (1-Cys PRX) (Acidic calcium-independent
Found in search of ppw_D6_129990871703.txt

Nominal mass (M_r): **24724**; Calculated pI value: **5.72**
NCBI BLAST search of PRDX6_MOUSE against nr
Unformatted sequence string for pasting into other applications

Taxonomy: Mus musculus

Variable modifications: Carbamidomethyl (C),Deamidated (NQ),Oxidation (M)
Cleavage by Trypsin: cuts C-term side of KR unless next residue is P
Sequence Coverage: 69%

Matched peptides shown in **Bold Red**

```
  1 PGGLLLGDEA PNFEANTTIG RIRFHDFLGD SWGILFSHPR DFTPVCTTEL
 51 GRAAKLAPEF AKRNVKLIAL SIDSVEDHLA WSKDINAYNG ETPTEKLPFP
101 IIDDKGRDLA ILLGMLDPVE KDDNNMPVTA RVVFIFGPDK KLKLSILYPA
151 TTGRNFDEIL RVVDSLQLTG TKPVATPVDW KKGESVMVVP TLSEEEAKQC
201 FPKGVFTKEL PSGKKYLRYT PQP
```

Show predicted peptides also

Sort Peptides By ◉ Residue Number ○ Increasing Mass ○ Decreasing Mass

Start - End	Observed	Mr(expt)	Mr(calc)	ppm	Miss	Sequence
1 - 21	2142.1814	2141.1741	2141.0753	46	0	-.PGGLLLGDEAPNFEANTTIGR.I (Ions score 71)
24 - 40	2031.0808	2030.0735	2029.9799	46	0	R.FHDFLGDSWGILFSHPR.D (Ions score 30)
41 - 52	1395.7209	1394.7136	1394.6500	46	0	R.DFTPVCTTELGR.A Carbamidomethyl (C) (Ions score
67 - 83	1897.0906	1896.0833	1895.9993	44	0	K.LIALSIDSVEDHLAWSK.D (Ions score 72)
84 - 96	1451.7247	1450.7174	1450.6576	41	0	K.DINAYNGETPTEK.L (Ions score 45)
97 - 105	1057.6310	1056.6237	1056.5855	36	0	K.LPFPIIDDK.G (Ions score 59)
97 - 107	1270.7704	1269.7631	1269.7081	43	1	K.LPFPIIDDKGR.D (Ions score 42)
132 - 140	1021.6072	1020.5999	1020.5644	35	0	R.VVFIFGPDK.K (Ions score 43)
132 - 141	1149.7048	1148.6975	1148.6594	33	1	R.VVFIFGPDKK.L (Ions score 35)
144 - 154	1191.7252	1190.7179	1190.6659	44	0	K.LSILYPATTGR.N (Ions score 36)
155 - 161	906.5024	905.4952	905.4607	38	0	R.NFDEILR.V (Ions score 40)
162 - 181	2154.2661	2153.2588	2153.1732	40	0	R.VVDSLQLTGTKPVATPVDWK.K (Ions score 55)
182 - 198	1833.0288	1832.0215	1831.9237	53	1	K.KGESVMVVPTLSEEEAK.Q (Ions score 24)

Sample M21

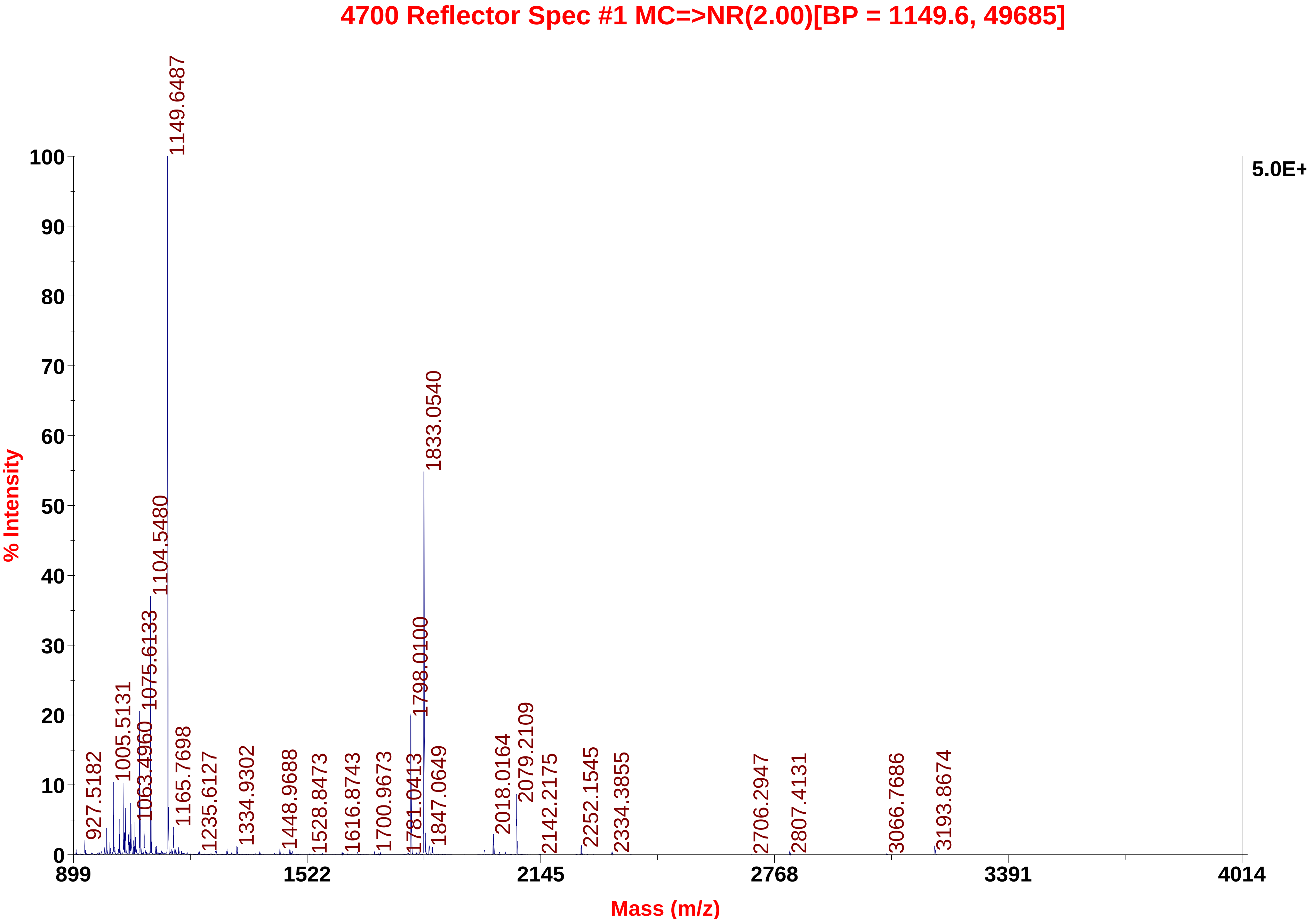
4700 Reflector Spec #1 MC=>NR(2.00)[BP = 1149.6, 49685]
1149.6487
1833.0540
1104.5480
1075.6133
1798.0100
1005.5131
1063.4960
927.5182
1165.7698
1235.6127
1334.9302
1448.9688
1528.8473
1616.8743
1700.9673
1781.0413
1847.0649
2018.0164
2079.2109
2142.2175
2252.1545
2334.3855
2706.2947
2807.4131
3066.7686
3193.8674
% Intensity
Mass (m/z)
5.0E+
100
90
80
70
60
50
40
30
20
10
0
899
1522
2145
2768
3391
4014

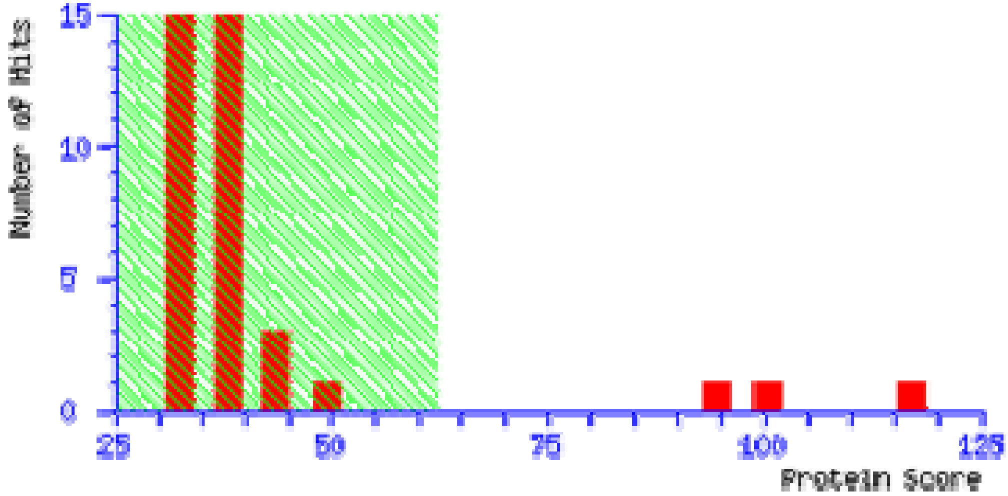

Concise Protein Summary Report

Format As | Concise Protein Summary | Help

Significance threshold p< 0.05 Max. number of hits AUTO

Re-Search All | Search Unmatched

1. JN0679 **Mass:** 23000 **Score:** 117 **Expect:** 1.8e-07 **Matches:** 18
heat shock protein 25 - mouse
S02143 **Mass:** 22868 **Score:** 106 **Expect:** 2.3e-06 **Matches:** 17
stress protein, 25K - mouse
CAB37341 **Mass:** 22838 **Score:** 94 **Expect:** 3.6e-05 **Matches:** 16
MURSPH NID: - Mus sp.
A53423 **Mass:** 22887 **Score:** 93 **Expect:** 4.6e-05 **Matches:** 15
HSP27-related protein (form a) - mouse

Protein View

Match to: **JN0679** Score: **117** Expect: **1.8e-07**
heat shock protein 25 - mouse
Found in search of pmf_E6_129990866104.txt

Nominal mass (M_r): **23000**; Calculated pI value: **6.12**
NCBI BLAST search of JN0679 against nr
Unformatted sequence string for pasting into other applications

Taxonomy: Mus musculus
Links to retrieve other entries containing this sequence from NCBI Entrez:
(no taxonomy information for this entry)
(no taxonomy information for this entry)
(no taxonomy information for this entry)
(no taxonomy information for this entry)
(no taxonomy information for this entry)
(no taxonomy information for this entry)
(no taxonomy information for this entry)
(no taxonomy information for this entry)
(no taxonomy information for this entry)

Variable modifications: Carbamidomethyl (C),Deamidated (NQ),Oxidation (M)
Cleavage by Trypsin: cuts C-term side of KR unless next residue is P
Number of mass values searched: **110**
Number of mass values matched: **18**
Sequence Coverage: **71%**

Matched peptides shown in **Bold Red**

```
  1 MTERRVPFSL LRSPSWEPFR DWYPAHSRLF DQAFGVPRLP DEWSQWFSAA
 51 GWPGYVRPLP AATAEGPAAV TLAAPAFSRA LNRQLSSGVS EIRQTADRWR
101 VSLDVNHFAP EELTVKTKEG VVEITGKHEE RQDEHGYISR CFTRKYTLPP
151 GVDPTLVSSS LSPEGTLTVE APLPKAVTQS AEITIPVTFE ARAQIGGPEA
201 GKSEQSGAK
```

Show predicted peptides also

Sort Peptides By ◉ Residue Number ○ Increasing Mass ○ Decreasing Mass

Start - End	Observed	Mr(expt)	Mr(calc)	ppm	Miss	Sequence
5 - 12	987.6417	986.6344	986.6025	32	1	R.RVPFSLLR.S
6 - 20	1818.0321	1817.0248	1816.9624	34	1	R.VPFSLLRSPSWEPFR.D
13 - 20	1005.5128	1004.5055	1004.4716	34	0	R.SPSWEPFR.D
13 - 28	2018.0139	2017.0066	2016.9231	41	1	R.SPSWEPFRDWYPAHSR.L
21 - 28	1031.5046	1030.4973	1030.4621	34	0	R.DWYPAHSR.L
29 - 38	1149.6465	1148.6392	1148.5979	36	0	R.LFDQAFGVPR.L
84 - 93	1075.6113	1074.6040	1074.5669	35	0	R.QLSSGVSEIR.Q
101 - 116	1798.0094	1797.0021	1796.9309	40	0	R.VSLDVNHFAPEELTVK.T
117 - 127	1160.6812	1159.6739	1159.6449	25	1	K.TKEGVVEITGK.H
119 - 127	931.5301	930.5228	930.5022	22	0	K.EGVVEITGK.H

M21

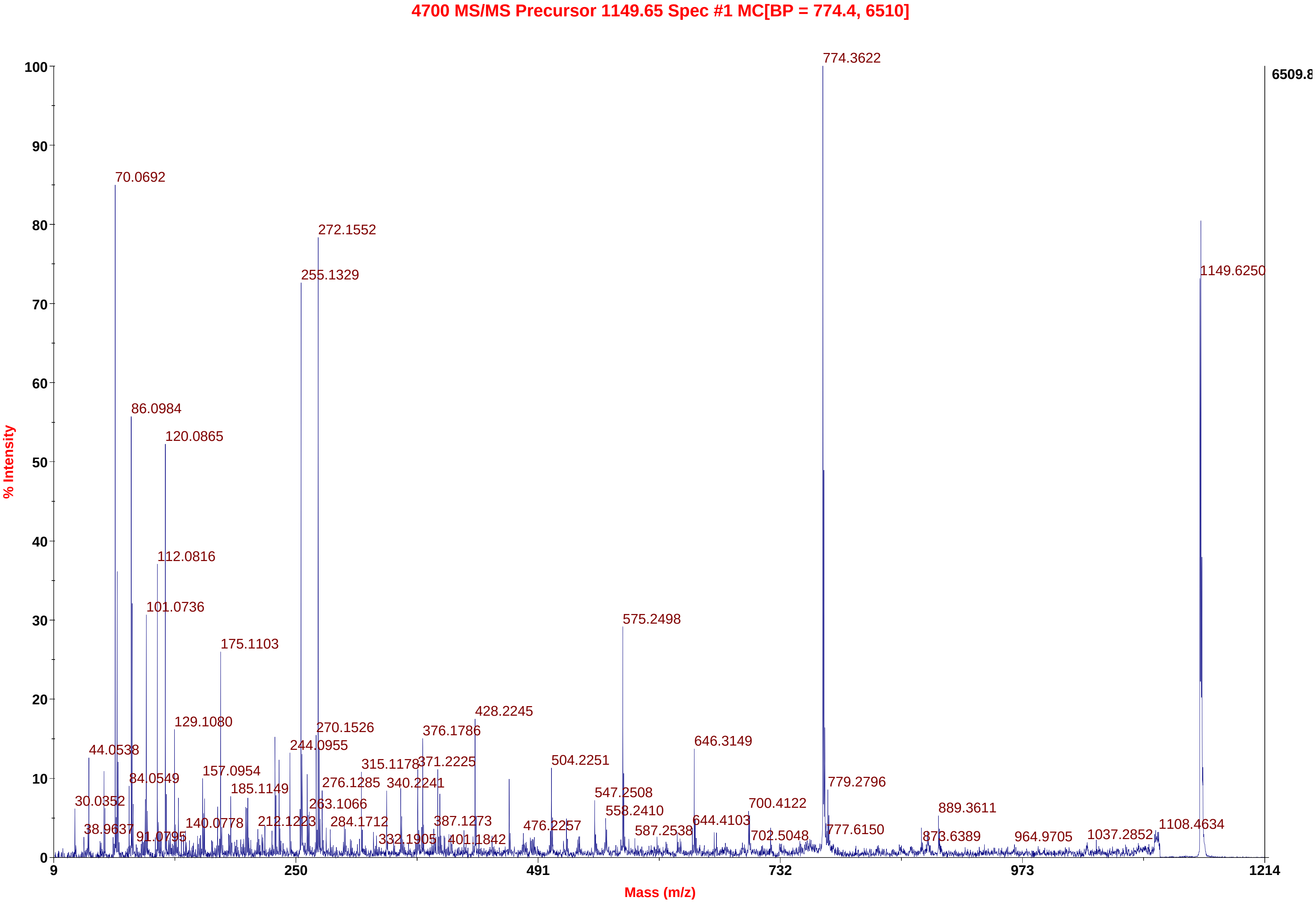
4700 MS/MS Precursor 1149.65 Spec #1 MC[BP = 774.4, 6510]
% Intensity
Mass (m/z)
6509.8
774.3622
70.0692
272.1552
255.1329
1149.6250
86.0984
120.0865
112.0816
101.0736
575.2498
175.1103
428.2245
129.1080
270.1526
376.1786
646.3149
44.0538
244.0955
315.1178
371.2225
504.2251
157.0954
84.0549
185.1149
276.1285
340.2241
779.2796
547.2508
30.0352
263.1066
558.2410
700.4122
889.3611
140.0778
212.1223
284.1712
387.1273
644.4103
476.2257
38.9637
587.2538
777.6150
1108.4634
91.0795
332.1905
401.1842
702.5048
873.6389
964.9705
1037.2852
0
10
20
30
40
50
60
70
80
90
100
9
250
491
732
973
1214

M21

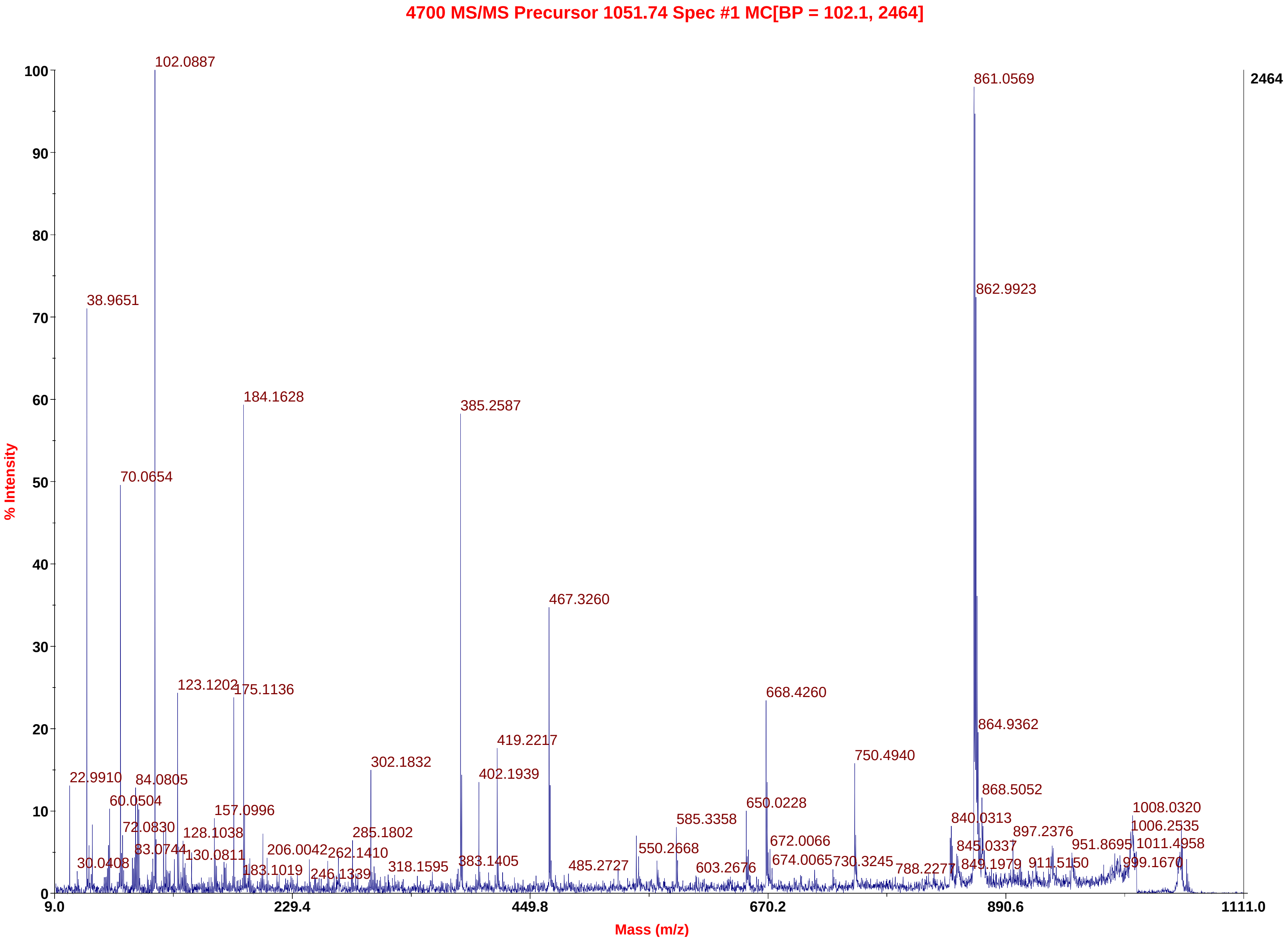
4700 MS/MS Precursor 1051.74 Spec #1 MC[BP = 102.1, 2464]
% Intensity
Mass (m/z)
2464
102.0887
861.0569
862.9923
38.9651
184.1628
385.2587
70.0654
467.3260
123.1202
175.1136
668.4260
864.9362
419.2217
302.1832
750.4940
22.9910
84.0805
402.1939
868.5052
60.0504
157.0996
650.0228
1008.0320
72.0830
128.1038
585.3358
840.0313
1006.2535
897.2376
83.0744
206.0042
262.1410
285.1802
550.2668
672.0066
845.0337
951.8695
1011.4958
30.0408
130.0811
183.1019
246.1339
318.1595
383.1405
485.2727
603.2676
674.0065
730.3245
788.2277
849.1979
911.5150
999.1670
9.0
229.4
449.8
670.2
890.6
1111.0

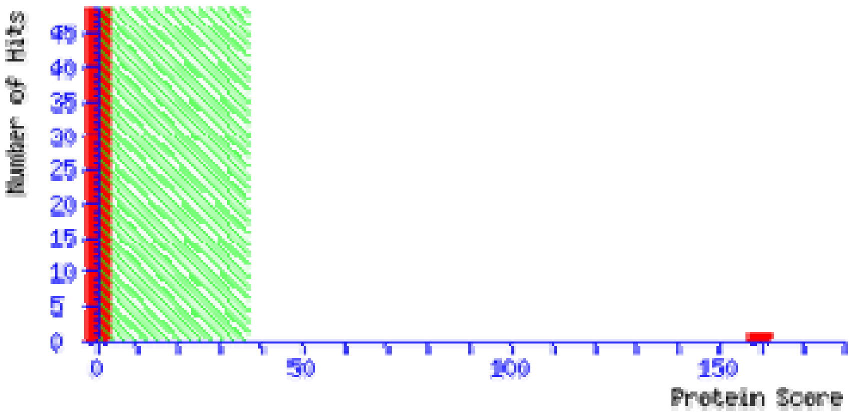

Peptide Summary Report

Format As | Peptide Summary | Help

Significance threshold p< 0.05 | Max. number of hits AUTO

Standard scoring ○ MudPIT scoring ● | Ions score or expect cut-off 0 | Show sub-sets 0

Show pop-ups ● Suppress pop-ups ○ | Sort unassigned Decreasing Score | Require bold red ☐

Select All | Select None | Search Selected | ☐ **Error tolerant**

1. JN0679 **Mass:** 23000 **Score:** 160 **Matches:** 9(5) **Sequences:** 9(5) **emPAI:** 2.46
heat shock protein 25 - mouse
☐ Check to include this hit in error tolerant search

	Query	Observed	Mr(expt)	Mr(calc)	ppm	Miss	Score	Expect	Rank	Unique	Peptide
☑	1	927.5178	926.5105	926.4821	30.7	0	36	0.051	1	U	R.AQIGGPEAGK.S
☑	2	987.6417	986.6344	986.6025	32.4	1	18	2.1	1	U	R.RVPFSLLR.S
☑	6	1031.5046	1030.4973	1030.4621	34.2	0	22	2.3	1	U	R.DWYPAHSR.L
☑	9	1075.6113	1074.6040	1074.5669	34.5	0	46	0.0067	1	U	R.QLSSGVSEIR.Q
☑	12	1149.6465	1148.6392	1148.5979	36.0	0	62	0.00016	1	U	R.LFDQAFGVPR.L
☑	14	1798.0094	1797.0021	1796.9309	39.7	0	68	3.6e-05	1	U	R.VSLDVNHFAPEELTVK.T

Protein View

Match to: **JN0679** Score: **160**
heat shock protein 25 - mouse
Found in search of ppw_E6_129990871804.txt

Nominal mass (M_r): **23000**; Calculated pI value: **6.12**
NCBI BLAST search of JN0679 against nr
Unformatted sequence string for pasting into other applications

Taxonomy: Mus musculus
Links to retrieve other entries containing this sequence from NCBI Entrez:
(no taxonomy information for this entry)
(no taxonomy information for this entry)
(no taxonomy information for this entry)
(no taxonomy information for this entry)
(no taxonomy information for this entry)
(no taxonomy information for this entry)
(no taxonomy information for this entry)
(no taxonomy information for this entry)
(no taxonomy information for this entry)

Variable modifications: Carbamidomethyl (C),Deamidated (NQ),Oxidation (M)
Cleavage by Trypsin: cuts C-term side of KR unless next residue is P
Sequence Coverage: **56%**

Matched peptides shown in **Bold Red**

1 MTER**RVPFSL LRSPSWEPFR DWYPAHSRLF DQAFGVPR**LP DEWSQWFSAA
51 GWPGYVRPLP AATAEGPAAV TLAAPAFSRA LNR**QLSSGVS EIR**QTADRWR
101 **VSLDVNHFAP EELTVK**TKEG VVEITGKHEE RQDEHGYISR CFTR**KYTLPP**
151 **GVDPTLVSSS LSPEGTLTVE APLPKAVTQS AEITIPVTFE ARAQIGGPEA**
201 **GK**SEQSGAK

Show predicted peptides also

Sort Peptides By ◉ Residue Number ○ Increasing Mass ○ Decreasing Mass

Start – End	Observed	Mr(expt)	Mr(calc)	ppm	Miss	Sequence	
5 – 12	987.6417	986.6344	986.6025	32	1	R.RVPFSLLR.S	(Ions score 18)
13 – 28	2018.0139	2017.0066	2016.9231	41	1	R.SPSWEPFRDWYPAHSR.L	(Ions score 14)
21 – 28	1031.5046	1030.4973	1030.4621	34	0	R.DWYPAHSR.L	(Ions score 22)
29 – 38	1149.6465	1148.6392	1148.5979	36	0	R.LFDQAFGVPR.L	(Ions score 62)
84 – 93	1075.6113	1074.6040	1074.5669	35	0	R.QLSSGVSEIR.Q	(Ions score 46)
101 – 116	1798.0094	1797.0021	1796.9309	40	0	R.VSLDVNHFAPEELTVK.T	(Ions score 68)
145 – 175	3193.8574	3192.8501	3192.7170	42	1	R.KYTLPPGVDPTLVSSSLSPEGTLTVEAPLPK.A	(
176 – 192	1833.0530	1832.0457	1831.9680	42	0	K.AVTQSAEITIPVTFEAR.A	(Ions score 69)
193 – 202	927.5178	926.5105	926.4821	31	0	R.AQIGGPEAGK.S	(Ions score 36)

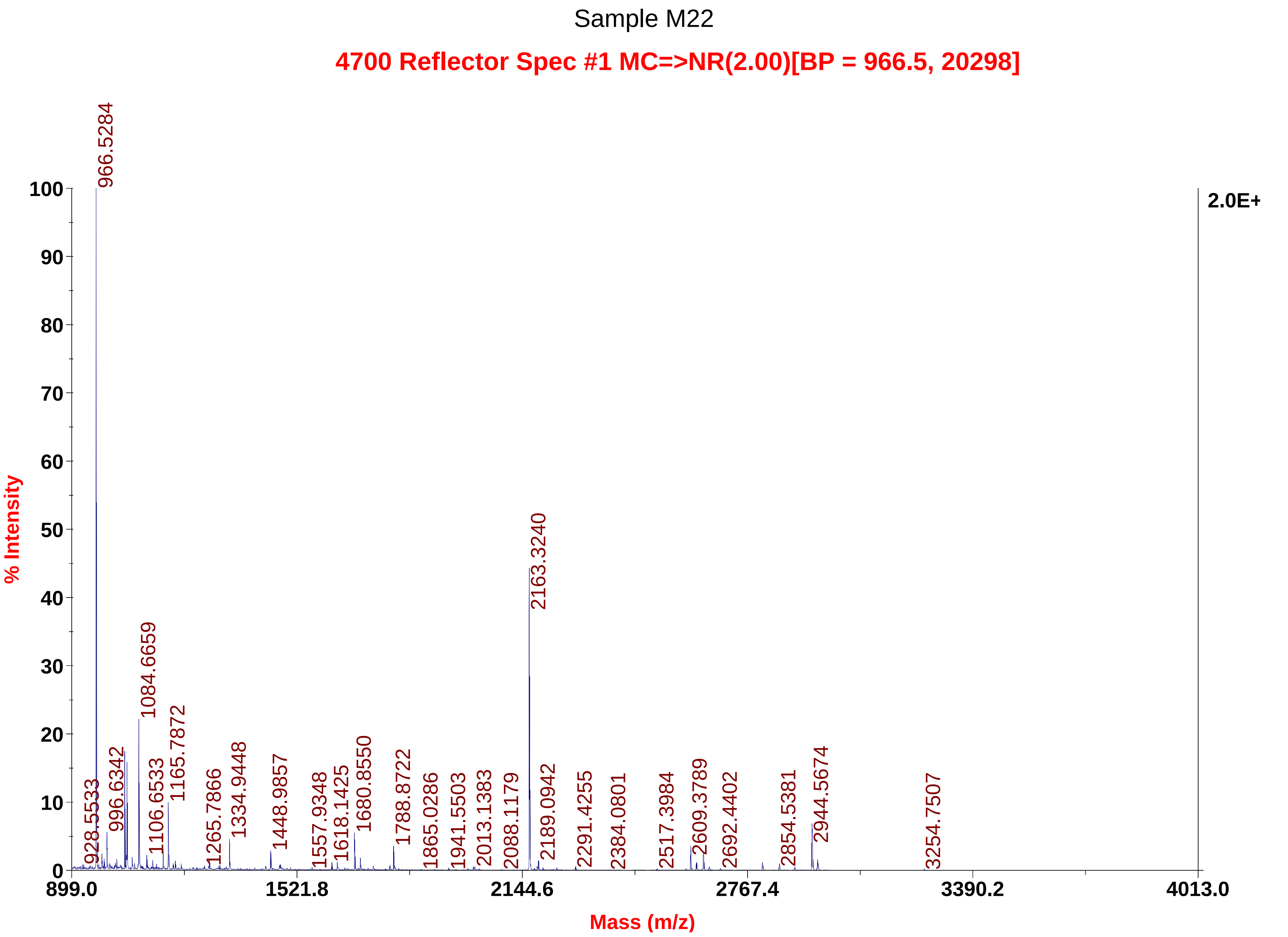
Sample M22
4700 Reflector Spec #1 MC=>NR(2.00)[BP = 966.5, 20298]
% Intensity
Mass (m/z)
2.0E+
100
90
80
70
60
50
40
30
20
10
0
899.0
1521.8
2144.6
2767.4
3390.2
4013.0
928.5533
966.5284
996.6342
1084.6659
1106.6533
1165.7872
1265.7866
1334.9448
1448.9857
1557.9348
1618.1425
1680.8550
1788.8722
1865.0286
1941.5503
2013.1383
2088.1179
2163.3240
2189.0942
2291.4255
2384.0801
2517.3984
2609.3789
2692.4402
2854.5381
2944.5674
3254.7507

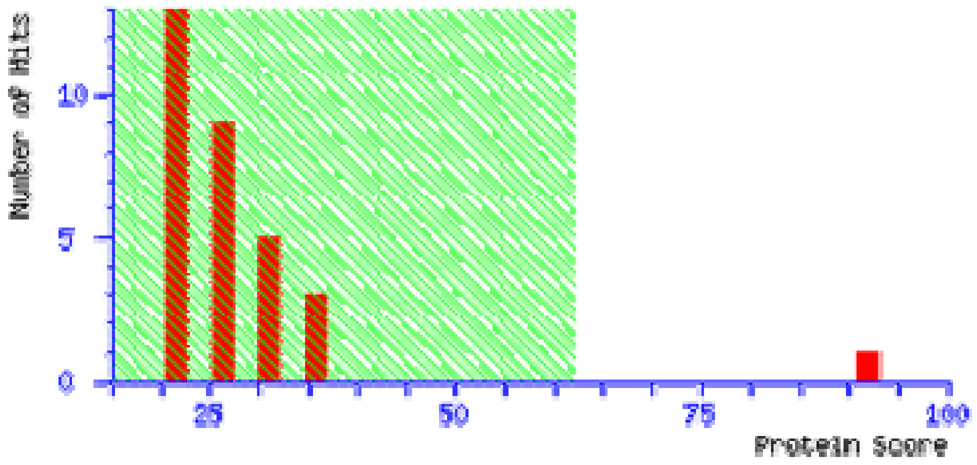

Concise Protein Summary Report

Format As | Concise Protein Summary | Help

Significance threshold p< 0.05 Max. number of hits AUTO

Re-Search All | Search Unmatched

1. GDIS_MOUSE **Mass:** 22705 **Score:** 92 **Expect:** 6.1e-05 **Matches:** 17
   Rho GDP-dissociation inhibitor 2 (Rho GDI 2) (Rho-GDI beta) (D4).- Mus musculus (Mouse
   I49687 **Mass:** 22836 **Score:** 92 **Expect:** 6.2e-05 **Matches:** 17
   GDP-dissociation inhibitor - mouse

Protein View

Match to: **GDIS_MOUSE** Score: **92** Expect: **6.1e-05**
Rho GDP-dissociation inhibitor 2 (Rho GDI 2) (Rho-GDI beta) (D4).- Mus musculus (Mouse).
Found in search of pmf_F6_12990866105.txt

Nominal mass (M_r): **22705**; Calculated pI value: **4.97**
NCBI BLAST search of GDIS_MOUSE against nr
Unformatted sequence string for pasting into other applications

Taxonomy: Mus musculus

Variable modifications: Carbamidomethyl (C),Deamidated (NQ),Oxidation (M)
Cleavage by Trypsin: cuts C-term side of KR unless next residue is P
Number of mass values searched: **97**
Number of mass values matched: **17**
Sequence Coverage: **81%**

Matched peptides shown in **Bold Red**

```
  1 TEKDAQPQLE EADDDLDSKL NYKPPPQKSL KELQEMDKDD ESLTKYKKTL
 51 LGDVPVVADP TVPNVTVTRL SLVCDSAPGP ITMDLTGDLE ALKKDTFVLK
101 EGIEYRVKIN FKVNKDIVSG LKYVQHTYRT GMRVDKATFM VGSYGPRPEE
151 YEFLTPVEEA PKGMLARGTY HNKSFFTDDD KQDHLTWEWN LAIKKDWTE
```

Show predicted peptides also

Sort Peptides By ◉ Residue Number ○ Increasing Mass ○ Decreasing Mass

Start – End	Observed	Mr(expt)	Mr(calc)	ppm	Miss	Sequence	
4 – 19	1788.8712	1787.8639	1787.7697	53	0	K.DAQPQLEEADDDLDSK.L	
4 – 28	2854.5359	2853.5286	2853.3668	57	1	K.DAQPQLEEADDDLDSKLNYKPPPQK.S	
20 – 28	1084.6661	1083.6588	1083.6077	47	0	K.LNYKPPPQK.S	
32 – 45	1680.8522	1679.8449	1679.7560	53	1	K.ELQEMDKDDESLTK.Y	
32 – 45	1696.8540	1695.8467	1695.7509	57	1	K.ELQEMDKDDESLTK.Y	Oxidation (M)
48 – 69	2291.4194	2290.4121	2290.2897	53	1	K.KTLLGDVPVVADPTVPNVTVTR.L	
49 – 69	2163.3232	2162.3159	2162.1947	56	0	K.TLLGDVPVVADPTVPNVTVTR.L	
70 – 93	2516.4006	2515.3933	2515.2550	55	0	R.LSLVCDSAPGPITMDLTGDLEALK.K	Car
70 – 94	2644.5061	2643.4988	2643.3499	56	1	R.LSLVCDSAPGPITMDLTGDLEALKK.D	Ca
70 – 94	2660.5107	2659.5034	2659.3448	60	1	R.LSLVCDSAPGPITMDLTGDLEALKK.D	Ca
95 – 106	1469.8688	1468.8615	1468.7562	72	1	K.DTFVLKEGIEYR.V	
113 – 122	1072.6866	1071.6793	1071.6288	47	1	K.VNKDIVSGLK.Y	
123 – 129	966.5283	965.5210	965.4719	51	0	K.YVQHTYR.T	
137 – 162	2944.5640	2943.5567	2943.4000	53	0	K.ATFMVGSYGPRPEEYEFLTPVEEAPK.G	
137 – 162	2960.5784	2959.5711	2959.3950	60	0	K.ATFMVGSYGPRPEEYEFLTPVEEAPK.G	O
174 – 194	2609.3772	2608.3699	2608.2234	56	1	K.SFFTDDDKQDHLTWEWNLAIK.K	
182 – 194	1653.9197	1652.9124	1652.8311	49	0	K.QDHLTWEWNLAIK.K	

M22

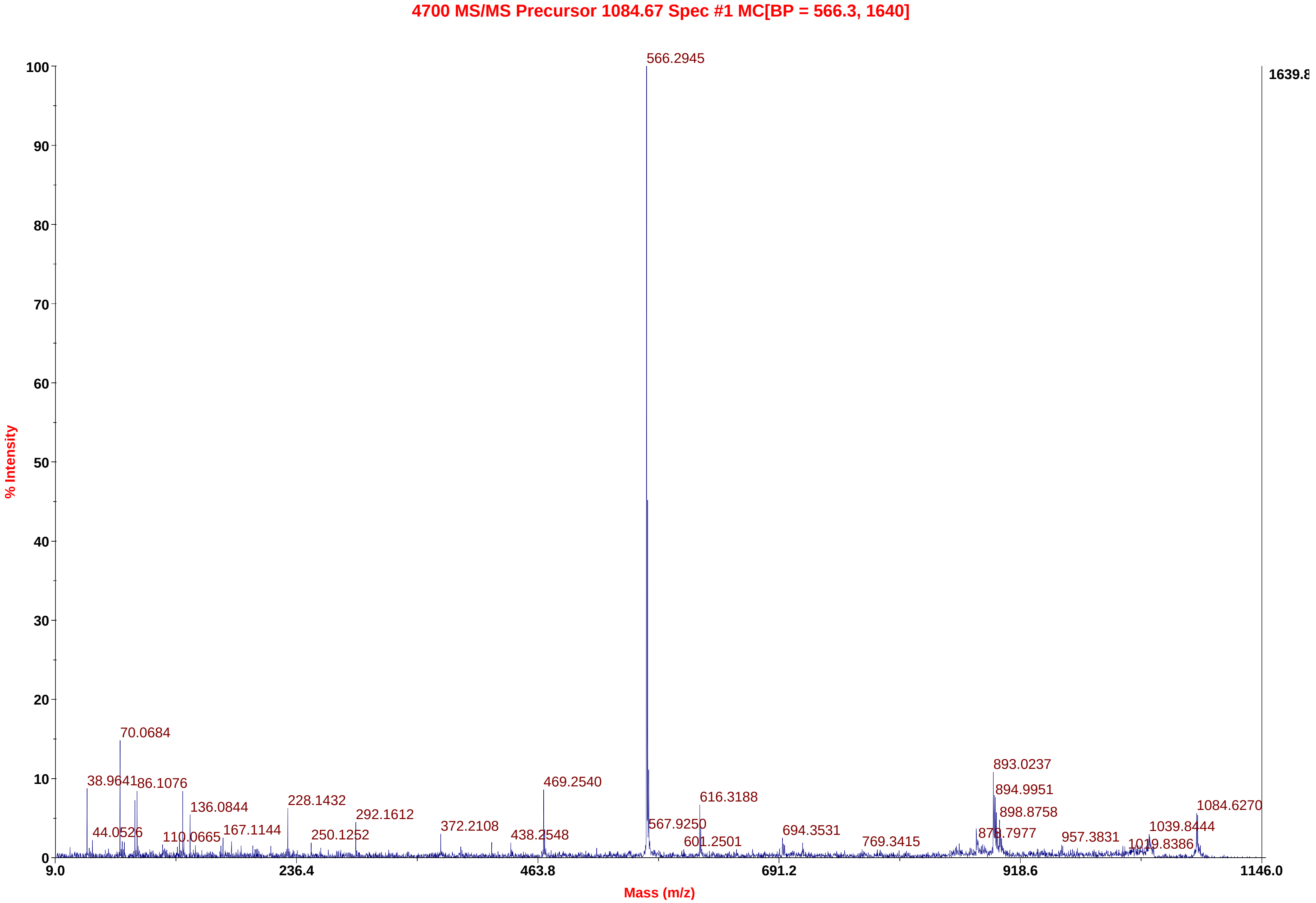
4700 MS/MS Precursor 1084.67 Spec #1 MC[BP = 566.3, 1640]
% Intensity
Mass (m/z)
1639.8
566.2945
70.0684
38.9641
86.1076
44.0526
110.0665
136.0844
167.1144
228.1432
250.1252
292.1612
372.2108
438.2548
469.2540
567.9250
601.2501
616.3188
694.3531
769.3415
878.7977
893.0237
894.9951
898.8758
957.3831
1019.8386
1039.8444
1084.6270
9.0
236.4
463.8
691.2
918.6
1146.0

M22

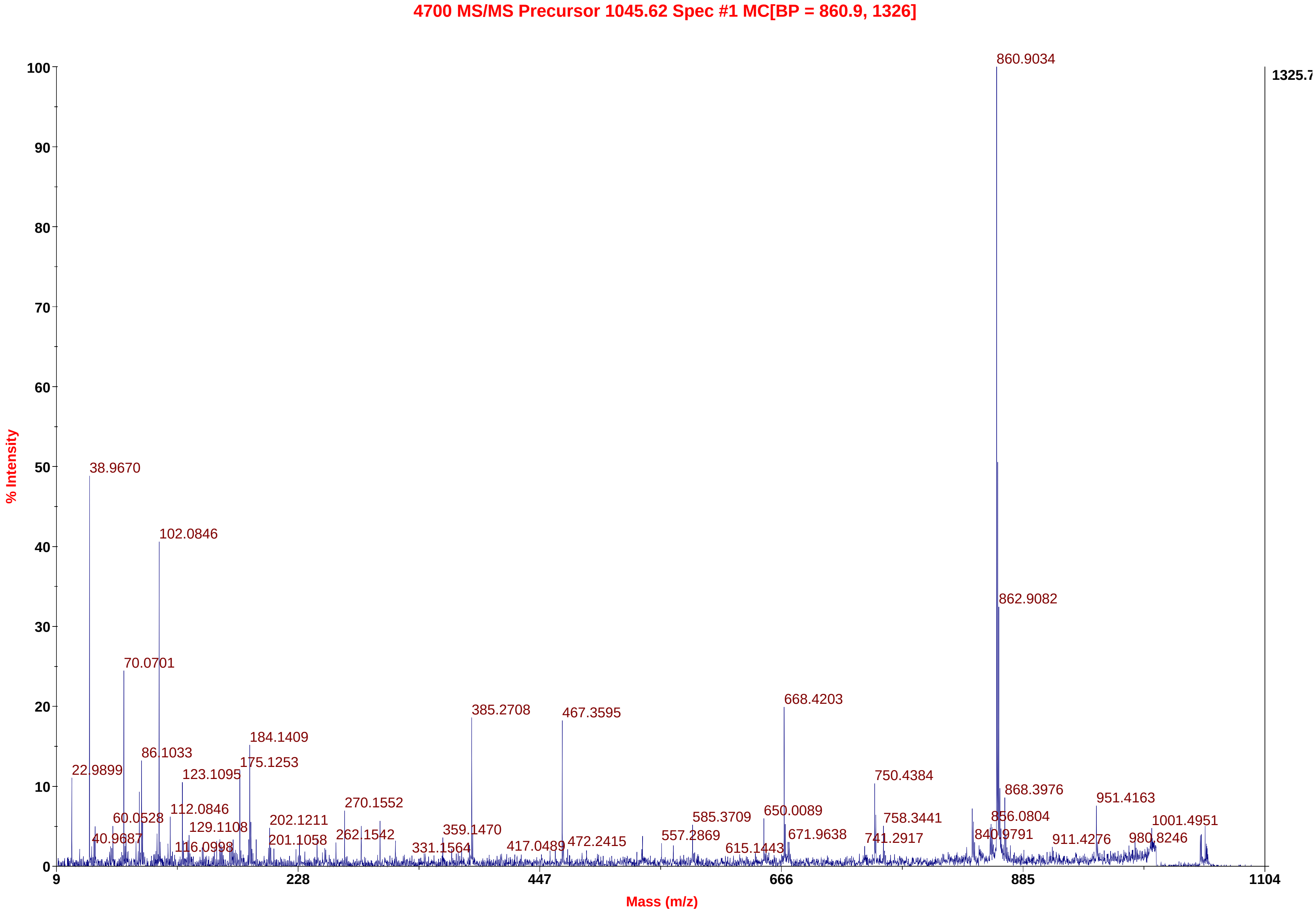
4700 MS/MS Precursor 1045.62 Spec #1 MC[BP = 860.9, 1326]
% Intensity
Mass (m/z)
1325.7
860.9034
38.9670
102.0846
862.9082
70.0701
668.4203
467.3595
385.2708
184.1409
86.1033
175.1253
22.9899
123.1095
750.4384
868.3976
951.4163
112.0846
270.1552
650.0089
60.0528
585.3709
758.3441
856.0804
1001.4951
202.1211
129.1108
359.1470
40.9687
201.1058
262.1542
557.2869
671.9638
840.9791
741.2917
911.4276
980.8246
116.0998
331.1564
417.0489
472.2415
615.1443
9
228
447
666
885
1104
0
10
20
30
40
50
60
70
80
90
100

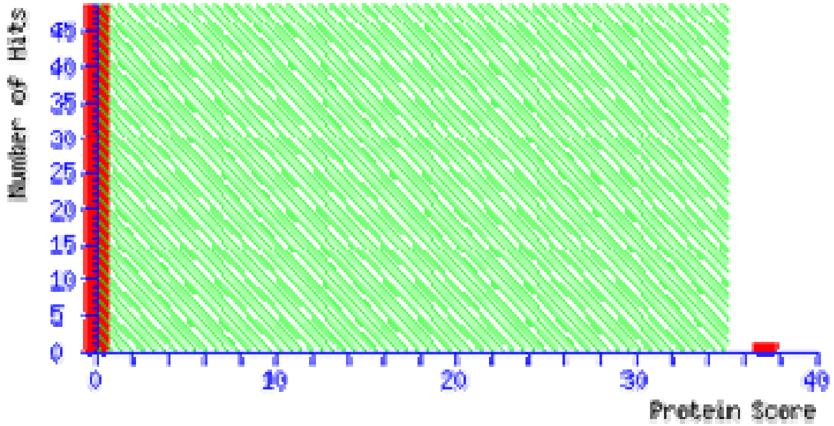

Peptide Summary Report

Format As | Peptide Summary | Help

Significance threshold p< 0.05 | Max. number of hits AUTO

Standard scoring | MudPIT scoring | Ions score or expect cut-off 0 | Show sub-sets 0

Show pop-ups | Suppress pop-ups | Sort unassigned Decreasing Score | Require bold red

Select All | Select None | Search Selected | **Error tolerant**

1. GDIS_MOUSE **Mass:** 22705 **Score:** 37 **Matches:** 5(0) **Sequences:** 5(0) **emPAI:** 0.65
Rho GDP-dissociation inhibitor 2 (Rho GDI 2) (Rho-GDI beta) (D4).- Mus musculus (Mouse).
Check to include this hit in error tolerant search

Query	Observed	Mr(expt)	Mr(calc)	ppm	Miss	Score	Expect	Rank	Unique	Peptide
1	966.5283	965.5210	965.4719	50.8	0	31	0.18	1	U	K.YVQHTYR.T
6	1084.6661	1083.6588	1083.6077	47.2	0	7	30	5	U	K.LNYKPPPQK.S
14	1788.8712	1787.8639	1787.7697	52.7	0	16	9.1	1	U	K.DAQPQLEEADDDLDSK.L
33	2163.3232	2162.3159	2162.1947	56.0	0	30	0.077	1	U	K.TLLGDVPVVADPTVPNVTVTR.L
55	2644.5061	2643.4988	2643.3499	56.3	1	9	24	1	U	R.LSLVCDSAPGPITMDLTGDLEALKK.D

Protein View

Match to: **GDIS_MOUSE** Score: 37
Rho GDP-dissociation inhibitor 2 (Rho GDI 2) (Rho-GDI beta) (D4).- Mus musculus (Mouse).
Found in search of ppw_F6_129990872005.txt

Nominal mass (M_r): **22705**; Calculated pI value: **4.97**
NCBI BLAST search of GDIS_MOUSE against nr
Unformatted sequence string for pasting into other applications

Taxonomy: Mus musculus

Variable modifications: Carbamidomethyl (C),Deamidated (NQ),Oxidation (M)
Cleavage by Trypsin: cuts C-term side of KR unless next residue is P
Sequence Coverage: 39%

Matched peptides shown in **Bold Red**

```
  1 TEKDAQPQLE EADDDLDSKL NYKPPPQKSL KELQEMDKDD ESLTKYKKTL
 51 LGDVPVVADP TVPNVTVTRL SLVCDSAPGP ITMDLTGDLE ALKKDTFVLK
101 EGIEYRVKIN FKVNKDIVSG LKYVQHTYRT GMRVDKATFM VGSYGPRPEE
151 YEFLTPVEEA PKGMLARGTY HNKSFFTDDD KQDHLTWEWN LAIKKDWTE
```

Show predicted peptides also

Sort Peptides By ◉ Residue Number ○ Increasing Mass ○ Decreasing Mass

Start - End	Observed	Mr(expt)	Mr(calc)	ppm	Miss	Sequence	
4 - 19	1788.8712	1787.8639	1787.7697	53	0	K.DAQPQLEEADDDLDSK.L	(Ions score 16)
20 - 28	1084.6661	1083.6588	1083.6077	47	0	K.LNYKPPPQK.S	(Ions score 7)
49 - 69	2163.3232	2162.3159	2162.1947	56	0	K.TLLGDVPVVADPTVPNVTVTR.L	(Ions score 30)
70 - 94	2644.5061	2643.4988	2643.3499	56	1	R.LSLVCDSAPGPITMDLTGDLEALKK.D	Carbamidomethyl (C)
123 - 129	966.5283	965.5210	965.4719	51	0	K.YVQHTYR.T	(Ions score 31)

Sample M23

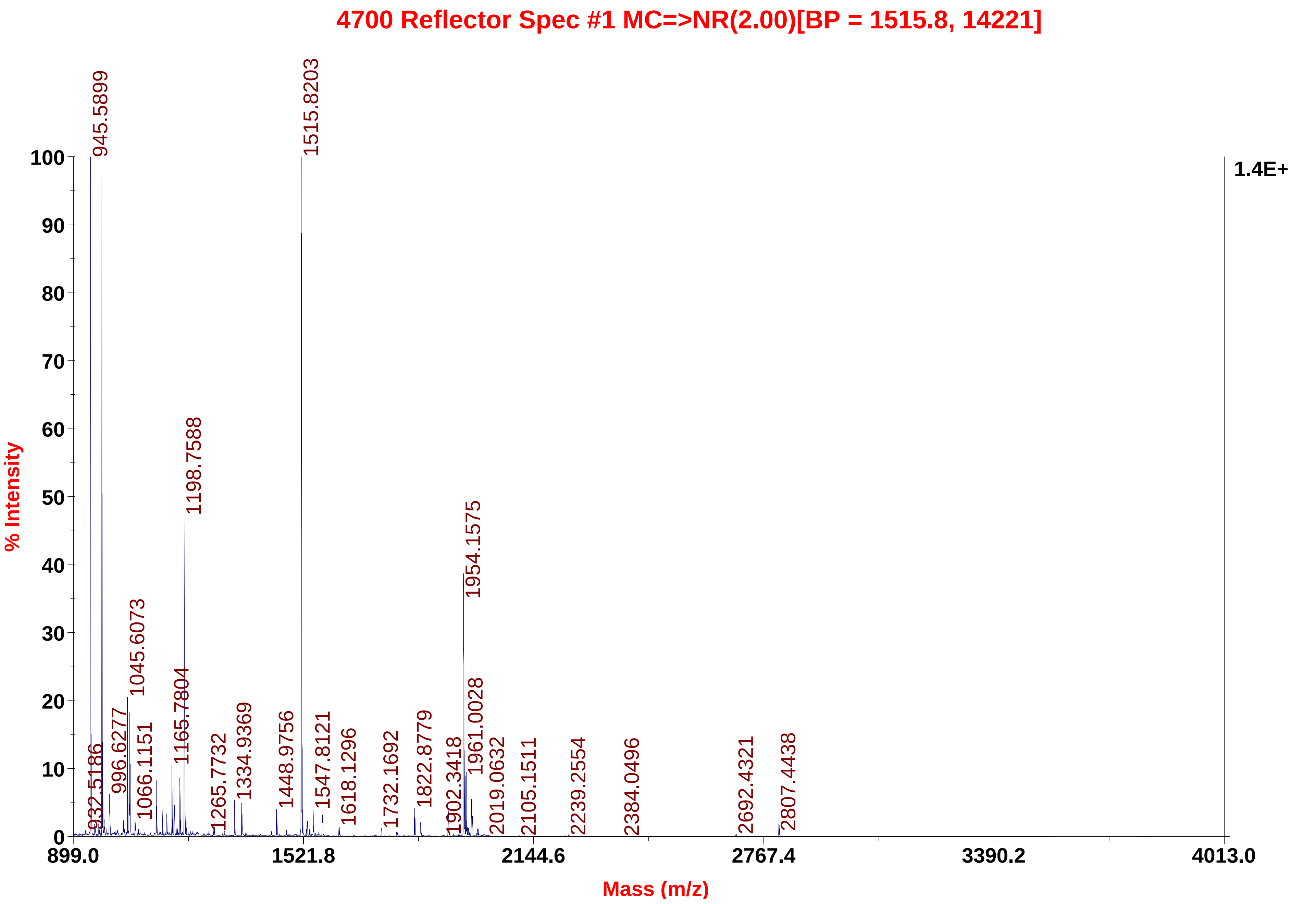

4700 Reflector Spec #1 MC=>NR(2.00)[BP = 1515.8, 14221]
945.5899
1515.8203
1198.7588
1954.1575
1045.6073
1165.7804
1961.0028
932.5186
996.6277
1066.1151
1265.7732
1334.9369
1448.9756
1547.8121
1618.1296
1732.1692
1822.8779
1902.3418
2019.0632
2105.1511
2239.2554
2384.0496
2692.4321
2807.4438
% Intensity
1.4E+
Mass (m/z)
899.0
1521.8
2144.6
2767.4
3390.2
4013.0

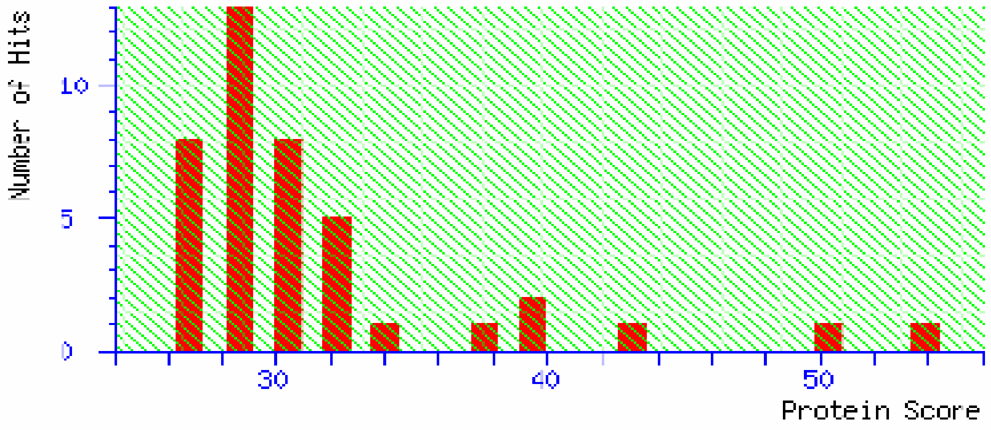

Concise Protein Summary Report

Format As | Concise Protein Summary | Help

Significance threshold p< 0.05

Max. number of hits AUTO

Re-Search All | Search Unmatched

1. AAF99333 **Mass:** 115840 **Score:** 54 **Expect:** 0.38 **Matches:** 29
AF289664 NID: - Mus musculus
AAH39162 **Mass:** 111802 **Score:** 49 **Expect:** 1.2 **Matches:** 26
BC039162 NID: - Mus musculus
AAH53048 **Mass:** 111787 **Score:** 48 **Expect:** 1.3 **Matches:** 26

Protein View

Match to: **AAF99333** Score: **54** Expect: **0.38**
AF289664 NID: - Mus musculus
Found in search of E:\Dekstop 30.5.2011\Muscle\Dekstop 2.5.11\Muscle DU\Muscle data \sample M23\pmf_G6_12990866106.txt

Nominal mass (M_r): **115840**; Calculated pI value: **6.07**
NCBI BLAST search of AAF99333 against nr
Unformatted sequence string for pasting into other applications

Taxonomy: Mus musculus
Links to retrieve other entries containing this sequence from NCBI Entrez:
(no taxonomy information for this entry)
(no taxonomy information for this entry)

Variable modifications: Carbamidomethyl (C),Deamidated (NQ),Oxidation (M)
Cleavage by Trypsin: cuts C-term side of KR unless next residue is P
Number of mass values searched: **83**
Number of mass values matched: **29**
Sequence Coverage: **24%**

Matched peptides shown in **Bold Red**

```
   1 MQKPSGLKPP GRGGKHSSPV GRPSVGSASS SVVASTSGSK EGSPLHKQAS
  51 GPSSSGAATT VSEKPGPKAA EVGDDFLGDF VVGERVWVNG VKPGVVQYLG
 101 ETQFAPGQWA GVVLDDPVGK NDGAVGGVRY FECPALQGIF TRPSKLTRQP
 151 TAEGSGSDTH SVESLTAQNL SLHSGTATPP LTGRVIPLRE SVLNSSVKTG
 201 NESGSNLSDS GSVKRGDKDL HLGDRVLVGG TKTGVVRYVG ETDFAKGEWC
 251 GVELDEPLGK NDGAVAGTRY FQCPPKFGLF APIHKVIRIG FPSTSPAKAK
 301 KTKRMAMGVS ALTHSPSSSS ISSVSSVASS VGGRPSRSGL LTETSSRYAR
 351 KISGTTALQE ALKEKQQHIE QLLAERDLER AEVAKATSHI CEVEKEIALL
 401 KAQHEQYVAE AEEKLQRARL LVENVRKEKV DLSNQLEEER RKVEDLQFRV
 451 EEESITKGDL ETQTQLEHAR IGELEQSLLL EKAQAERLLR ELADNRLTTV
 501 AEKSRVLQLE EELSLRRGEI EELQHCLLQS GPPPADHPEA AETLRLRERL
 551 LSASKEHQRD STLLQDKYEH MLKTYQTEVD KLRAANEKYA QEVADLKAKV
 601 QQATTENMGL MDNWKSKLDS LASDHQKSLE DLKATLNSGP GAQQKEIGEL
 651 KALVEGIKME HQLELGNLQA KHDLETAMHG KEKEGLRQKL QEVQEELAGL
 701 QQHWREQLEE QASQHRLELQ EAQDQCRDAQ LRAQELEGLD VEYRGQAQAI
 751 EFLKEQISLA EKKMLDYEML QRAEAQSRQE AERLREKLLV AENRLQAAES
 801 LCSAQHSHVI ESSDLSEETI RMKETVEGLQ DKLNKRDKEV TALTSQMDML
 851 RAQVSALENK CKSGEKKIDS LLKEKRRLEA ELEAVSRKTH DASGQLVHIS
 901 QELLRKERSL NELRVLLLEA NRHSPGPERD LSREVHKAEW RIKEQKLKDD
 951 IRGLREKLTG LDKEKSLSEQ RRYSLIDPAS PPELLKLQHQ LVSTEDALRD
1001 ALNQAQQVER LVEALRGCSD RTQTISNSGS ANGIHQPDKA HKQEDKH
```

M23

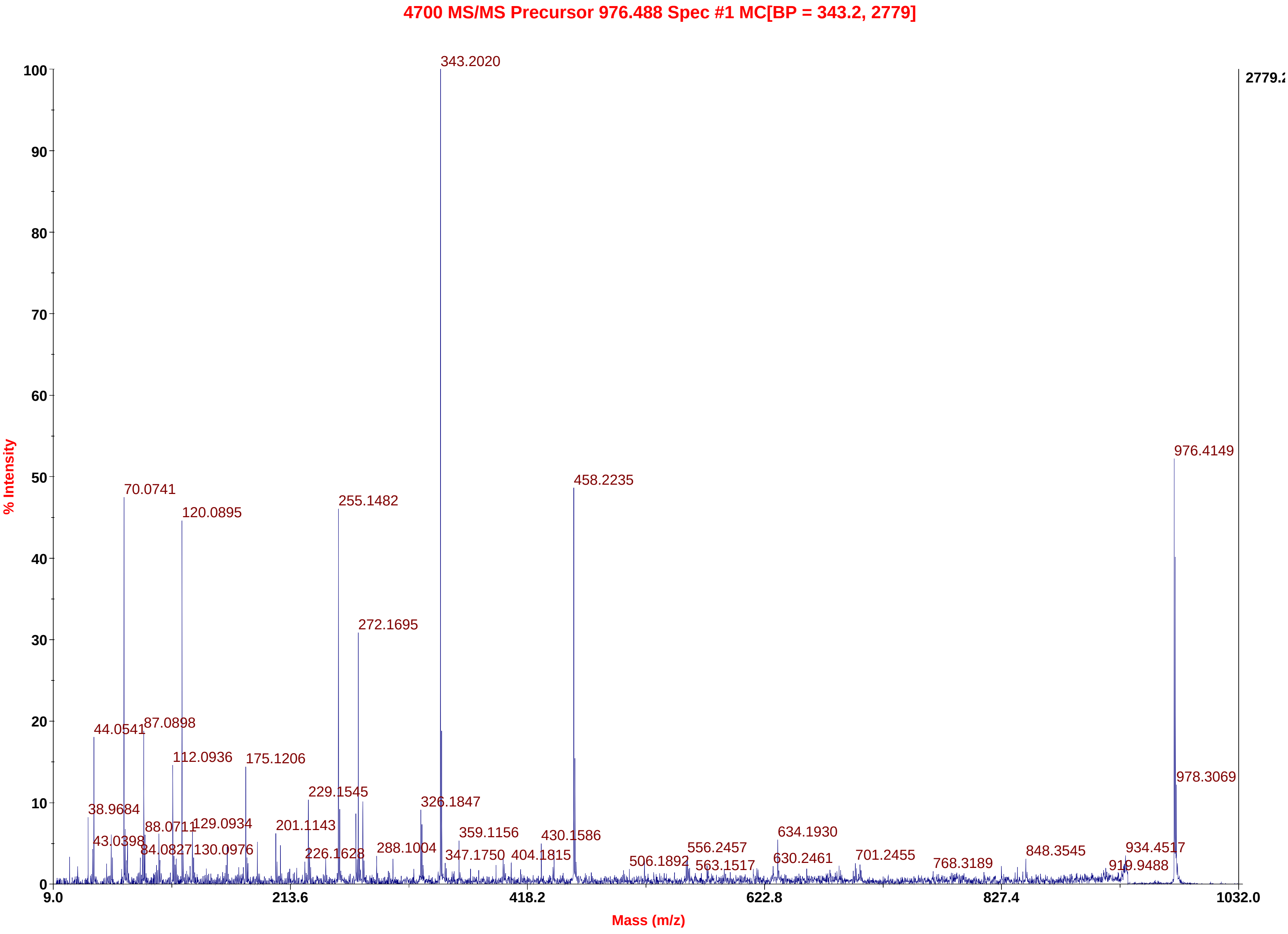
4700 MS/MS Precursor 976.488 Spec #1 MC[BP = 343.2, 2779]
% Intensity
Mass (m/z)
2779.2
343.2020
976.4149
458.2235
70.0741
120.0895
255.1482
272.1695
44.0541
87.0898
112.0936
175.1206
978.3069
229.1545
38.9684
326.1847
88.0711
129.0934
201.1143
359.1156
430.1586
634.1930
43.0398
556.2457
934.4517
84.0827
130.0976
288.1004
848.3545
226.1628
347.1750
404.1815
701.2455
630.2461
506.1892
768.3189
563.1517
919.9488
9.0
213.6
418.2
622.8
827.4
1032.0
0
10
20
30
40
50
60
70
80
90
100

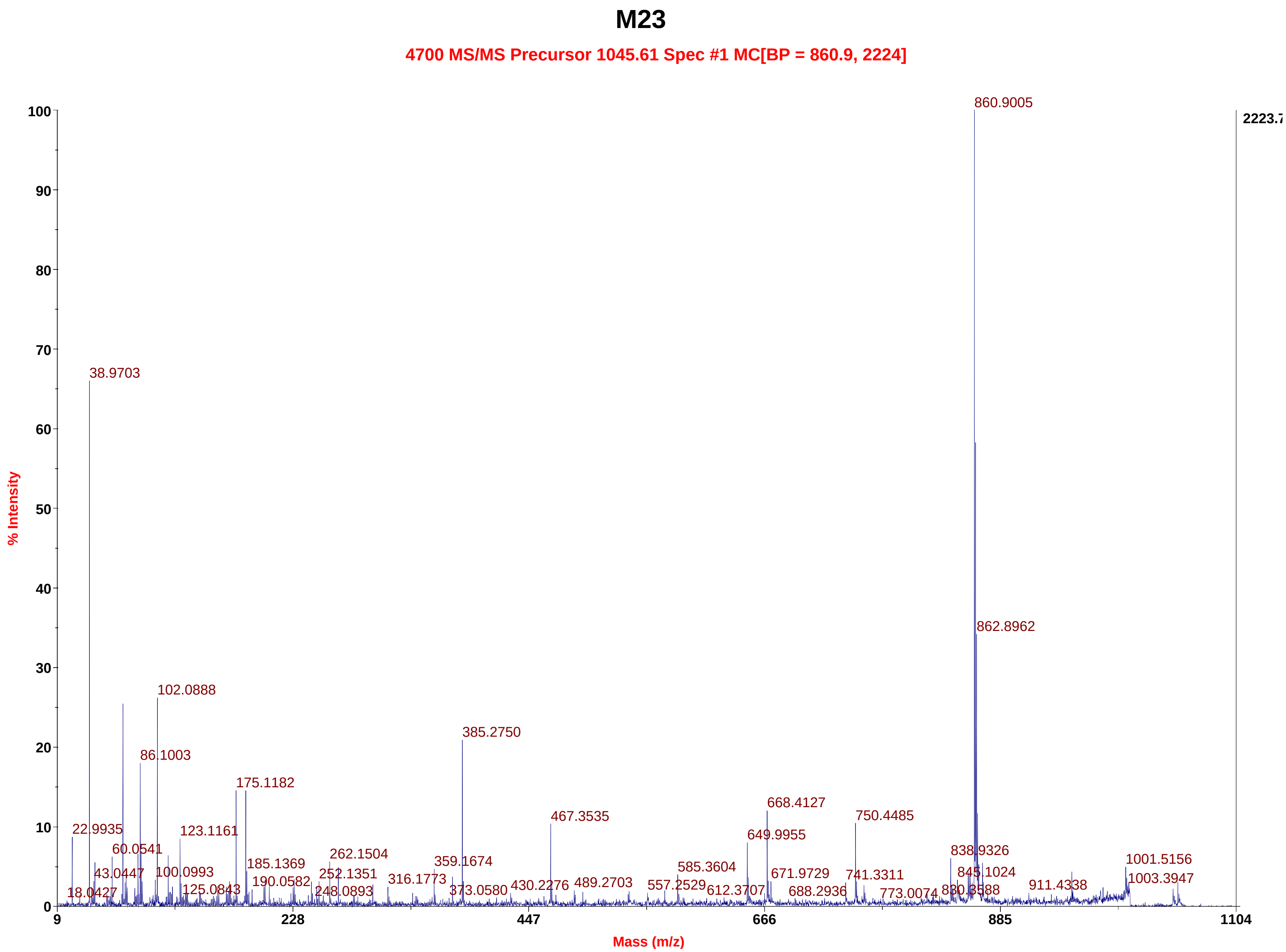
M23
4700 MS/MS Precursor 1045.61 Spec #1 MC[BP = 860.9, 2224]
% Intensity
Mass (m/z)
860.9005
862.8962
38.9703
102.0888
86.1003
385.2750
175.1182
22.9935
123.1161
467.3535
668.4127
750.4485
649.9955
60.0541
838.9326
1001.5156
185.1369
262.1504
359.1674
585.3604
43.0447
100.0993
252.1351
671.9729
741.3311
845.1024
1003.3947
190.0582
316.1773
430.2276
489.2703
557.2529
911.4338
125.0843
248.0893
373.0580
612.3707
688.2936
830.3588
18.0427
773.0074
2223.7

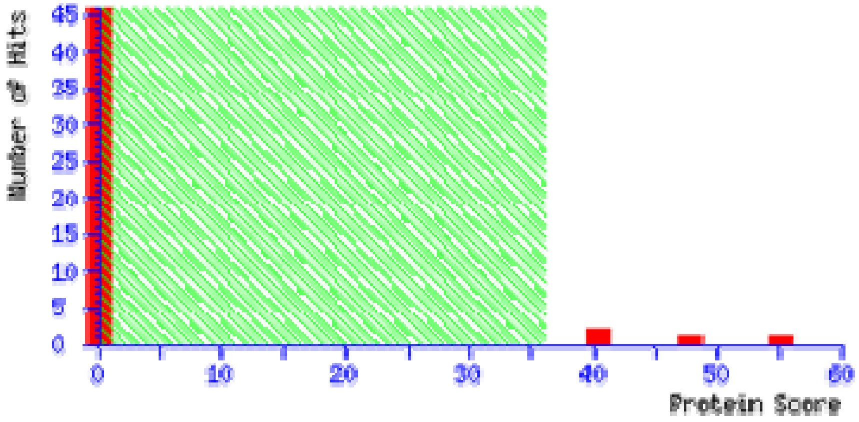

Peptide Summary Report

Format As | Peptide Summary | Help

Significance threshold p< 0.05 | Max. number of hits AUTO

Standard scoring ○ MudPIT scoring ● | Ions score or expect cut-off 0 | Show sub-sets 0

Show pop-ups ● Suppress pop-ups ○ | Sort unassigned Decreasing Score | Require bold red ☐

Select All | Select None | Search Selected | ☐ **Error tolerant**

1. CAA27396 **Mass:** 39161 **Score:** 55 **Matches:** 5(3) **Sequences:** 5(3) **emPAI:** 0.59
   MMACTBR2 NID: - Mus musculus
   ☐ Check to include this hit in error tolerant search

	Query	Observed	Mr(expt)	Mr(calc)	ppm	Miss	Score	Expect	Rank	Unique	Peptide
☑	1	945.5899	944.5826	944.5444	40.5	0	42	0.014	1	U	R.AVFPSIVGR.S
☑	8	1171.6173	1170.6100	1170.5638	39.5	0	42	0.017	1		R.HQGVMVGMGQK.D
☑	12	1354.6886	1353.6813	1353.6161	48.2	1	27	0.6	1		K.DSYVGDEAQSKR.G
☑	14	1515.8197	1514.8124	1514.7419	46.6	0	18	4.4	1		K.IWHHTFYNELR.V
☑	26	1954.1547	1953.1474	1953.0571	46.2	0	37	0.032	1		R.VAPEEHPVLLTEAPLNPK.A

Protein View

Match to: **CAA27396** Score: **55**
MMACTBR2 NID: - Mus musculus
Found in search of ppw_G6_129990872106.txt

Nominal mass (M_r): **39161**; Calculated pI value: **5.78**
NCBI BLAST search of CAA27396 against nr
Unformatted sequence string for pasting into other applications

Taxonomy: Mus musculus
Links to retrieve other entries containing this sequence from NCBI Entrez:
(no taxonomy information for this entry)

Variable modifications: Carbamidomethyl (C),Deamidated (NQ),Oxidation (M)
Cleavage by Trypsin: cuts C-term side of KR unless next residue is P
Sequence Coverage: **17%**

Matched peptides shown in **Bold Red**

```
  1 PRAVFPSIVG RSRHQGVMVG MGQKDSYVGD EAQSKRGILT LKYPIEHGIV
 51 TNWDDMEKIW HHTFYNELRV APEEHPVLLT EAPLNPKANR EKMTQIMFET
101 FNTPAMYVAI QAVLSLYASG RTTGIVMDSG DGVTHTVPIY EGYALPHAIL
151 RLDLAGRDLT DYLMKILTER GYSFTTTAER EIVRDIKEKL CYVALDFEQE
201 MATAASSSSL EKSYELPDGQ VITIGNERFR CPEALFQPSF LGMESCGIHE
251 TTFNSIMKCD VDIRKDLYAN TVLSGGTTMY PGIADRMQKE ITALAPSTMK
301 IKIIAPPERK YSVWIGGSIL ASLSTFQQMW ISKQEYDESG PSIVHRKCF
```

Show predicted peptides also

Sort Peptides By ◉ Residue Number ○ Increasing Mass ○ Decreasing Mass

Start - End	Observed	Mr(expt)	Mr(calc)	ppm	Miss	Sequence
3 - 11	945.5899	944.5826	944.5444	41	0	R.AVFPSIVGR.S (Ions score 42)
14 - 24	1171.6173	1170.6100	1170.5638	39	0	R.HQGVMVGMGQK.D (Ions score 42)
25 - 36	1354.6886	1353.6813	1353.6161	48	1	K.DSYVGDEAQSKR.G (Ions score 27)
59 - 69	1515.8197	1514.8124	1514.7419	47	0	K.IWHHTFYNELR.V (Ions score 18)
70 - 87	1954.1547	1953.1474	1953.0571	46	0	R.VAPEEHPVLLTEAPLNPK.A (Ions score 37)

Sample M24

4700 Reflector Spec #1 MC=>NR(2.00)[BP = 1475.8, 16515]

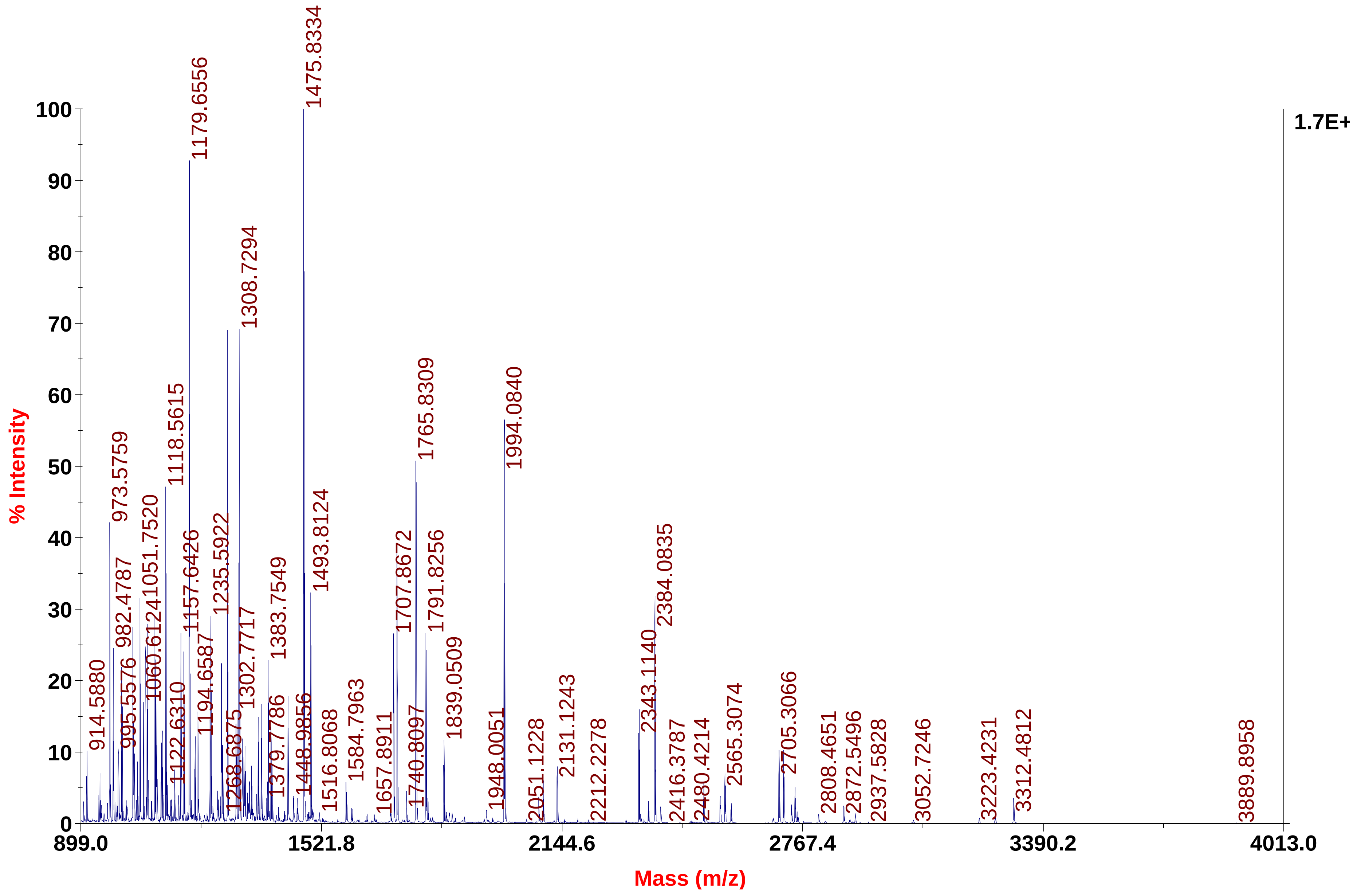

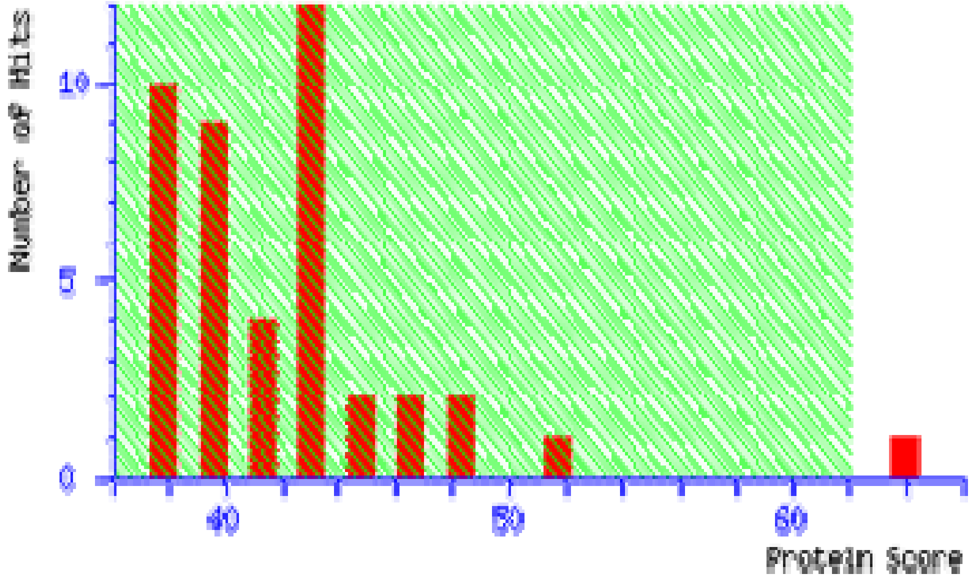

Concise Protein Summary Report

Format As | Concise Protein Summary | Help

Significance threshold p< 0.05 Max. number of hits AUTO

Re-Search All | Search Unmatched

1. Q6ZQK2_MOUSE **Mass:** 191193 **Score:** 64 **Expect:** 0.038 **Matches:** 65
MKIAA0051 protein (Fragment).- Mus musculus (Mouse).
Q8CGH5_MOUSE **Mass:** 106421 **Score:** 51 **Expect:** 0.72 **Matches:** 45
Iqgap1 protein (Fragment).- Mus musculus (Mouse).

Protein View

Match to: **Q6ZQK2_MOUSE** Score: **64** Expect: **0.038**
MKIAA0051 protein (Fragment).- Mus musculus (Mouse).
Found in search of pmf_H6_12990866107.txt

Nominal mass (M_r): **191193**; Calculated pI value: **6.15**
NCBI BLAST search of Q6ZQK2_MOUSE against nr
Unformatted sequence string for pasting into other applications

Taxonomy: Mus musculus
Links to retrieve other entries containing this sequence from NCBI Entrez:
(no taxonomy information for this entry)

Variable modifications: Carbamidomethyl (C),Deamidated (NQ),Oxidation (M)
Cleavage by Trypsin: cuts C-term side of KR unless next residue is P
Number of mass values searched: **180**
Number of mass values matched: **65**
Sequence Coverage: **37%**

Matched peptides shown in **Bold Red**

```
   1 LLLLRSPASS RSLRFLTGDL DSAAMSAAEE VDGLGVVRPH YGSVLDNERL
  51 TAEEMDERRR QNVAYEYLCH LEEAKRWMEA CLGEDLPPTT ELEEGLRNGV
 101 YLAKLGNFFS PKVVSLKKIY DREQTRYKAT GLHFRHTDNV IQWLNAMDEI
 151 GLPKIFYPET TDIYDRKNMP RCIYCIHALS LYLFKLGLAP QIQDLYGKVD
 201 FTEEEINNMK IELEKYGIQM PAFSKIGGIL ANELSVDEAA LHAAVIAINE
 251 AIDRRVAADT FTALKNPNAM LVNLEEGLAP TYQDVLYQAK QDKMTNAKNR
 301 TENSDRERDV YEELLTQAEI QGNVNKVNTS SALANISLAL EQGCAVTLLK
 351 ALQSLALGLR GLQTQNSDWY MKQLQSDLQQ KRQSGQTDPL QKEEVQAGVD
 401 AANSAAQQYQ RRLAAVAAIN AAIQKGIAEK TVLELMNPEA QLPQVYPFAA
 451 DLYQKELATL QQQSPEHSLT HPELTVAVEM LSSVALINRA LESGDMTTVW
 501 KQLSSSVTGL TNIEEENCQR YLDELMKLKA QAHAENNAFI TWNDIQACVD
 551 HVNLVVHEEH ERILAIGLIN EALDEGDAQK TLQALQIPAA KLEGVLAEVA
 601 QHYQDTLIRA KREKAQETQD ESAVLWLDEI QGGIWQSNKD TQEAQRFALG
 651 ISAINEAVDS GDVGRTLSAL RSPDVGLYGV IPECGETYRS DLAEAKKKRL
 701 AAGDNNSKWV KHWVKGGYHY YHNLETQAGG WAEPPDFVQN SVQLSREEIQ
 751 SSISGVTAAY NREQLWLANE GLITKLQACC RGYLVRQEFR SRMNFLKKQI
 801 PAITCIQSQW RGYKQKKAYQ DRLAYLHSHK DEVVKIQSLA RMHQARKRYR
 851 DRLQYFRDHI NDIIKIQAFI RANKARDDYK TLINAEDPPM IVVRKFVHLL
 901 DQSDQDFQEE LDLMKMREEV ITLIRSNQQL ENDLNLMDIK IGLLVKNKIT
 951 LQDVVSHSKK LTKKNKEQLS DMMMINKQKG GLKALSKEKR EKLEAYQHLF
1001 YLLQTNPTYL AKLIFQMPQN KSTKFMDSVI FTLYNYASNQ REEYLLLRLF
1051 QTALQEEIKS KVDQIQEIVT GNPTVIKMVV SFNRGARGQN ALRQILAPVV
1101 KEIMDDKSLN IKTDPVDIYK SWVNQMESQT GEASKLPYDV TPEQALSHEE
1151 VKTRLDNSIR NMRAVTDKFL SAIVSSVDKI PYGMRFIAKV LKDSLHEKFP
1201 DAGEDELLKI IGNLLYYRYM NPAIVAPDAF DIIDLSAGGQ LTTDQRRNLG
1251 SIAKMLQHAA SNKMFLGDNA HLSIINEYLS QSYQKFRRFF QVACDVPELQ
1301 DKFNVDEYSD LVTLTKPVIY ISIGEIINTH TLLLDHQDAI APEHNDPIHE
1351 LLDDLGEVPT IESLIGESCG NSNDPNKEAL AKTEVSLTLT NKFDVPGDEN
1401 AEMDARTILL NTKRLIVDVI RFQPGETLTE ILETPATNEQ EAEHQRAMQR
1451 RAIRDAKTPD KMKKSKPMKE DNNLSLQEKK EKIQTGLKKL TELGTVDPKN
1501 RYQELINDIA KDIRNQRRYR QRRKAELVKL QQTYSALNSK ATFYGEQVDY
1551 YKSYIKTCLD NLASKGKVSK KPREMKGKKS KKISLKYTAA RLHEKGVLLE
1601 IEDLQANQFK NVIFEIGPTE EVGDFEVKAK FMGVQMETFM LHYQDLLQLQ
1651 YEGVAVMKLF DRAKVNVNLL IFLLNKKFYG K
```

Show predicted peptides also

Sort Peptides By ◉ Residue Number ○ Increasing Mass ○ Decreasing Mass

Start – End	Observed	Mr(expt)	Mr(calc)	ppm	Miss	Sequence
50 – 58	1109.5443	1108.5370	1108.4706	60	0	R.LTAEEMDER.R Oxidation (M)
50 – 59	1265.7042	1264.6969	1264.5717	99	1	R.LTAEEMDERR.R Oxidation (M)
61 – 75	1868.0181	1867.0108	1866.8458	88	0	R.QNVAYEYLCHLEEAK.R Carbamido
119 – 126	1081.5437	1080.5364	1080.5200	15	1	K.IYDREQTR.Y Deamidated (NQ)
127 – 135	1092.5634	1091.5561	1091.5876	-29	1	R.YKATGLHFR.H
168 – 185	2315.2419	2314.2346	2314.1424	40	1	K.NMPRCIYCIHALSLYLFK.L 2 Carb

M24

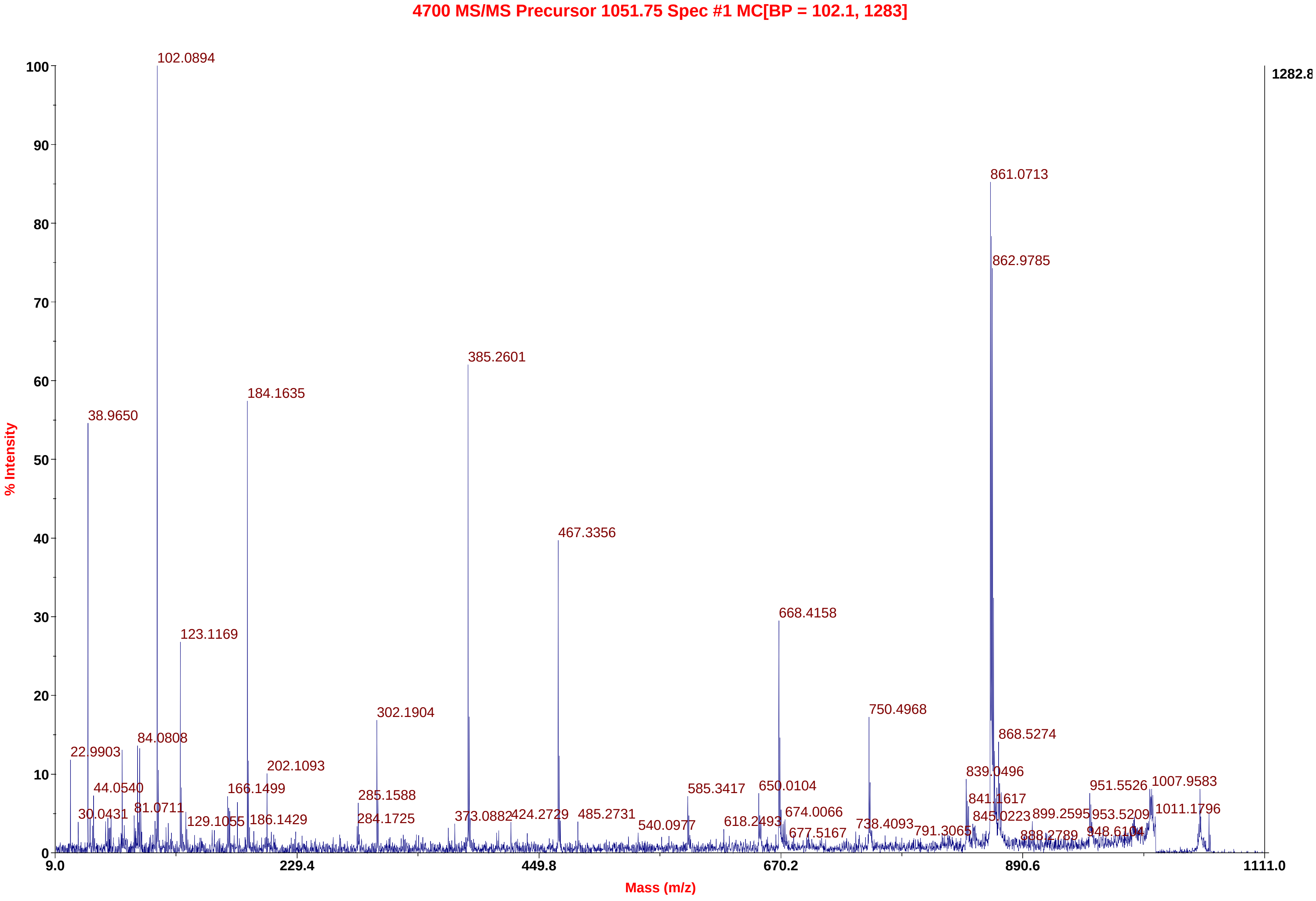

4700 MS/MS Precursor 1051.75 Spec #1 MC[BP = 102.1, 1283]
% Intensity
Mass (m/z)
1282.8
102.0894
861.0713
862.9785
385.2601
184.1635
38.9650
467.3356
123.1169
668.4158
302.1904
750.4968
868.5274
84.0808
22.9903
202.1093
839.0496
44.0540
166.1499
285.1588
585.3417
650.0104
951.5526
1007.9583
841.1617
30.0431
81.0711
129.1055
186.1429
284.1725
373.0882
424.2729
485.2731
540.0977
618.2493
674.0066
677.5167
738.4093
791.3065
845.0223
899.2595
953.5209
888.2789
948.6104
1011.1796

M24

4700 MS/MS Precursor 1090.58 Spec #1 MC[BP = 175.1, 1795]

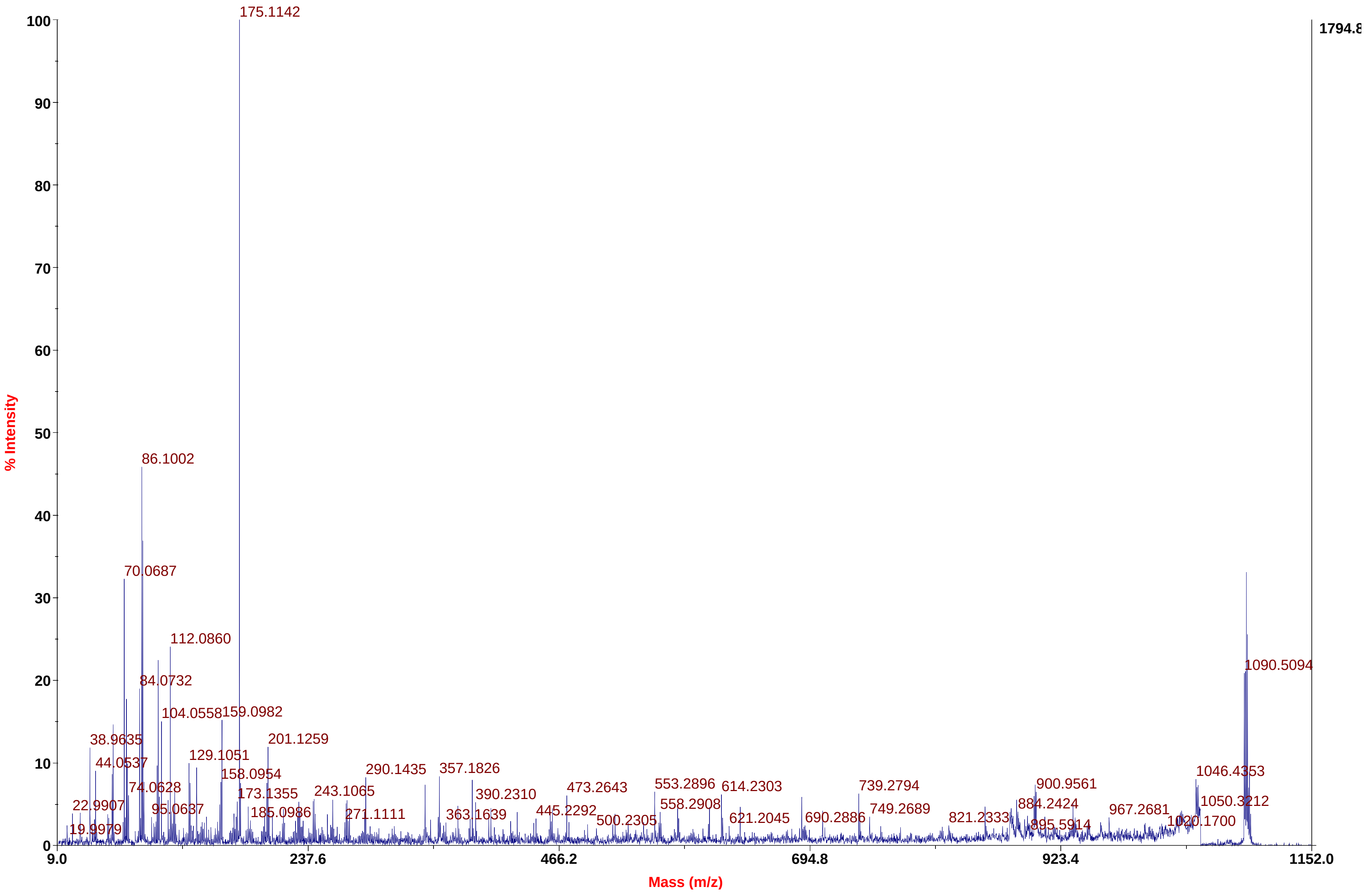

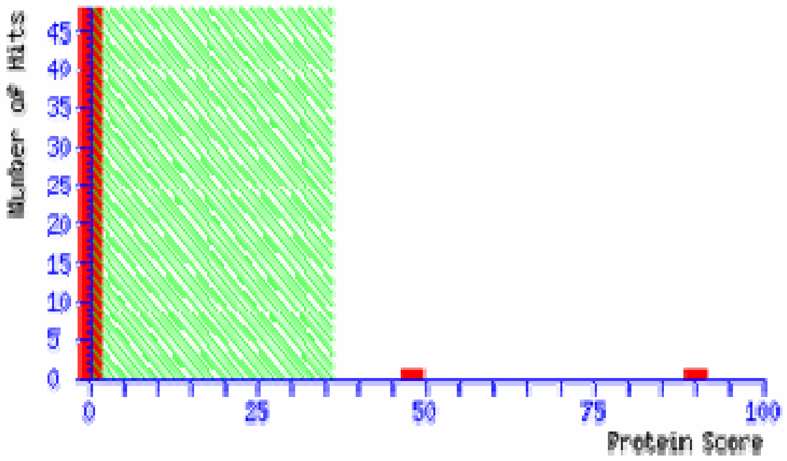

Peptide Summary Report

Format As | Peptide Summary | Help

Significance threshold p< 0.05 | Max. number of hits AUTO

Standard scoring ○ MudPIT scoring ● | Ions score or expect cut-off 0 | Show sub-sets 0

Show pop-ups ● Suppress pop-ups ○ | Sort unassigned Decreasing Score | Require bold red ☐

Select All | Select None | Search Selected | ☐ **Error tolerant**

1. Q3TK56_MOUSE **Mass:** 10117 **Score:** 90 **Matches:** 2(1) **Sequences:** 2(1) **emPAI:** 0.70

Blastocyst blastocyst cDNA, RIKEN full-length enriched library, clone:I1C0034H10 product:actin related protein 2/

☐ Check to include this hit in error tolerant search

	Query	Observed	Mr(expt)	Mr(calc)	ppm	Miss	Score	Expect	Rank	Unique	Peptide
☑	4	1070.6798	1069.6725	1069.6244	45.0	0	16	2.5	1	U	K.ALAAGGVGSIVR.V
☑	44	2131.1230	2130.1157	2130.0018	53.5	0	90	3e-07	1	U	K.GFESPSDNSSAVLLQWHEK.A

Protein View

Match to: **Q3TK56_MOUSE** Score: **90**
Blastocyst blastocyst cDNA, RIKEN full-length enriched library, clone:I1C0034H10 product:actin related protein 2/3
Found in search of ppw_H6_129990872207.txt

Nominal mass (M_r): **10117**; Calculated pI value: **9.79**
NCBI BLAST search of Q3TK56_MOUSE against nr
Unformatted sequence string for pasting into other applications

Taxonomy: Mus musculus
Links to retrieve other entries containing this sequence from NCBI Entrez:
(no taxonomy information for this entry)

Variable modifications: Carbamidomethyl (C),Deamidated (NQ),Oxidation (M)
Cleavage by Trypsin: cuts C-term side of KR unless next residue is P
Sequence Coverage: 32%

Matched peptides shown in **Bold Red**

```
 1 ALKNPPINTK SQAVKDRAGS IVLKVLISFK ANDIEKAVQS LDKNGVDLLM
51 KYIYKGFESP SDNSSAVLLQ WHEKALAAGG VGSIVRVLTA RKTV
```

Show predicted peptides also

Sort Peptides By ◉ Residue Number ○ Increasing Mass ○ Decreasing Mass

Start - End	Observed	Mr(expt)	Mr(calc)	ppm	Miss	Sequence	
56 - 74	2131.1230	2130.1157	2130.0018	53	0	K.GFESPSDNSSAVLLQWHEK.A	(Ions score 90)
75 - 86	1070.6798	1069.6725	1069.6244	45	0	K.ALAAGGVGSIVR.V	(Ions score 16)

Sample M25

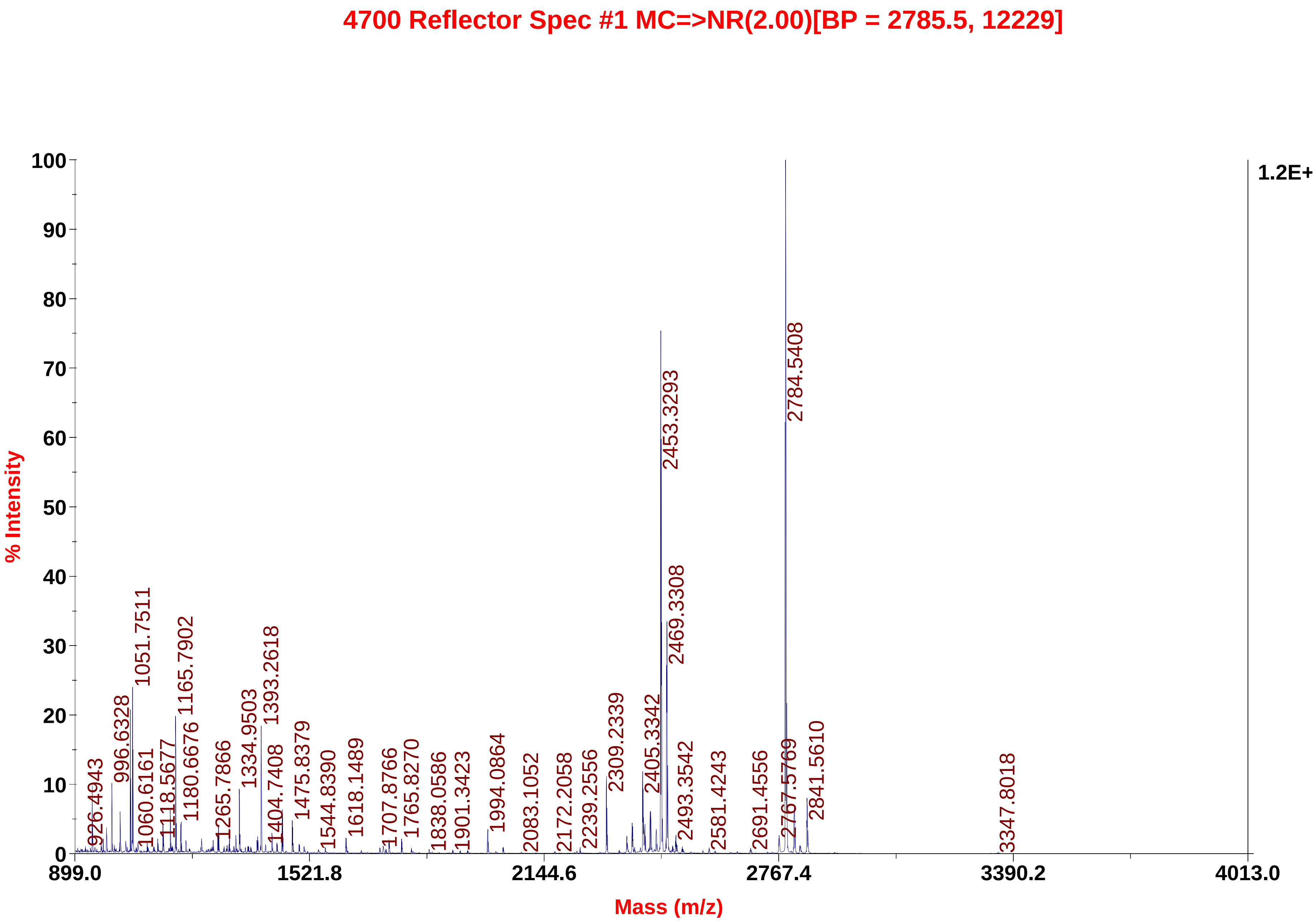
4700 Reflector Spec #1 MC=>NR(2.00)[BP = 2785.5, 12229]
% Intensity
Mass (m/z)
1.2E+
926.4943
996.6328
1051.7511
1060.6161
1118.5677
1165.7902
1180.6676
1265.7866
1334.9503
1393.2618
1404.7408
1475.8379
1544.8390
1618.1489
1707.8766
1765.8270
1838.0586
1901.3423
1994.0864
2083.1052
2172.2058
2239.2556
2309.2339
2405.3342
2453.3293
2469.3308
2493.3542
2581.4243
2691.4556
2767.5769
2784.5408
2841.5610
3347.8018

M25

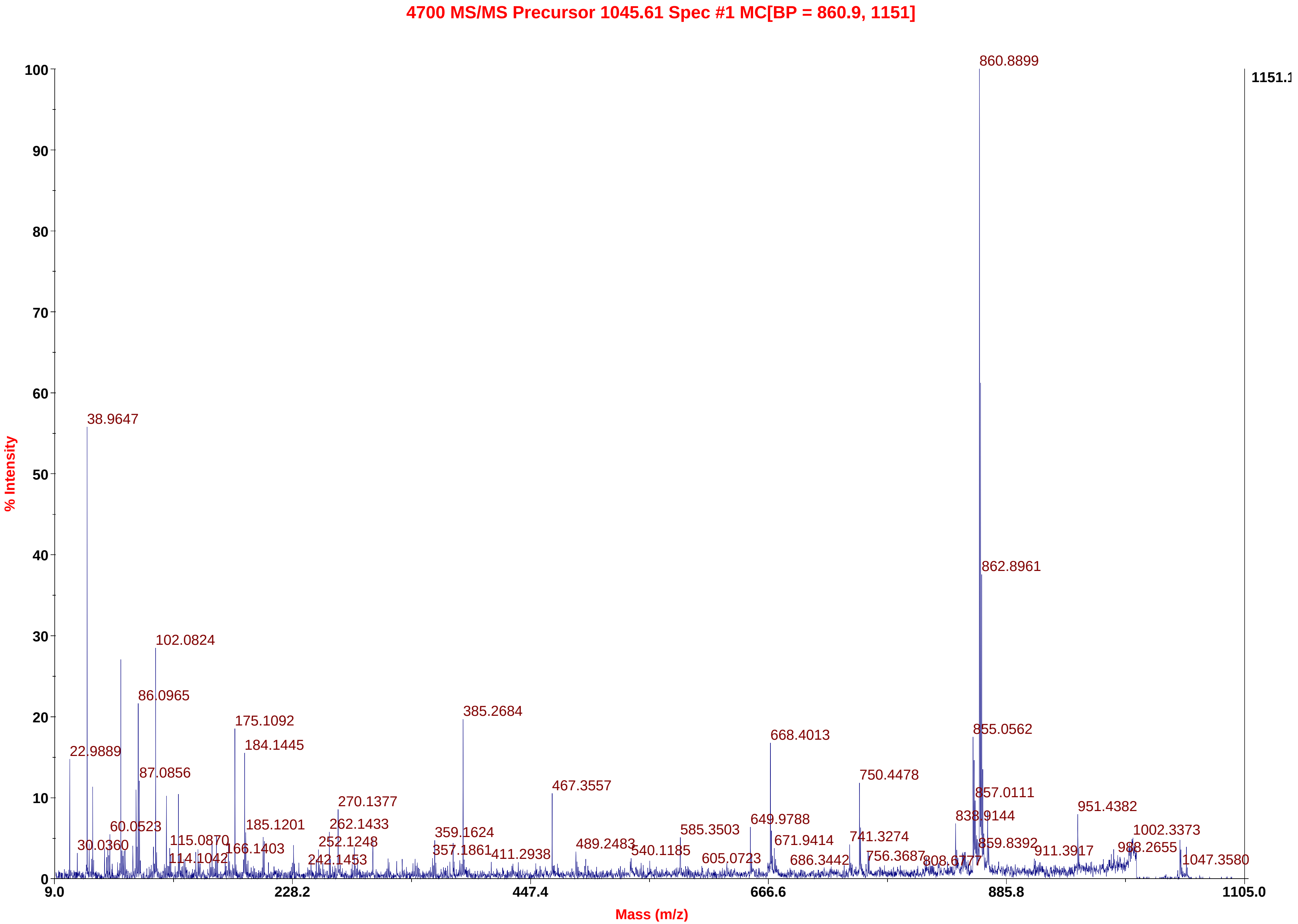

4700 MS/MS Precursor 1045.61 Spec #1 MC[BP = 860.9, 1151]
% Intensity
Mass (m/z)
860.8899
862.8961
855.0562
857.0111
838.9144
859.8392
38.9647
102.0824
86.0965
87.0856
22.9889
60.0523
30.0360
115.0870
114.1042
166.1403
175.1092
184.1445
185.1201
270.1377
262.1433
252.1248
242.1453
385.2684
359.1624
357.1861
411.2938
467.3557
489.2483
540.1185
585.3503
605.0723
668.4013
649.9788
671.9414
686.3442
750.4478
741.3274
756.3687
808.6777
951.4382
911.3917
1002.3373
988.2655
1047.3580

M25

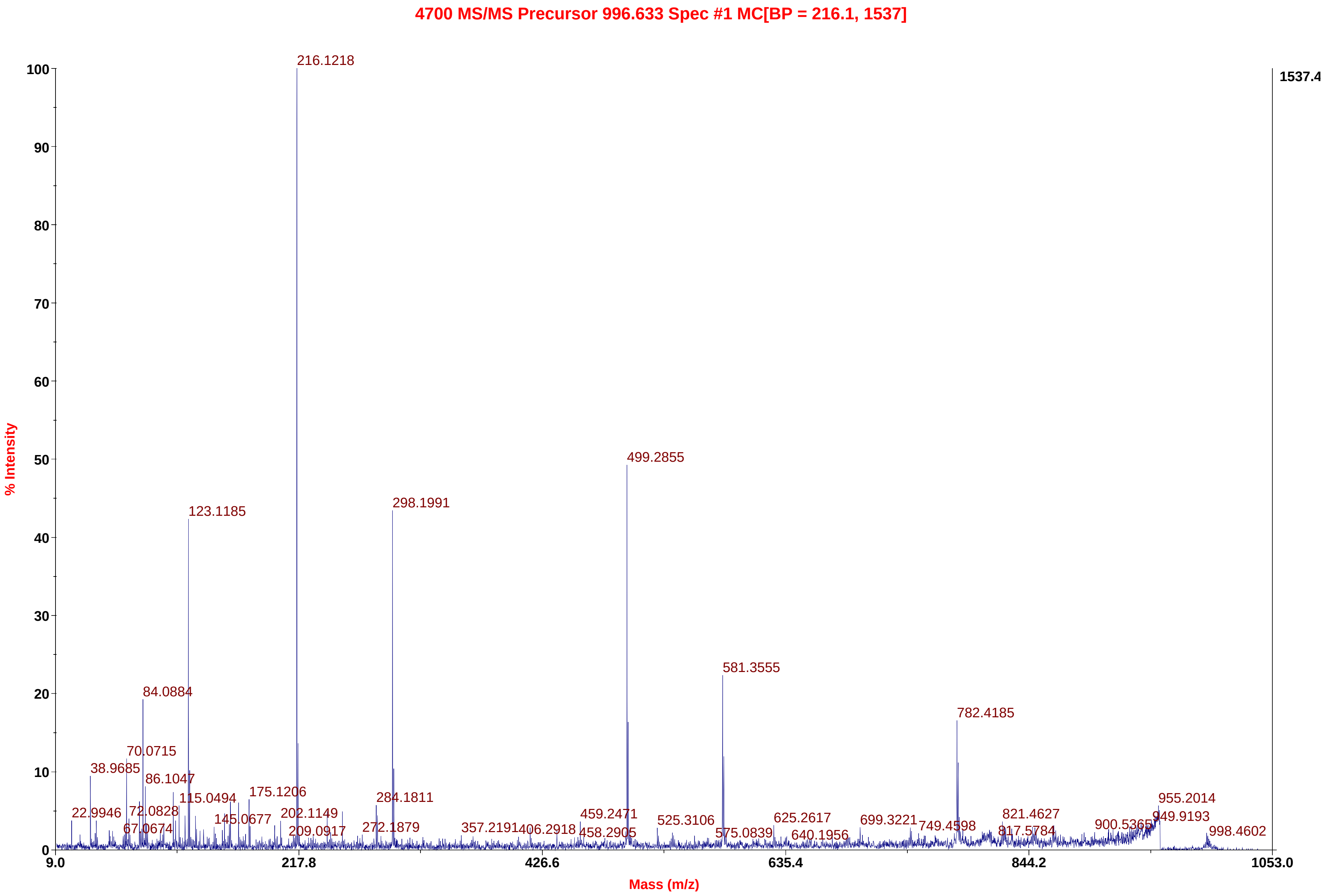

4700 MS/MS Precursor 996.633 Spec #1 MC[BP = 216.1, 1537]
% Intensity
Mass (m/z)
1537.4
216.1218
499.2855
298.1991
123.1185
581.3555
84.0884
782.4185
70.0715
38.9685
86.1047
175.1206
115.0494
284.1811
955.2014
22.9946
72.0828
202.1149
459.2471
821.4627
625.2617
699.3221
145.0677
525.3106
949.9193
67.0674
272.1879
357.2191
406.2918
749.4598
900.5365
209.0917
458.2905
575.0839
640.1956
817.5784
998.4602
9.0
217.8
426.6
635.4
844.2
1053.0

Sample M26

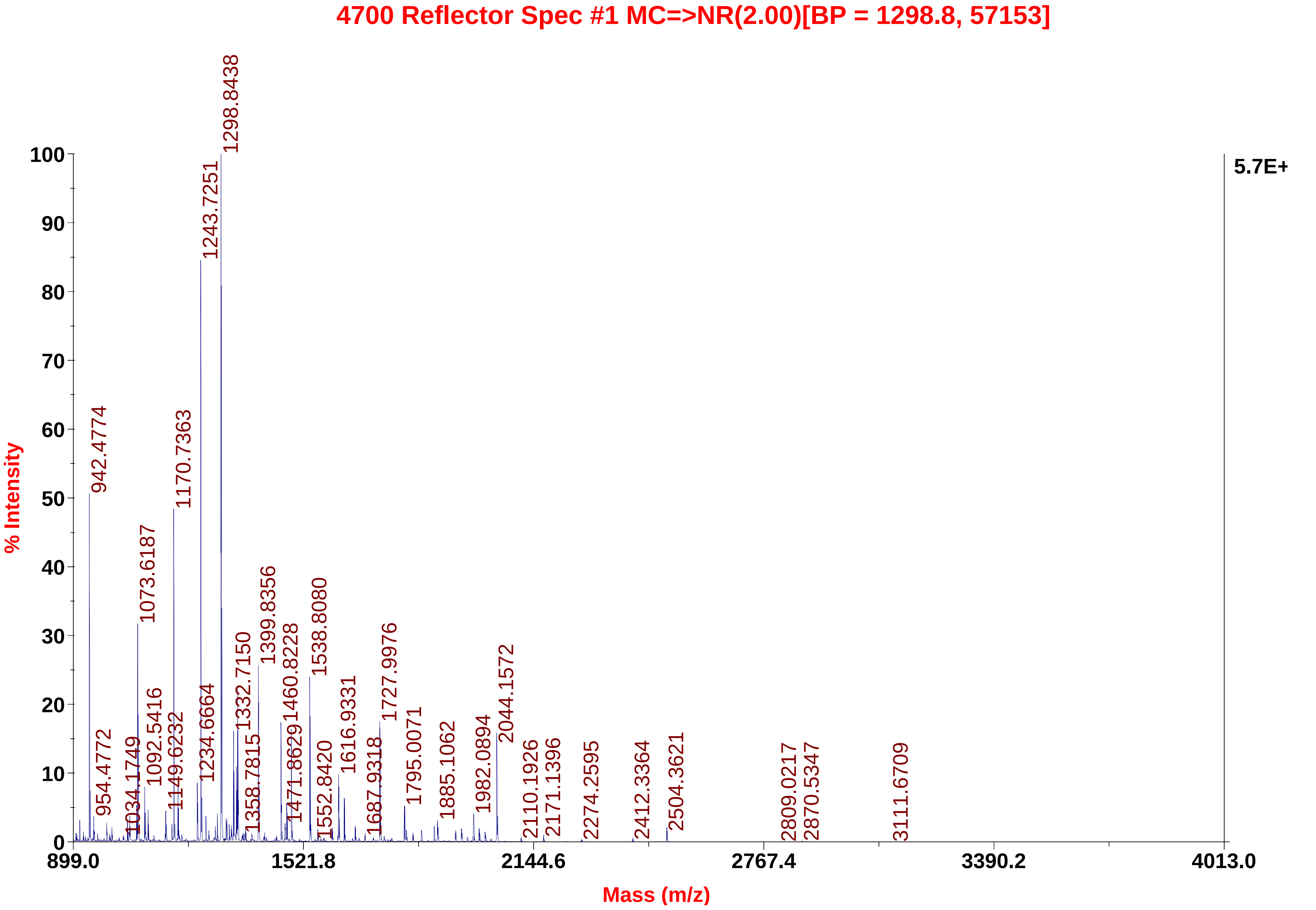

4700 Reflector Spec #1 MC=>NR(2.00)[BP = 1298.8, 57153]
% Intensity
Mass (m/z)
5.7E+
100
90
80
70
60
50
40
30
20
10
0
899.0
1521.8
2144.6
2767.4
3390.2
4013.0
942.4774
954.4772
1034.1749
1073.6187
1092.5416
1149.6232
1170.7363
1234.6664
1243.7251
1298.8438
1332.7150
1358.7815
1399.8356
1460.8228
1471.8629
1538.8080
1552.8420
1616.9331
1687.9318
1727.9976
1795.0071
1885.1062
1982.0894
2044.1572
2110.1926
2171.1396
2274.2595
2412.3364
2504.3621
2809.0217
2870.5347
3111.6709

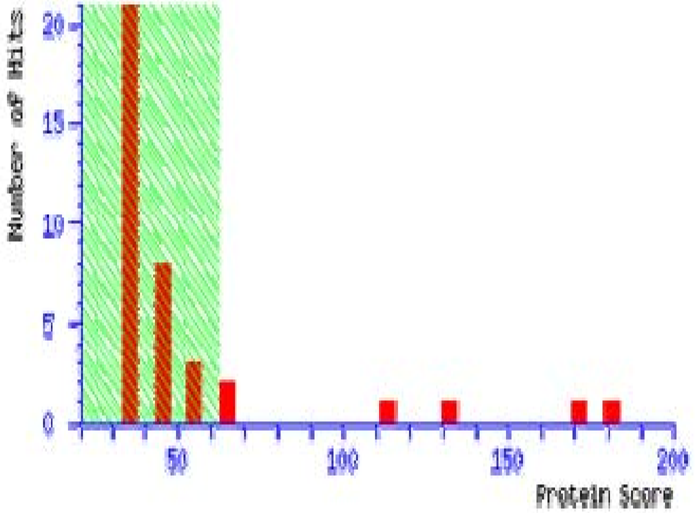

Concise Protein Summary Report

Format As | Concise Protein Summary | Help

Significance threshold p< 0.05 Max. number of hits AUTO

Re-Search All | Search Unmatched

1. **Mixture 1** **Total score:** 181 **Expect:** 7.3e-14 **Matches:** 45

   Components (only one family member shown for each component):

   Q9D1R6_MOUSE **Mass:** 32804 **Score:** 167 **Expect:** 1.8e-12 **Matches:** 35

   18-day embryo whole body cDNA, RIKEN full-length enriched library, clone:1100001K11 product:tropomyosin 2, beta, full insert sequ

   Q9CY74_MOUSE **Mass:** 17645 **Score:** 53 **Expect:** 0.43 **Matches:** 11

   8 days embryo whole body cDNA, RIKEN full-length enriched library, clone:5730592G18 product:RIKEN cDNA 5730592G18.- Mus musculus

Protein View

Match to: **Q9D1R6_MOUSE** Score: **167** Expect: **1.8e-12**
18-day embryo whole body cDNA, RIKEN full-length enriched library, clone:1100001K11 product:tropomyosin 2, beta, full
Found in search of pmf_J6_129990866209.txt

Nominal mass (M_r): **32804**; Calculated pI value: **4.64**
NCBI BLAST search of Q9D1R6_MOUSE against nr
Unformatted sequence string for pasting into other applications

Taxonomy: Mus musculus
Links to retrieve other entries containing this sequence from NCBI Entrez:
(no taxonomy information for this entry)

Variable modifications: Carbamidomethyl (C),Deamidated (NQ),Oxidation (M)
Cleavage by Trypsin: cuts C-term side of KR unless next residue is P
Number of mass values searched: **129**
Number of mass values matched: **35**
Sequence Coverage: **70%**

Matched peptides shown in **Bold Red**

```
  1 MDAIKKKMQM LKLDKENAID RADEAEADKK QAEDRCKQLE EEQQALQKKL
 51 KGTEDEVEKY SESVKDAQEK LEQAEKKATD AEADVASLNR RIQLVEEELD
101 RAQERLATAL QKLEEAEKAA DESERGMKVI ENRAMKDEEK MELQEMQLKE
151 AKHIAEDSDR KYEEVARKLV ILEGELERSE ERAEVAESKC GDLEEELKIV
201 TNNLKSLEAQ ADKYSTKEDK YEEEIKLLEE KLKEAETRAE FAERSVAKLE
251 KTIDDLEDEV YAQKMKYKAI SEELDNALND ITSL
```

Show predicted peptides also

Sort Peptides By ● Residue Number ○ Increasing Mass ○ Decreasing Mass

Start – End	Observed	Mr(expt)	Mr(calc)	ppm	Miss	Sequence
13 – 21	1073.6161	1072.6088	1072.5513	54	1	K.LDKENAIDR.A
31 – 37	906.4929	905.4856	905.4025	92	1	K.QAEDRCK.Q Carbamidomethyl (C)
36 – 48	1576.7727	1575.7654	1575.7450	13	1	R.CKQLEEEQQALQK.K 2 Deamidated (NQ)
36 – 48	1631.8989	1630.8916	1630.7984	57	1	R.CKQLEEEQQALQK.K Carbamidomethyl (C)
38 – 48	1343.7493	1342.7420	1342.6728	52	0	K.QLEEEQQALQK.K
38 – 49	1471.8601	1470.8528	1470.7678	58	1	K.QLEEEQQALQKK.L
38 – 49	1475.8446	1474.8373	1474.7038	91	1	K.QLEEEQQALQKK.L 4 Deamidated (NQ)
50 – 59	1147.6428	1146.6355	1146.5768	51	1	K.LKGTEDEVEK.Y
52 – 65	1599.8319	1598.8246	1598.7311	58	1	K.GTEDEVEKYSESVK.D
60 – 70	1283.6857	1282.6784	1282.6041	58	1	K.YSESVKDAQEK.L
66 – 76	1288.7073	1287.7000	1287.6306	54	1	K.DAQEKLEQAEK.K
77 – 90	1460.8223	1459.8150	1459.7267	61	1	K.KATDAEADVASLNR.R
78 – 90	1332.7152	1331.7079	1331.6317	57	0	K.ATDAEADVASLNR.R
78 – 91	1488.8296	1487.8223	1487.7328	60	1	K.ATDAEADVASLNRR.I
91 – 101	1399.8350	1398.8277	1398.7467	58	1	R.RIQLVEEELDR.A
92 – 101	1243.7239	1242.7166	1242.6456	57	0	R.IQLVEEELDR.A
92 – 105	1727.9966	1726.9893	1726.8849	60	1	R.IQLVEEELDRAQER.L
113 – 125	1476.7725	1475.7652	1475.6739	62	1	K.LEEAEKAADESER.G
141 – 149	1149.6221	1148.6148	1148.5569	50	0	K.MELQEMQLK.E
141 – 149	1165.6195	1164.6122	1164.5518	52	0	K.MELQEMQLK.E Oxidation (M)
141 – 152	1510.8165	1509.8092	1509.7054	69	1	K.MELQEMQLKEAK.H Deamidated (NQ); 2 Oxidation (M)
153 – 160	942.4771	941.4698	941.4203	53	0	K.HIAEDSDR.K
153 – 161	1070.5800	1069.5727	1069.5152	54	1	K.HIAEDSDRK.Y
168 – 178	1298.8428	1297.8355	1297.7605	58	1	R.KLVILEGELER.S
169 – 178	1170.7357	1169.7284	1169.6656	54	0	K.LVILEGELER.S
169 – 182	1671.9833	1670.9760	1670.8839	55	1	K.LVILEGELERSEER.A
179 – 189	1234.6641	1233.6568	1233.5837	59	1	R.SEERAEVAESK.C
183 – 198	1749.9827	1748.9754	1748.8138	92	1	R.AEVAESKCGDLEEELK.I
190 – 198	1092.5413	1091.5340	1091.4805	49	0	K.CGDLEEELK.I Carbamidomethyl (C)
190 – 205	1817.9924	1816.9851	1816.9240	34	1	K.CGDLEEELKIVTNNLK.S
190 – 205	1875.0626	1874.0553	1873.9455	59	1	K.CGDLEEELKIVTNNLK.S Carbamidomethyl (C)
206 – 217	1340.7412	1339.7339	1339.6619	54	1	K.SLEAQADKYSTK.E
218 – 226	1182.6132	1181.6059	1181.5452	51	1	K.EDKYEEEIK.L
234 – 244	1308.7366	1307.7293	1307.6106	91	1	K.EAETRAEFAER.S
252 – 264	1538.8053	1537.7980	1537.7148	54	0	K.TIDDLEDEVYAQK.M

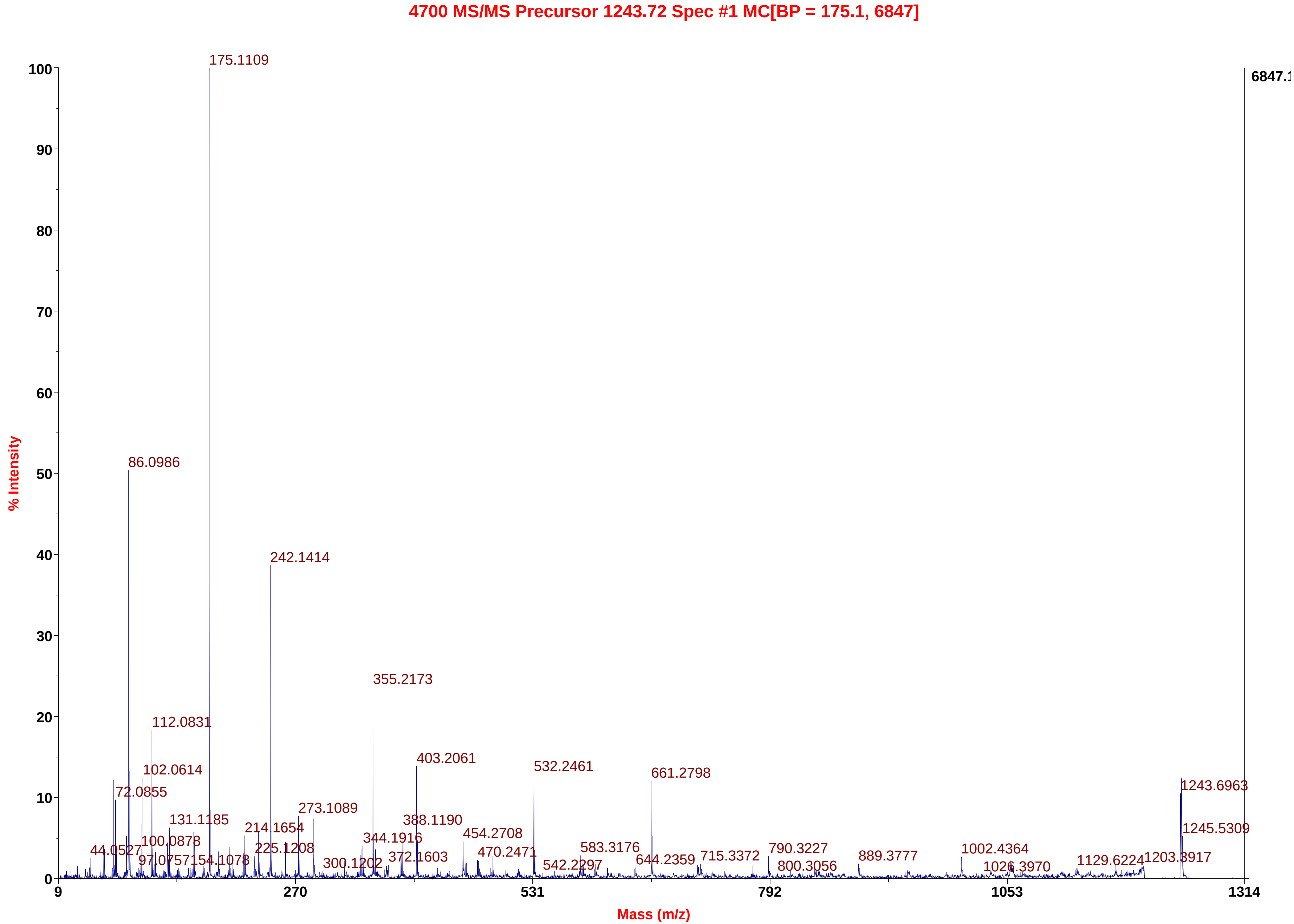
4700 MS/MS Precursor 1243.72 Spec #1 MC[BP = 175.1, 6847]
% Intensity
Mass (m/z)
6847.1
175.1109
86.0986
242.1414
355.2173
112.0831
403.2061
532.2461
661.2798
102.0614
72.0855
1243.6963
273.1089
131.1185
388.1190
214.1654
454.2708
1245.5309
344.1916
44.0527
100.0878
225.1208
583.3176
790.3227
1002.4364
470.2471
372.1603
715.3372
889.3777
644.2359
1203.3917
1129.6224
97.0757
154.1078
300.1202
542.2297
800.3056
1026.3970
0
10
20
30
40
50
60
70
80
90
100
9
270
531
792
1053
1314

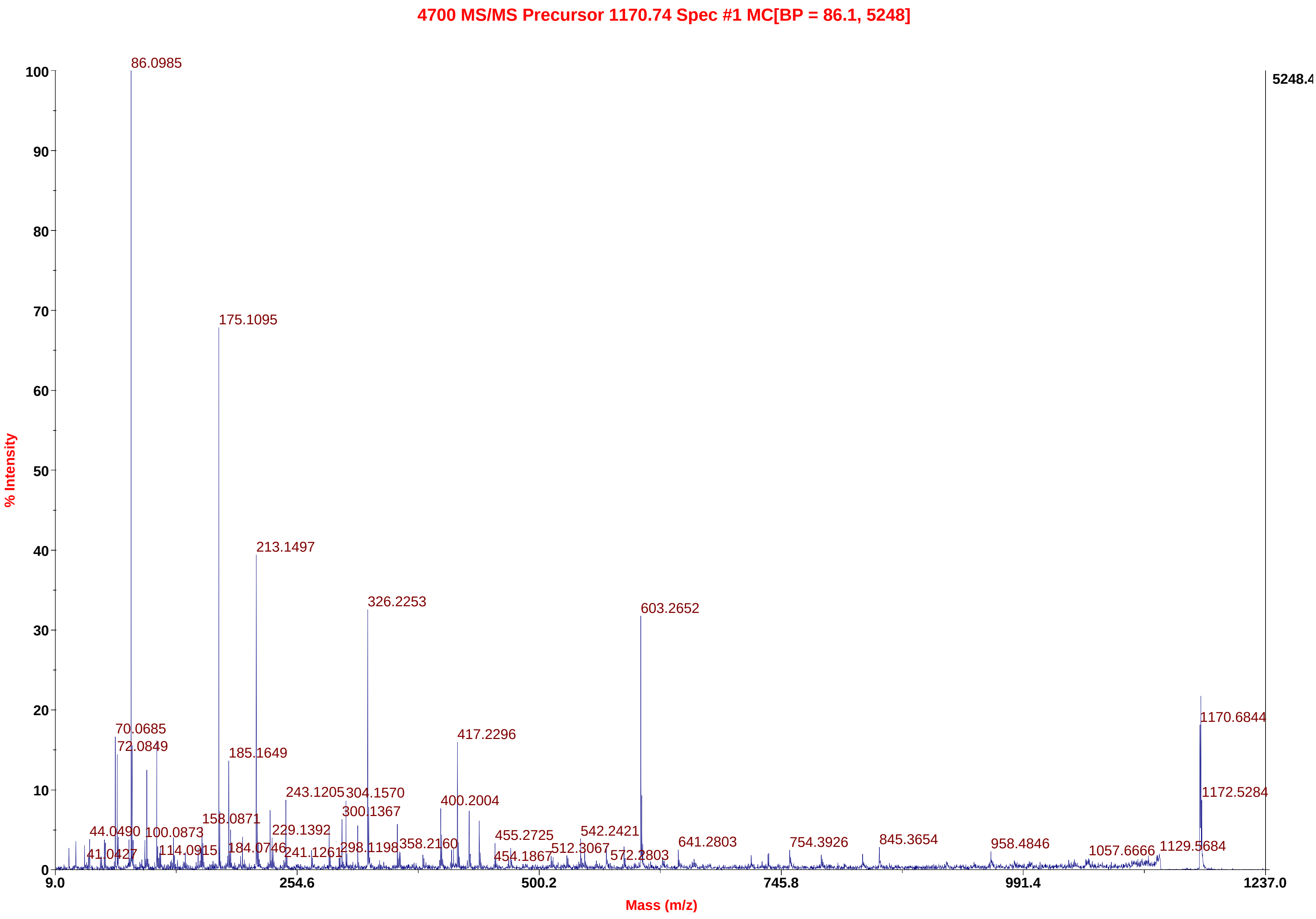

4700 MS/MS Precursor 1170.74 Spec #1 MC[BP = 86.1, 5248]
% Intensity
Mass (m/z)
5248.4
100
90
80
70
60
50
40
30
20
10
0
9.0
254.6
500.2
745.8
991.4
1237.0
86.0985
175.1095
213.1497
326.2253
603.2652
70.0685
72.0849
185.1649
417.2296
1170.6844
1172.5284
243.1205
304.1570
300.1367
400.2004
158.0871
44.0490
100.0873
229.1392
455.2725
542.2421
641.2803
754.3926
845.3654
958.4846
1057.6666
1129.5684
41.0427
114.0915
184.0746
241.1261
298.1198
358.2160
454.1867
512.3067
572.2803

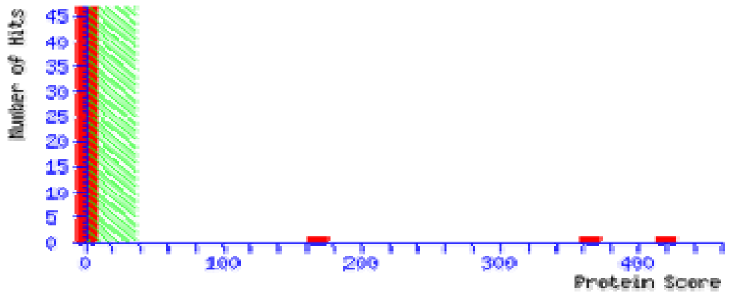

Peptide Summary Report

Format As | Peptide Summary | Help

Significance threshold p< 0.05 | Max. number of hits AUTO

Standard scoring ○ MudPIT scoring ◉ | Ions score or expect cut-off 0 | Show sub-sets 0

Show pop-ups ◉ Suppress pop-ups ○ | Sort unassigned Decreasing Score | Require bold red ☐

Select All | Select None | Search Selected | ☐ **Error tolerant**

1. TMRBB **Mass:** 32817 **Score:** 420 **Matches:** 15(12) **Sequences:** 15(12) **emPAI:** 8.90

tropomyosin beta chain, skeletal muscle [validated] - rabbit (tentative sequence)

☐ Check to include this hit in error tolerant search

	Query	Observed	Mr(expt)	Mr(calc)	ppm	Miss	Score	Expect	Rank	Unique	Peptide
☑	1	942.4771	941.4698	941.4203	52.7	0	46	0.0052	1		K.HIAEDSDR.K
☑	2	1073.6161	1072.6088	1072.5513	53.7	1	37	0.048	1		K.LDKENAIDR.A
☑	3	1092.5413	1091.5340	1091.4805	49.1	0	55	0.001	1	U	K.CGDLEEELK.I + Carbamidomethyl (C)
☑	5	1170.7357	1169.7284	1169.6656	53.7	0	76	2.3e-06	1		K.LVILEGELER.S
☑	6	1182.6132	1181.6059	1181.5452	51.4	1	35	0.091	1		K.EDKYEEEIK.L
☑	7	1243.7239	1242.7166	1242.6456	57.2	0	65	7e-05	1		R.IQLVEEELDR.A
☑	8	1298.8428	1297.8355	1297.7605	57.8	1	82	3.1e-07	1		R.KLVILEGELER.S
☑	9	1343.7493	1342.7420	1342.6728	51.5	0	57	0.0005	1		K.QLEEEQQALQK.K
☑	10	1399.8350	1398.8277	1398.7467	58.0	1	16	4	1		R.RIQLVEEELDR.A
☑	11	1460.8223	1459.8150	1459.7267	60.5	1	72	1.6e-05	1		K.KATDAEADVASLNR.R
☑	12	1476.7725	1475.7652	1475.6739	61.9	1	74	1.4e-05	1		K.LEEAEKAADESER.G
☑	13	1488.8296	1487.8223	1487.7328	60.2	1	33	0.14	1		K.ATDAEADVASLNRR.I
☑	14	1538.8053	1537.7980	1537.7148	54.1	0	85	1e-06	1	U	K.TIDDLEDEVYAQK.M
☑	16	1631.8989	1630.8916	1630.7984	57.1	1	85	8.1e-07	1		R.CKQLEEEQQALQK.K + Carbamidomethyl (C)
☑	17	1727.9966	1726.9893	1726.8849	60.5	1	51	0.0017	1		R.IQLVEEELDRAQER.L

Protein View

Match to: **TMRBB** Score: **420**
tropomyosin beta chain, skeletal muscle [validated] - rabbit (tentative sequence)
Found in search of ppw_J6_129990872509.txt

Nominal mass (M_r): **32817**; Calculated pI value: **4.66**
NCBI BLAST search of TMRBB against nr
Unformatted sequence string for pasting into other applications

Taxonomy: Oryctolagus cuniculus
Links to retrieve other entries containing this sequence from NCBI Entrez:
(no taxonomy information for this entry)
(no taxonomy information for this entry)
(no taxonomy information for this entry)
(no taxonomy information for this entry)
(no taxonomy information for this entry)
(no taxonomy information for this entry)
(no taxonomy information for this entry)
(no taxonomy information for this entry)
(no taxonomy information for this entry)
(no taxonomy information for this entry)

Variable modifications: Carbamidomethyl (C),Deamidated (NQ),Oxidation (M)
Cleavage by Trypsin: cuts C-term side of KR unless next residue is P
Sequence Coverage: **40%**

Matched peptides shown in **Bold Red**

```
  1 MDAIKKKMQM LKLDKENAID RAEQAEADKK QAEDRCKQLE EEQQALQKKL
 51 KGTEDEVEKY SESVKDAQEK LEQAEKKATD AEADVASLNR RIQLVEEELD
101 RAQERLATAL QKLEEAEKAA DESERGMKVI ENRAMKDEEK MELQEMQLKE
151 AKHIAEDSDR KYEEVARKLV ILEGELERSE ERAEVAESKC GDLEEELKIV
201 TNNLKSLEAQ ADKYSTKEDK YEEEIKLLEE KLKEAETRAE FAERSVAKLE
251 KTIDDLEDEV YAQKMKYKAI SEELDNALND ITSL
```

Show predicted peptides also

Sort Peptides By ◉ Residue Number ○ Increasing Mass ○ Decreasing Mass

Start - End	Observed	Mr(expt)	Mr(calc)	ppm	Miss	Sequence
13 - 21	1073.6161	1072.6088	1072.5513	54	1	K.LDKENAIDR.A (Ions score 37)
36 - 48	1631.8989	1630.8916	1630.7984	57	1	R.CKQLEEEQQALQK.K Carbamidomethyl (C) (Ions score 85)
38 - 48	1343.7493	1342.7420	1342.6728	52	0	K.QLEEEQQALQK.K (Ions score 57)
77 - 90	1460.8223	1459.8150	1459.7267	61	1	K.KATDAEADVASLNR.R (Ions score 72)
78 - 91	1488.8296	1487.8223	1487.7328	60	1	K.ATDAEADVASLNRR.I (Ions score 33)
91 - 101	1399.8350	1398.8277	1398.7467	58	1	R.RIQLVEEELDR.A (Ions score 16)
92 - 101	1243.7239	1242.7166	1242.6456	57	0	R.IQLVEEELDR.A (Ions score 65)
92 - 105	1727.9966	1726.9893	1726.8849	60	1	R.IQLVEEELDRAQER.L (Ions score 51)
113 - 125	1476.7725	1475.7652	1475.6739	62	1	K.LEEAEKAADESER.G (Ions score 74)
153 - 160	942.4771	941.4698	941.4203	53	0	K.HIAEDSDR.K (Ions score 46)
168 - 178	1298.8428	1297.8355	1297.7605	58	1	R.KLVILEGELER.S (Ions score 82)
169 - 178	1170.7357	1169.7284	1169.6656	54	0	K.LVILEGELER.S (Ions score 76)
190 - 198	1092.5413	1091.5340	1091.4805	49	0	K.CGDLEEELK.I Carbamidomethyl (C) (Ions score 55)
218 - 226	1182.6132	1181.6059	1181.5452	51	1	K.EDKYEEEIK.L (Ions score 35)
252 - 264	1538.8053	1537.7980	1537.7148	54	0	K.TIDDLEDEVYAQK.M (Ions score 85)

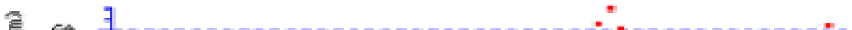

Sample M27

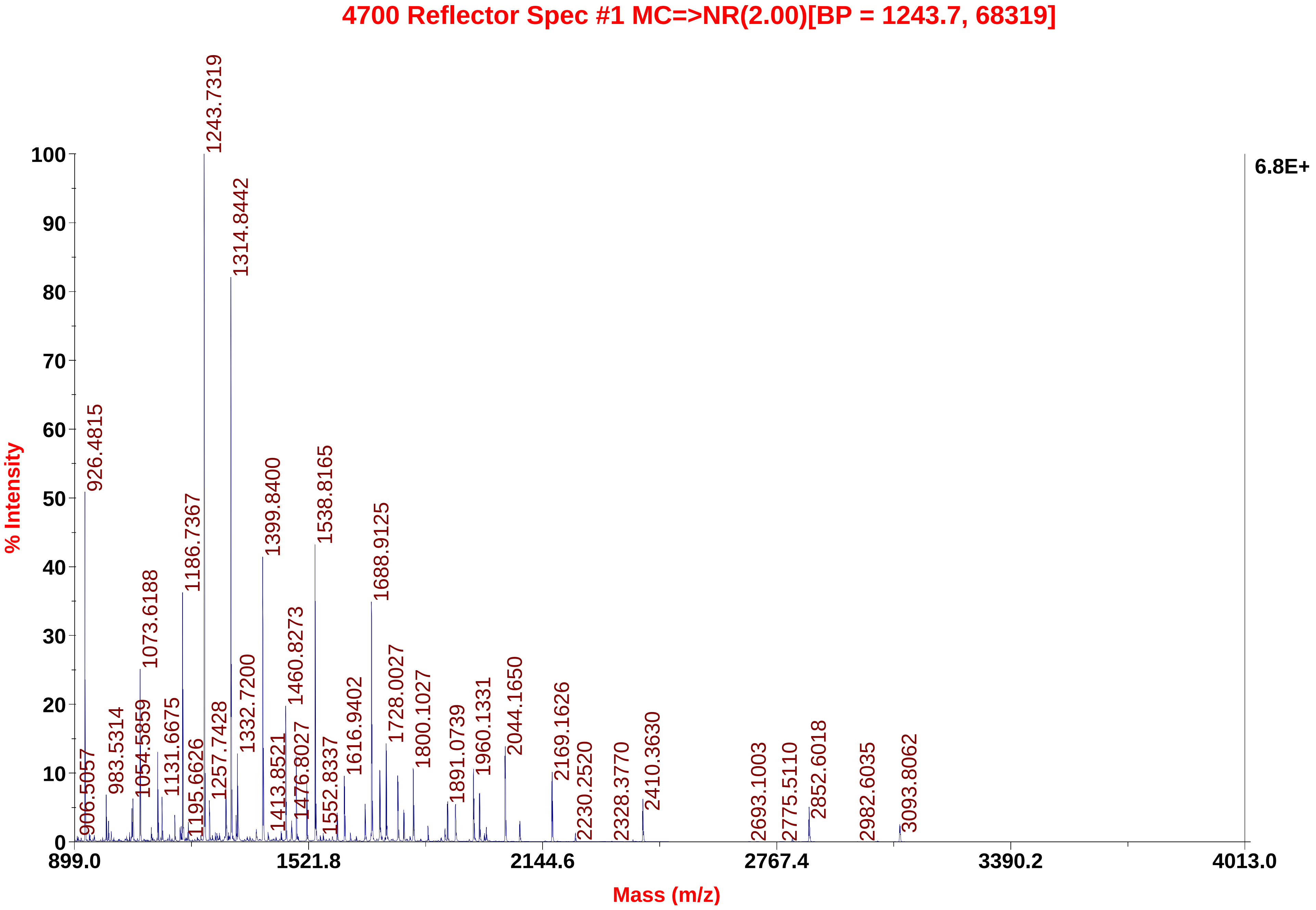
4700 Reflector Spec #1 MC=>NR(2.00)[BP = 1243.7, 68319]
% Intensity
Mass (m/z)
6.8E+
100
90
80
70
60
50
40
30
20
10
0
899.0
1521.8
2144.6
2767.4
3390.2
4013.0
906.5057
926.4815
983.5314
1054.5859
1073.6188
1131.6675
1186.7367
1195.6626
1243.7319
1257.7428
1314.8442
1332.7200
1399.8400
1413.8521
1460.8273
1476.8027
1538.8165
1552.8337
1616.9402
1688.9125
1728.0027
1800.1027
1891.0739
1960.1331
2044.1650
2169.1626
2230.2520
2328.3770
2410.3630
2693.1003
2775.5110
2852.6018
2982.6035
3093.8062

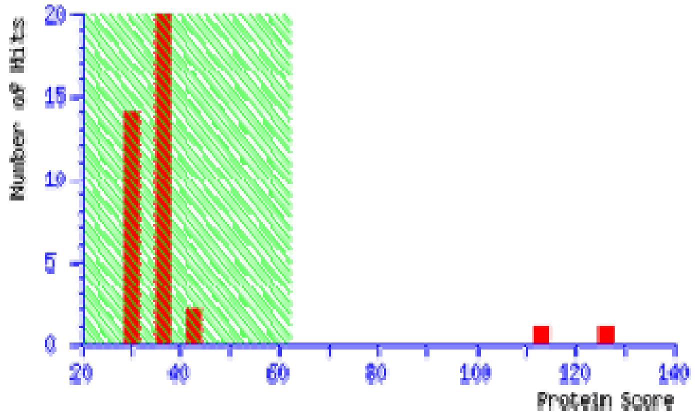

Concise Protein Summary Report

Format As | Concise Protein Summary | Help

Significance threshold p< 0.05 Max. number of hits AUTO

Re-Search All | Search Unmatched

1. TMRBA **Mass:** 32661 **Score:** 126 **Expect:** 2.3e-08 **Matches:** 31
   tropomyosin alpha chain, cardiac and skeletal muscle [validated] - rabbit
   Q8BSH3_MOUSE **Mass:** 32679 **Score:** 111 **Expect:** 7.3e-07 **Matches:** 29
   12 days embryo male wolffian duct includes surrounding region cDNA, RIKEN full-length enriched library, clone:6720477I24
   A39816 **Mass:** 32689 **Score:** 94 **Expect:** 3.8e-05 **Matches:** 28
   tropomyosin 2, fibroblast - rat
   Q99PB8_MOUSE **Mass:** 1525 **Score:** 33 **Expect:** 48 **Matches:** 3
   Adenosine kinase (EC 2.7.1.20) (Fragment).- Mus musculus (Mouse).

Protein View

Match to: **TMRBA** Score: **126** Expect: **2.3e-08**
tropomyosin alpha chain, cardiac and skeletal muscle [validated] - rabbit
Found in search of pmf_K6_129990866210.txt

Nominal mass (M_r): **32661**; Calculated pI value: **4.69**
NCBI BLAST search of TMRBA against nr
Unformatted sequence string for pasting into other applications

Taxonomy: Oryctolagus cuniculus
Links to retrieve other entries containing this sequence from NCBI Entrez:
(no taxonomy information for this entry)
(no taxonomy information for this entry)
(no taxonomy information for this entry)
(no taxonomy information for this entry)
(no taxonomy information for this entry)
(no taxonomy information for this entry)
(no taxonomy information for this entry)
(no taxonomy information for this entry)
(no taxonomy information for this entry)
(no taxonomy information for this entry)

Variable modifications: Carbamidomethyl (C),Deamidated (NQ),Oxidation (M)
Cleavage by Trypsin: cuts C-term side of KR unless next residue is P
Number of mass values searched: **142**
Number of mass values matched: **31**
Sequence Coverage: **69%**

Matched peptides shown in **Bold Red**

```
  1 MDAIKKKMQM LKLDKENALD RAEQAEADKK AAEDRSKQLE DELVSLQKKL
 51 KGTEDELDKY SEALKDAQEK LELAEKKATD AEADVASLNR RIQLVEEELD
101 RAQERLATAL QKLEEAEKAA DESERGMKVI ESRAQKDEEK MEIQEIQLKE
151 AKHIAEDADR KYEEVARKLV IIESDLERAE ERAELSEGKC AELEEELKTV
201 TNNLKSLEAQ AEKYSQKEDK YEEEIKVLSD KLKEAETRAE FAERSVTKLE
251 KSIDDLEDEL YAQKLKYKAI SEELDHALND MTSI
```

Show predicted peptides also

Sort Peptides By ◉ Residue Number ○ Increasing Mass ○ Decreasing Mass

Start – End	Observed	Mr(expt)	Mr(calc)	ppm	Miss	Sequence
13 – 21	1073.6185	1072.6112	1072.5513	56	1	K.LDKENALDR.A
16 – 29	1560.8030	1559.7957	1559.7063	57	1	K.ENALDRAEQAEADK.K Deamidated (NQ)
22 – 30	989.5439	988.5366	988.4825	55	1	R.AEQAEADKK.A
36 – 48	1516.9116	1515.9043	1515.8144	59	1	R.SKQLEDELVSLQK.K
38 – 48	1301.7655	1300.7582	1300.6874	54	0	K.QLEDELVSLQK.K
52 – 65	1597.8563	1596.8490	1596.7519	61	1	K.GTEDELDKYSEALK.D
66 – 76	1273.7345	1272.7272	1272.6561	56	1	K.DAQEKLELAEK.K
78 – 90	1332.7179	1331.7106	1331.6317	59	0	K.ATDAEADVASLNR.R
78 – 91	1488.8333	1487.8260	1487.7328	63	1	K.ATDAEADVASLNRR.I
91 – 101	1399.8395	1398.8322	1398.7467	61	1	R.RIQLVEEELDR.A
92 – 101	1243.7312	1242.7239	1242.6456	63	0	R.IQLVEEELDR.A
92 – 105	1728.0024	1726.9951	1726.8849	64	1	R.IQLVEEELDRAQER.L
137 – 149	1632.9154	1631.9081	1631.8076	62	1	K.DEEKMEIQEIQLK.E
141 – 149	1131.6670	1130.6597	1130.6005	52	0	K.MEIQEIQLK.E
141 – 149	1147.6593	1146.6520	1146.5954	49	0	K.MEIQEIQLK.E Oxidation (M)
141 – 152	1460.8265	1459.8192	1459.7592	41	1	K.MEIQEIQLKEAK.H Deamidated (NQ)
141 – 152	1475.8484	1474.8411	1474.7701	48	1	K.MEIQEIQLKEAK.H Oxidation (M)
141 – 152	1476.8030	1475.7957	1475.7541	28	1	K.MEIQEIQLKEAK.H Deamidated (NQ); Oxidation (M)
153 – 160	926.4818	925.4745	925.4253	53	0	K.HIAEDADR.K
153 – 161	1054.5851	1053.5778	1053.5203	55	1	K.HIAEDADRK.Y
168 – 178	1314.8416	1313.8343	1313.7554	60	1	R.KLVIIESDLER.A
169 – 178	1186.7365	1185.7292	1185.6605	58	0	K.LVIIESDLER.A
169 – 182	1671.9976	1670.9903	1670.8839	64	1	K.LVIIESDLERAEER.A
190 – 198	1120.5782	1119.5709	1119.5117	53	0	K.CAELEEELK.T Carbamidomethyl (C)
190 – 205	1891.0714	1890.0641	1889.9404	65	1	K.CAELEEELKTVTNNLK.S Carbamidomethyl (C)
199 – 213	1648.9156	1647.9083	1647.8203	53	1	K.TVTNNLKSLEAQAEK.Y 3 Deamidated (NQ)
218 – 226	1182.6207	1181.6134	1181.5452	58	1	K.EDKYEEEIK.V
234 – 244	1308.7417	1307.7344	1307.6106	95	1	K.EAETRAEFAER.S
252 – 264	1538.8136	1537.8063	1537.7148	60	0	K.SIDDLEDELYAQK.L
269 – 284	1758.9299	1757.9226	1757.8141	62	0	K.AISEELDHALNDMTSI.-
269 – 284	1774.9274	1773.9201	1773.8091	63	0	K.AISEELDHALNDMTSI.- Oxidation (M)

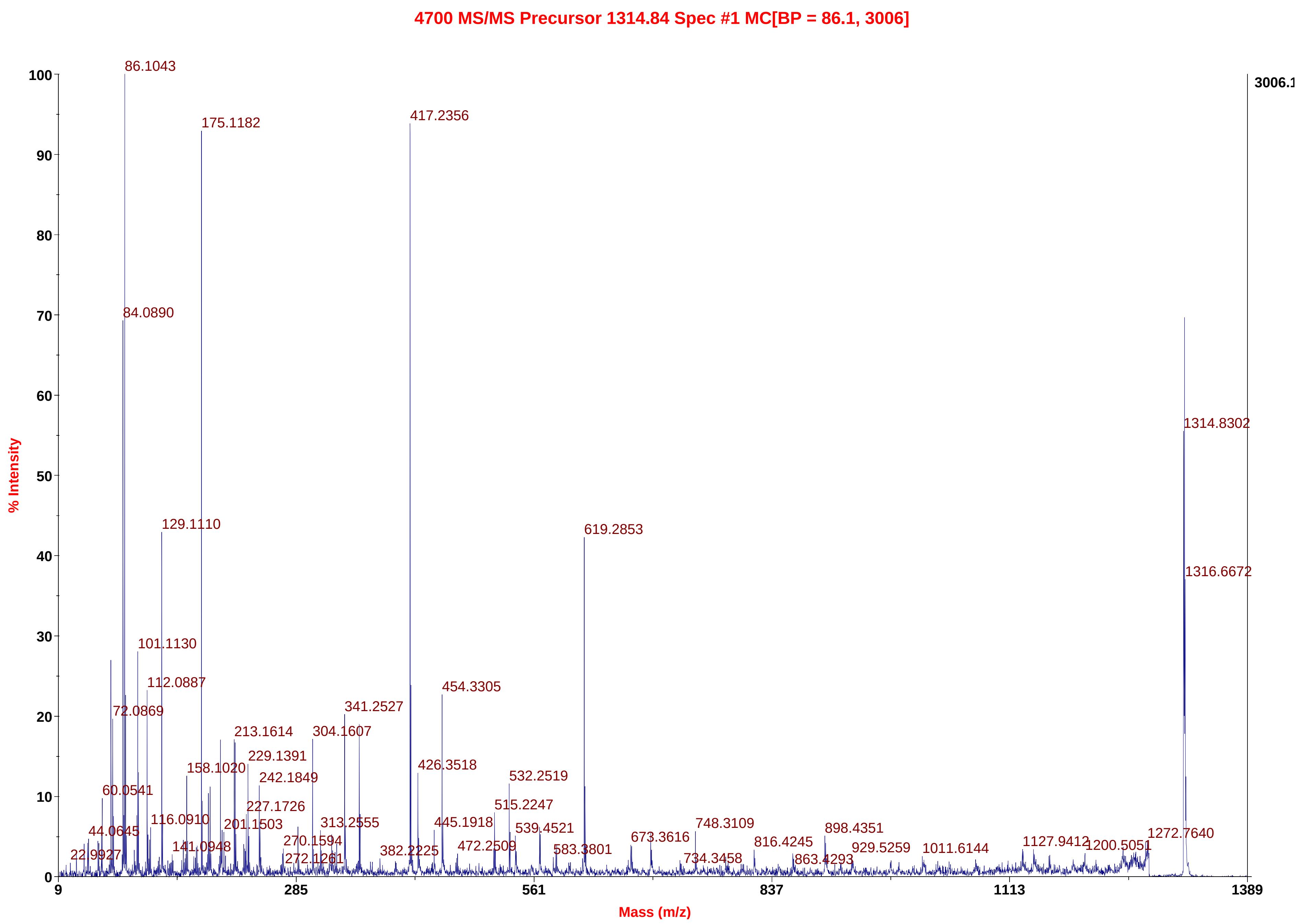

4700 MS/MS Precursor 1314.84 Spec #1 MC[BP = 86.1, 3006]
% Intensity
Mass (m/z)
3006.1
86.1043
84.0890
175.1182
417.2356
129.1110
101.1130
112.0887
72.0869
60.0541
44.0645
22.9927
116.0910
141.0948
158.1020
201.1503
213.1614
229.1391
242.1849
227.1726
270.1594
272.1261
304.1607
313.2555
341.2527
382.2225
426.3518
445.1918
454.3305
472.2509
515.2247
532.2519
539.4521
583.3801
619.2853
673.3616
734.3458
748.3109
816.4245
863.4293
898.4351
929.5259
1011.6144
1127.9412
1200.5051
1272.7640
1314.8302
1316.6672

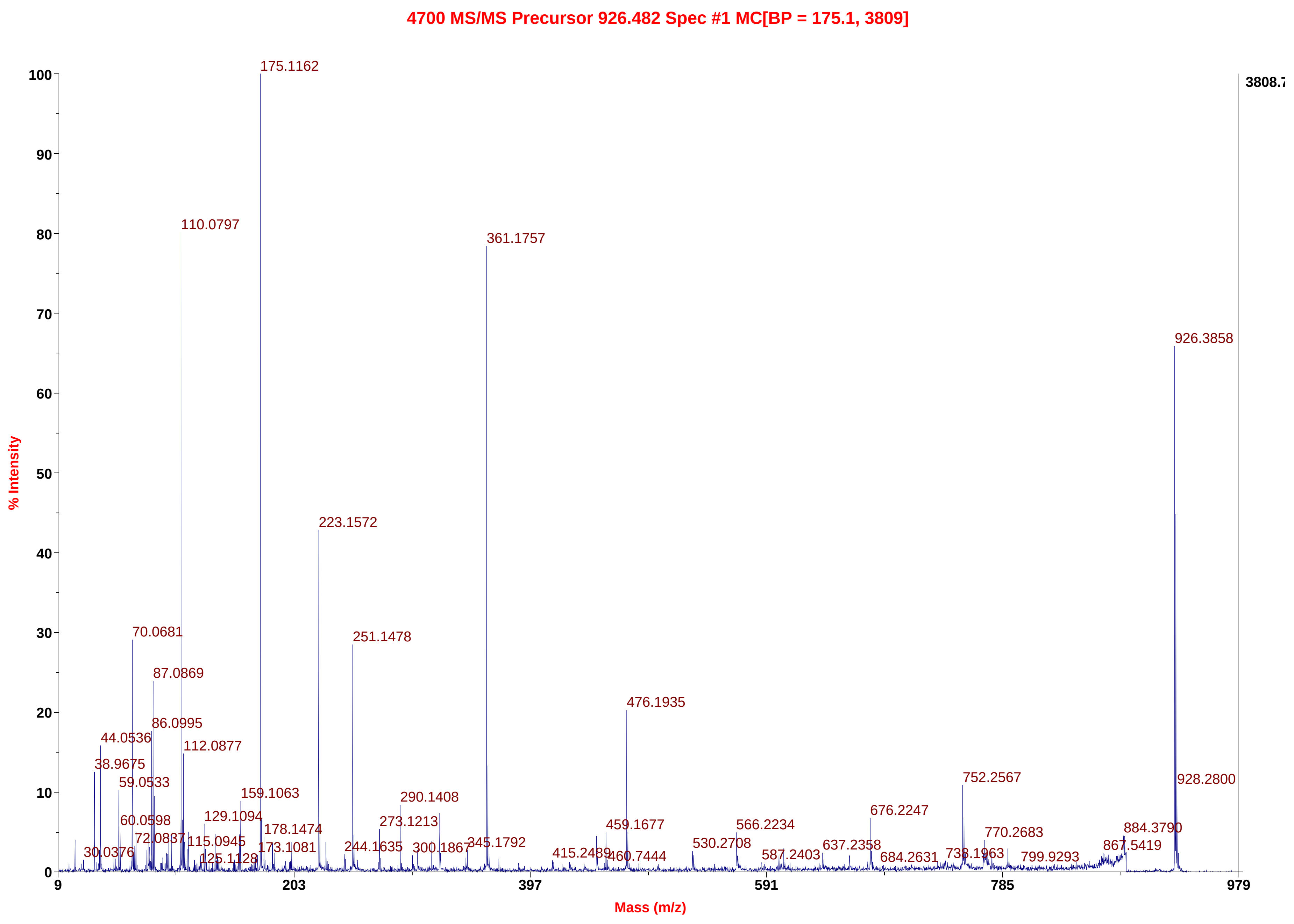
4700 MS/MS Precursor 926.482 Spec #1 MC[BP = 175.1, 3809]
% Intensity
Mass (m/z)
3808.7
175.1162
110.0797
361.1757
926.3858
223.1572
70.0681
251.1478
87.0869
476.1935
86.0995
44.0536
112.0877
38.9675
59.0533
159.1063
290.1408
752.2567
928.2800
676.2247
129.1094
60.0598
273.1213
459.1677
566.2234
770.2683
884.3790
178.1474
72.0837
115.0945
345.1792
530.2708
637.2358
867.5419
30.0376
173.1081
244.1635
300.1867
415.2489
460.7444
587.2403
738.1963
799.9293
684.2631
125.1128

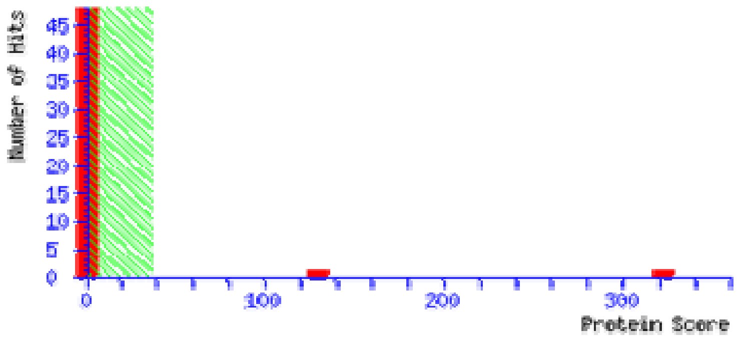

Peptide Summary Report

[Format As] Peptide Summary — Help

Significance threshold p< 0.05 — Max. number of hits AUTO

Standard scoring ○ MudPIT scoring ● — Ions score or expect cut-off 0 — Show sub-sets 0

Show pop-ups ● Suppress pop-ups ○ — Sort unassigned Decreasing Score — Require bold red ☐

[Select All] [Select None] [Search Selected] ☐ **Error tolerant**

1. TMRBA **Mass:** 32661 **Score:** 323 **Matches:** 12(11) **Sequences:** 12(11) **emPAI:** 5.50
tropomyosin alpha chain, cardiac and skeletal muscle [validated] - rabbit
☐ Check to include this hit in error tolerant search

	Query	Observed	Mr(expt)	Mr(calc)	ppm	Miss	Score	Expect	Rank	Unique	Peptide
☑	1	926.4818	925.4745	925.4253	53.2	0	61	0.00017	1	U	K.HIAEDADR.K
☑	2	1073.6185	1072.6112	1072.5513	55.9	1	49	0.0032	1	U	K.LDKENALDR.A
☑	3	1120.5782	1119.5709	1119.5117	52.9	0	46	0.0079	1	U	K.CAELEEELK.T + Carbamidomethyl (C)
☑	4	1186.7365	1185.7292	1185.6605	58.0	0	44	0.0046	1	U	K.LVIIESDLER.A
☑	5	1243.7312	1242.7239	1242.6456	63.1	0	50	0.0018	1		R.IQLVEEELDR.A
☑	6	1314.8416	1313.8343	1313.7554	60.1	1	67	1.6e-05	1	U	R.KLVIIESDLER.A
☑	7	1399.8395	1398.8322	1398.7467	61.2	1	24	0.62	1		R.RIQLVEEELDR.A
☑	8	1460.8265	1459.8192	1459.7267	63.4	1	80	2.7e-06	1		K.KATDAEADVASLNR.R
☑	9	1488.8333	1487.8260	1487.7328	62.7	1	50	0.0025	1		K.ATDAEADVASLNRR.I
☑	10	1538.8136	1537.8063	1537.7148	59.6	0	85	9.4e-07	1	U	K.SIDDLEDELYAQK.L
☑	14	1728.0024	1726.9951	1726.8849	63.8	1	37	0.039	1		R.IQLVEEELDRAQER.L
☑	15	1758.9299	1757.9226	1757.8141	61.7	0	89	3.7e-07	1	U	K.AISEELDHALNDMTSI.-

Protein View

Match to: **TMRBA** Score: **323**
tropomyosin alpha chain, cardiac and skeletal muscle [validated] - rabbit
Found in search of ppw_K6_129990872610.txt

Nominal mass (M_r): **32661**; Calculated pI value: **4.69**
NCBI BLAST search of TMRBA against nr
Unformatted sequence string for pasting into other applications

Taxonomy: Oryctolagus cuniculus
Links to retrieve other entries containing this sequence from NCBI Entrez:
(no taxonomy information for this entry)
(no taxonomy information for this entry)
(no taxonomy information for this entry)
(no taxonomy information for this entry)
(no taxonomy information for this entry)
(no taxonomy information for this entry)
(no taxonomy information for this entry)
(no taxonomy information for this entry)
(no taxonomy information for this entry)
(no taxonomy information for this entry)

Variable modifications: Carbamidomethyl (C),Deamidated (NQ),Oxidation (M)
Cleavage by Trypsin: cuts C-term side of KR unless next residue is P
Sequence Coverage: **33%**

Matched peptides shown in **Bold Red**

```
  1 MDAIKKKMQM LKLDKENALD RAEQAEADKK AAEDRSKQLE DELVSLQKKL
 51 KGTEDELDKY SEALKDAQEK LELAEKKATD AEADVASLNR RIQLVEEELD
101 RAQERLATAL QKLEEAEKAA DESERGMKVI ESRAQKDEEK MEIQEIQLKE
151 AKHIAEDADR KYEEVARKLV IIESDLERAE ERAELSEGKC AELEEELKTV
201 TNNLKSLEAQ AEKYSQKEDK YEEEIKVLSD KLKEAETRAE FAERSVTKLE
251 KSIDDLEDEL YAQKLKYKAI SEELDHALND MTSI
```

Show predicted peptides also

Sort Peptides By ◉ Residue Number ○ Increasing Mass ○ Decreasing Mass

Start - End	Observed	Mr(expt)	Mr(calc)	ppm	Miss	Sequence
13 - 21	1073.6185	1072.6112	1072.5513	56	1	K.LDKENALDR.A (Ions score 49)
77 - 90	1460.8265	1459.8192	1459.7267	63	1	K.KATDAEADVASLNR.R (Ions score 80)
78 - 91	1488.8333	1487.8260	1487.7328	63	1	K.ATDAEADVASLNRR.I (Ions score 50)
91 - 101	1399.8395	1398.8322	1398.7467	61	1	R.RIQLVEEELDR.A (Ions score 24)
92 - 101	1243.7312	1242.7239	1242.6456	63	0	R.IQLVEEELDR.A (Ions score 50)
92 - 105	1728.0024	1726.9951	1726.8849	64	1	R.IQLVEEELDRAQER.L (Ions score 37)
153 - 160	926.4818	925.4745	925.4253	53	0	K.HIAEDADR.K (Ions score 61)
168 - 178	1314.8416	1313.8343	1313.7554	60	1	R.KLVIIESDLER.A (Ions score 67)
169 - 178	1186.7365	1185.7292	1185.6605	58	0	K.LVIIESDLER.A (Ions score 44)
190 - 198	1120.5782	1119.5709	1119.5117	53	0	K.CAELEEELK.T Carbamidomethyl (C) (Ions score 46)
252 - 264	1538.8136	1537.8063	1537.7148	60	0	K.SIDDLEDELYAQK.L (Ions score 85)
269 - 284	1758.9299	1757.9226	1757.8141	62	0	K.AISEELDHALNDMTSI.- (Ions score 89)
